# Genomic architecture of endogenous ichnoviruses reveals distinct evolutionary pathways leading to virus domestication in parasitic wasps

**DOI:** 10.1186/s12915-020-00822-3

**Published:** 2020-07-24

**Authors:** Fabrice Legeai, Bernardo F. Santos, Stéphanie Robin, Anthony Bretaudeau, Rebecca B. Dikow, Claire Lemaitre, Véronique Jouan, Marc Ravallec, Jean-Michel Drezen, Denis Tagu, Frédéric Baudat, Gabor Gyapay, Xin Zhou, Shanlin Liu, Bruce A. Webb, Seán G. Brady, Anne-Nathalie Volkoff

**Affiliations:** 1 IGEPP, Agrocampus Ouest, INRAE, Université de Rennes 1, 35650 Le Rheu, France; 2 Université Rennes 1, INRIA, CNRS, IRISA, F-35000 Rennes, France; 3 Department of Entomology, National Museum of Natural History, Smithsonian Institution, 10th and Constitution Avenue NW, Washington, DC, 20560-0165 USA; 4 Data Science Lab, Office of the Chief Information Officer, Smithsonian Institution, 10th and Constitution Avenue NW, Washington, DC, 20560-0165 USA; 5 DGIMI, INRAE, University of Montpellier, 34095 Montpellier, France; 6 Institut de Recherche sur la Biologie de l’Insecte, UMR 7261, CNRS - Université de Tours, UFR des Sciences et Techniques, Parc de Grandmont, Tours, France; 7 Institut de Génétique Humaine, CNRS, University of Montpellier, 34396 Montpellier, France; 8 Commissariat à l’Energie Atomique (CEA), Institut de Génomique (IG), Genoscope, 2 rue Gaston Crémieux, BP5706, 91057 Evry, France; 9 Department of Entomology, China Agricultural University, Beijing, 100193 People’s Republic of China; 10 China National GeneBank, BGI-Shenzhen, Shenzhen, Guangdong Province 518083 People’s Republic of China; 11 Department of Entomology, University of Kentucky, Lexington, USA

**Keywords:** Endogenous virus architecture, Polydnavirus, Parasitoid wasp, Koinobiont, Campopleginae, IVSPERs

## Abstract

**Background:**

Polydnaviruses (PDVs) are mutualistic endogenous viruses inoculated by some lineages of parasitoid wasps into their hosts, where they facilitate successful wasp development. PDVs include the ichnoviruses and bracoviruses that originate from independent viral acquisitions in ichneumonid and braconid wasps respectively. PDV genomes are fully incorporated into the wasp genomes and consist of (1) genes involved in viral particle production, which derive from the viral ancestor and are not encapsidated, and (2) proviral segments harboring virulence genes, which are packaged into the viral particle. To help elucidating the mechanisms that have facilitated viral domestication in ichneumonid wasps, we analyzed the structure of the viral insertions by sequencing the whole genome of two ichnovirus-carrying wasp species, *Hyposoter didymator* and *Campoletis sonorensis*.

**Results:**

Assemblies with long scaffold sizes allowed us to unravel the organization of the endogenous ichnovirus and revealed considerable dispersion of the viral loci within the wasp genomes. Proviral segments contained species-specific sets of genes and occupied distinct genomic locations in the two ichneumonid wasps. In contrast, viral machinery genes were organized in clusters showing highly conserved gene content and order, with some loci located in collinear wasp genomic regions. This genomic architecture clearly differs from the organization of PDVs in braconid wasps, in which proviral segments are clustered and viral machinery elements are more dispersed.

**Conclusions:**

The contrasting structures of the two types of ichnovirus genomic elements are consistent with their different functions: proviral segments are vehicles for virulence proteins expected to adapt according to different host defense systems, whereas the genes involved in virus particle production in the wasp are likely more stable and may reflect ancestral viral architecture. The distinct genomic architectures seen in ichnoviruses versus bracoviruses reveal different evolutionary trajectories that have led to virus domestication in the two wasp lineages.

## Background

Parasites and their hosts are involved in a continual coevolutionary arms race, with hosts evolving defense mechanisms and parasites developing strategies to overcome them [1, 2]. Identifying the genomic basis of such adaptations is crucial to understand the evolutionary dynamics of host-parasite interactions [3], with cycles of adaptation and counter-adaptation often resulting in complex biological strategies with far-reaching consequences at the genomic level. The use of endogenous viruses by parasitoid wasps provides an example of how complex host-parasite interactions can lead to specific genomic adaptations.

Parasitoid wasps are among the most successful groups of parasitic organisms, potentially comprising several hundred thousand species and playing major ecological roles in terrestrial ecosystems [4]. While the adult wasps are free-living, during their immature stages they develop as parasites of other arthropods, eventually killing their host. Parasitoid wasps have diverse biological strategies, and many groups develop inside a host that remains active after being parasitized (koinobiont endoparasitoids). In order to survive within a developing organism, some lineages of parasitic wasps have evolved strategies to manipulate their host by employing mutualistic viruses from the Polydnaviridae (PDVs) family. PDV particles are produced exclusively within the calyx region of the ovary during female wasp pupation and adulthood. Particles enclose a packaged genome composed of several circular molecules, or “segments,” of double-stranded DNA. Mature virions are secreted into the oviduct and transferred into the host, usually a caterpillar, during oviposition. Once inside the host, PDVs do not replicate but express genes that induce profound physiological alterations in the parasitized host, such as impairment of the immune response or developmental alterations, which are required for successful development of the wasp larva [5–9].

Two groups of PDVs have been reported, associated with the hyperdiverse sister wasp families Braconidae (bracoviruses) and Ichneumonidae (ichnoviruses) [10, 11]. In both cases, PDVs persist in all cells of the wasp as integrated sequences (provirus), allowing the vertical transfer of the PDV genetic material [12, 13]. Bracoviruses and ichnoviruses differ in their morphology and gene content but share the life cycle described above [14]. Each PDV group derives from the genomic integration of a different virus during the evolution of braconid and ichneumonid lineages [15, 16]: the independent origin of these two groups of PDVs illustrates an astonishing example of convergent evolution. Bracoviruses, found in wasps from the “microgastroid complex” lineage, result from the integration of a nudivirus genome [17]; about 50,000 species belonging to six braconid subfamilies are estimated to carry these mutualistic viruses [18, 19]. Ichnoviruses descend from the integration of a virus of unknown origin [16] and are found in two distantly related ichneumonid subfamilies, the Campopleginae and the Banchinae [20], which together comprise over 3860 species [21]. Whether ichnoviruses in these two subfamilies result from a single virus integration or two independent events involving related viruses remains unclear [20]. At least one campoplegine, *Venturia canescens*, has lost its PDVs and instead produces virus-like particles devoid of DNA deriving from a third event of virus integration that occurred in this lineage [22].

Following integration, viral sequences retained in the wasp genome underwent rearrangements leading to the present genomic architecture of bracoviruses and ichnoviruses. The integrated PDV genomes include two types of functional components [15, 23, 24]. The first type, hereinafter called “proviral segments,” corresponds to sequences that serve as templates for the PDV segments packaged within the particles. Proviral segments exhibit direct repeated sequence at their extremities (“direct repeat junctions,” or DRJs) that allow homologous recombination and generation of the circular molecule [25, 26]. The packaged DNA segments of several PDVs have been sequenced, revealing that their content differs between bracoviruses and ichnoviruses (reviewed in [6]). PDV segments encode virulence genes that will be expressed in the parasitoid’s host; no typical virus replication genes have been identified in PDV packaged genomes. Although considered as part of the PDV genome, they probably consist of a mosaic of sequences from various organismic backgrounds (ancestral virus, insect host, and others still unknown) [27]. The second type of PDV endogenous sequences, hereafter “replication genes,” are those involved in the production of the PDV particles. They are expressed exclusively in the wasp calyx cells during the process of PDV production [16, 17, 28]), but in contrast with viral segments, are not packaged in the viral particles.

Knowledge on PDV genomic architecture is currently focused on bracoviruses, based on whole genome sequencing of PDV-carrying braconid wasps [16, 24]. In braconid genomes, most viral segments are located in clusters which may comprise up to 18 segments [15, 23, 29] organized in tandem arrays and separated by regions of intersegmental DNA that are not encapsidated. This organization leads to a particular mode of replication of the bracovirus segments in the wasp calyx cells. They are first amplified within replication units encompassing several segments [30–32]. A concatemer is then excised and finally sub-divided in individual segments by homologous recombination between the direct repeats (DRJ) present at each end of the segment. All bracovirus DRJs contain a conserved tetramer AGCT, shown to be the site where the segment is circularized [26, 30, 33]. On the other hand, the genomic architecture of ichnoviruses is still poorly known: in the absence of PDV-carrying ichneumonid genomes, there is no information on the distribution and organization of the segments within the wasp genome. Note that direct repeats have also been reported in ichnoviruses [25, 34], but this finding was restricted to a few segments and so far there is no evidence of a conserved motif in ichnovirus DRJs, neither if segment excision relies on similar or different mechanisms in the two groups of PDV.

The data are slightly more consistent for the two types of PDVs as regards genomic architecture of the replication genes. In braconids, particle production relies on 100 endogenous genes highly similar to nudivirus genes, including a large proportion of the core structural genes seen in nudiviruses [23]. Half of the nudiviral genes identified within the genome of the braconids *Cotesia congregata* and *Microplitis demolitor* are located in a “nudiviral cluster;” the other nudiviral genes are dispersed and isolated in the wasp genome [15, 23, 31]. Genes involved in ichnovirus particle formation, but lacking similarity with known virus genes, have been identified in two ichneumonids belonging to two subfamilies, the campoplegine *Hyposoter didymator* [16, 35] and the banchine *Glypta fumiferanae* [20]. Analysis of bacterial artificial chromosomes (BAC) for these two species revealed that the approximately 40 replication genes that have been identified are organized in three large clusters, named “Ichnovirus Structural Protein Encoding Regions” (IVSPER) [16, 20]. However, the manner in which IVSPERs are distributed within the wasp genome is unknown, and other IVSPERs may remain undiscovered.

Elucidating how viral insertions are distributed and organized in the wasp genomes is required both to understand the machinery that produces PDVs and to determine the mechanisms that have facilitated the “domestication” of viruses by parasitic wasps. While a clear picture of this organization has started to emerge for bracoviruses as shown above, there is still little information on how PDV sequences are distributed in the genome of ichnovirus-carrying wasps. For example, it is not known whether ichnovirus proviral segments are clustered like bracovirus ones, and, if so, whether there are conserved recombination motifs analogous to those in bracoviruses allowing excision/circularization of individual segments. It is also unclear whether ichnovirus replication genes are all clustered in the few loci identified so far, and whether the position and gene composition of IVSPERs are conserved across wasp species within the same clade. Since ichnoviruses and bracoviruses derive from the integration of unrelated viral ancestors, comparing their genomic characteristics should provide insights regarding the selection forces that have operated on the domestication of the two types of ancestral viruses.

To address these questions, we sequenced the genomes of two ichneumonid wasps from the campoplegine subfamily, *Hyposoter didymator* and *Campoletis sonorensis*. Both species are parasitoids of larvae of owlet moths (Lepidoptera, Noctuidae) and are associated with endogenous ichnoviruses. The packaged PDV genomes produced in the two species (*H. didymator* ichnovirus or “HdIV”, and *C. sonorensis* ichnovirus or “CsIV”) have been previously sequenced [36, 37], showing they share homologous genes. High-quality genome assemblies were obtained for both species, allowing a clear picture of ichnoviruses genomic architecture and comparisons with that of bracoviruses. The availability of the first ichnovirus-carrying wasp genomes revealed a differing genomic architecture between ichnoviruses and bracoviruses. Ichnoviral loci include a large number of isolated proviral segments scattered along the genome scaffolds while replication genes are all clustered in half a dozen IVSPERs. The observed differences between ichnovirus and bracovirus gene content and genomic architectures suggest that viral domestication may have followed substantially different evolutionary paths in the two wasp families.

## Results

The genomes of the two campoplegine ichneumonid wasps were sequenced using high-throughput Illumina HiSeq technology. The datasets were then assembled using either Supernova v.2.1.1 [38] or Platanus assembler v1.2.1 [39], depending on the species (see “Methods” section). Assembled genomes were then annotated automatically using Augustus v3.3 [40] for *Campoletis sonorensis* and BRAKER1 v1.10 [41] for *Hyposoter didymator* (see “Methods” section). The annotated whole genomes are the first ever produced for ichnovirus-carrying ichneumonids.

### Shared features of *Hyposoter didymator* and *Campoletis sonorensis* genomes

The draft assembled genome of *H. didymator* consisted of 199 Mb in 2591 scaffolds ranging in size from 1 kbp to 15.7 Mbp, with a scaffold N50 of 3.999 Mbp and a contig N50 of 151,312 bp (Table 1). The *C. sonorensis* assembled genome consisted of 259 Mb in 11,756 scaffolds with sizes ranging from 400 bp to 6.1 Mbp, with an N50 of 725,399 bp and a contig N50 of 315,222 bp (Table 1). For both ichneumonid species, G+C content was similar to most other parasitoid species for which genomes are available (between 33.6 and 39.5%) (Table 1).

**Table 1:** Statistics for *Hyposoter didymator* and *Campoletis sonorensis* assembled genomes. Summary statistics are compared to other selected parasitoid genomes. Assemblathon2 [42] was used to calculate metrics of genome assemblies

Family	Ichneumonid			Braconid			Pteromalid
Species	Hyposoter didymator*	Campoletis sonorensis*	***Venturia canescens***^**§**^	Microplitis demolitor*	***Fopius arisanus***^**§**^	***Diachasma alloeum***	***Nasonia vitripennis***
Number of scaffolds	2591	11,756	62,001	1794	1042	3968	6098
Total length (Mbp)	198.7	258.9	237.8	241.2	153.6	388.8	295.8
Longest scaffold (Mbp)	15.7	6.1	0.85	7.15	5.5	6.61	33.57
Scaffold N50 (Mbp)	4.00	0.73	0.114	1.14	0.98	0.65	0.90
Median scaffold size (nt)	1941	3200	233	2621	12,305	4372	2037
Contig N50 (bp)	151,312	315,222	15,077	14,499	59,408	50,453	18,840
%N	1.35%	0.40%	1.70%	14.65%	8.24%	6.16%	19.33%
GC (%)	39.5%	37.2%	39.33%	25.99%	35.42%	36.09%	33.65%
Reference	/	/	[22]	[31]	[43]	[44]	[45]

*Species carrying PDV; ^**§**^species that produces virus-like particles (devoid of DNA)

Transposable elements (TE) represented 15.09% of the *H. didymator* and 17.38% of the *C. sonorensis* genomes. The major TE groups (LTR, LINE, SINE retrotransposons, and DNA transposons) contribute to 54% of the total TE coverage in *H. didymator* and up to 79% in *C. sonorensis* (Additional file 1: Table S1). The two wasp species differed by the number of class 1 elements (retrotransposons), which was higher in *C. sonorensis* (46% of the TEs) compared to *H. didymator* genome (24% of the TEs).

**Fig. 1:**
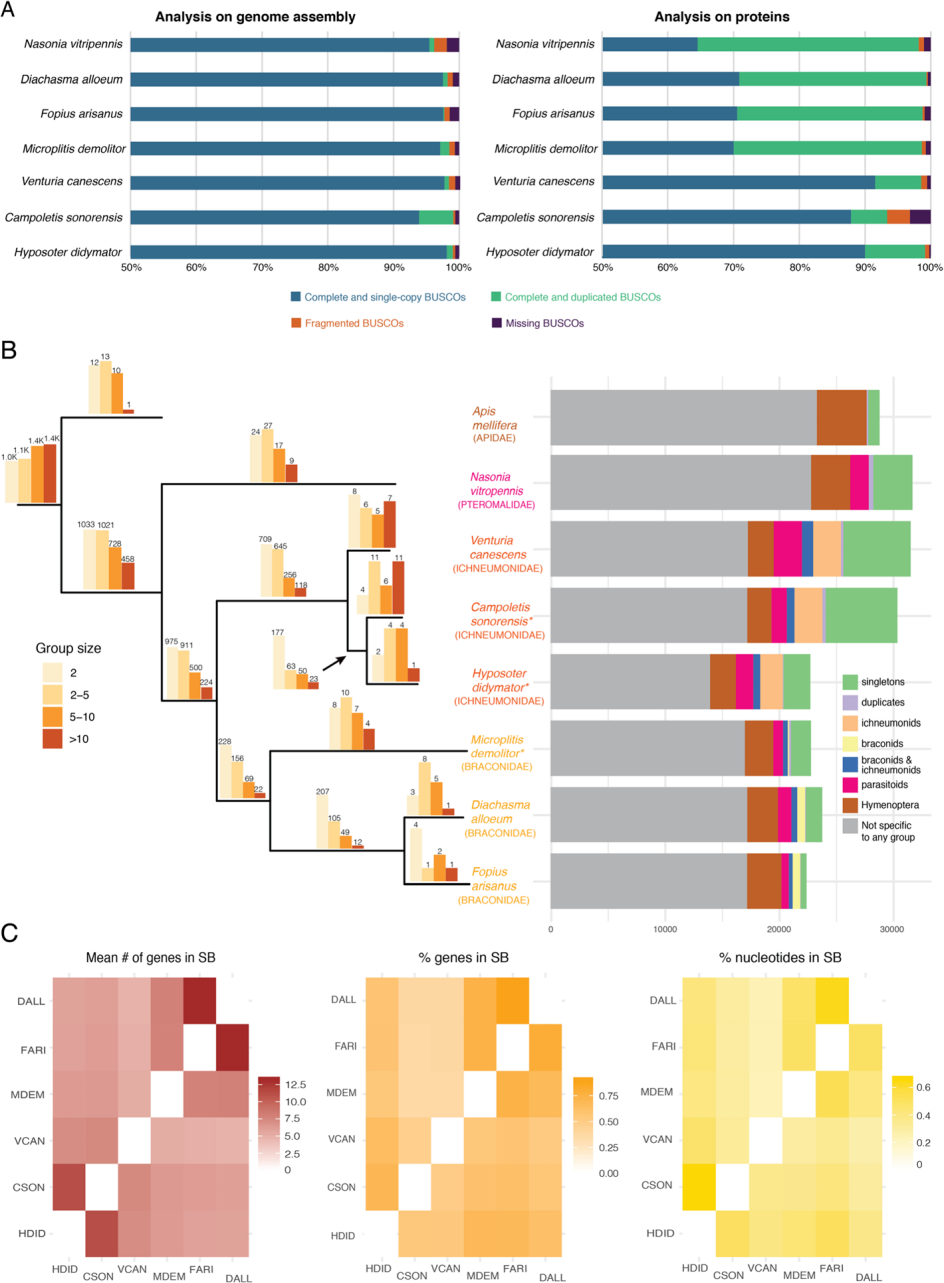
Genomic features of *Campoletis sonorensis* and *Hyposoter didymator* genomes. **a** BUSCO analysis of parasitoid wasp genomes (Insecta protein set with 1658 proteins). On the left, results using the genome assemblies; on the right, results using the predicted protein set. **b** Orthogroups analysis. *Left panel*: Barplots above each branch of the phylogenic tree indicate the number of orthogroups specific to each species or group of species; the color of the bar indicates the size range of the corresponding orthogroups. Phylogenetic tree was constructed by aligning the complete genomes with Cactus ([49]), converting the resulting HAL alignment to MAF and then to multi fastas with the requirement of full alignment (all taxa present); fasta files were then concatenated into a single matrix (620 kb) and used in a maximum likelihood analysis with RAxML [50] with 1000 fast bootstrap replicates. Asterisks indicate the species carrying polydnaviruses. *Right panel*: Number of genes for each species that were (i) specific to the species and present either as singletons or duplicates, (ii) present in ichneumonids, (iii) present in braconids, (iv) present in both ichneumonids and braconids, (v) present in all parasitoids, and (vi) present in all hymenoptera. **c** Heatmaps indicating, for each species pair, the mean number (#) of genes in synteny blocs (SB), the percentage (%) of genes in SBs, and the size of the genome (% nucleotides) in SBs. HDID, *Hyposoter didymator* (ichneumonid, with PDV); CSON, *Campoletis sonorensis* (ichneumonid, with PDV); VCAN, *Venturia canescens* (ichneumonid); MDEM, *Microplitis demolitor* (braconid, with PDV); FARI, *Fopius arisanus* (braconid); DALL, *Diachasma alloeum* (braconid)

Automatic gene annotation for *H. didymator* (for which RNA-seq datasets were available) and for *C. sonorensis* (for which no RNA-seq dataset was available) yielded 18,119 and 21,915 transcripts, respectively (Table 2). These two genome assemblies and annotations are available at BIPAA website [46, 47]. Although different software packages were used for gene prediction, the two species have similar gene annotation statistics, except for transcript size, which was longer in *H. didymator*, and which also showed a higher predicted intron size (Table 2). Benchmarking Universal Single-Copy Orthologs (BUSCO) [48] analyses indicated a high level of completeness of the two genome assemblies and annotations, with 99% of the BUSCO Insecta protein set (1658 proteins) identified as complete sequences (Fig. 1a).

**Table 2:** Gene annotation statistics for *Hyposoter didymator* and *Campoletis sonorensis* assembled genomes. Statistics are given for transcripts, exons, introns, and coding sequences (CDS)

	*Hyposoter didymator*	*Campoletis sonorensis*
Transcript number	18,119	21,915
Total transcript size (nt)	95,868,518	68,669,265
Mean transcript size (nt)	5291	3133
Median transcript size (nt)	2428	1934
Total exon number	98,639	93,590
Mean exon number	5.4	4.3
Median exon number	4	3
Total exon size (nt)	28,900,964	27,144,418
Mean exon size (nt)	292	290
Median exon size (nt)	186	192
Total intron size (nt)	66,540,946	41,453,172
Mean intron size (nt)	826	578
Median intron size (nt)	245	296
Total CDS size (nt)	28,900,964	27,144,418
Mean CDS size (nt)	1595	1239
Median CDS size (nt)	1090	806

Orthologous gene families were identified with Orthofinder by computing each pair’s similarity among the proteomes of different parasitoid wasps, either harboring polydnaviruses (the braconid *Microplitis demolitor*) or not (the ichneumonid *Venturia canescens*, the braconids *Fopius arisanus* and *Diachasma alloeum*, and the pteromalid *Nasonia vitripennis*). For *H. didymator* and *C. sonorensis*, a total of ~ 10,000 orthogroups were identified (Fig. 1b; Additional file 2: Table S2). The orthogroups included a large majority of the *H. didymator* (87.1%) and *C. sonorensis* (71.4%) genes. Among those, only a small portion corresponded to species-specific orthogroups: 11 orthogroups for *H. didymator* (69 genes) and 32 for *C. sonorensis* (288 genes). The number of shared orthogroups decreases with the increasing evolutionary distance among the other species (higher in the campoplegine ichneumonid *Venturia canescens* to lower in the dipteran *Drosophila melanogaster*; Additional file 2: Table S3). Among the orthogroups shared by *H. didymator* and *C. sonorensis* genes, 313 were specific to these two ichnovirus-carrying species (Fig. 1b; Additional file 2: Table S4), representing 875 genes for *C. sonorensis* and 509 genes for *H. didymator*.

Global synteny analysis revealed a number of syntenic blocks between the two genomes, enabling evaluation of the genomic reorganization between the two species, even using fragmented assemblies. When comparing the *C. sonorensis* and *H. didymator* genomes, the mean number of genes per synteny block obtained was 11.2, one of the highest pairwise values for the evaluated species (Additional file 3: Table S5), including one other campoplegine species, *Venturia canescens* (mean number of 7 genes per synteny block). The percentage of regions in syntenic blocks shared between *C. sonorensis* and *H. didymator* compared to the complete genome size was 67% for *H. didymator* and 50% for *C. sonorensis* (Fig. 1c). The percentage of genes that are located in syntenic blocks was 71% for *H. didymator* and 54% for *C. sonorensis* (Fig. 1c). The high pairwise values show that global collinearity of *H. didymator* and *C. sonorensis* genomes is well conserved, as expected by their close evolutionary relationship.

### The two campoplegine genomes include numerous and dispersed ichnovirus loci

**Fig. 2:**
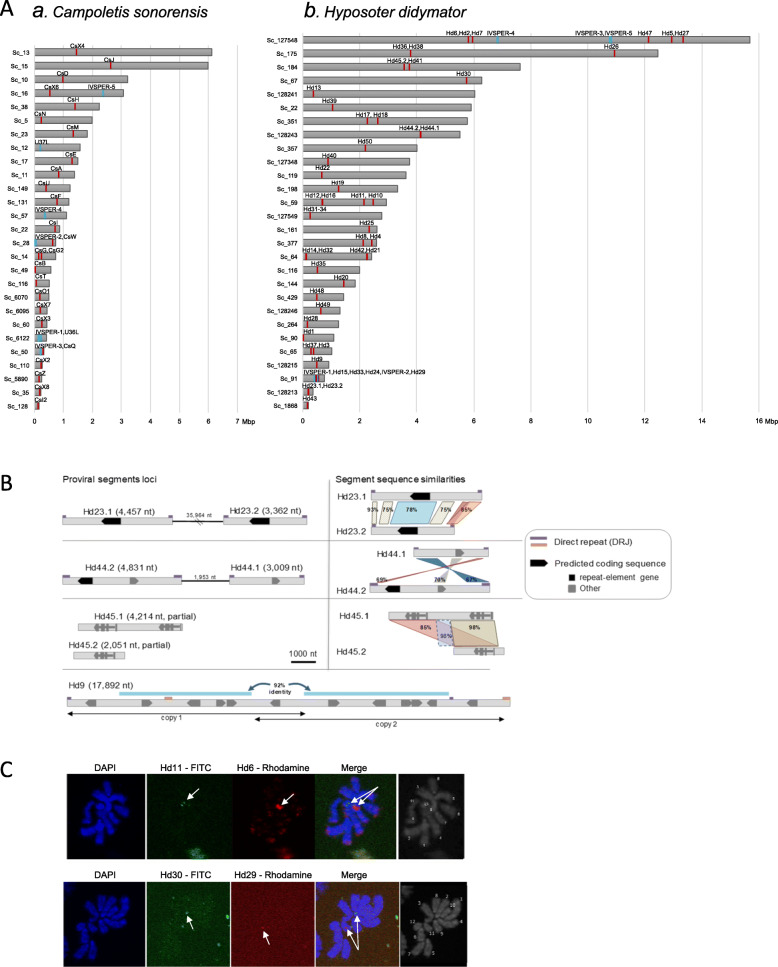
Distribution of ichnovirus sequences within *Campoletis sonorensis* and *Hyposoter didymator* genomes. **A** Schematic representation of ichnovirus sequences within wasp genome scaffolds. (a) *C. sonorensis* scaffolds containing viral loci. *C. sonorensis* ichnovirus (CsIV) segments are named CsA to CsX8. Segments CsP and CsL, located in short scaffolds, are not shown. IVSPER-1 to IVSPER-5 corresponds to clusters of replication genes; U36L and U37L to isolated replication genes. **(**b) *H. didymator* scaffolds containing viral loci. *H. didymator* ichnovirus (HdIV) segments are named Hd1 to Hd51. Segments Hd45.1, Hd46, and Hd51, located in short scaffolds, are not shown. IVSPER-1 to IVSPER-5 corresponds to clusters of replication genes; the isolated replication gene U37, located in a short scaffold, is not shown. Complete scaffold list available in Additional file 4: Table S5. **B** Segments duplicated in *H. didymator* genome. Segments Hd23 (Genbank# KJ586309.1), Hd44 (Genbank# KJ586285.1) and Hd45 (Genbank# KJ586284.1), described as part of the packaged HdIV genome in [36], have two copies (named Hd(n).1 and Hd(n).2) that are either in the same scaffold (Hd23.1 and Hd23.2, Hd44.1, and Hd44.2) but in different insertion sites or in two different scaffold (Hd45.1 and Hd45.2). Nucleotide percentage identity between the two related segment sequences is given on the right part of the figure. By contrast, Hd9 (Genbank# KJ586324.1), initially described as a separate segment, is located in a genomic locus composed of a tandemly duplicated sequence (“copy 1” and “copy 2” in the diagram). **C** FISH on *H. didymator* chromosomes using BAC genomic clones containing HdIV segments. Upper panel shows hybridization using the probes containing segments Hd11 (labeled with FITC) and Hd6 (labeled with rhodamine); lower panel the probes containing viral segments Hd30 (labeled with FITC) and Hd29 (labeled with rhodamine). Each of the probes hybridized with a different *H. didymator* chromosome: Hd11 hybridized with chromosome #12, Hd6 to a medium-sized chromosome (potentially #5), Hd30 with chromosome #2 and Hd29 with chromosome #11

To analyze the relationship between the wasps and their endogenous viruses, we identified the location of the viral sequences in those genomes using blast searches with available ichnovirus sequences. In the assembled *C. sonorensis* genome, a total of 35 scaffolds, ranging in size from 2.3 kbp to more than 6 Mbp, contained *C. sonorensis* ichnovirus (CsIV) sequences (Fig. 2A; Additional file 4: Table S6). Within these scaffolds, 40 viral loci were identified, corresponding either to viral segments, to clusters of replication genes (IVSPERs), or to isolated replication genes. A total of 31 proviral segments were recognized, with sizes varying from 6.4 to 23.2 kbp (Additional file 4: Table S6). They included all segments reported in a previous study [37] except for two (Table 3). Eight previously uncharacterized segments, named CsX1 to CsX8, were additionally identified (Table 3). Two short scaffolds each contained a repeat element gene (i.e., a member of a gene family known to be encoded by ichnovirus segments) and were considered as probable additional viral segments (Table 3). Altogether, the *C. sonorensis* genome contained 33 loci corresponding to CsIV proviral segments. Finally, and for the first time in *C. sonorensis*, we identified 48 replication genes located in six different scaffolds, corresponding to five clusters (named Cs_IVSPER-1 to Cs_IVSPER-5) and two isolated genes (named IVSP_U36L and IVSP_U37L). The IVSPERs in *C. sonorensis* varied in size from 8.6 to 33.3 kbp and contained from 3 to 19 genes (Additional file 4: Table S6; Additional file 5: Table S7).

**Table 3:** Summary of the number of viral loci identified in the genomes of *Campoletis sonorensis* and *Hyposoter didymator*, in comparison with data available in NCBI database

	*Campoletis sonorensis*	*Hyposoter didymator*
Number of segments in NCBI ^a^	25	50
Number of segments in genome	31	54
NCBI segments not found in genome	2 (CsA2, CsK)	0
Merged segments (compared to NCBI)^b^	0	6 (Hd2, Hd11, Hd17, Hd20, Hd26, Hd31–34)
Duplicated segments (compared to NCBI) ^c^	0	3 (Hd23, Hd44, Hd45)
Number of newly identified segments	8 (CsX1 to CsX8)	6 (Hd46 to Hd51)
“Isolated” segment genes (short scaffolds)	2	1
Total number of segment loci in genome	**33**	**55**
Number of IVSPERs in NCBI ^a^	0	3
Number of IVSPERs in genome	5	5
NCBI IVSPERs not found in genome	na	0
Newly identified IVSPERs	5	2
“Isolated” IVSPER genes	2	1
Total number of IVSPER loci in genome	**7**	**6**

^a^Numbers of segments and IVSPER deposited in NCBI and available before this study

^b^*H. didymator* viral loci corresponding to two segments formerly deposited in NCBI as distincts

^c^*H. didymator* viral loci found in two copies in the wasp genome

In the *H. didymator* assembled genome, a total of 60 proviral loci were identified (Fig. 2A); they were located in 32 scaffolds ranging in size from 1.5 kbp to over 15 Mbp (Additional file 4: Table S6). Loci corresponding to all the previously described *H. didymator* ichnovirus (HdIV) segments [36] were identified in the wasp genome (Table 3). When the first HdIV packaged genome was sequenced [36] some HdIV circular molecules shared part of their sequences (i.e., segments Hd2a and Hd2b, Hd11a and b, Hd17a and b, Hd20a and b, Hd26a and b, Hd31 and Hd34). Mapping on the *H. didymator* genome revealed that the six segments pairs actually each co-localized at the same proviral locus (Additional file 6: Fig. S1). Thanks to the availability of the genome, we found three HdIV segments present in two copies that were not identical but clearly recognizable as duplications (Fig. 2B); two had copies in the same scaffold (Hd23.1 and Hd23.2; Hd44.1 and Hd44.2), one in two different scaffolds (Hd45.1 and Hd45.2). Finally, Hd9 was tandemly duplicated at a single locus (Fig. 2B). In addition, six previously uncharacterized segments were identified (named Hd46 to Hd51). Altogether, 54 HdIV proviral segments were found, ranging in size from 2.0 to 17.9 kbp (Additional file 4: Table S6). Finally, new replication genes were identified in the *H. didymator* genome. In addition to the three IVSPERs previously described [16], two novel clusters (names Hd_IVSPER-4 and Hd_IVSPER-5) and one isolated gene (IVSP_U37) were identified, making up a total of 54 predicted replication genes present in *H. didymator* genome. All except one were organized in five IVSPERs, varying in size from 1.6 to 26.6 kbp (Additional file 4: Table S6; Additional file 5: Table S7).

Analysis of the genome assemblies revealed a large number of widely dispersed viral loci, separated by wasp sequences with a median size of 115.1 kb for viral fragments located on the same scaffold (Additional file 7: Table S8, Fig. S2). To independently confirm dispersion of ichnovirus proviral segment sequences, we carried out fluorescent in situ hybridization in *H. didymator*, using genomic clones including viral segment loci as probes (Fig. 2C). Four probes were used, containing Hd11, Hd6, Hd30, and Hd29 segments, all corresponding to different genomic scaffolds. Each of the probes hybridized with a different chromosome, indicating that HdIV segments are indeed widely dispersed across the *H. didymator* genome.

To assess whether dispersion of the viral loci could have been mediated during genome evolution by transposable elements, distribution of TEs was investigated in the regions surrounding the proviral loci. The analysis of the families of transposable elements in the regions surrounding *H. didymator* proviral sequences (Additional file 8: Table S9) did not reveal any particular enrichment that might suggest a role of TEs in the dispersion of the ichnovirus sequences in the wasp genomes.

### Ichnovirus proviral segments harbor direct repeats with variable architecture and multiple putative excision sites

PDV segments are circularized by homologous recombination between direct repeats (DRJs) located at each end of the proviral segment. This mechanism has been verified for bracoviruses, where the DRJs contain a conserved tetramer which is the potential excision site [30]. For ichnoviruses, although the same mechanism is assumed to take place, based on the analysis of the only two proviral segments for which sequence was available [25, 34], it was unknown whether or not ichnovirus DRJs contain a conserved motif, and if so, whether this motif was similar to that described for bracoviruses. To investigate if the excision process for ichnovirus proviral segments could potentially rely on mechanisms similar to those described for bracoviruses, we searched for direct repeats at the extremities of the newly identified HdIV and CsIV proviral segments. Flanking direct repeated sequences were found for the large majority of HdIV and CsIV loci. All HdIV segment loci, except for Hd45.1 and Hd45.2, had DRJs, which varied significantly in size, ranging from 69 to 949 bp (Additional file 9: Table S10). Similarly, most CsIV segments (25 of 32) were flanked by DRJs, which ranged in size from 99 bp to as much as 1132 bp (Additional file 8: Table S9). The main finding that emerged from the availability of dozens of ichnovirus DRJs is their segment specificity in terms of length and sequence. The number of direct repeats also showed high variability across proviral sequences (Additional file 9: Table S10; Additional file 10: Fig. S3) even though the majority of the HdIV (28) and CsIV (19) segments contained a single repeated sequence, one copy located on their right and left ends. A few HdIV and CsIV segments also contained internal repeats of the same sequence, potentially allowing the generation of more than one related circular molecule by recombination between different pairs of DRJ copies (nested segments). Other ichnovirus proviral segments (21 HdIV segments, but only one CsIV segment) contained two different repeated sequences, named DRJ1 and DRJ2, present in two or more copies. Note that the segments initially described as distinct [36] but which were found at the same locus are segments flanked either by a DRJ1 or by a DRJ2 (see Additional file 6: Fig. S1). As an example, the proviral segment Hd2 displays two different types of DRJs, which consequently allows generation of two segments that share part of their sequence: segment Hd2a, generated through recombination between DRJ1; and segment Hd2b, through recombination between DRJ2 (Additional file 6: Fig. S1). Occurrence of several repeats differing in sequence and in position suggests that a mixture of overlapping and/or nested segments may be generated by homologous recombination in this context.

With the aim of assessing whether a conserved excision site motifs is embedded in ichnovirus DRJ sequences, as described for bracovirus segments, we used the “regulatory DNA motif identification and analyses” (DMINDA) webserver [51] using all 99 DRJs identified in the two wasp species. From all the motifs found, seven occurred in 70% of all 99 DRJs, and two motifs occurred at least once in 98% of the analyzed DRJs (Additional file 10: Table S11). However, analysis of the *H. didymator* genome revealed this motif had the same chance of occurring in the DRJs as in the rest of the wasp genome (Additional file 10: Table S12). Hence, circularization of ichnovirus segments probably does not rely on the presence of a conserved nucleotide motif as in bracoviruses.

**Fig. 3:**
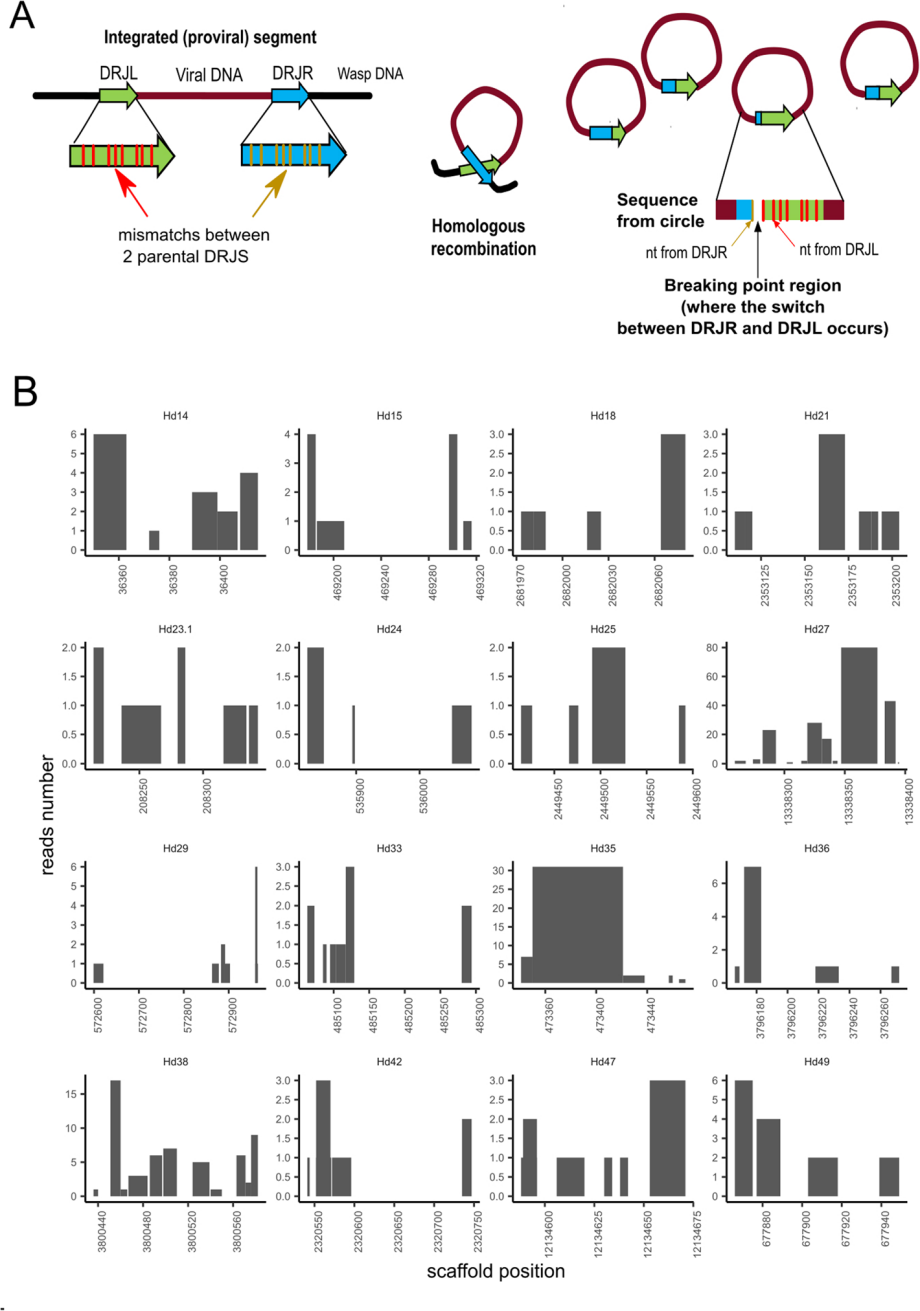
Segment DRJ variability in terms of excision sites in *Hyposoter didymator*. **a** Schematic representation of the homologous recombination between the two DRJs flanking the proviral sequence (left DRJL and right DRJR ends) to produce the circular molecule (segment) containing one recombined DRJ sequence. When excision sites are located at different positions in the DRJ, segments differing in their recombined DRJ sequence are generated. Excision occurs more frequently at some positions, resulting in different relative amounts of each isoform. On the right, rationale of the algorithm developed to identify the “break points.” Mapping of the segment sequence (DRJsegment) with the two parental DRJs, which differ in their sequences (nucleotide (nt) mismatches), allows identification of the regions where the switch from one parental DRJ to the other has occurred (in the diagram, between the first and second mismatch). **b** Prediction of putative recombination break points in *H. didymator* DRJs. Each graph corresponds to the left copy of the DRJ for a given segment (indicated below each graph). The *X*-axis is the position in the scaffold. The *Y*-axis indicates the number of reads (obtained from sequencing of the packaged circular DNA molecules) confirming that the circle has been recombined between these two positions, based on the observed mismatches at both end of the segment for each read. We observed between 1 and 80 reads per breakpoint region according to the analyzed segment

In the absence of a known conserved motif, we searched for potential excision sites allowing to generate ichnovirus circular molecules in *H. didymator* using an algorithm developed for this purpose, DrjBreakpointFinder (see “Methods”). The rationale is illustrated in Fig. 3a. To identify the excision site, or “breakpoint,” in a given recombined DRJ sequence (i.e., the circular molecule), the latter was compared to sequence of the two copies present at each end of the segments in their linear integrated form. These two copies exhibit several sequence differences (Additional file 9: Table S10), allowing identification of the excision site in the corresponding recombined DRJ sequence with certain accuracy, depending on the divergence between the parental DRJ copies. Thus, two sets of *H. didymator* segment sequences (circular DNA molecules extracted from *H. didymator* ichnovirus particles) were analyzed using DrjBreakpointFinder. Automated analysis of this large set revealed that excision could occur in different sites within a same DRJ, although some positions were more frequent than others for a given DRJ (Fig. 3b). This unexpected variability in the excision site position was confirmed for a subset of eight segments by manual comparative analysis of segment isoforms sequenced using Sanger technology (Additional file 11).

### Proviral sequences serving as templates for ichnovirus packaged genome show features specific to each campoplegine species

To understand evolutionary changes experienced by proviral segments in the two different wasp species, we analyzed their characteristics, gene contents, and positions within the two genomes. Altogether, ichnovirus segment loci represent a similar total size for the two species (307.1 kbp for HdIV and 314.1 kbp for CsIV). The total sizes of the endogenous viruses are slightly underestimated since two HdIV segments (Hd1 and Hd45.1) and five CsIV segments (CsV, CsX3, CsX4, CsX5, and CsX7) were only partially identified due to genome fragmentation (see Additional file 4: Table S6 for details). However, general segment features differed between the two wasp species. Prior sequencing of packaged genomes had already highlighted differences between the viral segments in *C. sonorensis* (CsIV) and *H. didymator* (HdIV) [36, 37]. CsIV particles enclose only half the number of segments compared to HdIV particles, and CsIV viral segments are on average longer than HdIV segments. Accordingly, the number of proviral loci identified in the *H. didymator* genome is higher (*n* = 54) than in the *C. sonorensis* genome (*n* = 33), while CsIV proviral segments are on average longer than HdIV ones (Additional file 12: Fig. S4). Regarding gene number, 111 were predicted for CsIV segment genes whereas a total of 152 genes were predicted in the HdIV segments (Table 4; Additional file 5: Table S7). Within-segment gene composition varied considerably between the two species. Both encapsidated genomes contain members of the ichnovirus-conserved multimembers families (repeat element genes, vankyrins, vinnexins, cys-motif, and N-genes), but in variable number (Table 4). HdIV contains more viral innexins, whereas CsIV more viral ankyrins and repeat element genes (Table 4). In addition, each of the genomes enclosed a number of genes (encoding hypothetical proteins; Table 4) specific either to *C. sonorensis* or to *H. didymator*. Overall, ichnovirus segments are characterized by gene content that is quite specific to each one of the two wasp species.

**Table 4:** Comparative segment gene content for the ichnoviruses carried by the campoplegine wasps *Hyposoter didymator* (HdIV) and *Campoletis sonorensis* (CsIV)

IV gene family	HdIV	CsIV
*Repeat element genes*	38	51
*Viral innexins*	17	6
*Viral ankyrins*	10	16
*Cys-motif proteins*	9	13
*Polar-residue-rich proteins*	5	nd
*N-genes*	3	3
Total	82	89
Hypothetical proteins	70	22
TOTAL	**152**	**111**

The high collinearity in gene order observed between the genomes of *H. didymator* and *C. sonorensis* made it possible to assess if the viral segments were located in the same genomic regions in the two species. We compared the sequences flanking viral insertions in *H. didymator* with their syntenic genomic regions in *C. sonorensis* (Additional file 12: Fig. S5). For the large majority of syntenic blocks containing an HdIV segment, there was no viral locus in the corresponding *C. sonorensis* block (Additional file 12: Fig. S5 a). Two exceptions were found for two segments present in the same *H. didymator* scaffold (scaffold 351). *H. didymator* segment Hd18 was flanked by the same wasp genes as *C. sonorensis* IVSPER-5 (Additional file 12: Fig. S5 b), and *H. didymator* segment Hd17 was inserted in the same wasp genomic region as *C. sonorensis* segment CsZ, a region that seemed to have undergone rearrangements like gene duplications (Additional file 12: Fig. S5 c). *H. didymator* Hd17 and *C. sonorensis* CsZ both contain genes from the repeat element genes family, suggesting the two segments may have arisen from the same ancestral sequence, already inserted in this wasp locus, prior to wasp species diversification.

### The ichnovirus machinery retained in wasp genomes (IVSPERs) is well conserved

To understand the evolutionary changes experienced by the IVSPER clusters, we analyzed their gene contents, and positions in the wasp genomes. A total of 45 different predicted IVSPER gene families were identified in the genomes of *H. didymator* and *C. sonorensis* (Additional file 5: Table S7). The majority (35, or 78%) are shared by both wasp species, with a number of gene copies within a multigene family that may differ between the two wasp species. Among the 36 different genes/gene families (corresponding to a total of 48 genes) identified in the *C. sonorensis* IVSPERs, only one had no homolog in the *H. didymator* genome. This gene, Gf_U27L, has similarities (BlastP *e*-value 1E−31) with an IVSPER gene described in the banchine *G. fumiferanae* [20]. Among the 44 distinct gene families identified in *H. didymator* IVSPERs (corresponding to 54 genes), nine were not detected in the *C. sonorensis* genome. Of these, three genes (U29, U32, and U33), transcribed in *H. didymator* ovarian tissue based on our transcriptome data (see “Methods” section), had not been previously characterized in *H. didymator*. They were classified as IVSPER genes because of their clustering with other conserved IVSPER genes (within IVSPER-4).

**Fig. 4:**
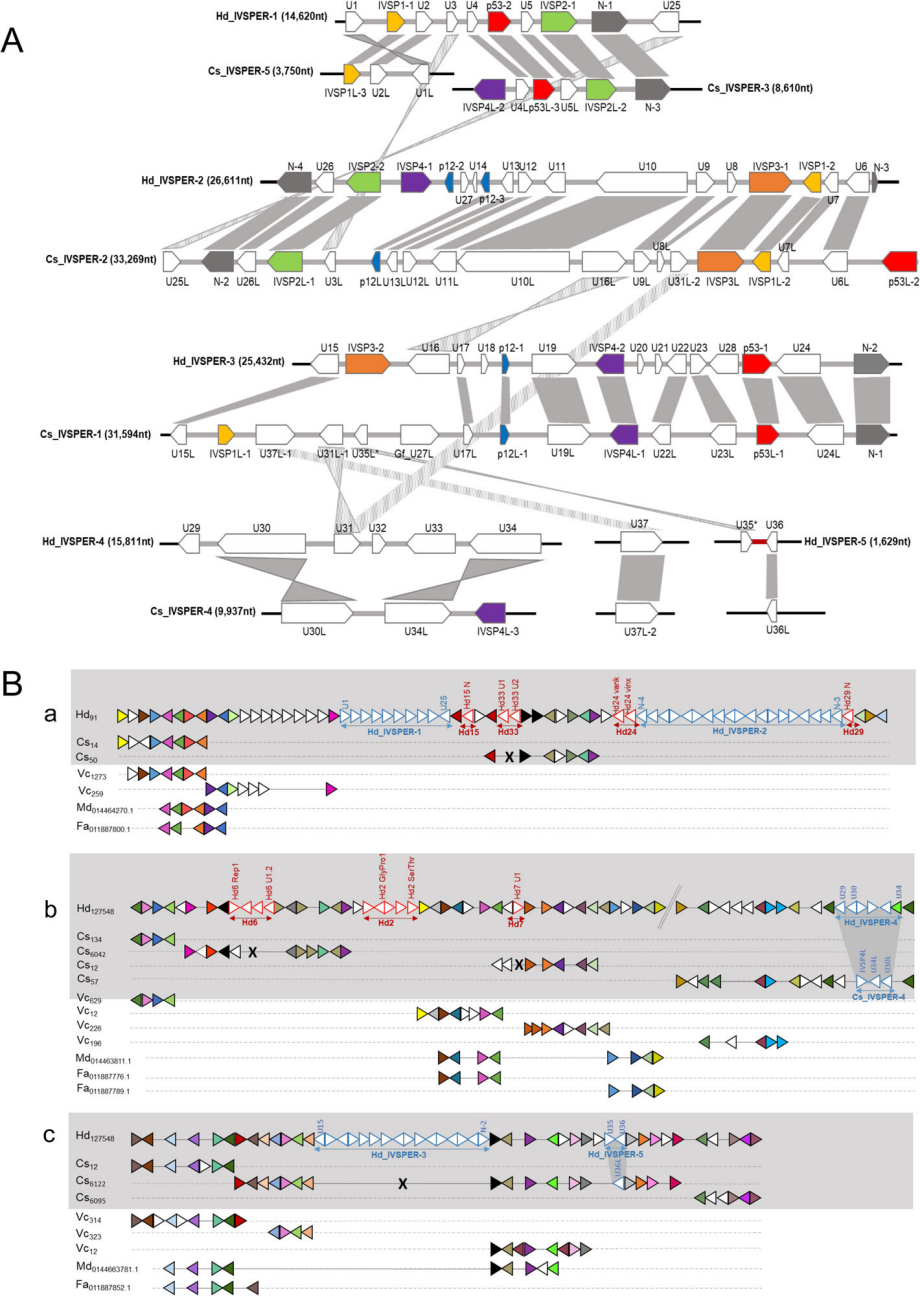
Comparative analysis of *Campoletis sonorensis* and *Hyposoter didymator* IVSPERs. **A** Synteny between the IVSPERs identified in *H. didymator* (Hd) and *C. sonorensis* (Cs) genomes. **B** Synteny of *H. didymator* genomic regions containing IVSPERs compared with *C. sonorensis* and other parasitoid genomes. (a) Synteny for *H. didymator* genomic region containing IVSPER-1 and IVSPER-2 (genes from HD016092 to HD016153); no *C. sonorensis* scaffold corresponded to the *H. didymator* IVSPER insertion sites. (b) Synteny for *H. didymator* genomic region containing IVSPER-4 (genes from HD001703 to HD001771); *H. didymator* IVSPER-4 and *C. sonorensis* IVSPER-4 are inserted in the same genomic environment. **c** Synteny for *H. didymator* genomic region containing IVSPER-3 and IVSPER-5 (genes from HD002066 to HD002111); in the region where *H. didymator* IVSPER-3 is inserted, there is conservation in gene order compared to *C. sonorensis* but no viral insertion; conversely, *H. didymator* IVSPER-5 and *C. sonorensis* IVSPER-5 are inserted in the same genomic environment. *H. didymator* genes from HD010503 to HD010526. Hd: *Hyposoter didymator*; Cs: *Campoletis sonorensis*; Vc: *Venturia canescens* (ichneumonid that has lost the ichnovirus [22]); Md: *Microplitis demolitor* (braconid with a bracovirus); Fa: *Fopius arisanus* (braconid with virus-like particles). Numbers following the species name correspond to scaffold number for Hd, Cs, and Vc, NCBI project codes for Md and Fa. Triangles within genomic regions correspond to predicted genes; triangles of the same color correspond to orthologs; white triangles are singletons or orphan genes. For better visualization, the name of the gene is indicated only for some viral (in red for segments, in blue for IVSPERs) genes. See Additional file 13: Table S13, for *H. didymator* genes list

The IVSPERs of the two wasp species show high synteny (Fig. 4A). Regions with conserved gene order are shared, for instance, between Hd_IVSPER-1 and Cs_IVSPER-5, Hd_IVSPER-2 and Cs_IVSPER-2, or Hd_IVSPER-3 and Cs_IVSPER-1. However, there are also rearrangements, inversions, and deletions when the two species are compared. The highest number of rearrangements involves Cs_IVSPER-1 and Cs_IVSPER-2, with homologs of Cs_IVSPER-1 and Cs_IVSPER-2 genes dispersed in several different *H. didymator* IVSPERs. Overall, and in contrast with proviral segments, IVSPER are well conserved in terms of gene content and order when comparing the two campoplegine species, suggesting this organization may be required for the IVSPER genes biological function, i.e., to produce the virus particles.

To assess if the IVSPER loci were located in the same genomic regions in *H. didymator* and *C. sonorensis*, we compared the genomic regions containing viral insertions in *H. didymator* with their syntenic genomic regions in *C. sonorensis* (Fig. 4B). For three *H. didymator* loci (Hd_IVSPER-1 and -2, U37), no corresponding *C. sonorensis* scaffold was found (Fig. 4B, a), making the comparison inconclusive. On the other hand, comparison was possible for the remaining *H. didymator* IVSPER loci. For *H. didymator* IVSPER-3, there was no viral locus in the corresponding *C. sonorensis* block (Fig. 4B, c). Conversely, Hd_IVSPER-4 and Hd_IVSPER-5, which are both IVSPERs containing a reduced number of genes, are flanked by orthologous wasp genes compared to the Cs_IVSPER-4 and U36L loci in *C. sonorensis* genome (Fig. 4B, b and c), indicating that these IVSPER loci have a conserved genomic location in the two genomes. Hence, as observed for viral segments, IVSPERs seem quite mobile in the wasp genomes, except for two small gene clusters that remained in a similar, putatively ancestral, location. The high within-cluster conservation, even between distantly related wasp species, suggests that they may move as a whole within the wasp genomes by mechanisms still to be elucidated.

## Discussion

### Dispersal of proviral loci through the wasp genome

The genome assemblies obtained allowed us to perform a comprehensive mapping of the viral inserts into the wasp genome. A major finding is the highly dispersed distribution of the ichnovirus proviral segments. All but two of the 32 CsIV segments loci are located in different scaffolds. Similarly, half of the 54 HdIV viral segments are located in different scaffolds; those located in the same scaffold are usually separated by relatively large, sometimes megabase-long portions of wasp genome. This dispersion was confirmed by FISH experiments for *H. didymator*, showing that viral loci are distributed across multiple chromosomes. Organization of ichnovirus proviral segments is therefore quite different from that of bracoviruses. Bracovirus segments are generally fewer compared to ichnoviruses, and they are for the most part clustered in a single locus, named the viral macro-locus [15, 31, 52]. To illustrate this, the *Microplitis demolitor* genome contains 26 proviral segments distributed in only eight loci, with 14 segments located at the same locus [31]. In contrast, ichnovirus genomes consist of a series of single viral segments scattered throughout the wasp genome. As no enrichment of transposable elements surrounding the ichnovirus segments has been observed, their dispersal in the wasp genome may result from reintegration events, multiple genomic rearrangements events, or yet another still unknown mechanism. In addition, based on the lack of conservation in their genomic position when comparing *H. didymator* and *C. sonorensis*, ichnovirus viral segment diversification and dispersion may result from transposition of individual viral sequence while, for bracoviruses, segment multiplication occurs mainly by duplication of large areas [15].

### Conserved IVSPER structure and its significance

As previously described, the proviral segments are the template for the DNA molecules that are packaged and transferred to the parasitized host, whereas replication genes, clustered in IVSPERs, are involved in the production of the virus particle. Until now, replication genes were known solely for one campoplegine wasp, *H. didymator* [16], and a banchine species, *G. fumiferanae* [20]. Our study discovered replication genes in another campoplegine wasp genome, *C. sonorensis*, and revealed a conserved IVSPER architecture when comparing the two campoplegine species. In both *H. didymator* and *C. sonorensis* genomes, the majority of the recognized replication genes are clustered. Indeed, only two isolated replication genes were identified in the *C. sonorensis* genome and only one in the *H. didymator* genome. A large portion of the IVSPER genes are shared between *H. didymator* and *C. sonorensis* and arranged in a conserved order. Furthermore, most genes found in campoplegines are also present in the banchine *G. fumiferanae*, though the gene order is less conserved in this case [6].

The two ichneumonid subfamilies that harbor PDVs, Banchinae and Campopleginae, do not form a monophyletic group [53, 54], and ichnoviruses are not reported for other subfamilies in the same lineage [55]. Hence, phylogenetic evidence would suggest separate origins for PDVs in Ichneumonidae. On the other hand, a high proportion of IVSPER genes are shared between campoplegine and banchine wasps, including the D5 primase-like and DEDXhelicase-like first described in the banchine *G. fumiferanae* (corresponding to U37 and U34 respectively in *H. didymator*). This similarity would suggest a common viral ancestor, or related viral ancestors for campoplegine and banchine ichnoviruses. Better understanding of the evolutionary trajectories of IVSPERs across ichneumonid lineages requires additional sequencing of banchine wasp genomes, as well as a thorough screening of species from other subfamilies for the presence of endogenous viruses.

### Specific features of bracoviruses and ichnoviruses genome architecture

Our study provides the opportunity to make direct comparisons of viral composition between ichneumonid and braconid genomes. The genomes of campoplegine wasps associated with ichnoviruses contain numerous dispersed viral loci consisting of single viral segments and clusters of replication genes. By contrast, genomes of braconid wasps associated with bracoviruses have clustered viral segments and more dispersed replication genes. For instance, while proviral segments in *M. demolitor* are located in only eight loci, the 76 nudiviral replication genes are dispersed across the wasp genome except for two sets of 12 and 8 genes respectively, separated by a stretch of 30 kbp, the so-called “nudiviral cluster” [23]. These alternative genomic architectures likely reflect different regulatory mechanisms governing viral replication and particle production in the two PDV taxa.

In both PDV taxa, viral loci are amplified in the calyx cells starting at early pupal stages. However, whereas ichnoviral loci are probably all amplified [16], only the proviral segments and the nudiviral cluster are amplified in braconids [30]. In braconids, viral segments are organized in replication units delimitated by palindromic AT-rich regions (amplification junction sites), an organization that allows simultaneous co-amplification of several segments [30, 32]. Amplification junction sites were detected only for the segment DNA, but not at vicinity of the nudivirus-like cluster [30], which suggests that mechanisms governing viral DNA amplification may differ between the two types of sequences in braconids. In contrast, knowledge on the mechanisms governing ichnovirus loci amplification is still lacking. Based on their genomic organization, ichnovirus segments are most probably individually amplified, suggesting that segment viral DNA amplification relies on distinct mechanisms in the two PDV taxa. However, more studies are necessary to determine whether or not sequences longer than the proviral ichnovirus segments—presently delimited by DRJs—are amplified, and if so, to analyze the flanking sequences of these ichnoviral “replication units” in order to identify potential amplification junction sites. Similarly, further studies are needed to characterize the limits of the amplified IVSPERs to identify potential amplification sites that could be compared to those of ichnovirus segments.

Different genomic architectures may have consequences for the mechanisms regulating the expression levels of replication genes in the calyx cells. In the case of bracoviruses, DNA amplification at the nudiviral cluster results in high expression levels of the corresponding genes [56]; however, transcriptional control also relies on the nudiviral RNA polymerase that allows expression of viral genes whatever their location. In ichneumonids, increase in gene copies is one, but also probably not the only, mechanism involved in transcriptional control. Indeed, IVSPER genes differ in their expression level in *H. didymator* calyx [35], which suggests involvement of other gene-specific mechanisms. Moreover, the clustering of replication genes in ichneumonids may facilitate coordinated regulation of their expression.

### Mechanism of excision of proviral loci in ichnoviruses and bracoviruses

Direct repeat junctions (DRJs) have been identified for most of the *H. didymator* and *C. sonorensis* proviral segments. DRJs present at the extremities of the integrated form of the viral segment were first described for CsIV [25], and for the bracoviruses associated with *Chelonus inanitus* [57] and *Cotesia congregata* [26]. The presence of internal repeats, which allows the generation of multiple circular molecules from the same proviral template in a process termed “segment nesting,” has also been reported, mainly for ichnoviruses [25] and very occasionally for bracoviruses [15].

Some of the ichnovirus proviral segments identified in this work lacked terminal direct repeats (five CsIV segments of the 32 identified and two HdIV segments), which suggests they have lost their ability to be excised. For four CsIV segments, this is consistent with the failure to observe their circular form when the packaged genome was sequenced [37]. Pseudo-segments may be generated from a mutation in the DRJ or from the reintegration of circular forms, thus harboring a single copy of the initial DRJ. The first case has been documented in the braconid *Cotesia congregata* and the second in *C. sesamiae* [15, 58]. In *H. didymator* and *C. sonorensis*, we have not detected any repeated sequence at the ends of the aforementioned loci, making the reintegration of circular forms the most plausible hypothesis.

The mechanism and the proteins involved in DNA excision remain to be identified for ichnoviruses. In the case of bracoviruses, there is a first step of amplification of replication units, which contain one or several proviral segments delimited by DRJs and surrounded by wasp intervening and flanking sequences [30, 32]. DRJs contain a conserved AGCT tetramer embedded within a larger motif, which corresponds to the site of excision [15, 23, 59]. The DRJs would act later separating the different bracovirus segments in the amplified molecule, thus generating the circular molecules. Bracoviruses have conserved tyrosine recombinase family members of nudiviral origin which are likely involved in regulating this excision step [27].

For ichnoviruses, the present work highlights the variability of DRJs in terms of sequence length and level of homology, and the lack of a detectable motif as found in bracoviruses. Ichnovirus proviral segments are individually amplified and currently there is no data on the limits of the replication units, making it difficult to assess whether DRJs are directly involved in the excision of the segment, or whether there is also a two-step process involving additional sequences. Moreover, no virus-derived recombinase has been identified in ichnoviruses so far. It is therefore very unlikely that IV excision relies on a site-specific recombination mechanism as bracoviruses. The distribution of breakpoints, which spread out over the whole length of the DRJ in provirus circles, suggests that a homology-based mechanism is involved. Within the context of circularizing a segment delimited by two direct repeats, mechanisms of homologous recombination (HR) or single-strand annealing (SSA) can be envisioned, but their outcomes are undistinguishable from each other [60, 61]. HR repair is defined by a step of strand invasion catalyzed by the strand-exchange protein Rad51, where one single stranded DNA segment invades a double-stranded homologous sequence. HR has the potential of generating products with or without crossover, but only crossovers can generate circles from recombination between direct repeats. The formation of crossover is usually infrequent in somatic cells and would therefore require some specific regulation in order to produce circles with high yield. On the other hand, the mechanism of SSA seems particularly plausible, because this DNA double-strand break repair mechanism involves annealing of homologous repeat sequences and produces a deletion rearrangement between the repeats, or it can similarly generate a circle by annealing two direct repeats on a linear fragment [62]. However, even low levels of sequence divergence (< 10%) between the repeats have been shown to inhibit strongly HR and SSA [63, 64], making it difficult to explain the efficiency of the process between CsIV and HdIV DRJs, which have on average only 85% identity. One possibility is that the excision process is less sensitive to sequence divergence than in previously studied systems: possibly because it takes place on amplified fragments outside the context of the chromosome, or because specific regulations take place in calyx cells. Alternatively, it has been proposed that the repair between divergent repeats can be taken on by a composite mechanism involving early steps of SSA to align the homologous sequences of the DRJs and a bridging mechanism based on annealing between very short homology stretches (< 10 bp), the alternative end joining (ALT-EJ) [62, 64]. Newly integrated proviral segments could have initially been excised through the ALT-EJ pathway, which is not expected to favor specific junction sites. From this ancestral situation, the acquisition of direct repeats would have fixed the junctions, allowing for a decrease of non-functional circles due to uncontrolled deletions and maybe a higher efficiency.

Our data also confirms the high level of segment nesting in ichnovirus segments, which may harbor multiple DRJs that differ in sequence and number. This complexity suggests the capacity to produce, from a single template, a series of related circular molecules by intrachromosomal homologous recombination. The possibility for this system of generating a large variety of molecules is further accentuated by the finding, using an automated search, of various possible excision and recombination sites within DRJs, with some sites appearing to occur more frequently than others.

### Evolutionary implications

Two main conclusions arise from the comparison of the two genomes, providing insights into the evolutionary forces driving ichnovirus domestication in campoplegine wasps. First, the genes potentially involved in ichnovirus particle production (replication genes) are highly conserved in terms of gene content and gene order. In both *H. didymator* and *C. sonorensis*, there are only a few replication gene clusters, and one or two isolated genes. Second, in direct contrast, the viral segments carrying virulence genes are divergent between the two species, despite the existence of common gene families. The two components of the ichnoviral genome also differ in terms of conservation of their genomic localization: proviral segments were not flanked by synteny blocks shared between *H. didymator* and *C. sonorensis*, unlike the situation in braconids, where the viral segments remain in homologous positions [15]. Conversely, two of the five IVSPERs were localized in the same genomic regions in *H. didymator* and *C. sonorensis*. These two IVSPER harbor related genes, which suggests a shared ancestral origin. These loci may represent ancestral viral insertion sites conserved in both wasp genomes.

PDVs in both braconids and ichneumonids provide a solution to the same adaptive demand: inducing physiological changes in the lepidopteran hosts to allow the survival of a koinobiont endoparasitoid. The independent domestication of unrelated viruses in these two lineages represents a remarkable example of convergent evolution, but the differences in genomic architecture in each virus group suggest that different pathways were followed in these two lineages to achieve these similar solutions. Considering the common life history strategy in both groups, why would the genomic architecture of the virus need to be so different between these groups? While this remains an unresolved question, the answer may be related to the divergent nature of the virus ancestor, or to pre-existing differences in the genomic environment of ichneumonids and braconids. Previous research has indicated that closely related species tend to share more similar genetic backgrounds, enabling them to use similar pathways to achieve adaptive solutions [65]. In contrast, relatively distant relatives may require different biochemical or genetic mechanisms to show the same adaptive functions [66, 67].

The conservation of IVSPER genes is consistent with their role in the machinery that allows the wasp to produce virus particles, which presumably prevents rapid change. On the other hand, proviral segments carry virulence genes that need to respond to counter-adaptations arising in the immune system of the parasitoid’s host [68]. Since campoplegines are koiniobiont endoparasitoids that often have a restricted host range [21, 69], proviral sequences are expected to evolve rapidly and in a species-specific manner. Finally, we did identify a shared syntenic block containing a proviral segment in *H. didymator* and an IVSPER in *C. sonorensis*. In a scenario assuming a common origin for IVSPER and viral segments (i.e., the ancestral virus), this locus may represent an ancestral viral insertion site, which may have contained the complete ancestral virus genome before its separation in two components, the proviral segments, and the replication gene clusters.

## Conclusions

**Fig. 5:**
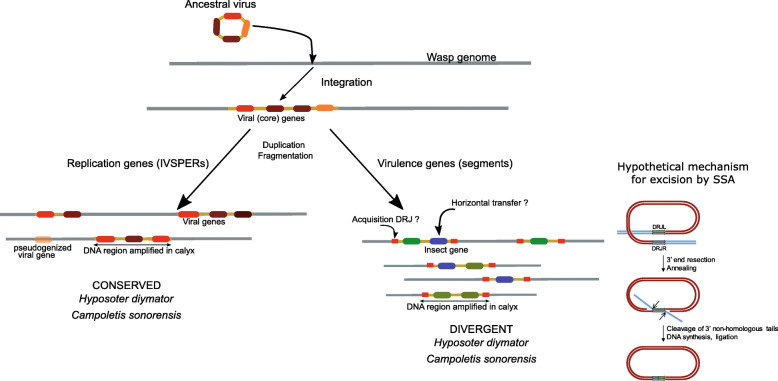
Steps of virus domestication in ichneumonids. Following integration of an ancestral virus genome in a wasp chromosome, the viral sequences were maintained but underwent significant modifications over evolutionary time. The viral sequences including genes necessary to produce particles (IVSPERs) were conserved through evolution, although they have undergone fragmentation and gene duplications and have lost some genes, including the viral DNA polymerase. The encapsidated sequences (proviral segments) include virulence genes involved in promoting parasitism. Ichnovirus segments likely derive from ancestral viral sequences that have acquired virulence genes from the wasp. Both proviral segments and IVSPERs are amplified in the replicative tissue in a coordinated manner suggesting regulation by a common mechanism and that they may both derive from the ancestral virus. Following amplification, viral segments are excised via homologous recombination or single-strand annealing mechanism (depicted) involving the direct repeated junctions

We report the whole genome sequencing of two parasitoid wasps, *H. didymator* and *C. sonorensis*, which both harbor integrated ichnoviruses. These annotated full genomes, the first for the family Ichneumonidae, provide a comprehensive picture of the architecture of ichnoviruses in these wasp genomes (Fig. 5). Our results reveal a clear duality between the proviral segments and the conserved viral machinery, differences that may be linked to the biological functions of these elements. Proviral segments, which are delivered from the parasitoid to its host and harbor virulence genes needed for successful parasitization, are isolated and scattered across the wasp genomes, in locations that differ between the two wasp species. By contrast, the replication genes required to produce the delivery system (the virus particle) are clustered in the wasp genome, and the gene content and gene order in the clusters (IVSPERs) are highly conserved between the two wasps. While conservation of the viral machinery versus diversification of the viral segments is also observed in bracoviruses, ichnovirus genomic organization of each component is in marked contrast to that observed in bracoviruses. This leads to the hypothesis, yet to be validated, that solutions to adaptive demands can arise convergently via different evolutionary pathways. Understanding the origins of the genomic architecture of modern ichnoviruses from the domestication of an ancestral virus will require the identification of the group of viruses to which the ancestrally integrated virus belongs and the sequencing of other ichneumonid wasps from multiple lineages.

## Methods

### Target species and insect rearing

Two species from the monophyletic subfamily Campopleginae [53, 54] were chosen as target taxa for whole genome sequencing: *Hyposoter didymator* occurs in all Western Europe and mainly parasitizes *Helicoverpa armigera* [70] whereas *Campoletis sonorensis* occurs from North to South America and parasitizes several noctuid species [71].

Specimens of *H. didymator* were reared on *Spodoptera frugiperda* larvae as previously described [36]. Male specimens of *C. sonorensis* wasps were furnished from a colony maintained at the University of Kentucky and reared as described in [72].

### *C. sonorensis* whole genome sequencing, assembly, and automatic annotation

Genomic DNA was extracted from one single adult male using the Qiagen™ MagAttract HMW DNA kit, following the manufacturer’s guidelines. The resulting extraction was quantified using a Qubit™ dsDNA High Sensitivity assay, and DNA fragment size was assessed using an Agilent™ Genomic DNA ScreenTape. Sample libraries were prepared using 10X Genomics Chromium technology (10X Genomics, Pleasanton, CA), followed by paired-end (150 base pairs) sequencing using one lane on an Illumina HiSeqX sequencer at the New York Genome Center (Additional file 14: Table S14).

Assembly of the sequenced reads was conducted using Supernova v.2.1.1 [38]. Reads were mapped back to the assembled genome using Long Ranger (https://support.10xgenomics.com/genome-exome/software/pipelines/latest/what-is-long-ranger) and error correction was performed by running Pilon [73]. Note that Supernova recommends 56X total coverage and sequencing deeper than 56X reduces the assembly quality. A full lane of HiSeqX produced several times the sequencing data we needed to reconstruct the genome. Hence, raw reads were divided in four subsets and four separate assemblies were conducted in parallel, with the best one chosen for downstream analyses.

We ran Kraken 2 [74] on the assembly to check for bacterial contamination. All contigs that were classified as bacteria were removed before proceeding with other analyses. We used RepeatMasker [75] to identify repeat regions using the honeybee, *Apis mellifera*, as the model species. *H. didymator* transcripts were aligned to the *C. sonorensis* genome using BLAT [76]. We created hints files for Augustus from the repeat-masked genome and the BLAT alignments. We also ran Benchmarking Universal Single-Copy Orthologs (BUSCO) version 3.0.2 [48] with the long option both to assess genome completeness and to generate a training set for Augustus. We then ran Augustus v3.3 [40] for gene prediction using the three lines of evidence, the RepeatMasker-generated hints, the BLAT-generated hints, and the BUSCO-generated training set.

### *H. didymator* whole genome sequencing, assembly, and automatic annotation

Genomic DNA was extracted from a batch of adult males (*n* = 30). DNA extractions that passed sample quality tests were then used to construct 3 paired-end (inserts lengths = 250, 500, and 800 bp) and 2 mate pairs (insert length = 2000 and 5000 bp) libraries, and qualified libraries were used for sequencing using Illumina Hiseq 2500 technology (Additional file 14: Table S15) at the BGI. For genome assembly, the raw data was filtered to obtain high-quality reads.

The reads were assembled with Platanus assembler v1.2.1 [39], in 2 steps (contigs assembly and scaffolding), then the scaffold gaps were filled with SOAPdenovo GapCloser 1.12 [77]. Finally, only scaffolds longer than 1000 bp were kept for further analyzes. Assemblathon2 [42] was used to calculate metrics of genome assemblies.

For annotation, EST reads from venom [78], as well as reads obtained using GS FLX (Roche/454), Titanium chemistry from total insects [78] and from ovaries [79], were mapped to the genome with GMAP [80], and Illumina reads published previously [35], or from the 1KITE consortium (http://1kite.org/subprojects.html) using STAR [81], and new calyx RNA-seq (SRA accession: PRJNA590863) with TopHat2 v2.1.0 [82]. BRAKER1 v1.10 [41] was used to predict genes in the genome of *H. didymator* using default settings. Gene annotation was evaluated using BUSCO version 3.0.2 [48] with a reference set of 1658 proteins (conserved in Insecta).

The other parasitoid genomes used in this work for comparison purposes were similarly analyzed (genomes available at NCBI for *Microplitis demolitor* [31] (PRJNA251518), *Fopius arisanus* [43] (PRJNA258104), *Diachasma alloeum* [44] (PRJNA306876), and *Nasonia vitripennis* [45] (PRJNA13660); *Venturia canescens* genome [22] available at https://bipaa.genouest.org/sp/venturia_canescens/).

### Manual annotation of the viral loci

Manual annotation of viral regions was performed using the genome annotation editor Apollo browser [83] available on the BIPAA platform (https://bipaa.genouest.org). The encapsidated forms of the HdIV and CsIV genomes were previously sequenced with Sanger technology by isolating DNA from virions [36, 37]. *H. didymator* IVSPER were also previously sequenced [16]. To identify the viral loci, sequences available at NCBI for campoplegine IV segments and IVSPER sequences from campoplegine and banchine species were used to search the *H. didymator* and *C. sonorensis* genome scaffolds using the Blastn tool implemented in the Apollo interface. To determine the limits of the proviral segments, we searched for direct repeats at the ends of the viral loci by aligning the two sequences located at each end using the Blastn suite at NCBI. The start or stop codons of the genes located at the ends of the IVSPER loci were considered as the borders of the IVSPER.

### Transposable element detection

Transposable elements (TEs) were identified in *H. didymator* and *C. sonorensis* genomes using the REPET pipeline [84, 85]. The enrichment analysis was performed using LOLA [86] comparing the observed number of each TE family member in a region encompassing IV segments and 10 kbp around, and 1000 random segments of the same length extracted with bedtools shuffle [87].

### Orthologous genes and syntenic regions

To identify homology relationships between sequences of *H. didymator*, *C. sonorensis*, and other parasitoids with available genomes (one ichneumonid *Venturia canescens*, three braconids *Microplitis demolitor*, *Fopius arisanus*, and *Diachasma alloeum*, and one pteromalid *Nasonia vitripennis*), as well as two taxonomically more distant insect sequences (the bee *Apis mellifera* and the fly *Drosophila melanogaster*), a clustering was performed using the orthogroup inference algorithm OrthoFinder version 2.2.7 [88]. Sequences predicted by automatic annotation (Braker or Augustus) but also some resulting from manual annotations were used. Thus, a total of 18,154 protein coding genes for *H. didymator* and 21,987 for *C. sonorensis* were included in the analysis (Table 4A). The syntenic blocks were reconstructed with Synchro [89] using the genomes and proteomes of the same species.

### *H. didymator* genomic BAC library construction and sequencing

Genomic BAC clones were obtained as described in [16]. Briefly, high molecular weight DNA was extracted from *H. didymator* larval nuclei embedded in agarose plugs. The nuclei were lysed and the proteins degraded by proteinase K treatment. DNA was partially digested with *Hin*dIII. The size of the fragments obtained averaged 40 kbp as controlled by Pulse Field Gel Electrophoresis. Fragments were ligated into the pBeloBAC11 vector. High-density filters were spotted (18,432 clones spotted twice on nylon membranes) and screened using specific 35-mer oligonucleotides. Positive clones were analyzed by fingerprint, and for each probe, one genomic clone was selected and sequenced using Sanger technology (shotgun method) by the Génoscope, Evry, France. The sequences obtained were then submitted to a Blastn similarity search against NCBI nr database in order to confirm presence of HdIV sequences. Four BAC clones containing HdIV sequences were used as probes in FISH experiments (see below).

### Fluorescent in situ hybridization (FISH) on *H. didymator* chromosomes

The *H. didymator* genome is composed of 12 chromosomes [90]. Karyotypes were prepared from male reproductive tracts from pupae and young adults. The testes were dissected in saline solution and placed in colchicine/colcemid solution (50μg/ml) for 10 min. After elimination of the liquid, a hypotonic solution (Na citrate 0.5%) was added for 10 min. The solution was then replaced by fixative (1 vol. acetic acid/3 vol. methanol) and let to incubate for 40 min. The genitalia were then placed on a glass slide, a drop of acetic acid 60% was added to further shred the tissue, and the slide was placed on a hot plate at 42 °C until complete evaporation of the liquid. The samples were stained with DAPI and observed under a fluorescent microscope in order to select slides with sufficient and suitable caryotypes. Four genomic clones containing a viral sequence (Hd11 (cloneCE-15P20), Hd6 (clone CF-16G11), Hd29 (clone AB-06P08), and Hd30 (clone BR-08O01)) were used as probes. They were alternatively labeled using the Dig RNA labeling mix (Roche) or the biotin RNA labeling mix (Roche). For hybridization, the samples were rehydrated and denatured during 6 min by a 0.07 N NaOH treatment. The anti-digoxigenin antibody was labeled with rhodamine (Roche) (dilution 1/50) and the anti-biotin antibody with FITC (Vector laboratories) (dilution 1/200) overnight at 37 °C. Images were captured on a Zeiss AxioImager Apotome microscope.

### Re-sequencing of HdIV packaged genome

The viral DNA was extracted following the procedure described in [91]. Briefly, ovaries from about 100 female wasps were dissected in PBS and placed in a 1.5-ml microfuge tube. The final volume was adjusted to 500 μl using Tris-EDTA buffer and the ovaries were homogenized by several passages through a 23-gauge needle. The resulting suspension was passed through a 0.45-um pore-size cellulose acetate filter to recover the HdIV viral particles. For viral DNA extraction, the filtrate was submitted to proteinase K and Sarcosyl treatment overnight at 37 °C, then to RNase A treatment 2 h at 37 °C. DNA was further extracted with phenol-chloroform-isoamyl alcohol and precipitated with ethanol. The DNA pellet was re-suspended in ultra-pure water and was sequenced using GS FLX (Roche/454), Titanium chemistry (Eurofins Genomics). The obtained reads [92] were used for DRJ excision site analyses (see below).

### DRJs and breakpoint analysis in *H. didymator*

The proviral integrated segments are circularized and excised by homologous recombination between its extreme DRJs (at left and right extremities of the given segment, named DRJL and DRJR respectively). When the two copies of the DRJ exhibit some punctual differences, the excision site or breakpoint can be identified in a given recombined DRJ sequence with more or less resolution depending on the level of divergence between the DRJ copies.

In order to identify and analyze DRJ excision sites of a set of circularized IV sequences in an automatic fashion, we developed the following method called DrjBreakpointFinder and freely distributed at http:// github.com/stephanierobin/DrjBreakpointFinder/. The method takes as input a set of circularized sequences (usually obtained by sequencing) and a reference genome. It is composed of two main steps. The first step consists in identifying triplets of sequences (read-DRJL-DRJR) representing the recombined DRJ and its two parental DRJs, by mapping the sequencing reads to the reference genome. In the second step, a precise multiple alignment is computed for each sequence triplet, and a segmentation algorithm, inspired from the breakpoint refinement method Cassis [93], is applied along the recombined DRJ sequence to identify in the best case scenario the excision site or more generally the breakpoint region. To do so, the segmentation algorithm estimates the best partition of the recombined DRJ sequence into three distinct segments, corresponding to homology with DRJR, the breakpoint region, and homology with DRJL respectively, given the repartition of punctual differences with the two parental DRJs. The segmentation algorithm is classically based on fitting a piecewise constant function with two changepoints to the punctual difference signal (see [94]). DrjBreakpointFinder further gathers breakpoint results by proviral segments or DRJ pairs, in order to obtain for each the distribution of potential excision sites observed in a given circular virus sequencing dataset. The output of DrjBreakpointFinder consists of breakpoint region coordinate files along with visual representations for each proviral segment or DRJ pair.

In this paper, DrjBreakpointFinder was applied to two circular viral DNA sequencing datasets. Circular DNA was extracted from HdIV particles and sequenced by 454 and Sanger technologies, resulting in 40,343 and 15,575 reads, respectively [92].

In addition, the DRJ copy was manually analyzed for a subset of 8 segments (Hd12, Hd16, Hd19, Hd22, Hd24, Hd28, Hd29, and Hd30) that presented only one right and left DRJs in their integrated form. Junctions were amplified by PCR using primers located within the viral sequence, downstream and upstream the DRJs. PCR products were cloned in pGEM and 3 to 5 plasmid clones were then sequenced using Sanger technology for each segment. The obtained recombined junction sequences were then aligned with the 2 parental DRJs in an attempt to localize the excision site, based on the nucleotides differing between the 2 DRJs (see Additional file 11).

## Supplementary information

**Additional file 1: **Table S1. Transposable elements (TE) in the genomes of *Hyposoter didymator* and *Campoletis sonorensis*. Total number and percentage are given for each TE class. Detection performed using the REPET pipeline (see Methods). LINE, long interspersed nuclear element; LTR, long terminal repeat; SINE, short interspersed nuclear element.

**Additional file 2:.** Orthogroups analyses. Table S2. Orthofinder clustering metrics. G50: cluster size at which 50% of genes are in an orthogroup (OG) of that size or greater. O50: fewest number of orthogroups required to reach G50; G50 (assigned genes) = 16; G50 (all genes) = 14; O50 (assigned genes) = 3063; O50 (all genes) = 4112. Species carrying a PDV are indicated with an asterisk. Species carrying polydnaviruses are indicated by asterisks. Table S3. Number of orthogroups shared by each species-pair (i.e. the number of orthogroups which contain at least one gene from each of the species-pairs). Species carrying a PDV are indicated with an asterisk. Table S4. Number of species-specific orthogroups. Number of orthogroups specific to one or groups of species.

**Additional file 3:.** Table S5. Synteny blocks between pairwise comparisons of multiple parasitoid genomes. Synteny blocks were computed using SynChro [89], a tool based on a simple algorithm that computes Reciprocal Best-Hits (RBH) to reconstruct the backbones of the synteny blocks. Species carrying polydnaviruses are indicated by asterisks. [Ichn.]: Ichneumonid; [Braco.]: Braconid.

**Additional file 4: **Table S6. List of scaffolds in *Hyposoter didymator* and *Campoletis sonorensis* genomes containing at least on ichnovirus sequence. Are indicated the scaffold name and length, the name of the proviral segment or of the Ichnovirus structural protein encoding region (IVSPER) found in the scaffold, its length and position in the scaffold, the name of the direct repeats flanking the segment or within the segment, and the name of the genes predicted in each viral locus. DRJ, direct repeat junction; R, right; L, left; int, internal.

**Additional file 5: **Table S7. List of ichnoviral genes identified in *Hyposoter didymator* and *Campoletis sonorensis* genome scaffolds containing at least on ichnovirus sequence. Are indicated the scaffold name, the name of the proviral segment or of the Ichnovirus structural protein encoding region (IVSPER) found in the scaffold, its length and position in the scaffold, the name of the gene, its position in the scaffold, if it contains or not introns, the size of the predicted protein, then the NCBI blast P search results (NCBI accession number and ID of the best match, the blast P e-value and the percentage of identities). Last column indicates comments, or notes reporting discrepancies in the genomic sequence compared with the original CDS sequence in NCBI database.

**Additional file 6: **Figure S1. *H. didymator* proviral loci corresponding to two segments previously described as “distinct” but sharing part of their sequence [36]. Segments Hd2a (GenBank: KJ586332.1) and Hd2b (GenBank: KJ586327.1) co-localize in the same genome locus here named Hd2; segments Hd11a (KJ586322.1) and Hd11b (KJ586302.1) co-localize in the same genome locus here named Hd11; Hd17a (KJ586314.1) and Hd17b (KJ586316.1) co-localize in the same genome locus here named Hd17; Hd20a (KJ586312.1) and Hd20b (KJ586297.1) co-localize in the same genome locus here named Hd20; Hd26a (KJ586301.1) and Hd26b (KJ586306.1) co-localize in the same genome locus here named Hd26; and finally, Hd31 (KJ586299.1) and Hd34 (KJ586295.1) co-localize in the same genome locus here named Hd31-34. Each proviral locus was characterized by the presence of two different direct repeated sequences (DRJ1 and DRJ2) at the extremities of each of the overlapping segments. Scale bar: 1000 nt.

**Additional file 7: **Dispersion of the viral loci within ichneumonid genomes. Table S8. Distance (in bp) between two segments, a segment and an IVSPER or between two IVSPERs localized in the same scaffold. Figure S2. Graphical representation of the mean distance (in Kbp) between viral loci in *H. didymator* and *C. sonorensis* genomes. Data are given between 2 segments, between a segment and an IVSPER, and/or between 2 IVSPERs.

**Additional file 8: **Table S9. Transposable elements (TE) found in *Hyposter didymator* segments, IVSPERs and neighboring regions. The LOLA package [86] was used to assess if some particular TE were enriched close to viral circles or IVSPER. Genomics positions were enlarged to 10 kbp at each segments ends and sampled against 1000 other similar regions from the genome, then used it a random reference. LOLA identifies overlaps and calculates enrichment for each TE. For each pairwise comparison, a series of columns describe the results of the statistical test (pvalueLog: -log10(pvalue) from the fisher’s exact result; oddsRatio: result from the fisher’s exact test; q-value transformation to provide false discovery rate (FDR) scores automatically). Some TE are enriched around viral locations, but after FDR correction, nothing was significant.

**Additional file 9: **Table S10. List of direct repeat junctions (DRJ) found at the ends or within proviral segments genes identified in *Hyposoter didymator* and *Campoletis sonorensis* genome scaffolds. Are indicated the scaffold name, the name of the proviral segment, its length and position in the scaffold, the name of the DRJ, its size and position in the scaffold and the DRJ sequence. Nucleotide identities are indicated for each pair of DRJ.

**Additional file 10:.** DRJs analysis. Figure S3. Examples of the different types of DRJ position. a. Proviral segment with two copies of a single direct repeat (DRJ1L and DRJ1R), one at each end of the segment. b. Proviral segment with two distinct repeated sequences (DRJ1, in yellow and DRJ2, in green), each present in two copies (DRJ1L and DRJ1R, DRJ2L and DRJ2R). c. Proviral segment with two repeated sequences, each present in two or more copies. DRJ1s in yellow, DRJ2s in green, HdIV genes represented by arrows. Table S11. DNA motifs found in the direct repeated sequences flanking the IV segments inserted in wasp genomes. Analysis was performed using the DNAMINDA2 webserver (http://bmbl.sdstate.edu/DMINDA2/annotate.php); the input dataset was composed of 99 DRJ sequences (right junctions of HdIV and CsIV segments). A total of 89 motifs were obtained; only those whose occurrence exceed 70% of the DRJs are reported. Table S12. Result of genome search using motifs predicted with DMINDA 2.0 webserver. Occurrence rate of motifs predicted with DMINDA 2.0 webserver in DRJs and whole genome sequences. Each of the two motifs was search among the 6 bp kmers present in the whole genome (201,969,604) and in the DRJs (33,930). The significance was evaluated using a Chi2 (taking into account the ratio of these motifs / all the other motifs in the DRJS and in the genome).

**Additional file 11 **Manual analysis of the DRJ regions containing an excision site. Alignments of Sanger-sequenced DRJ regions from integrated and circular forms of seven *H. didymator* IV segments containing a putative excision site.

**Additional file 12: **Comparative analysis of *Campoletis sonorensis* and *Hyposoter didymator* viral segments. Figure S4. Proviral segment size and gene content, i.e. the number of genes of each multigenic family found per segment. a. *C. sonorensis* ichnovirus (CsIV). b. *H. didymator* ichnovirus (HdIV). Ichnovirus genes: rep, repeat element genes; repM, repeat element genes with multiple repeated elements; vinx, viral innexin; vank, viral ankyrin; cys, cys-motif rich protein; PRRP, polar residue rich protein; N, N gene; Gly-Pro, glycine-proline rich protein. Figure S5. Synteny of *H. didymator* genomic regions where viral segments are inserted compared with *C. sonorensis* and other parasitoid genomes. a. Example of a syntenic region where only the *H. didymator* genome presents a viral segment insertion. *H. didymator* genes from HD005010 to HD005030. b. The unique case found of a syntenic region where a viral segment in *H. didymator* and an IVSPER in *C. sonorensis* are inserted in the same position. *H. didymator* genes from HD010552 to HD010574. c. The unique case found of a syntenic region where a viral segment is inserted in both *H. didymator* and *C. sonorensis* genomes, but in two different positions. *H. didymator* genes from HD010503 to HD010526. Hd: *Hyposoter didymator*; Cs: *Campoletis sonorensis*; Vc: *Venturia canescens* (ichneumonid that has lost the ichnovirus ([22]); Md: *Microplitis demolitor* (braconid with a bracovirus); Fa: *Fopius arisanus* (braconid with virus-like particles). Numbers following the species name correspond to scaffold number for Hd, Cs and Vc, NCBI project codes for Md and Fa). Triangles within genomic regions correspond to predicted genes; triangles of the same color correspond to orthologs; white triangles are singletons or orphan genes. For better visualization, the name of the gene is indicated only for some viral (in red for segments, in blue for IVSPERs) genes. See Additional file 13: Table S13, for *H. didymator* genes list.

**Additional file 13: **Table S13. List of the *Hyposoter didymator* genes present in the syntenic blocks represented in Additional file 12: Fig. S5 and in Fig. 4. Are indicated the *H. didymator* scaffold name, gene ID, its position in the scaffold, the number of the orthogroup to which it belongs and the result of the best match obtained following Blast similarity search. For each *H. didymator* gene, the corresponding *Campoletis sonorensis* gene ID, orthogroup number and position in *C. sonorensis* scaffold are indicated.

**Additional file 14: **Characteristics of the libraries used for genome assembly. Table S14. *Campoletis sonorensis*. Table S15. *Hyposoter didymator*.

## Data Availability

The datasets supporting the conclusions in this article are available at the NCBI under the Bioproject accession numbers PRJNA589497 for *Hyposoter didymator* [95] and PRJNA590982 for *Campoletis sonorensis* [96]. The DrjBreakpointFinder method developed in this study is freely distributed at http:// github.com/stephanierobin/DrjBreakpointFinder. All other data are included within this article and its additional files.
