# Supplementary material for: Genomic architecture of endogenous ichnoviruses reveals distinct evolutionary pathways leading to virus domestication in parasitic wasps
Source: BMC Biol. 2020 Jul 24;18:89. doi: 10.1186/s12915-020-00822-3 (PMC7379367; doi:10.1186/s12915-020-00822-3)
Supplement: Supplementary file 1 — Additional file 1: Table S1. Transposable elements (TE) in the genomes of Hyposoter didymator and Campoletis sonorensis. Total number and percentage are given for each TE class. Detection performed using the REPET pipeline (see Methods). LINE, long interspersed nuclear element; LTR, long terminal repeat; SINE, short interspersed nuclear element. [file 12915_2020_822_MOESM1_ESM.pdf]

Additional file 1. Table S1. Transposable elements (TE) in the genomes of *Hyposoter didymator* and *Campoletis sonorensis*. Total number and percentage are given for each TE class. Detection performed using the REPET pipeline (see Methods). LINE, long interspersed nuclear element; LTR, long terminal repeat; SINE, short interspersed nuclear element.

	Hyposoter didymator		*Campoletis sonorensis*	
Transposable elements (TE) classes	Total number	Percentage of TE classes	Total number	Percentage of TE classes
LINE	98	10%	189	20%
LTR retrotransposons	106	11%	193	20%
SINE retrotransposons	33	3%	56	6%
DNA transposons	299	30%	314	33%
Others	464	46%	199	21%
Total	1000	/	951	/
