# Supplementary material for: Genomic architecture of endogenous ichnoviruses reveals distinct evolutionary pathways leading to virus domestication in parasitic wasps
Source: BMC Biol. 2020 Jul 24;18:89. doi: 10.1186/s12915-020-00822-3 (PMC7379367; doi:10.1186/s12915-020-00822-3)
Supplement: Supplementary file 2 — Additional file 2:. Orthogroups analyses. Table S2. Orthofinder clustering metrics. G50: cluster size at which 50% of genes are in an orthogroup (OG) of that size or greater. O50: fewest number of orthogroups required to reach G50; G50 (assigned genes) = 16; G50 (all genes) = 14; O50 (assigned genes) = 3063; O50 (all genes) = 4112. Species carrying a PDV are indicated with an asterisk. Species carrying polydnaviruses are indicated by asterisks. Table S3. Number of orthogroups shared by each species-pair (i.e. the number of orthogroups which contain at least one gene from each of the species-pairs). Species carrying a PDV are indicated with an asterisk. Table S4. Number of species-specific orthogroups. Number of orthogroups specific to one or groups of species. [file 12915_2020_822_MOESM2_ESM.pdf]

Additional file 2. Orthogroups analyses

Table S2. Orthofinder clustering metrics. G50: cluster size at which 50% of genes are in an orthogroup (OG) of that size or greater. O50: fewest number of orthogroups required to reach G50: G50 (assigned genes) = 16; G50 (all genes) = 14; O50 (assigned genes) = 3,063; O50 (all genes) = 4,112. Species carrying polydnaviruses are indicated by asterisks.

		Hymenoptera: Ichneumonidae			*Hymenoptera: Braconidae*			*Hymenoptera: Pteromalidae*	*Hymenoptera: Apidae*	*Diptera: Drosophilidae*
	All	*Hyposoter didymator**	*Campoletis sonorensis**	*Venturia canescens*	*Microplitis demolitor**	*Fopius arisanus*	*Diachasma alloeum*	*Nasonia vitripennis*	*Apis mellifera*	*Drosophila melanogaster*
Number of genes	199,523	18,154	21,987	23,401	18,586	18,906	19,692	24,846	23,471	30,480
Number of genes in OGs	168,268	15,808	15,691	17,511	16,843	18,392	18,270	21,417	22,483	21,853
% of genes in OGs	84.3	87.1	71.4	74.8	90.6	97.3	92.8	86.2	95.8	71.7
Number of unassigned genes	31,255	2,346	6,296	5,890	1,743	514	1,422	3,429	988	8,627
Total number of OGs	12,825	10,880	10,293	11,041	9,121	9,558	9,616	9,094	8,976	7,932
Number species-specific OGs	501	11	32	26	29	8	17	77	36	265
Number genes in species-specific OGs	2,773	69	288	216	163	36	76	406	141	1,378
% genes in species-specific OGs	1.4	0.4	1.3	0.9	0.9	0.2	0.4	1.6	0.6	4.5
Number of single-copy OGs	454									

Table S3. Number of orthogroups shared by each species-pair (i.e. the number of orthogroups which contain at least one gene from each of the species-pairs). Species carrying polydnaviruses are indicated by asterisks.

	*Hyposoter didymator**	*Campoletis sonorensis**	*Venturia canescens*	*Microplitis demolitor**	*Fopius arisanus*	*Diachasma alloeum*	*Nasonia vitripennis*	*Apis mellifera*	*Drosophila melanogaster*
*Hyposoter didymator**	10,869	9,696	10,362	8,664	8,830	8,838	8,508	8,586	7,148
*Campoletis sonorensis**		10,261	9,789	8,086	8,258	8,257	7,955	8,031	6,710
Venturia canescens			11,015	8,665	8,800	8,806	8,492	8,545	7,123
*Microplitis demolitor**				9,092	8,592	8,597	8,159	8,207	6,991
Fopius arisanus					9,550	9,256	8,284	8,371	7,062
Diachasma alloeum						9,599	8,312	8,357	7,068
Nasonia vitripennis							9,017	8,134	6,912
Apis mellifera								8,940	7,022
Drosophila melanogaster									7,667

Table S4. Number of species-specific orthogroups. Number of orthogroups specific to one or groups of species.

**Species or groups**	**Number species-specific orthogroups**
Hyposoter didymator	11
Campoletis sonorensis	32
Venturia canescens	26
Ichnovirus-carrying Ichneumonids (*H. didymator*, *C. sonorensis*)	313
Ichneumonids (*H. didymator*, *C. sonorensis*, *V. canescens*)	1,728
Ichneumonids & Braconids (*H. didymator*, *C. sonorensis*, *V. canescens*, *M. demolitor*, *F. arisanus*, *D. alloeum*)	2,610
Parasitic wasps (*H. didymator*, *C. sonorensis*, *V. canescens*, *M. demolitor*, *F. arisanus*, *D. alloeum*, *N. vitripennis*)	3,240
Hymenoptera	5,158
Hymenoptera + diptera	12,825
