# Supplementary material for: Genomic architecture of endogenous ichnoviruses reveals distinct evolutionary pathways leading to virus domestication in parasitic wasps
Source: BMC Biol. 2020 Jul 24;18:89. doi: 10.1186/s12915-020-00822-3 (PMC7379367; doi:10.1186/s12915-020-00822-3)
Supplement: Supplementary file 3 — Additional file 3:. Table S5. Synteny blocks between pairwise comparisons of multiple parasitoid genomes. Synteny blocks were computed using SynChro [89], a tool based on a simple algorithm that computes Reciprocal Best-Hits (RBH) to reconstruct the backbones of the synteny blocks. Species carrying polydnaviruses are indicated by asterisks. [Ichn.]: Ichneumonid; [Braco.]: Braconid. [file 12915_2020_822_MOESM3_ESM.pdf]

Additional file 3. Table S5. Synteny blocks between pairwise comparisons of multiple parasitoid genomes. Synteny blocks were computed using SynChro [76], a tool based on a simple algorithm that computes Reciprocal Best-Hits (RBH) to reconstruct the backbones of the synteny blocks. Species carrying polydnaviruses are indicated by asterisks. [Ichn.]: Ichneumonid; [Braco.]: Braconid.

species 1	species 2	# genes in Species 1	# genes in Species 2	RBH	mean % similarity of RBH genes	# synteny blocks (SB)	mean number of genes in SB	Species 1 nucleotides In SB	Species 2 nucleotides in SB	# Species 1 genes in SB	# Species 2 genes in SB
***Campoletis sonorensis* [Ichn.]**	***Hyposoter didymator* [Ichn.]**	**21,987**	**18,154**	**9,201**	**82.2258**	**1,112**	**11.2334**	**130,986,569**	**136,689,043**	**11,816**	**12,828**
Campoletis sonorensis [Ichn.]*	*Diachasma alloeum [Braco.]*	21,987	19,692	7,054	72.4531	1,581	6.1151	79,457,000	121,585,809	8,101	11,023
Campoletis sonorensis [Ichn.]*	*Fopius arisanus [Braco.]*	21,987	18,906	6,963	71.7424	1,583	6.2612	78,144,432	64,120,970	7,957	11,583
Campoletis sonorensis [Ichn.]*	*Microplitis demolitor* [Braco.]*	21,987	18,586	6,889	71.3963	1,503	6.4112	79,951,790	87,227,954	8,066	10,937
Campoletis sonorensis [Ichn.]*	*Venturia canescens [Ichn.]*	21,987	23,401	9,602	81.9265	1,501	7.3997	84,998,388	87,756,926	10,31	11,589
Diachasma alloeum [Braco.]	*Fopius arisanus [Braco.]*	19,692	18,906	10,85	84.4038	1,244	13.2826	188,094,018	98,011,380	15,707	17,083
Diachasma alloeum [Braco.]	*Hyposoter didymator* [Ichn.]*	19,692	18,154	8,784	71.9646	1,949	5.8489	147,155,684	85,519,660	12,100	10,426
Diachasma alloeum [Braco.]	*Microplitis demolitor**	19,692	18,586	9,227	75.5011	1,723	8.1993	156,017,415	115,880,628	13,263	13,987
Diachasma alloeum [Braco.]	*Venturia canescens [Ichn.]*	19,692	23,401	8,435	71.8280	1,960	4.8980	101,537,848	57,440,014	10,261	8,663
Fopius arisanus [Braco.]	*Hyposoter didymator* [Ichn.]*	18,906	18,154	8,622	71.5998	2,004	5.9214	78,694,026	88,834,572	12,881	10,551
Fopius arisanus [Braco.]	*Microplitis demolitor* [Braco.]*	18,906	18,586	9,151	74.8577	1,798	7.8715	82,643,504	117,622,728	14,057	13,906

Fopius arisanus [Braco.]	*Venturia canescens [Ichn.]*	18,906	23,401	8,203	71.5408	1,957	5.0424	53,941,273	57,217,953	10,812	8,631
Hyposoter didymator [Ichn.]*	*Microplitis demolitor* [Braco.]*	18,154	18,586	8,513	72.4243	1,844	6.2796	82,632,725	106,502,067	10,063	12,767
Hyposoter didymator [Ichn.]*	*Venturia canescens [Ichn.]*	18,154	23,401	11,21	82.4090	1,695	7.2375	96,760,707	94,022,094	11,769	12,407
Microplitis demolitor [Braco.]*	*Venturia canescens [Ichn.]*	18,586	23,401	8,163	70.9083	1,848	5.1366	72,847,449	56,076,459	10,217	8,494
