# Supplementary material for: Genomic architecture of endogenous ichnoviruses reveals distinct evolutionary pathways leading to virus domestication in parasitic wasps
Source: BMC Biol. 2020 Jul 24;18:89. doi: 10.1186/s12915-020-00822-3 (PMC7379367; doi:10.1186/s12915-020-00822-3)
Supplement: Supplementary file 4 — Additional file 4: Table S6. List of scaffolds in Hyposoter didymator and Campoletis sonorensis genomes containing at least on ichnovirus sequence. Are indicated the scaffold name and length, the name of the proviral segment or of the Ichnovirus structural protein encoding region (IVSPER) found in the scaffold, its length and position in the scaffold, the name of the direct repeats flanking the segment or within the segment, and the name of the genes predicted in each viral locus. DRJ, direct repeat junction; R, right; L, left; int, internal. [file 12915_2020_822_MOESM4_ESM.pdf]

Scaffold	Scaffold length (nt)	Name of proviral segment /IVSPER	Proviral/IVSPER sequence length (nt)	Position in scaffold	Direct repeats	Genes present in viral sequence
Hyposoter didymator						
scaffold49647	1586	similar to Hd2	1537	1-1537 (partial)	not applicable	Gly-Pro_P40-like, partial
scaffold28498	4159	Hd45.1	NA	1-4214 (partial)	no repeat found at extremities	U1_Hd45.1, U2_Hd45.1
scaffold82201	6109	Hd51	4632	1077-5708	Hd51_DRJ1R/L, Hd51_DRJ2R/L	Rep1_Hd51
scaffold29771	25145	IVSP_U37	1839	16848-18686	not applicable	single CDS: U37
scaffold29771	25145	Hd46	4109	19344-23452	Hd46_DRJ1R/L, Hd46_DRJ2R/L	Vank_Hd46
scaffold1868	202142	Hd43	4159	193639-197797	Hd43_DRJ1R/L, Hd43_DRJ2R/L	U1_Hd43, Vank1_Hd43
scaffold128213	363895	Hd23.1	4457	208205-212661	Hd23.1_DRJ1R/L	Rep1_Hd23.1
scaffold128213	363895	Hd23.2	3362	248624-251985	Hd23.2_DRJ1R/L	Rep1_Hd23.2
scaffold91	761975	Hd_IVSPER-1	14020	453857-467876	not applicable	U1, IVSP1-1, U2, U3, U4, p53-2, U5, IVSP2-1, N-1, U25
scaffold91	761975	Hd15	4987	469105-474091	Hd15_DRJ1R/L	N1_Hd15
scaffold91	761975	Hd33	3835	485063-488897	Hd33_DRJ1R/L	U1_Hd33, U2_Hd33
scaffold91	761975	Hd24	4697	535698-540394	Hd24_DRJ1R/L	Vank1_Hd24, Vinx1_Hd24
scaffold91	761975	Hd_IVSPER-2	26611	541304-567914	not applicable	N-4, U26, IVSP2-2, IVSP4-1, p12-2, U27, U14, p12-3, U13, U12, U11, U10, U9, U8, IVSP3-1, IVSP1-2, U7, U6, N-3
scaffold91	761975	Hd29	4356	572514-576869	Hd29_DRJ1R/L	N1_Hd29
scaffold128215	912714	Hd9	17892	526917-544808	Hd9_DRJ1R/int/L, Hd9_DRJ2R/L	U1.1_Hd9, U2_Hd9, U6.1_Hd9, U3.1_Hd9, U4.1_Hd9, U5.1_Hd9, U1.2_Hd9, U6.2_Hd9, U3.2_Hd9, U4.2_Hd9, U5.2_Hd9, U1.3_Hd9
scaffold65	1016704	Hd37	3708	342293-346000	Hd37_DRJ1R/L	Rep1_Hd37, U1_Hd37, U2_Hd37
scaffold65	1016704	Hd3	10014	437082-447095	Hd3_DRJ1R/int/L, Hd3_DRJ2R/int/L	Cys1_Hd3, Cys2_Hd3, Cys3_Hd3, Cys4_Hd3, Cys5_Hd3
scaffold90	1092007	Hd1	NA	1-14771 (partial)	Hd1_DRJ1R/int, Hd1_DRJ2R/int-1/int-2	U1_Hd1, U2_Hd1, U3_Hd1, U4_Hd1, U5_Hd1, U6_Hd1
scaffold264	1259582	Hd28	4614	135485-140098	Hd28_DRJ1R/L	Vinx1_Hd28, Vank1_Hd28
scaffold128246	1305886	Hd49	5265	677866-683130	Hd49_DRJ1R/L	Rep1_Hd49, Rep2_Hd49, Rep3_Hd49
scaffold429	1434187	Hd48	9673	531833-541505	Hd48_DRJ1R/L, Hd48_DRJ2R/L	Rep1_Hd48, Rep2_Hd48
scaffold144	1844462	Hd20	6864	1449468-1456331	Hd20_DRJ1R/L, Hd20_DRJ2R/L	Cys1_Hd20, Cys2_Hd20
scaffold116	1992701	Hd35	3710	473341-477050	Hd35_DRJ1R/L	Rep1_Hd35
scaffold64	2416597	Hd14	5196	36336-41531	Hd14_DRJ1R/L	Vinx1_Hd14, U1_Hd14
scaffold64	2416597	Hd32	7916	88702-96617	Hd32_DRJ1R/L, Hd32_DRJ2R/L	Vinx1_Hd32, Vinx2_Hd32, U1.1_Hd32, U1.2_Hd32
scaffold64	2416597	Hd42	3157	2320523-2323679	Hd42_DRJ1R/L	Rep1_Hd42
scaffold64	2416597	Hd21	4368	2353107-2357474	Hd21_DRJ1R/L	Rep1_Hd21, Vinx1_Hd21
scaffold377	2592399	Hd8	7356	2186417-2193772	Hd8_DRJ1R/L, Hd8_DRJ2R/L	U1_Hd8, U2_Hd8, U3_Hd8, U4_Hd8, U5_Hd8
scaffold377	2592399	Hd4	10326	2459681-2470006	Hd4_DRJ1R/int/L	Rep1_Hd4, Rep2_Hd4, Rep3_Hd4, Rep4_Hd4, Rep5_Hd4, Rep6_Hd4
scaffold161	2602850	Hd25	4174	2449400-2453573	Hd25_DRJ1R/L	PRRP1_Hd25, PRRP2_Hd25, PRRP3_Hd25
scaffold127549	2774194	Hd31-34	4119	144445-148563	Hd31-34_DRJ1R/L, Hd31-34_DRJ2R/L	U1_Hd31-34, U2_Hd31-34
scaffold59	2934223	Hd12	5902	674917-680818	Hd12_DRJ1R/L	U1_Hd12, Rep1_Hd12, U2_Hd12, Rep2_Hd12
scaffold59	2934223	Hd16	7704	690205-697908	Hd16_DRJ1R/L, Hd16_DRJ2R/L	Rep1_Hd16, Rep2_Hd16, Rep3_Hd16, U1_Hd16, U2_Hd16
scaffold59	2934223	Hd11	9190	2183512-2192701	Hd11_DRJ1R/L, Hd11_DRJ2R/L	U1_Hd11, Rep1_Hd11, Vank1_Hd11, Vank1p_Hd11, Vank2_Hd11, Vank3_Hd11, Vank4_Hd11, Vank5_Hd11
scaffold59	2934223	Hd10	6507	2500377-2506883	Hd10_DRJ1R/int/L, Hd10_DRJ2R/L	Rep1_Hd10, Rep2_Hd10, Rep3_Hd10
scaffold198	3327987	Hd19	4440	1265454-1269893	Hd19_DRJ1R/L	U1_Hd19
scaffold119	3623981	Hd22	4178	698427-702604	Hd22_DRJ1R/L	Rep1_Hd22, U1_Hd22
scaffold127348	3747147	Hd40	3494	926528-930021	Hd40_DRJ1R/L	U1_Hd40, U2_Hd40
scaffold357	4004213	Hd50	5787	2218373-2224159	Hd50_DRJ1R/L	Vinx1_Hd50, Vinx2_Hd50
scaffold128243	5513913	Hd44.2	4831	4197203-4202033	Hd44.2_DRJ1R/L	Rep1_Hd44.2, U1_Hd44.2
scaffold128243	5513913	Hd44.1	3009	4203985-4206993	Hd44.1_DRJ1R/L	U1_Hd44.1
scaffold351	5773413	Hd18	4696	2681961-2686656	Hd18_DRJ1R/L	N1_Hd18
scaffold351	5773413	Hd17	7730	2329273-2337002	Hd17_DRJ1R/L, Hd17_DRJ2R/L	Rep1_Hd17, Rep2_Hd17, Rep3_Hd17, Rep4_Hd17, Rep5_Hd17
scaffold22	5910489	Hd39	4122	1002620-1006741	Hd39_DRJ1R/L, Hd39_DRJ2R/L	Rep1_Hd39

scaffold128241	6033897	Hd13	5757	402487-408243	Hd13_DRJ1R/L	Cys1_Hd13, Cys2_Hd13
scaffold67	6283432	Hd30	4164	5867182-5871345	Hd30_DRJ1R/L, Hd30_DRJ2R/L	Vinx1_Hd30, U1_Hd30, U2_Hd30, U3_Hd30
scaffold184	7628668	Hd45.2	2051	3564791-3566841	no repeat found at extremities	U1_Hd45.2
scaffold184	7628668	Hd41	7953	3768924-3776876	Hd41_DRJ1R/L, Hd41_DRJ2R/L	U1_Hd41, U2_Hd41, U3_Hd41
scaffold175	12456768	Hd36	3738	3796140-3799877	Hd36_DRJ1R/L	U1_Hd36, Vinx1_Hd36
scaffold175	12456768	Hd38	3664	3800393-3804056	Hd38_DRJ1R/L	U1_Hd38, Vinx1_Hd38, U2_Hd38
scaffold175	12456768	Hd26	5018	10942034-10947051	Hd26_DRJ1R/L, Hd26_DRJ2R/L	PRRP1_Hd26, PRRP2_Hd26, U1_Hd26
scaffold127548	15678958	Hd6	10461	5808388-5818848	Hd6_DRJ1R/int/L, Hd6_DRJ2R/L	U1.1_Hd6, P30_Hd6, Rep1_Hd6, U1.2_Hd6
scaffold127548	15678958	Hd2	13937	5940296-5954232	Hd2_DRJ1R/int/L, Hd2_DRJ2R/L	GlyPro1_Hd2, U1_Hd2, U2_Hd2, GlyPro2_Hd2, SerThr1_Hd2
scaffold127548	15678958	Hd7	8066	6062921-6070986	Hd7_DRJ1R/L	U1_Hd7
scaffold127548	15678958	Hd_IVSPER-4	15811	6832835-6848646	not applicable	U29, U30, U31, U32, U33, U34
scaffold127548	15678958	Hd_IVSPER-3	25432	10761570-10787001	not applicable	U15, IVSP3-2, U16, U17, U18, p12-1, U19, IVSP4-2, U20, U21, U22, U23, U28, p53-1, U24, N-2
scaffold127548	15678958	Hd_IVSPER-5	1629	10860001-10861630	not applicable	U35, U36
scaffold127548	15678958	Hd47	4503	12134587-12139089	Hd47_DRJ1R/L	Rep1_Hd47, Rep2_Hd47
scaffold127548	15678958	Hd5	13713	12941247-12954959	Hd5_DRJ1R/int-1/int-2/L	Vinx1_Hd5, Vinx2_Hd5, U1_Hd5, Vinx3_Hd5, Vinx4_Hd5, Vinx5_Hd5, Vinx6_Hd5
scaffold127548	15678958	Hd27	4002	13338255-13342256	Hd27_DRJ1R/L	U1_Hd27, K19_Hd27
Campoletis sonorensis						
scaffold_8749	2269	rep gene	NA	not applicable	not applicable	rep gene 1
scaffold_8748	2287	rep gene	NA	not applicable	not applicable	rep gene 2
scaffold_7280	2380	CsV	NA	not applicable	not applicable	cys_CsV, partial
scaffold_8362	5297	CsX5, partial	>5297	1-5297	no repeat searched	rep1_CsX5, rep2_CsX5
scaffold_4391	18751	CsL	10024	8079-18102	CsL_DRJ1R/L	cys_CsL
scaffold_5218	32996	CsP	12113	15720-27832	CsP_DRJ1R/L	vank4_CsP, vank3_CsP, vank2_CsP, vank1_CsP
scaffold_5934	46742	CsX1	17335	19391-36725	CsX1_DRJ1R/int1/int2/L	vank1_CsX1, vnx1_CsX1, rep1_CsX1, vank2_CsX1, vank3_CsX1, vank4_CsX1, vnx2_CsX1, rep2_CsX1, vank5_CsX1
scaffold_128	152063	CsI2	9042	110016-119057	CsI2_DRJ1R/L	vank1_CsI2, rep_CsI2, vank2_CsI2, vank3_CsI2
scaffold_35	217784	CsX8	9999	164467-174465	CsX8_DRJ1R/L	HP1_CsX8, cys_CsX8, HP2_CsX8
scaffold_5890	247009	CsZ	15871	134147-150017	CsZ_DRJ1R/L	rep1_CsZ, rep2_CsZ, rep3_CsZ, rep4_CsZ, rep5_CsZ, rep6_CsZ, rep7_CsZ
scaffold_110	266287	CsX2	10806	248068-258873	CsX2_DRJ1R/L, CsX2_DRJ2R/L	vank1_CsX2, vank2_CsX2, vank3_CsX2, vank4_CsX2, rep_CsX2
scaffold_50	323180	Cs_IVSPER-3	8610	218627-227236	not applicable	IVSP4L-2, U4L, p53L-3, U5L, IVSP2L-2, CsN-3
scaffold_50	323180	CsQ	12543	290527-303069	CsQ_DRJ1R/int/L	rep1_CsQ, vinx1_CsQ, vinx2_CsQ, rep2_CsQ, rep3_CsQ, rep4_CsQ
scaffold_6122	412025	Cs_IVSPER-1	31594	122689-154282	not applicable	U15L, IVSP1L-1, U37L-1, U31L-1, U35L, Gf_U27L, U17L, p12L-1, U19L, IVSP4L-1, U22L, U23L, p53L-1, U24L, CsN-1
scaffold_6122	412025	IVSP_U36L	471	234803-235273	not applicable	U36L
scaffold_60	424299	CsX3, partial	>7876	304794-312669	no repeat found at extremities	rep1_CsX3, rep2_CsX3, rep3_CsX3, rep4_CsX3
scaffold_6095	433103	CsX7, partial	>6041	183042-189082	no repeat found at extremities	rep1_CsX7, rep2_CsX7, rep3_CsX7
scaffold_6070	495171	CsO1	12746	168701-181446	no repeat found at extremities	4rep_CsO1, 3rep_CsO1
scaffold_116	506917	CsT	23217	7789-31005	CsT_DRJ1R/L, CsT_DRJ2R/L	no gene found
scaffold_49	562263	CsB	6626	22030-28655	CsB_DRJ1R/L	rep_CsB
scaffold_14	725399	CsG2	8338	192247-200584	CsG2_DRJ1R/L	rep1_CsG2, rep2_CsG2, rep3_CsG2, rep4_CsG2
scaffold_14	725399	CsG	8656	76017-84672	CsG_DRJ1R/L	vnx_CsG
scaffold_28	729583	CsC	7350	25280-32629	no repeat found at extremities	overlap Cs_IVSPER-2
scaffold_28	729583	CsW	15807	614005-629811	CsW_DRJ1R/int1/int2/L	cys1_CsW, cys2_CsW, rep1_CsW, cys3_CsW, rep2_CsW, rep3_CsW
scaffold_28	729583	Cs_IVSPER-2	33269	7310-40578	not applicable	p53L-2, U6L, U7L, IVSP1L-2, IVSP3L, U31L-2, U8L, U9L, U16L, U10L, U11L, U12L, U13L, p12L-2, U3L, IVSP2L-1, U26L, CsN-2, U25L
scaffold_22	866858	CsI	8779	695663-704441	CsI_DRJ1R/L	rep1_CsI, rep2_CsI, rep3_CsI
scaffold_57	1098263	Cs_IVSPER-4	9937	383305-393241	not applicable	U30L, U34L, IVSP4L-3
scaffold_131	1180360	CsF	8155	808380-816534	CsF_DRJ1R/L	cys_CsF
scaffold_149	1226226	CsU	15338	374074-389411	CsU_DRJ1R/L	cys1_CsU, cys2_CsU, cys3_CsU, cys4_CsU, cys5_CsU
scaffold_11	1376756	CsA	6368	861628-867995	CsA_DRJ1R/L	cys_CsA
scaffold_17	1497664	CsE	7990	1330025-1338014	CsE_DRJ1R/L	rep1_CsE, rep2_CsE, rep3_CsE
scaffold_12	1572921	IVSP_U37L	1863	104145-106007	not applicable	U37L-2

scaffold_23	1817383	CsM	12197	1422227-1434423	CsM_DRJ1R/L	N_CsM
scaffold_5	1987530	CsN	10943	175169-186111	CsN_DRJ1R/L	N1_CsN, N2_CsN
scaffold_38	2232507	CsH	9050	1398066-1407115	CsH_DRJ1R/L	5rep_CsH
scaffold_16	3063130	Cs_IVSPER-5	3750	2424942-2428691	not applicable	IVSP1L-3, U2L, U1L
scaffold_16	3063130	CsX6	9213	504600-513812	CsX6_DRJ1R/L	rep1_CsX6, rep2_CsX6
scaffold_10	3211285	CsD	8168	961052-969219	CsD_DRJ1R/L	vnx_CsD
scaffold_15	5987914	CsJ	9484	2621922-2631405	CsJ_DRJ1R/int/L	rep1_CsJ, rep2_CsJ, rep3_CsJ
scaffold_13	6115246	CsX4, partial	>9181	1407384-1416564	no repeat found at extremities	rep1_CsX4, rep2_CsX4, rep3_CsX4
