# Supplementary material for: Genomic architecture of endogenous ichnoviruses reveals distinct evolutionary pathways leading to virus domestication in parasitic wasps
Source: BMC Biol. 2020 Jul 24;18:89. doi: 10.1186/s12915-020-00822-3 (PMC7379367; doi:10.1186/s12915-020-00822-3)
Supplement: Supplementary file 5 — Additional file 5: Table S7. List of ichnoviral genes identified in Hyposoter didymator and Campoletis sonorensis genome scaffolds containing at least on ichnovirus sequence. Are indicated the scaffold name, the name of the proviral segment or of the Ichnovirus structural protein encoding region (IVSPER) found in the scaffold, its length and position in the scaffold, the name of the gene, its position in the scaffold, if it contains or not introns, the size of the predicted protein, then the NCBI blast P search results (NCBI accession number and ID of the best match, the blast P e-value and the percentage of identities). Last column indicates comments, or notes reporting discrepancies in the genomic sequence compared with the original CDS sequence in NCBI database. [file 12915_2020_822_MOESM5_ESM.pdf]

Scaffold	Proviral segment /IVSPER	Proviral/IVSPER sequence size (nt)	Proviral/IVSPER position in scaffold	Gene name	Gene position in scaffold	Introns in gene (yes/no)	Predicted protein size (aa)	NCBI accession	protein ID	Length NCBI seq. (nt)	BlastP e-value	Identities (%)	Comments/ discrepancies compared to original NCBI sequence
**Hyposoter didymator**													
**HdIV proviral segment**													
scaffold49647	similar to Hd2	1537	1-1537 (partial)	Gly-Pro_P40-like,	1-1235 - strand	yes	315	AAD40678.1	P40 protein [Hyposoter didymator ichnovirus]	397	2,00E-168	282/318(89%)	
scaffold184	Hd45.2	2051	3564791-3566841	U1_Hd45.2	3565610-3566474 - strand	yes	135	AIK25614.1	D8 [Hyposoter didymator ichnovirus]	138	4,00E-34	82/140(59%)	
scaffold128243	Hd44.1	3009	4203985-4206993	U1_Hd44.1	4205686-4205997 + strand	no	104	AIK25616.1	U2 [Hyposoter didymator ichnovirus]	104	1,00E-65	101/104(97%)	
scaffold64	Hd42	3157	2320523-2323679	Rep1_Hd42	2322099-2322881 - strand	no	261	AIK25619.1	Rep1 [Hyposoter didymator ichnovirus]	261	0,00E+00	248/261(95%)	
scaffold128213	Hd23.2	3362	248624-251985	Rep1_Hd23.2	249754-250470 - strand	no	238	AIK25664.1	Rep1 [Hyposoter didymator ichnovirus]	263	1,00E-124	180/239(75%)	
scaffold127348	Hd40	3494	926528-930021	U2_Hd40	926730-927180 - strand	no	148	AIK25622.1	U2 [Hyposoter didymator ichnovirus]	115	2,00E-69	101/104(97%)	insertion within CDS
scaffold127348	Hd40	3494	926528-930021	U1_Hd40	928473-928898 - strand	no	142	AIK25621.1	U1 [Hyposoter didymator ichnovirus]	143	5,00E-75	116/142(82%)	
scaffold175	Hd38	3664	3800393-3804056	U1_Hd38	3800677-3801003 - strand	no	108	AIK25624.1	U1 [Hyposoter didymator ichnovirus]	108	3,00E-69	105/108(97%)	insertion of a stop codon within CDS
scaffold175	Hd38	3664	3800393-3804056	Vinx1_Hd38	3801226-3802356 + strand	no	377	AIK25625.1	Vinx1 [Hyposoter didymator ichnovirus]	377	0,00E+00	362/377(96%)	
scaffold175	Hd38	3664	3800393-3804056	U2_Hd38	3802480-3802809 - strand	no	109	AIK25626.1	F1U2 [Hyposoter didymator ichnovirus]	107	5,00E-68	101/107(94%)	insertion of a stop codon within CDS
scaffold65	Hd37	3708	342293-346000	U1_Hd37	342555-342665 - strand	no	36	AIK25627.1	U1 [Hyposoter didymator ichnovirus]	143	2,00E-16	36/36(100%)	partial; corresponds to the N-term of the protein
scaffold65	Hd37	3708	342293-346000	Rep1_Hd37	343245-344048 + strand	no	268	AIK25629.1	Rep1 [Hyposoter didymator ichnovirus]	268	0,00E+00	262/268(98%)	
scaffold65	Hd37	3708	342293-346000	U2_Hd37	344748-344996 - strand	no	82	AIK25628.1	U2 [Hyposoter didymator ichnovirus]	146	1,00E-46	76/82(93%)	partial; corresponds to the C-term of the protein
scaffold116	Hd35	3710	473341-477050	Rep1_Hd35	475117-475821 + strand	no	235	AIK25632.1	Rep1 [Hyposoter didymator ichnovirus]	235	9,00E-177	234/235(99%)	
scaffold175	Hd36	3738	3796140-3799877	U1_Hd36	3797417-3797740 + strand	no	107	AIK25630.1	F1U1 [Hyposoter didymator ichnovirus]	107	2,00E-70	102/107(95%)	start codon modified (ATT instead of ATG)
scaffold175	Hd36	3738	3796140-3799877	Vinx1_Hd36	3797918-3799006 - strand	no	363	AAO16963.1	viral innexin [Hyposoter didymator ichnovirus]	363	0,00E+00	335/363(92%)	
scaffold91	Hd33	3835	485063-488897	U1_Hd33	485760-486020 - strand	no	86		No significant similarity found				
scaffold91	Hd33	3835	485063-488897	U2_Hd33	487963-488427 - strand	no	154		No significant similarity found				
scaffold127548	Hd27	4002	13338255-13342256	U1_Hd27	13338842-13339126 + strand	no	94	AIK25654.1	U1 [Hyposoter didymator ichnovirus]	113	6,00E-62	92/93(99%)	modified start codon
scaffold127548	Hd27	4002	13338255-13342256	K19_Hd27	13340915-13341354 - strand	yes	106	AAF91314.1	P12 [Hyposoter didymator ichnovirus]	106	2,00E-71	105/106(99%)	
scaffold29771	Hd46	4109	19344-23452	Vank1_Hd46	20304-20807 + strand	no	167	YP_00103123	vankyrin-b1 [Hyposoter fugitivus ichnovirus]	167	9,00E-86	127/165(77%)	
scaffold127549	Hd31-34	4119	144445-148563	U1_Hd31-34	145530-145901 + strand	no	124	AIK25633.1	F4U1 [Hyposoter didymator ichnovirus]	124	1,00E-71	121/124(98%)	
scaffold127549	Hd31-34	4119	144445-148563	U2_Hd31-34	146442-146729 - strand	no	95	AIK25634.1	F5U2 [Hyposoter didymator ichnovirus]	124	3,00E-64	94/95(99%)	shorter in C-term
scaffold22	Hd39	4122	1002620-1006741	Rep1_Hd39	1003991-1004692 - strand	no	234	AIK25623.1	Rep1 [Hyposoter didymator ichnovirus]	234	9,00E-170	227/234(97%)	
scaffold1868	Hd43	4159	193639-197797	U1_Hd43	194377-194676 - strand	no	100	AIK25617.1	U1 [Hyposoter didymator ichnovirus]	100	3,00E-67	97/100(97%)	
scaffold1868	Hd43	4159	193639-197797	Vank1_Hd43	196094-196618 - strand	no	175	AIK25618.1	Vank1 [Hyposoter didymator ichnovirus]	175	1,00E-125	171/175(98%)	
scaffold67	Hd30	4164	5867182-5871345	U1_Hd30	5867451-5867846 + strand	no	131	AIK25641.1	U1 [Hyposoter didymator ichnovirus]	104	3,00E-67	100/104(96%)	longuer in C-term
scaffold67	Hd30	4164	5867182-5871345	U2_Hd30	5867871-5868737 - strand	no	288	AIK25642.1	U2 [Hyposoter didymator ichnovirus]	288	0,00E+00	284/288(99%)	
scaffold67	Hd30	4164	5867182-5871345	Vinx1_Hd30	5869217-5870347 - strand	no	376	AIK25643.1	Vinx1 [Hyposoter didymator ichnovirus]	376	0,00E+00	375/376(99%)	
scaffold67	Hd30	4164	5867182-5871345	U3_Hd30	5870662-5870985 + strand	no	107	AIK25644.1	U3 [Hyposoter didymator ichnovirus]	107	2,00E-68	105/107(98%)	
scaffold161	Hd25	4174	2449400-2453573	PRRP3_Hd25	2450118-2450525 + strand	no	136	AIK25660.1	PRRP2 [Hyposoter didymator ichnovirus]	136	1,00E-83	136/136(100%)	
scaffold161	Hd25	4174	2449400-2453573	PRRP2_Hd25	2451908-2452147 - strand	no	80	AIK25661.1	PRRP3 [Hyposoter didymator ichnovirus]	80	7,00E-46	75/80(94%)	
scaffold161	Hd25	4174	2449400-2453573	PRRP1_Hd25	2452642-2452827 - strand	no	61	AIK25659.1	PRRP1 [Hyposoter didymator ichnovirus]	61	4,00E-09	58/61(95%)	
scaffold28498	Hd45.1	NA	1-4214 (partial)	U1_Hd45.1	679-1541 - strand	yes	138	AAO33352.1	unknown [Hyposoter didymator ichnovirus]	138	3,00E-91	133/138(96%)	
scaffold28498	Hd45.1	NA	1-4214 (partial)	U2_Hd45.1	3317-4180 - strand	yes	135	AIK25614.1	D8 [Hyposoter didymator ichnovirus]	138	3,00E-33	82/140(59%)	
scaffold119	Hd22	4233	698410-702642	U1_Hd22	698775-699101 - strand	no	109	AIK25666.1	U1 [Hyposoter didymator ichnovirus]	109	2,00E-72	106/109(97%)	
scaffold119	Hd22	4233	698410-702642	Rep1_Hd22	700424-701035 - strand	no	204	AAR89180.1	repeat element protein 8 [Hyposoter didymator ichnovirus]	204	9,00E-150	204/204(100%)	
scaffold91	Hd29	4356	572514-576869	N1_Hd29	573680-574969 - strand	no	430	AIK25650.1	N-gene1 [Hyposoter didymator ichnovirus]	430	0,00E+00	427/430(99%)	
scaffold64	Hd21	4368	2353107-2357474	Rep1_Hd21	2353545-2354333 - strand	no	263	AIK25667.1	Rep1 [Hyposoter didymator ichnovirus]	263	0,00E+00	258/263(98%)	
scaffold64	Hd21	4368	2353107-2357474	Vinx1_Hd21	2355781-2356860 - strand	no	360	AIK25668.1	Vinx1 [Hyposoter didymator ichnovirus]	360	0,00E+00	356/360(99%)	
scaffold198	Hd19	4440	1265454-1269893	U1_Hd19	1266250-1268833 - strand	yes	618	AIK25671.1	U1 [Hyposoter didymator ichnovirus]	618	0,00E+00	606/618(98%)	
scaffold128213	Hd23.1	4457	208205-212661	Rep1_Hd23.1	209796-210587 - strand	no	263	AIK25664.1	Rep1 [Hyposoter didymator ichnovirus]	263	0,00E+00	262/263(99%)	
scaffold127548	Hd47	4503	12134587-12139089	Rep1_Hd47	12135113-12135841 - strand	no	242	YP_00103131	repeat element protein-d4.2 [Hyposoter fugitivus ichnovirus]	248	2,00E-122	166/238(70%)	
scaffold127548	Hd47	4503	12134587-12139089	Rep2_Hd47	12136931-12137599 - strand	no	222	YP_00103131	repeat element protein-d4.1 [Hyposoter fugitivus ichnovirus]	255	4,00E-116	154/220(70%)	
scaffold264	Hd28	4614	135485-140098	Vinx1_Hd28	136611-137621 + strand	no	336	AIK25662.1	Vinx1 [Hyposoter didymator ichnovirus]	357	0,00E+00	301/334(90%)	
scaffold264	Hd28	4614	135485-140098	Vank1_Hd28	138475-138984 + strand	no	170	AFH35114.1	vankyrin 1 [Hyposoter didymator ichnovirus]	170	2,00E-122	168/170(99%)	
scaffold82201	Hd51	4632	1077-5708	Rep1_Hd51	2537-3115 - strand	no	192	AIK25629.1	Rep1 [Hyposoter didymator ichnovirus]	268	2,00E-89	129/190(68%)	
scaffold351	Hd18	4696	2681961-2686656	N1_Hd18	2683737-2685050 + strand	no	438	AIK25675.1	N-gene1 [Hyposoter didymator ichnovirus]	438	0,00E+00	434/438(99%)	
scaffold91	Hd24	4697	535698-540394	Vank1_Hd24	536918-537427 - strand	no	170	AFH35112.1	vankyrin 1 [Hyposoter didymator ichnovirus]	170	1,00E-123	170/170(100%)	
scaffold91	Hd24	4697	535698-540394	Vinx1_Hd24	538200-539270 - strand	no	357	AIK25662.1	Vinx1 [Hyposoter didymator ichnovirus]	357	0,00E+00	351/357(98%)	
scaffold128243	Hd44.2	4831	4197203-4202033	Rep1_Hd44.2	4197838-4198440 - strand	no	200	YP_00103128	repeat element protein-c11.1 [Hyposoter fugitivus ichnovirus]	201	3,00E-58	106/186(57%)	

scaffold128243	Hd44.2	4831	4197203-4202033	U1_Hd44.2	4199950-4200297 + strand	no	115	AIG88525.1	hypothetical protein A7.1 [Diadegma fenestrale ichnovirus]	76	1,00E-20	56/76(74%)	strand may be incorrect
scaffold91	Hd15	4987	469105-474091	N1_Hd15	470135-471607 - strand	no	491	AIK25681.1	N-gene1 [Hyposoter didymator ichnovirus]	491	0,00E+00	478/491(97%)	
scaffold175	Hd26	5018	10942034-10947051	PRRP1_Hd26	10943182-10943589 + strand	no	136	AIK25646.1	PRRP1 [Hyposoter didymator ichnovirus]	136	2,00E-91	136/136(100%)	
scaffold175	Hd26	5018	10942034-10947051	PRRP2_Hd26	10945126-10945518 + strand	no	131	AIK25645.1	PRRP2 [Hyposoter didymator ichnovirus]	131	4,00E-83	130/131(99%)	
scaffold175	Hd26	5018	10942034-10947051	U1_Hd26	10946124-10946471 - strand	no	116	AIK25658.1	U1 [Hyposoter didymator ichnovirus]	116	2,00E-80	116/116(100%)	
scaffold64	Hd14	5196	36336-41531	Vinx1_Hd14	38977-39405 + strand	no	143	AIK25682.1	Vinx1 [Hyposoter didymator ichnovirus]	143	5,00E-101	143/143(100%)	
scaffold64	Hd14	5196	36336-41531	U1_Hd14	41196-41501 + strand	no	102	AIK25683.1	U1 [Hyposoter didymator ichnovirus]	102	8,00E-70	102/102(100%)	
scaffold128246	Hd49	5265	677866-683130	Rep1_Hd49	678304-679005 + strand	no	233	YP_001031325.1	repeat element protein-d7.2 [Hyposoter fugitivus ichnovirus]	242	1,00E-119	173/232(75%)	
scaffold128246	Hd49	5265	677866-683130	Rep2_Hd49	679573-680190 + strand	no	205	YP_001031326.1	repeat element protein-d7.3 [Hyposoter fugitivus ichnovirus]	240	3,00E-114	160/205(78%)	
scaffold128246	Hd49	5265	677866-683130	Rep3_Hd49	681373-682041 + strand	no	222	YP_001031323.1	repeat element protein-d7.1 [Hyposoter fugitivus ichnovirus]	255	5,00E-93	156/257(61%)	
scaffold128241	Hd13	5757	402487-408243	Cys2_Hd13	403839-404424 - strand	yes	157	AIK25685.1	Cys2 [Hyposoter didymator ichnovirus]	157	5,00E-94	133/157(85%)	
scaffold128241	Hd13	5757	402487-408243	Cys1_Hd13	406027-406868 - strand	yes	162	AIK25684.1	Cys1 [Hyposoter didymator ichnovirus]	162	1,00E-107	149/162(92%)	
scaffold357	Hd50	5787	2218373-2224159	Vinx1_Hd50	2219976-2221058 + strand	no	360	YP_001031228.1	innexin Vnx-d5.1 [Hyposoter fugitivus ichnovirus]	375	0,00E+00	280/357(78%)	
scaffold357	Hd50	5787	2218373-2224159	Vinx2_Hd50	2221574-2222647 + strand	no	357	YP_001031229.1	innexin Vnx-d5.2 [Hyposoter fugitivus ichnovirus]	378	7,00E-170	241/378(64%)	
scaffold59	Hd12	5902	674917-680818	U1_Hd12	675013-675345 + strand	no	111	AIK25686.1	U1 [Hyposoter didymator ichnovirus]	111	9,00E-77	110/111(99%)	
scaffold59	Hd12	5902	674917-680818	Rep1_Hd12	675996-676679 - strand	no	228	AIK25689.1	Rep1 [Hyposoter didymator ichnovirus]	228	1,00E-169	226/228(99%)	
scaffold59	Hd12	5902	674917-680818	U2_Hd12	678859-679170 - strand	no	104	AIK25687.1	U2 [Hyposoter didymator ichnovirus]	104	2,00E-67	103/104(99%)	
scaffold59	Hd12	5902	674917-680818	Rep2_Hd12	679544-680233 - strand	no	230	AIK25688.1	Rep2 [Hyposoter didymator ichnovirus]	230	1,00E-173	229/230(99%)	
scaffold59	Hd10	6507	2500377-2506883	Rep3_Hd10	2501422-2502045 - strand	no	208	AIK25696.1	Rep3 [Hyposoter didymator ichnovirus]	208	9,00E-154	207/208(99%)	
scaffold59	Hd10	6507	2500377-2506883	Rep2_Hd10	2502434-2503066 - strand	no	211	AIK25695.1	Rep2 [Hyposoter didymator ichnovirus]	211	3,00E-155	211/211(100%)	
scaffold59	Hd10	6507	2500377-2506883	Rep1_Hd10	2504201-2504872 - strand	no	224	AAR89179.1	repeat element protein 7 [Hyposoter didymator ichnovirus]	224	4,00E-167	224/224(100%)	
scaffold144	Hd20	6864	1449468-1456331	Cys2_Hd20	1450485-1451362 - strand	yes	145	AIK25636.1	Cys1 [Hyposoter didymator ichnovirus]	145	3,00E-104	144/145(99%)	
scaffold144	Hd20	6864	1449468-1456331	Cys1_Hd20	1454299-1455511 - strand	yes	254	AIK25669.1	Cys1 [Hyposoter didymator ichnovirus]	254	0,00E+00	252/254(99%)	
scaffold377	Hd8	7356	2186417-2193772	U5_Hd8	2186651-2186968 + strand	no	105	AIK25705.1	U4 [Hyposoter didymator ichnovirus]	105	2,00E-71	103/105(98%)	
scaffold377	Hd8	7356	2186417-2193772	U4_Hd8	2188345-2188632 - strand	no	95	YP_0010313€	e1.1 [Hyposoter fugitivus ichnovirus]	131	4,00E-07	46/131(35%)	
scaffold377	Hd8	7356	2186417-2193772	U3_Hd8	2190149-2190466 + strand	no	105	AIK25702.1	F6U1 [Hyposoter didymator ichnovirus]	105	5,00E-71	102/105(97%)	
scaffold377	Hd8	7356	2186417-2193772	U2_Hd8	2191793-2192095 - strand	no	100	AIK25703.1	U2 [Hyposoter didymator ichnovirus]	100	1,00E-64	98/100(98%)	
scaffold377	Hd8	7356	2186417-2193772	U1_Hd8	2192394-2192693 + strand	no	99	AIK25704.1	F6U3 [Hyposoter didymator ichnovirus]	106	6,00E-44	71/75(95%)	
scaffold59	Hd16	7704	690205-697908	Rep1_Hd16	690229-690951 - strand	no	241	AIK25680.1	Rep1 [Hyposoter didymator ichnovirus]	241	0,00E+00	241/241(100%)	
scaffold59	Hd16	7704	690205-697908	U2_Hd16	691425-691661 + strand	no	78		No significant similarity found				possibly not a CDS
scaffold59	Hd16	7704	690205-697908	U1_Hd16	693257-693817 + strand	no	187	AIK25679.1	U1 [Hyposoter didymator ichnovirus]	187	9,00E-122	186/187(99%)	
scaffold59	Hd16	7704	690205-697908	Rep2_Hd16	695154-695813 - strand	no	219	AAR89178.1	repeat element protein 6 [Hyposoter didymator ichnovirus]	219	1,00E-159	213/219(97%)	
scaffold59	Hd16	7704	690205-697908	Rep3_Hd16	696463-697149 - strand	no	228	AAR89177.1	repeat element protein 5 [Hyposoter didymator ichnovirus]	228	9,00E-170	228/228(100%)	
scaffold351	Hd17	7730	2329273-2337002	Rep5_Hd17	2329743-2330387 - strand	no	214	AHY22036.1	repeat element 36 [Diadegma semiclausum ichnovirus]	262	3,00E-124	174/214(81%)	
scaffold351	Hd17	7730	2329273-2337002	Rep4_Hd17	2330936-2331505 - strand	no	189	AIK25678.1	Rep1 [Hyposoter didymator ichnovirus]	230	1,00E-120	166/182(91%)	
scaffold351	Hd17	7730	2329273-2337002	Rep3_Hd17	2332279-2333010 - strand	no	244	AAO16957.1	repeat element protein [Hyposoter didymator ichnovirus]	244	0,00E+00	244/244(100%)	
scaffold351	Hd17	7730	2329273-2337002	Rep2_Hd17	2334085-2334759 - strand	no	225	AAO16959.1	repeat element protein [Hyposoter didymator ichnovirus]	225	5,00E-166	221/225(98%)	
scaffold351	Hd17	7730	2329273-2337002	Rep1_Hd17	2335459-2336166 - strand	no	236	AIK25673.1	Rep1 [Hyposoter didymator ichnovirus]	236	4,00E-173	232/236(98%)	
scaffold64	Hd32	7916	88702-96617	U1.2_Hd32	88842-89159 - strand	no	105	AIK25638.1	U1 [Hyposoter didymator ichnovirus]	105	5,00E-65	96/105(91%)	
scaffold64	Hd32	7916	88702-96617	Vinx2_Hd32	90142-91254 - strand	no	370	AIK25637.1	Vinx1 [Hyposoter didymator ichnovirus]	366	0,00E+00	279/353(79%)	
scaffold64	Hd32	7916	88702-96617	U1.1_Hd32	93443-93757 - strand	no	105	AIK25638.1	U1 [Hyposoter didymator ichnovirus]	105	6,00E-72	105/105(100%)	
scaffold64	Hd32	7916	88702-96617	Vinx1_Hd32	94429-95526 - strand	no	366	AIK25637.1	Vinx1 [Hyposoter didymator ichnovirus]	366	0,00E+00	366/366(100%)	
scaffold184	Hd41	7953	3768924-3776876	U1_Hd41	3769919-3770824 + strand	yes	152	AIK25620.1	U1 [Hyposoter didymator ichnovirus]	197	9,00E-64	112/152(74%)	N-term shorter
scaffold184	Hd41	7953	3768924-3776876	U2_Hd41	3772675-3773516 + strand	yes	168	AIK25614.1	D8 [Hyposoter didymator ichnovirus]	138	3,00E-22	72/170(42%)	
scaffold184	Hd41	7953	3768924-3776876	U3_Hd41	3775212-3776089 + strand	yes	159	AIK25620.1	U1 [Hyposoter didymator ichnovirus]	197	2,00E-22	59/96(61%)	
scaffold127548	Hd7	8066	6062921-6070986	U1_Hd7	6065301-6068372 + strand	no	1024	AIK25706.1	U1 [Hyposoter didymator ichnovirus]	1080	0,00E+00	916/1080(85%)	repeated motif missing from the CDS
scaffold59	Hd11	9190	2183512-2192701	U1_Hd11	2184311-2184694 + strand	no	128	AIK25649.1	U1 [Hyposoter didymator ichnovirus]	128	8,00E-87	125/128(98%)	
scaffold59	Hd11	9190	2183512-2192701	Rep1_Hd11	2184915-2185607 + strand	no	231	AIK25648.1	Rep1 [Hyposoter didymator ichnovirus]	231	7,00E-172	229/231(99%)	
scaffold59	Hd11	9190	2183512-2192701	Vank1_Hd11	2186326-2186802 + strand	no	159	AFH35113.1	vankyrin 1 [Hyposoter didymator ichnovirus]	159	2,00E-113	159/159(100%)	
scaffold59	Hd11	9190	2183512-2192701	Vank5_Hd11	2187892-2188395 + strand	no	168	AFH35119.1	vankyrin 5 [Hyposoter didymator ichnovirus]	168	2,00E-123	168/168(100%)	
scaffold59	Hd11	9190	2183512-2192701	Vank4_Hd11	2189456-2189962 + strand	no	169	AFH35118.1	vankyrin 4 [Hyposoter didymator ichnovirus]	169	4,00E-123	168/169(99%)	

scaffold59	Hd11	9190	2183512-2192701	Vank3_Hd11	2190561-2191067 + strand	no	169	AFH35117.1	vankyrin 3 [Hyposoter didymator ichnovirus]	169	1,00E-121	168/169(99%)	
scaffold59	Hd11	9190	2183512-2192701	Vank2_Hd11	2191436-2191942 + strand	no	169	AFH35116.1	vankyrin 2 [Hyposoter didymator ichnovirus]	169	2,00E-116	163/169(96%)	
scaffold59	Hd11	9190	2183512-2192701	Vank1p_Hd11	2192415-2192701 + strand	no	95	AFH35113.1	vankyrin 1 [Hyposoter didymator ichnovirus]	159	2,00E-58	88/95(93%)	partial; corresponds to the N-term of the protein
scaffold429	Hd48	9673	531833-541505	Rep1_Hd48	533753-534487 - strand	no	244	AHY22018.1	repeat element 27 [Diadegma semiclausum ichnovirus]	246	3,00E-127	175/245(71%)	
scaffold429	Hd48	9673	531833-541505	Rep2_Hd48	538812-539549 - strand	no	245	YP_001031283.1	repeat element protein-c7.1 [Hyposoter fugitivus ichnovirus]	282	4,00E-133	179/244(73%)	
scaffold65	Hd3	10014	437082-447095	Cys1_Hd3	438828-439558 - strand	yes	181	AIK25727.1	Cys1 [Hyposoter didymator ichnovirus]	181	4,00E-136	181/181(100%)	
scaffold65	Hd3	10014	437082-447095	Cys2_Hd3	440422-441241 - strand	yes	200	AIK25728.1	Cys2 [Hyposoter didymator ichnovirus]	200	1,00E-147	200/200(100%)	
scaffold65	Hd3	10014	437082-447095	Cys3_Hd3	442538-443060 - strand	yes	147	AIK25729.1	Cys3 [Hyposoter didymator ichnovirus]	147	3,00E-105	144/147(98%)	
scaffold65	Hd3	10014	437082-447095	Cys4_Hd3	443574-444657 - strand	yes	267	AIK25730.1	Cys4 [Hyposoter didymator ichnovirus]	241	3,00E-168	232/267(87%)	
scaffold65	Hd3	10014	437082-447095	Cys5_Hd3	445264-445755 - strand	yes	125	AIK25731.1	Cys5 [Hyposoter didymator ichnovirus]	125	3,00E-83	117/125(94%)	
scaffold377	Hd4	10326	2459681-2470006	Rep1_Hd4	2461072-2461818 - strand	no	248	AIK25721.1	Rep1 [Hyposoter didymator ichnovirus]	248	0,00E+00	248/248(100%)	
scaffold377	Hd4	10326	2459681-2470006	Rep2_Hd4	2462404-2463012 + strand	no	202	AIK25722.1	Rep2 [Hyposoter didymator ichnovirus]	202	8,00E-144	197/202(98%)	
scaffold377	Hd4	10326	2459681-2470006	Rep3_Hd4	2463770-2464366 + strand	no	198	AIK25723.1	Rep3 [Hyposoter didymator ichnovirus]	198	2,00E-145	198/198(100%)	
scaffold377	Hd4	10326	2459681-2470006	Rep4_Hd4	2465099-2465706 + strand	no	202	AIK25724.1	Rep4 [Hyposoter didymator ichnovirus]	202	6,00E-150	202/202(100%)	
scaffold377	Hd4	10326	2459681-2470006	Rep5_Hd4	2466854-2467603 - strand	no	249	AIK25725.1	Rep5 [Hyposoter didymator ichnovirus]	249	0,00E+00	246/249(99%)	
scaffold377	Hd4	10326	2459681-2470006	Rep6_Hd4	2468587-2469195 + strand	no	202	AIK25726.1	Rep6 [Hyposoter didymator ichnovirus]	202	3,00E-149	200/202(99%)	
scaffold127548	Hd6	10461	5808388-5818848	Rep1_Hd6	5809242-5809907 + strand	no	221	AAO33572.1	rep protein [Hyposoter didymator ichnovirus]	221	1,00E-164	221/221(100%)	
scaffold127548	Hd6	10461	5808388-5818848	P30_Hd6	5810559-5811315 - strand	yes	159	AIK25713.1	P30 [Hyposoter didymator ichnovirus]	414	2,00E-64	121/131(92%)	repeated motifs missing from the CDS
scaffold127548	Hd6	10461	5808388-5818848	U1.1_Hd6	5814030-5814758 - strand	yes	151	AIK25712.1	U1 [Hyposoter didymator ichnovirus]	159	2,00E-94	151/159(95%)	
scaffold127548	Hd6	10461	5808388-5818848	U1.2_Hd6	5818232-5818829 - strand	yes	143	AIK25712.1	U1 [Hyposoter didymator ichnovirus]	159	7,00E-12	53/129(41%)	
scaffold127548	Hd5	10510	12941242-12951751	Vinx1_Hd5	12941923-12942945 - strand	no	340	AIK25715.1	Vinx1 [Hyposoter didymator ichnovirus]	340	0,00E+00	336/340(99%)	
scaffold127548	Hd5	10510	12941242-12951751	Vinx2_Hd5	12943091-12943896 - strand	no	267	AIK25716.1	Vinx2 [Hyposoter didymator ichnovirus]	180	1,00E-99	141/143(99%)	
scaffold127548	Hd5	10510	12941242-12951751	U1_Hd5	12944615-12944923 - strand	no	102	AIK25717.1	U1 [Hyposoter didymator ichnovirus]	102	1,00E-65	98/102(96%)	
scaffold127548	Hd5	10510	12941242-12951751	Vinx3_Hd5	12945139-12946200 - strand	no	353	AIK25718.1	Vinx3 [Hyposoter didymator ichnovirus]	353	0,00E+00	352/353(99%)	
scaffold127548	Hd5	10510	12941242-12951751	Vinx4_Hd5	12947754-12948800 - strand	no	348	AIK25719.1	Vinx4 [Hyposoter didymator ichnovirus]	348	0,00E+00	346/348(99%)	
scaffold127548	Hd5	10510	12941242-12951751	Vinx5_Hd5	12950651-12951697 - strand	no	348	AAR82838.1	innexin-like protein 2 [Hyposoter didymator ichnovirus]	348	0,00E+00	346/348(99%)	
scaffold127548	Hd5	10510	12941242-12951751	Vinx6_Hd5	12952927-12953967 - strand	no	346	AAR82840.1	innexin-like protein 4 [Hyposoter didymator ichnovirus]	393	0,00E+00	334/343(97%)	
scaffold127548	Hd2	13937	5940296-5954232	GlyPro1_Hd2	5941600-5943451 + strand	yes	452	AAF08193.1	glycine and proline-rich protein P45 precursor [Hyposoter didymator ichnovirus]	452	0,00E+00	451/452(99%)	
scaffold127548	Hd2	13937	5940296-5954232	U1_Hd2	5944265-5944609 - strand	no	115	AIK25733.1	F2U1 [Hyposoter didymator ichnovirus]	115	5,00E-77	115/115(100%)	
scaffold127548	Hd2	13937	5940296-5954232	U2_Hd2	5945077-5945436 + strand	no	120	AAO33351.1	unknown [Hyposoter didymator ichnovirus]	120	8,00E-79	120/120(100%)	
scaffold127548	Hd2	13937	5940296-5954232	GlyPro2_Hd2	5946971-5951238 + strand	yes	1357	AAF08192.1	glycine and proline-rich protein P69 precursor [Hyposoter didymator ichnovirus]	683	6,00E-157	417/786(53%)	differences in the repeated region
scaffold127548	Hd2	13937	5940296-5954232	SerThr1_Hd2	5952553-5953461 + strand	yes	216	AIK25709.1	SerThr [Hyposoter didymator ichnovirus]	216	2,00E-153	211/216(98%)	
scaffold90	Hd1	NA	1-14771 (partial)	U6_Hd1	3039-3506 + strand	no	156	AIK25750.1	F9U13 [Hyposoter didymator ichnovirus]	156	6,00E-109	153/156(98%)	
scaffold90	Hd1	NA	1-14771 (partial)	U5_Hd1	4779-5282 - strand	no	168	AIK25739.1	F8U2 [Hyposoter didymator ichnovirus]	168	9,00E-119	168/168(100%)	
scaffold90	Hd1	NA	1-14771 (partial)	U4_Hd1	6629-7054 + strand	no	142	AIK25738.1	F7U1 [Hyposoter didymator ichnovirus]	142	2,00E-99	142/142(100%)	
scaffold90	Hd1	NA	1-14771 (partial)	U3_Hd1	8197-8511 - strand	no	105	AIK25745.1	F11U8 [Hyposoter didymator ichnovirus]	106	2,00E-46	82/106(77%)	
scaffold90	Hd1	NA	1-14771 (partial)	U2_Hd1	8984-9325 - strand	no	114	AIK25746.1	U9 [Hyposoter didymator ichnovirus]	114	2,00E-79	114/114(100%)	
scaffold90	Hd1	NA	1-14771 (partial)	U1_Hd1	12650-12967 - strand	no	105	AIK25745.1	F11U8 [Hyposoter didymator ichnovirus]	106	2,00E-46	82/106(77%)	
scaffold128215	Hd9	17892	526917-544808	U1.3_Hd9	527725-528057 - strand	no	110	AIK25698.1	U1 [Hyposoter didymator ichnovirus]	111	3,00E-32	81/110(74%)	
scaffold128215	Hd9	17892	526917-544808	U5.2_Hd9	529943-530425 + strand	no	160		No significant similarity found				
scaffold128215	Hd9	17892	526917-544808	U4.2_Hd9	531702-532277 - strand	no	191	AIK25701.1	U4 [Hyposoter didymator ichnovirus]	116	1,00E-48	99/116(85%)	
scaffold128215	Hd9	17892	526917-544808	U3.2_Hd9	532913-533263 + strand	no	117	AIK25700.1	U3 [Hyposoter didymator ichnovirus]	117	9,00E-76	112/117(96%)	
scaffold128215	Hd9	17892	526917-544808	U6.2_Hd9	533349-533735 + strand	no	128	AIK25748.1	F7U11 [Hyposoter didymator ichnovirus]	111	6,00E-18	56/126(44%)	
scaffold128215	Hd9	17892	526917-544808	U1.2_Hd9	535076-535489 - strand	no	137	AIK25698.1	U1 [Hyposoter didymator ichnovirus]	111	2,00E-30	76/110(69%)	
scaffold128215	Hd9	17892	526917-544808	U5.1_Hd9	537399-537881 + strand	no	160		No significant similarity found				
scaffold128215	Hd9	17892	526917-544808	U4.1_Hd9	539200-539775 - strand	no	191	AIK25701.1	U4 [Hyposoter didymator ichnovirus]	116	5,00E-48	99/116(85%)	
scaffold128215	Hd9	17892	526917-544808	U3.1_Hd9	540411-540941 + strand	no	176	AIK25700.1	U3 [Hyposoter didymator ichnovirus]	117	2,00E-72	110/117(94%)	
scaffold128215	Hd9	17892	526917-544808	U6.1_Hd9	540847-541233 + strand	no	128	AIK25748.1	F7U11 [Hyposoter didymator ichnovirus]	111	8,00E-18	60/128(47%)	
scaffold128215	Hd9	17892	526917-544808	U2_Hd9	541468-541782 - strand	no	105	AIK25699.1	U2 [Hyposoter didymator ichnovirus]	104	3,00E-67	102/105(97%)	
scaffold128215	Hd9	17892	526917-544808	U1.1_Hd9	543144-543476 - strand	no	111	AIK25698.1	U1 [Hyposoter didymator ichnovirus]	111	3,00E-70	110/111(99%)	
***H. didymator* IVSPER**													
scaffold127548	Hd_IVSPER-5	1629	10860001-10861630	U35	10860001-10860537 + strand	no	178	AKD28058.1	hypothetical protein [Glypta fumiferanae] gene="U26"	180	1,00E-29	60/150(40%)	transcribed in calyx
scaffold127548	Hd_IVSPER-5	1629	10860001-10861630	U36	10861166-10861630 - strand	no	154	AKD28080.1	hypothetical protein [Glypta fumiferanae] gene="U38"	175	3,00E-14	45/144(31%)	transcribed in calyx
scaffold29771	IVSP_U37	1839	16848-18686	single CDS: U37	16848-18686 + strand	no	612	AKD28048.1	helicase-primase domain [Glypta fumiferanae]	721	0,00E+00	340/625(54%)	

scaffold91	Hd_IVSPER-1	14020	453857-467876	U1	453857-454591 + strand	no	244	ADI40452.1	unknown [Hyposoter didymator]	244	0,00E+00	241/243(99%)	
scaffold91	Hd_IVSPER-1	14020	453857-467876	IVSP1-1	455614-456375 + strand	no	253	ADI40453.1	unknown [Hyposoter didymator]	253	0,00E+00	245/253(97%)	
scaffold91	Hd_IVSPER-1	14020	453857-467876	U2	456897-457582 + strand	no	228	ADI40454.1	unknown [Hyposoter didymator]	228	3,00E-171	228/228(100%)	
scaffold91	Hd_IVSPER-1	14020	453857-467876	U3	458215-458709 + strand	no	164	ADI40455.1	unknown [Hyposoter didymator]	162	2,00E-119	162/162(100%)	
scaffold91	Hd_IVSPER-1	14020	453857-467876	U4	459123-459518 + strand	no	131	ADI40456.1	unknown [Hyposoter didymator]	131	5,00E-91	130/131(99%)	
scaffold91	Hd_IVSPER-1	14020	453857-467876	p53-2	460033-461034 + strand	no	333	ADI40457.1	unknown [Hyposoter didymator]	333	0,00E+00	329/333(99%)	
scaffold91	Hd_IVSPER-1	14020	453857-467876	U5	461470-462000 + strand	no	176	ADI40458.1	unknown [Hyposoter didymator]	176	6,00E-129	175/176(99%)	
scaffold91	Hd_IVSPER-1	14020	453857-467876	IVSP2-1	462381-463910 + strand	no	509	ADI40459.1	unknown [Hyposoter didymator]	509	0,00E+00	508/509(99%)	
scaffold91	Hd_IVSPER-1	14020	453857-467876	N-1	464563-466050 + strand	no	495	ADI40460.1	unknown [Hyposoter didymator]	470	0,00E+00	467/470(99%)	N-term longuer
scaffold91	Hd_IVSPER-1	14020	453857-467876	U25	467172-467876 - strand	no	234	AKD28083.1	ring finger domain [Glypta fumiferanae]	237	7,00E-14	37/118(31%)	putative IVSPER gene; may be wasp gene
scaffold127548	Hd_IVSPER-4	15811	6832835-6848646	U29	6832835-6833713 - strand	no	292		No significant similarity found				highly transcribed in calyx
scaffold127548	Hd_IVSPER-4	15811	6832835-6848646	U30	6834523-6838368 - strand	no	1281	AKD28060.1	hypothetical protein [Glypta fumiferanae] gene="U28"	1322	0,00E+00	616/1339(46%)	
scaffold127548	Hd_IVSPER-4	15811	6832835-6848646	U31	6839646-6840788 + strand	no	380	AKD28063.1	hypothetical protein [Glypta fumiferanae] gene="U31"	370	8,00E-27	93/323(29%)	transcribed in calyx
scaffold127548	Hd_IVSPER-4	15811	6832835-6848646	U32	6841304-6841912 + strand	no	202		No significant similarity found				transcribed in calyx
scaffold127548	Hd_IVSPER-4	15811	6832835-6848646	U33	6842790-6844940 - strand	no	716		No significant similarity found				transcribed in calyx
scaffold127548	Hd_IVSPER-4	15811	6832835-6848646	U34	6845548-6848646 - strand	no	1032	AKD28054.1	helicase domain [Glypta fumiferanae]	1012	0,00E+00	488/1040(47%)	
scaffold127548	Hd_IVSPER-3	25432	10761570-10787001	U15	10761570-10762802 - strand	no	410	ADI40477.1	unknown [Hyposoter didymator]	410	0,00E+00	407/410(99%)	
scaffold127548	Hd_IVSPER-3	25432	10761570-10787001	IVSP3-2	10763133-10765037 + strand	no	634	ADI40478.1	unknown [Hyposoter didymator]	527	0,00E+00	526/527(99%)	N-term 100aa longuer
scaffold127548	Hd_IVSPER-3	25432	10761570-10787001	U16	10765894-10767732 - strand	no	612	ADI40479.1	unknown [Hyposoter didymator]	612	0,00E+00	612/612(100%)	
scaffold127548	Hd_IVSPER-3	25432	10761570-10787001	U17	10768078-10768325 + strand	no	82	ADI40480.1	unknown [Hyposoter didymator]	82	1,00E-53	82/82(100%)	
scaffold127548	Hd_IVSPER-3	25432	10761570-10787001	U18	10769122-10769379 + strand	no	85	ADI40481.1	unknown [Hyposoter didymator]	85	7,00E-53	84/85(99%)	
scaffold127548	Hd_IVSPER-3	25432	10761570-10787001	p12-1	10770065-10770298 + strand	no	77	CAR31591.1	p12-like1 protein [Hyposoter didymator]	77	6,00E-45	77/77(100%)	
scaffold127548	Hd_IVSPER-3	25432	10761570-10787001	U19	10771340-10773322 + strand	no	660	ADI40483.1	unknown [Hyposoter didymator]	660	0,00E+00	658/660(99%)	
scaffold127548	Hd_IVSPER-3	25432	10761570-10787001	IVSP4-2	10774112-10775407 - strand	no	431	ADI40484.1	unknown [Hyposoter didymator]	431	0,00E+00	430/431(99%)	
scaffold127548	Hd_IVSPER-3	25432	10761570-10787001	U20	10776029-10776240 + strand	no	70	ADI40485.1	unknown [Hyposoter didymator]	70	6,00E-44	69/70(99%)	
scaffold127548	Hd_IVSPER-3	25432	10761570-10787001	U21	10776822-10777061 + strand	no	79	ADI40486.1	unknown [Hyposoter didymator]	79	4,00E-47	77/79(97%)	
scaffold127548	Hd_IVSPER-3	25432	10761570-10787001	U22	10777309-10778172 - strand	no	287	ADI40487.1	unknown [Hyposoter didymator]	257	0,00E+00	255/257(99%)	N-term longuer
scaffold127548	Hd_IVSPER-3	25432	10761570-10787001	U28	10778369-10779088 + strand	no	239		No significant similarity found				newly identified, putative
scaffold127548	Hd_IVSPER-3	25432	10761570-10787001	U23	10779153-10780400 - strand	no	415	ADI40488.1	unknown [Hyposoter didymator]	415	0,00E+00	408/415(98%)	
scaffold127548	Hd_IVSPER-3	25432	10761570-10787001	p53-1	10780635-10781822 + strand	no	395	CAR31590.1	p53-like1 protein [Hyposoter didymator]	395	0,00E+00	391/395(99%)	
scaffold127548	Hd_IVSPER-3	25432	10761570-10787001	U24	10782094-10783785 - strand	no	563	ADI40490.1	unknown [Hyposoter didymator]	563	0,00E+00	561/563(99%)	
scaffold127548	Hd_IVSPER-3	25432	10761570-10787001	N-2	10785523-10787001 + strand	no	492	ADI40491.1	unknown [Hyposoter didymator]	492	0,00E+00	490/492(99%)	
scaffold91	Hd_IVSPER-2	26611	541304-567914	N-4	541304-542734 - strand	no	476	ADI40491.1	unknown [Hyposoter didymator] gene="N-2"	492	0,00E+00	277/491(56%)	
scaffold91	Hd_IVSPER-2	26611	541304-567914	U26	542904-543740 - strand	no	278	AKD28081.1	hypothetical protein [Glypta fumiferanae] Gf_U39	298	9,00E-30	59/139(42%)	
scaffold91	Hd_IVSPER-2	26611	541304-567914	IVSP2-2	544341-545867 - strand	no	508	ADI40476.1	unknown, partial [Hyposoter didymator]	143	5,00E-96	143/143(100%)	completed IVSP2-2 sequence
scaffold91	Hd_IVSPER-2	26611	541304-567914	IVSP4-1	546767-548107 + strand	no	446	ADI40475.1	unknown [Hyposoter didymator]	446	0,00E+00	443/446(99%)	
scaffold91	Hd_IVSPER-2	26611	541304-567914	p12-2	548725-549039 - strand	no	104	ADI40474.1	unknown [Hyposoter didymator]	104	6,00E-69	103/104(99%)	
scaffold91	Hd_IVSPER-2	26611	541304-567914	U27	549438-549740 + strand	no	100		No significant similarity found				
scaffold91	Hd_IVSPER-2	26611	541304-567914	U14	549917-550078 - strand	no	53	ADI40473.1	unknown [Hyposoter didymator]	53	3,00E-29	53/53(100%)	
scaffold91	Hd_IVSPER-2	26611	541304-567914	p12-3	550341-550643 - strand	no	100	ADI40472.1	unknown [Hyposoter didymator]	100	8,00E-68	100/100(100%)	
scaffold91	Hd_IVSPER-2	26611	541304-567914	U13	551219-551677 - strand	no	152	ADI40471.1	unknown [Hyposoter didymator]	152	1,00E-107	152/152(100%)	
scaffold91	Hd_IVSPER-2	26611	541304-567914	U12	551947-552558 + strand	no	203	ADI40470.1	unknown [Hyposoter didymator]	203	1,00E-147	203/203(100%)	
scaffold91	Hd_IVSPER-2	26611	541304-567914	U11	553154-554104 - strand	no	316	ADI40469.1	unknown [Hyposoter didymator]	316	0,00E+00	312/316(99%)	
scaffold91	Hd_IVSPER-2	26611	541304-567914	U10	555456-559523 - strand	no	1355	ADI40468.1	unknown [Hyposoter didymator]	1355	0,00E+00	1352/1355(99%)	
scaffold91	Hd_IVSPER-2	26611	541304-567914	U9	559893-560717 + strand	no	274	ADI40467.1	unknown [Hyposoter didymator]	274	0,00E+00	274/274(100%)	
scaffold91	Hd_IVSPER-2	26611	541304-567914	U8	561319-561552 + strand	no	77	ADI40466.1	unknown [Hyposoter didymator]	77	3,00E-47	77/77(100%)	
scaffold91	Hd_IVSPER-2	26611	541304-567914	IVSP3-1	562225-564117 - strand	no	630	ADI40465.1	unknown [Hyposoter didymator]	630	0,00E+00	625/630(99%)	
scaffold91	Hd_IVSPER-2	26611	541304-567914	IVSP1-2	564643-565380 - strand	no	245	ADI40464.1	unknown [Hyposoter didymator]	196	5,00E-141	194/196(99%)	N-term longuer
scaffold91	Hd_IVSPER-2	26611	541304-567914	U7	565507-566145 - strand	no	212	ADI40463.1	unknown [Hyposoter didymator]	212	4,00E-157	212/212(100%)	
scaffold91	Hd_IVSPER-2	26611	541304-567914	U6	566499-567548 - strand	no	349	ADI40462.1	unknown [Hyposoter didymator]	349	0,00E+00	348/349(99%)	
scaffold91	Hd_IVSPER-2	26611	541304-567914	N-3	567663-567944 - strand	no	93	ADI40461.1	unknown [Hyposoter didymator]	93	1,00E-60	90/93(97%)	
**Campoletis sonorensis**													
**CsIV proviral segment**													
scaffold_8749	rep gene	2269	na	rep gene 1	45-674 + strand	no	209	AHY22033.1	repeat element 33 [Diadegma semiclausum i	243	3,00E-64	101/213(47%)	
scaffold_8748	rep gene	2287	na	rep gene 2	1596-2286 - strand	no	229	AHY22033.1	repeat element 33 [Diadegma semiclausum i	243	4,00E-72	114/223(51%)	partial?
scaffold_7280	CsV	2380	not applicable	cys_CsV, partial	1546-nn + strand	yes	NA	YP_589077.1	VHv1.4 protein [Campoletis sonorensis ichno	322			
scaffold_11	CsA	6368	861628-867995	HP_CsA	861717-862004 - strand	no	95		no significant similarity				
scaffold_11	CsA	6368	861628-867995	cys_CsA	864776-865537 - strand	yes	177	AAO43443.1	A'Hv0.8 cys-motif protein precursor [Campole	177	8,00E-127	173/177(98%)	
scaffold_49	CsB	6626	22030-28655	rep_CsB	23475-24182 - strand	no	235	AAA42923.1	repeat element protein [Campoletis sonorens	235	2,00E-174	235/235(100%)	

scaffold_49	CsB	6626	22030-28655	HP_CsB	26564-27903 + strand	yes	220		no significant similarity				may not be a gene
scaffold_28	CsC	7350	25280-32629	overlap with Cs_IVSPER-2									
scaffold_17	CsE	7990	1330025-1338014	rep1_CsE	1331076-1331756 - strand	no	226	YP_00103133	repeat element protein-d11.1 [Hyposoter fugi	199	4,00E-06	42/175(24%)	
scaffold_17	CsE	7990	1330025-1338014	rep2_CsE	1333207-1333722 - strand	no	171	YP_00103133	repeat element protein-d11.1 [Hyposoter fugi	199	0.004	33/133(25%)	
scaffold_17	CsE	7990	1330025-1338014	rep3_CsE	1336256-1336924 - strand	no	222	AAA42923.1	repeat element protein [Campoletis sonorens	235	8,00E-53	87/197(44%)	
scaffold_131	CsF	8155	808380-816534	cys_CsF	812667-814550 - strand	yes	403	AY197491	FHv1.4 cys-motif protein precursor [Campole	403	0.0	403/403 (100%)	
scaffold_10	CsD	8168	961052-969219	vnx_CsD	963294-964382 - strand	no	362	AAO45828.1	innexin Vnx-d1 [Campoletis sonorensis ichno	362	0.0	361/362(99%)	
scaffold_10	CsD	8168	961052-969219	HP_CsD	964936-965238 - strand	no	100		no significant similarity				
scaffold_14	CsG2	8338	192247-200584	rep1_CsG2	192863-193606 + strand	no	247	BAF45598.1	c7-1.1 [Tranosema rostrale ichnovirus]	248	5,00E-48	98/237(41%)	
scaffold_14	CsG2	8338	192247-200584	rep2_CsG2	194788-195492 + strand	no	234	BAF45598.1	c7-1.1 [Tranosema rostrale ichnovirus]	248	2,00E-46	92/203(45%)	
scaffold_14	CsG2	8338	192247-200584	rep3_CsG2	196511-197119 + strand	no	202	BAF45598.1	c7-1.1 [Tranosema rostrale ichnovirus]	248	1,00E-59	95/215(44%)	
scaffold_14	CsG2	8338	192247-200584	rep4_CsG2	198753-199601 + strand	no	282	BAF45598.1	c7-1.1 [Tranosema rostrale ichnovirus]	248	1,00E-59	107/214(50%)	
scaffold_14	CsG	8656	76017-84672	vnx_CsG	80980-82059 - strand	no	359	AAO45829.1	innexin Vnx-g1 [Campoletis sonorensis ichno	359	0.0	359/359(100%)	
scaffold_14	CsG	8656	76017-84672	HP_CsG	83584-83949 + strand	no	121		no significant similarity				
scaffold_22	CsI	8779	695663-704441	rep1_CsI	696515-697228 + strand	no	237	AAA42923.1	repeat element protein [Campoletis sonorens	235	3,00E-54	91/197(46%)	
scaffold_22	CsI	8779	695663-704441	rep2_CsI	697713-698441 + strand	no	242	AAA42923.1	repeat element protein [Campoletis sonorens	235	2,00E-62	98/199(49%)	
scaffold_22	CsI	8779	695663-704441	HP_CsI	700059-700382 - strand	no	107		no significant similarity				
scaffold_22	CsI	8779	695663-704441	rep3_CsI	700879-701784 + strand	no	301	AAA42923.1	repeat element protein [Campoletis sonorens	235	3,00E-51	96/190(51%)	
scaffold_128	CsI2	9042	110016-119057	vank1_CsI2	110664-111179 - strand	no	171	AAX56959.1	vankyrin 3 [Campoletis sonorensis ichnovirus	171	3,00E-125	171/171(100%)	
scaffold_128	CsI2	9042	110016-119057	rep_CsI2	112670-113302 - strand	no	210	BAF45598.1	c7-1.1 [Tranosema rostrale ichnovirus]	248	8,00E-61	98/187(52%)	
scaffold_128	CsI2	9042	110016-119057	vank2_CsI2	114140-114607 - strand	no	155	AAX56957.1	vankyrin 1 [Campoletis sonorensis ichnovirus	155	2,00E-111	155/155(100%)	
scaffold_128	CsI2	9042	110016-119057	HP_CsI2	115736-116062 - strand	no	108		no significant similarity				
scaffold_128	CsI2	9042	110016-119057	vank3_CsI2	117111-117617 - strand	no	168	AAX56958.1	vankyrin 2 [Campoletis sonorensis ichnovirus	168	8,00E-121	168/168(100%)	
scaffold_38	CsH	9050	1398066-1407115	HP_CsH	1400102-1400407 - strand	no	101		no significant similarity				
scaffold_38	CsH	9050	1398066-1407115	5rep_CsH	1402633-1405449 + strand	no	938	AAA42923.1	repeat element protein [Campoletis sonorens	235	2,00E-41	89/215(41%)	
scaffold_16	CsX6	9213	504600-513812	2rep_CsX6	506770-507660 - strand	no	296	AAA42923.1	repeat element protein [Campoletis sonorens	235	8,00E-45	84/175(48%)	
scaffold_16	CsX6	9213	504600-513812	rep_CsX6	512155-512874 - strand	no	239	AAA42923.1	repeat element protein [Campoletis sonorens	235	8,00E-57	95/203(47%)	
scaffold_15	CsJ	9484	2621922-2631405	rep1_CsJ	2623490-2624194 + strand	no	234	BAF45598.1	c7-1.1 [Tranosema rostrale ichnovirus]	248	3,00E-59	98/216(45%)	
scaffold_15	CsJ	9484	2621922-2631405	rep2_CsJ	2625029-2625739 - strand	no	236	BAF45598.1	c7-1.1 [Tranosema rostrale ichnovirus]	248	6,00E-50	91/218(42%)	
scaffold_15	CsJ	9484	2621922-2631405	rep3_CsJ	2629669-2630322 + strand	no	217	BAF45598.1	c7-1.1 [Tranosema rostrale ichnovirus]	248	2,00E-37	80/187(43%)	
scaffold_35	CsX8	9999	164467-174465	HP1_CsX8	165205-165672 - strand	no	155	no significant similarity					
scaffold_35	CsX8	9999	164467-174465	cys_CsX8	168692-172561 - strand	yes	581	AAO43445.1	LHv2.8 cys-motif protein precursor [Campole	678	0.0	409/671(61%)	
scaffold_35	CsX8	9999	164467-174465	HP2_CsX8	172947-173360 - strand	no	137	no significant similarity					
scaffold_4391	CsL	10024	8079-18102	cys_CsL	10645-14521 - strand	yes	678	AAO43445.1	LHv2.8 cys-motif protein precursor [Campole	678	0.0	675/678(99%)	shorter
scaffold_4391	CsL	10024	8079-18102	HP_CsL	16908-17306 + strand	no	132		no significant similarity				
scaffold_110	CsX2	10806	248068-258873	vank1_CsX2	249108-249605 + strand	no	165	ABH10021.1	vankyrin 2 [Campoletis chlorideae ichnovirus]	168	2,00E-105	144/165(87%)	
scaffold_110	CsX2	10806	248068-258873	vank2_CsX2	251609-252118 + strand	no	169	AAX56956.1	vankyrin 4 [Campoletis sonorensis ichnovirus	160	2,00E-65	100/151(66%)	short
scaffold_110	CsX2	10806	248068-258873	vank3_CsX2	252614-253099 + strand	no	161	AAX56955.1	vankyrin 3 [Campoletis sonorensis ichnovirus	161	9,00E-81	122/149(82%)	
scaffold_110	CsX2	10806	248068-258873	vank4_CsX2	254294-254672 + strand	no	125	AAX56955.1	vankyrin 3 [Campoletis sonorensis ichnovirus	161	2,00E-23	62/147(42%)	
scaffold_110	CsX2	10806	248068-258873	rep_CsX2	256559-257191 + strand	no	210	BAF45598.1	c7-1.1 [Tranosema rostrale ichnovirus]	248	2,00E-60	94/189(50%)	
scaffold_5	CsN	10943	175169-186111	N1_CsN	177111-178547 - strand	no	478	AAS79017.1	NHv1.2 protein [Campoletis sonorensis ichno	400	0.0	305/404(75%)	
scaffold_5	CsN	10943	175169-186111	N2_CsN	180542-181744 - strand	no	400	AAS79017.1	NHv1.2 protein [Campoletis sonorensis ichno	400	0.0	400/400(100%)	
scaffold_5	CsN	10943	175169-186111	HP_CsN	185333-185656 + strand	no	107		no significant similarity				
scaffold_5218	CsP	12113	15720-27832	HP_CsP	19074-19379 - strand	no	101		no significant similarity				
scaffold_5218	CsP	12113	15720-27832	vank4_CsP	19628-20110 + strand	no	160	AAX56956.1	vankyrin 4 [Campoletis sonorensis ichnovirus	160	3,00E-114	159/160(99%)	
scaffold_5218	CsP	12113	15720-27832	vank3_CsP	20609-21094 + strand	no	161	AAX56955.1	vankyrin 3 [Campoletis sonorensis ichnovirus	161	4,00E-115	161/161(100%)	
scaffold_5218	CsP	12113	15720-27832	vank2_CsP	23733-24215 + strand	no	160	AAX56954.1	vankyrin 2 [Campoletis sonorensis ichnovirus	160	1,00E-114	160/160(100%)	
scaffold_5218	CsP	12113	15720-27832	vank1_CsP	26747-27262 + strand	no	171	AAX56953.1	vankyrin 1 [Campoletis sonorensis ichnovirus	171	3,00E-124	171/171(100%)	
scaffold_23	CsM	12197	1422227-1434423	HP1_CsM	1425348-1425740 - strand	no	130		no significant similarity				
scaffold_23	CsM	12197	1422227-1434423	N_CsM	1428224-1429615 + strand	no	463	AAS79017.1	NHv1.2 protein [Campoletis sonorensis ichno	400	3,00E-166	245/407(60%)	
scaffold_23	CsM	12197	1422227-1434423	HP2_CsM	1431734-1432099 - strand	no	121		no significant similarity				
scaffold_50	CsQ	12543	290527-303069	rep1_CsQ	291919-292629 - strand	no	236	AAA42923.1	repeat element protein [Campoletis sonorens	235	2,00E-73	116/231(50%)	
scaffold_50	CsQ	12543	290527-303069	vinx1_CsQ	294036-295118 - strand	no	360	YP_589076.1	innexin-like protein 1 [Campoletis sonorensis	369	0.0	323/328(98%)	
scaffold_50	CsQ	12543	290527-303069	vinx2_CsQ	296198-297295 + strand	no	365	YP_589075.1	innexin-like protein 2 [Campoletis sonorensis	365	0.0	365/365(100%)	
scaffold_50	CsQ	12543	290527-303069	rep2_CsQ	299186-299929 - strand	no	247	AAA42923.1	repeat element protein [Campoletis sonorens	235	9,00E-48	85/187(45%)	
scaffold_50	CsQ	12543	290527-303069	rep3_CsQ	300417-301001 - strand		194	AAA42923.1	repeat element protein [Campoletis sonorens	235	6,00E-45	83/191(43%)	
scaffold_50	CsQ	12543	290527-303069	rep4_CsQ	301383-302144 - strand	no	253	AAA42923.1	repeat element protein [Campoletis sonorens	235	2,00E-53	94/210(45%)	
scaffold_6070	CsO1	12746	168701-181446	4rep_CsO1	171805-173964 + strand	no	719	AAA42923.1	repeat element protein [Campoletis sonorens	235	3,00E-43	83/184(45%)	
scaffold_6070	CsO1	12746	168701-181446	HP1_CsO1	174818-175135 + strand	no	105		no significant similarity				
scaffold_6070	CsO1	12746	168701-181446	HP2_CsO1	176713-177060 + strand	no	115		no significant similarity				
scaffold_6070	CsO1	12746	168701-181446	3rep_CsO1	177630-179456 + strand	no	608	AAA42923.1	repeat element protein [Campoletis sonorens	235	5,00E-33	81/223(36%)	
scaffold_149	CsU	15338	374074-389411	cys1_CsU	375123-375900 - strand	yes	180	AAO43446.1	UHv0.8a cys-motif protein precursor [Campol	180	8,00E-131	180/180(100%)	Number of Matches: 5
scaffold_149	CsU	15338	374074-389411	cys2_CsU	377416-378140 - strand	yes	161	AAO43447.1	UHv0.8 cys-motif protein precursor [Campole	152	6,00E-54	91/151(60%)	

scaffold_149	CsU	15338	374074-389411	cys3_CsU	383382-384157 - strand	yes	178	AAO43446.1	UHv0.8a cys-motif protein precursor [Campol	180	1,00E-62	96/164(59%)	
scaffold_149	CsU	15338	374074-389411	cys4_CsU	385182-385875 - strand	yes	152	AAO43447.1	UHv0.8 cys-motif protein precursor [Campole	152	2,00E-108	152/152(100%)	
scaffold_149	CsU	15338	374074-389411	cys5_CsU	387355-388121 - strand	yes	175	AAO43447.1	UHv0.8 cys-motif protein precursor [Campole	152	2,00E-66	104/152(68%)	NNN within the nt sequence
scaffold_28	CsW	15807	614005-629811	cys1_CsW	615213-616060 + strand	yes	203	YP_589078.1	cysteine-rich protein [Campoletis sonorensis	203	3,00E-140	192/203(95%)	
scaffold_28	CsW	15807	614005-629811	cys2_CsW	617470-618890 + strand	yes	261	YP_589079.1	cysteine-rich protein [Campoletis sonorensis	198	8,00E-43	71/120(59%)	
scaffold_28	CsW	15807	614005-629811	rep1_CsW	619946-620629 - strand	no	227	BAF73402.1	f3.1 [Tranosema rostrale ichnovirus]	226	8,00E-73	107/222(48%)	
scaffold_28	CsW	15807	614005-629811	cys3_CsW	622282-623280 + strand	yes	198	YP_589079.1	cysteine-rich protein [Campoletis sonorensis	198	2,00E-145	198/198(100%)	
scaffold_28	CsW	15807	614005-629811	rep2_CsW	624629-625342 - strand	no	237	YP_0010312€	repeat element protein-b15.1 [Hyposoter fugi	272	5,00E-80	122/237(51%)	
scaffold_28	CsW	15807	614005-629811	HP_CsW	627004-627324 - strand	no	106		no significant similarity				
scaffold_28	CsW	15807	614005-629811	rep3_CsW	628358-629077 - strand	no	239	YP_00103131	repeat element protein-d4.2 [Hyposoter fugitiv	248	6,00E-98	132/226(58%)	
scaffold_5890	CsZ	15871	134147-150017	rep1_CsZ	134843-135562 + strand	no	239	AHY22033.1	repeat element 33 [Diadegma semiclausum i	243	5,00E-69	103/219(47%)	
scaffold_5890	CsZ	15871	134147-150017	rep2_CsZ	137736-138422 + strand	no	228	AIK25648.1	Rep1 [Hyposoter didymator ichnovirus]	231	9,00E-72	105/213(49%)	
scaffold_5890	CsZ	15871	134147-150017	rep3_CsZ	139610-140290 + strand	no	226	BAF45626.1	f3.2 [Tranosema rostrale ichnovirus]	237	3,00E-62	93/189(49%)	
scaffold_5890	CsZ	15871	134147-150017	rep4_CsZ	140771-141505 + strand	no	244	AHY21950.1	repeat element 11 [Diadegma semiclausum i	225	3,00E-55	87/202(43%)	
scaffold_5890	CsZ	15871	134147-150017	rep5_CsZ	142565-143128 + strand	no	187	YP_00103131	repeat element protein-d3.2 [Hyposoter fugitiv	230	4,00E-51	87/178(49%)	
scaffold_5890	CsZ	15871	134147-150017	rep6_CsZ	144223-144912 + strand	no	229	BAF45611.1	d5.2 [Tranosema rostrale ichnovirus]	218	4,00E-72	107/206(52%)	
scaffold_5890	CsZ	15871	134147-150017	rep7_CsZ	146321-147118 + strand	no	265	BAF45598.1	c7-1.1 [Tranosema rostrale ichnovirus]	248	3,00E-106	153/249(61%)	
scaffold_5934	CsX1	17335	19391-36725	vank1_CsX1	20620-21141 + strand	no	173	AFH35119.1	vankyrin 5 [Hyposoter didymator ichnovirus]	168	7,00E-43	75/164(46%)	
scaffold_5934	CsX1	17335	19391-36725	vnx1_CsX1	22756-23850 + strand	no	364	AHY21960.1	viral innexin 3 [Diadegma semiclausum ichno	357	7,00E-131	182/351(52%)	
scaffold_5934	CsX1	17335	19391-36725	rep1_CsX1	24562-25275 + strand	no	237	BAF45598.1	c7-1.1 [Tranosema rostrale ichnovirus]	248	6,00E-65	108/216(50%)	NNN within the nt sequence
scaffold_5934	CsX1	17335	19391-36725	vank2_CsX1	26300-26770 + strand	no	156	AFH35115.1	vankyrin 1 [Hyposoter didymator ichnovirus]	159	3,00E-52	82/152(54%)	Number of Matches: 4
scaffold_5934	CsX1	17335	19391-36725	vank3_CsX1	28742-29212 + strand	no	156	YP_00103123	vankyrin-b17 [Hyposoter fugitivus ichnovirus]	170	1,00E-46	78/156(50%)	Number of Matches: 3
scaffold_5934	CsX1	17335	19391-36725	vank4_CsX1	30120-30608 + strand	no	162	YP_00103123	vankyrin-b1 [Hyposoter fugitivus ichnovirus]	167	8,00E-55	88/162(54%)	
scaffold_5934	CsX1	17335	19391-36725	vnx2_CsX1	31535-32626 + strand	no	363	BAF45609.1	d4.1 [Tranosema rostrale ichnovirus]	376	3,00E-141	198/360(55%)	
scaffold_5934	CsX1	17335	19391-36725	rep2_CsX1	34635-35156 + strand	no	173	BAF45626.1	f3.2 [Tranosema rostrale ichnovirus]	237	3,00E-55	93/180(52%)	
scaffold_5934	CsX1	17335	19391-36725	vank5_CsX1	35714-36208 + strand	no	164	ABH10021.1	vankyrin 2 [Campoletis chlorideae ichnovirus]	168	6,00E-50	80/163(49%)	
scaffold_116	CsT	23217	7789-31005	HP1_CsT	14790-15116 - strand	no	108		no significant similarity				
scaffold_116	CsT	23217	7789-31005	HP2_CsT	21883-22209 - strand	no	108		no significant similarity				
scaffold_116	CsT	23217	7789-31005	HP3_CsT	27023-27376 + strand	no	117		no significant similarity				
scaffold_116	CsT	23217	7789-31005	HP4_CsT	28660-29043 + strand	no	127		no significant similarity				
scaffold_8362	CsX5, partial	>5297	1-5297	rep1_CsX5	2289-3026 - strand	no	245	AAA42923.1	repeat element protein [Campoletis sonorens	235	3,00E-50	95/240(40%)	
scaffold_8362	CsX5, partial	>5297	1-5297	rep2_CsX5	3571-4296 + strand	no	241	AAA42923.1	repeat element protein [Campoletis sonorens	235	5,00E-57	104/233(45%)	
scaffold_6095	CsX7, partial	>6041	183042-189082	rep1_CsX7	183042-183731 - strand	no	229	BAF45626.1	f3.2 [Tranosema rostrale ichnovirus]	237	2,00E-76	112/202(55%)	
scaffold_6095	CsX7, partial	>6041	183042-189082	rep2_CsX7	185566-186273 - strand	no	235	BAF45626.1	f3.2 [Tranosema rostrale ichnovirus]	237	5,00E-72	106/201(53%)	
scaffold_6095	CsX7, partial	>6041	183042-189082	rep3_CsX7	188342-189082 - strand	no	246	BAF45626.1	f3.2 [Tranosema rostrale ichnovirus]	237	2,00E-74	112/201(56%)	
scaffold_60	CsX3, partial	>7876	304794-312669	rep1_CsX3	304794-305507 + strand	no	237	AHY22033.1	repeat element 33 [Diadegma semiclausum i	243	1,00E-77	115/225(51%)	
scaffold_60	CsX3, partial	>7876	304794-312669	rep2_CsX3	306761-307570 + strand	no	269	BAF45598.1	c7-1.1 [Tranosema rostrale ichnovirus]	248	2,00E-119	165/247(67%)	Number of Matches: 2, NNN in 5'region
scaffold_60	CsX3, partial	>7876	304794-312669	rep3_CsX3	309164-309979 + strand	no	271	BAF45598.1	c7-1.1 [Tranosema rostrale ichnovirus]	248	2,00E-115	162/247(66%)	
scaffold_60	CsX3, partial	>7876	304794-312669	rep4_CsX3	311747-312586 + strand	no	279	BAF45598.1	c7-1.1 [Tranosema rostrale ichnovirus]	248	8,00E-116	158/237(67%)	
scaffold_13	CsX4, partial	>9181	1407384-1416564	rep1_CsX4	1409287-1410012 - strand	no	241	AAA42923.1	repeat element protein [Campoletis sonorens	235	5,00E-57	104/233(45%)	
scaffold_13	CsX4, partial	>9181	1407384-1416564	rep2_CsX4	1410557-1411294 + strand	no	245	AAA42923.1	repeat element protein [Campoletis sonorens	235	3,00E-50	95/240(40%)	
scaffold_13	CsX4, partial	>9181	1407384-1416564	HP_CsX4	1411909-1412361 + strand	no	150	no significant similarity					
scaffold_13	CsX4, partial	>9181	1407384-1416564	rep3_CsX4	1415602-1416564 + strand	no	320	AAA42923.1	repeat element protein [Campoletis sonorens	235	6,00E-51	96/232(41%)	
***C. sonorensis* IVSPER**													
scaffold_6122	IVSP_U36L	471	234803-235273	U36L	234803-235273 - strand	no	156	AKD28080.1	hypothetical protein [Glypta fumiferanae] Gf_	175	7,00E-15	49/146(34%)	
scaffold_12	IVSP_U37L	1863	104145-106007	U37L-2	104145-106007 - strand	no	620	AKD28048.1	helicase-primase domain [Glypta fumiferanae	721	0.0	337/621(54%)	
scaffold_16	Cs_IVSPER-5	3750	2424942-2428691	IVSP1L-3	2424942-2425688 + strand	no	248	ADI40453.1	unknown [Hyposoter didymator] Hd_IVSP1-1	253	6,00E-57	104/257(40%)	
scaffold_16	Cs_IVSPER-5	3750	2424942-2428691	U2L	2426081-2426764 + strand	no	227	ADI40454.1	unknown [Hyposoter didymator] Hd_U2	228	3,00E-120	165/226(73%)	
scaffold_16	Cs_IVSPER-5	3750	2424942-2428691	U1L	2427951-2428691 - strand	no	246	ADI40452.1	unknown [Hyposoter didymator] Hd_U1	244	1,00E-65	101/245(41%)	
scaffold_50	Cs_IVSPER-3	8610	218627-227236	IVSP4L-2	218627-219940 - strand	no	437	ADI40475.1	unknown [Hyposoter didymator] Hd_IVSP4-1	446	7,00E-156	217/399(54%)	
scaffold_50	Cs_IVSPER-3	8610	218627-227236	U4L	220560-221022 + strand	no	138	ADI40456.1	unknown [Hyposoter didymator] Hd_U4	131	2,00E-29	52/118(44%)	
scaffold_50	Cs_IVSPER-3	8610	218627-227236	p53L-3	221232-222284 + strand	no	350	CAR31590.1	p53-like1 protein [Hyposoter didymator]	395	6,00E-67	139/337(41%)	
scaffold_50	Cs_IVSPER-3	8610	218627-227236	U5L	222667-223197 + strand	no	176	ADI40458.1	unknown [Hyposoter didymator] Hd_U5	176	1,00E-74	105/174(60%)	
scaffold_50	Cs_IVSPER-3	8610	218627-227236	IVSP2L-2	223589-224965 + strand	no	458	ADI40459.1	unknown [Hyposoter didymator] Hd_IVSP2-1	509	0.0	303/459(66%)	
scaffold_50	Cs_IVSPER-3	8610	218627-227236	CsN-3	225761-227236 + strand	no	491	ADI40491.1	unknown [Hyposoter didymator] Hd_N-2	492	0.0	346/492(70%)	
scaffold_57	Cs_IVSPER-4	9937	383305-393241	U30L	383305-386526 + strand	no	1073	AKD28060.1	hypothetical protein [Glypta fumiferanae] Gf_	1322	0.0	502/1044(48%)	
scaffold_57	Cs_IVSPER-4	9937	383305-393241	U34L	387873-390950 + strand	no	1025	AKD28054.1	helicase domain [Glypta fumiferanae] Hd_U3	1012	0.0	486/1009(48%)	
scaffold_57	Cs_IVSPER-4	9937	383305-393241	IVSP4L-3	391820-393241 - strand	no	473	ADI40484.1	unknown [Hyposoter didymator] Hd_IVSP4-2	431	2,00E-169	230/405(57%)	
scaffold_6122	Cs_IVSPER-1	31594	122689-154282	U15L	122689-123342 - strand	no	217	ADI40477.1	unknown [Hyposoter didymator] Hd_U15	410	3,00E-97	130/212(61%)	
scaffold_6122	Cs_IVSPER-1	31594	122689-154282	IVSP1L-1	124770-125432 + strand	no	220	ADI40464.1	unknown [Hyposoter didymator] Hd_IVSP1-2	196	6,00E-63	99/197(50%)	
scaffold_6122	Cs_IVSPER-1	31594	122689-154282	U37L-1	126353-128176 + strand	no	607	AKD28048.1	helicase-primase domain [Glypta fumiferanae	721	0.0	338/624(54%)	
scaffold_6122	Cs_IVSPER-1	31594	122689-154282	U31L-1	129117-130226 - strand	no	369	AKD28063.1	hypothetical protein [Glypta fumiferanae] Gf_	370	4,00E-32	95/341(28%)	
scaffold_6122	Cs_IVSPER-1	31594	122689-154282	U35L	130724-131257 - strand	no	177	AKD28058.1	hypothetical protein [Glypta fumiferanae] Gf_	180	5,00E-37	70/156(45%)	

scaffold_6122	Cs_IVSPER-1	31594	122689-154282	Gf_U27L	132692-134518 + strand	no	608	AKD28059.1	hypothetical protein [Glypta fumiferanae] Gf_	325	1,00E-31	97/312(31%)	
scaffold_6122	Cs_IVSPER-1	31594	122689-154282	U17L	135604-135929 + strand	no	79	ADI40480.1	unknown [Hyposoter didymator] Hd_U17	82	1,00E-14	31/66(47%)	
scaffold_6122	Cs_IVSPER-1	31594	122689-154282	p12L-1	137295-137555 + strand	no	86	AAD01200.1	p12 [Campoletis sonorensis ichnovirus]	92	8,00E-17	43/92(47%)	
scaffold_6122	Cs_IVSPER-1	31594	122689-154282	U19L	139263-141209 + strand	no	648	ADI40483.1	unknown [Hyposoter didymator] Hd_U19	660	0.0	480/649(74%)	
scaffold_6122	Cs_IVSPER-1	31594	122689-154282	IVSP4L-1	141938-143242 - strand	no	434	ADI40484.1	unknown [Hyposoter didymator] Hd_IVSP4-2	431	5,00E-175	232/434(53%)	
scaffold_6122	Cs_IVSPER-1	31594	122689-154282	U22L	143868-144761 - strand	no	297	ADI40487.1	unknown [Hyposoter didymator] Hd_U22	257	4,00E-59	95/249(38%)	
scaffold_6122	Cs_IVSPER-1	31594	122689-154282	U23L	146427-147608 - strand	no	393	ADI40488.1	unknown [Hyposoter didymator] Hd_U23	415	2,00E-170	245/389(63%)	
scaffold_6122	Cs_IVSPER-1	31594	122689-154282	p53L-1	148451-149548 + strand	no	365	AAD01199.1	p53 [Campoletis sonorensis ichnovirus]	364	0.0	306/365(84%)	
scaffold_6122	Cs_IVSPER-1	31594	122689-154282	U24L	150683-152365 - strand	no	560	ADI40490.1	unknown [Hyposoter didymator] Hd_U24	563	0.0	387/558(69%)	
scaffold_6122	Cs_IVSPER-1	31594	122689-154282	CsN-1	152861-154282 + strand	no	473	ADI40491.1	unknown [Hyposoter didymator] Hd_N-2	492	0.0	307/491(63%)	
scaffold_28	Cs_IVSPER-2	33269	7310-40578	U6L	10522-11574 + strand	no	350	ADI40462.1	unknown [Hyposoter didymator] Hd_U6	349	0.0	297/350(85%)	
scaffold_28	Cs_IVSPER-2	33269	7310-40578	U7L	13049-13684 + strand	no	211	ADI40463.1	unknown [Hyposoter didymator] Hd_U7	212	5,00E-61	119/211(56%)	
scaffold_28	Cs_IVSPER-2	33269	7310-40578	IVSP1L-2	13781-14701 + strand	no	306	ADI40464	unknown [Hyposoter didymator] Hd_IVSP1-2	196	9,00E-58	93/195(48%)	
scaffold_28	Cs_IVSPER-2	33269	7310-40578	IVSP3L	15113-17002 + strand	no	629	ADI40465.1	unknown [Hyposoter didymator] Hd_IVSP3-1	630	0.0	423/628(67%)	
scaffold_28	Cs_IVSPER-2	33269	7310-40578	U31L-2	17484-18179 - strand	no	231	AKD28063.1	hypothetical protein [Glypta fumiferanae] Gf_	370	0.029	40/136(29%)	
scaffold_28	Cs_IVSPER-2	33269	7310-40578	U8L	18541-18768 - strand	no	75	ADI40466.1	unknown [Hyposoter didymator] Hd_U8	77	4,00E-14	36/75(48%)	
scaffold_28	Cs_IVSPER-2	33269	7310-40578	U9L	19075-19890 - strand	no	271	ADI40467.1	unknown [Hyposoter didymator] Hd_U9	274	2,00E-112	170/269(63%)	
scaffold_28	Cs_IVSPER-2	33269	7310-40578	U16L	20258-22096 - strand	no	612	ADI40479.1	unknown [Hyposoter didymator] Hd_U16	612	0.0	491/605(81%)	
scaffold_28	Cs_IVSPER-2	33269	7310-40578	U10L	22789-27768 + strand	no	1344	ADI40468.1	unknown [Hyposoter didymator] Hd_U10	1355	0.0	1035/1353(76%)	partial?
scaffold_28	Cs_IVSPER-2	33269	7310-40578	U11L	27768-28709 + strand	no	313	ADI40469.1	unknown [Hyposoter didymator] Hd_U11	316	3,00E-180	244/317(77%)	
scaffold_28	Cs_IVSPER-2	33269	7310-40578	U12L	29360-29971 - strand	no	203	ADI40470.1	unknown [Hyposoter didymator] Hd_U12	203	1,00E-121	168/203(83%)	
scaffold_28	Cs_IVSPER-2	33269	7310-40578	U13L	30286-30768 + strand	no	160	ADI40471.1	unknown [Hyposoter didymator] Hd_U13	152	5,00E-29	66/160(41%)	
scaffold_28	Cs_IVSPER-2	33269	7310-40578	p12L-2	31075-31353 + strand	no	92	AAD01200.1	p12 [Campoletis sonorensis ichnovirus]	92	5,00E-59	92/92(100%)	
scaffold_28	Cs_IVSPER-2	33269	7310-40578	U3L	32951-33436 + strand	no	161	ADI40455.1	unknown [Hyposoter didymator] Hd_U3	162	3,00E-67	93/155(60%)	frameshifts, partial?
scaffold_28	Cs_IVSPER-2	33269	7310-40578	IVSP2L-1	34504-36036 + strand	no	510	ADI40459.1	unknown [Hyposoter didymator] Hd_IVSP2	509	0.0	325/510(64%)	
scaffold_28	Cs_IVSPER-2	33269	7310-40578	U26L	36497-37327 + strand	no	276	AKD28081.1	hypothetical protein [Glypta fumiferanae] Gf_	298	3,00E-29	66/165(40%)	
scaffold_28	Cs_IVSPER-2	33269	7310-40578	CsN-2	37470-38912 + strand	no	480	ADI40491.1	unknown [Hyposoter didymator] Hd_N-2	492	0.0	323/491(66%)	
scaffold_28	Cs_IVSPER-2	33269	7310-40578	U25L	39883-40578 - strand	no	231	AKD28083.1	ring finger domain [Glypta fumiferanae] Hd_U	237	2,00E-16	41/128(32%)	
scaffold_28	Cs_IVSPER-2	33269	7310-40578	p53L-2	7310-8944 + strand	no	544	CAR31590.1	p53-like1 protein [Hyposoter didymator]	395	6,00E-57	155/247(62%)	
