# Supplementary material for: Genomic architecture of endogenous ichnoviruses reveals distinct evolutionary pathways leading to virus domestication in parasitic wasps
Source: BMC Biol. 2020 Jul 24;18:89. doi: 10.1186/s12915-020-00822-3 (PMC7379367; doi:10.1186/s12915-020-00822-3)

**Additional file 6. Figure S1.** *H. didymator* proviral loci corresponding to two segments previously described as "distinct" but sharing part of their sequence (Dorémus et al., 2014). Segments Hd2a (GenBank: KJ586332.1) and Hd2b (GenBank: KJ586327.1) co-localize in the same genome locus here named Hd2; segments Hd11a (KJ586322.1) and Hd11b (KJ586302.1) co-localize in the same genome locus here named Hd11; Hd17a (KJ586314.1) and Hd17b (KJ586316.1) co-localize in the same genome locus here named Hd17; Hd20a (KJ586312.1) and Hd20b (KJ586297.1) co-localize in the same genome locus here named Hd20; Hd26a (KJ586301.1) and Hd26b (KJ586306.1) co-localize in the same genome locus here named Hd26; and finally, Hd31 (KJ586299.1) and Hd34 (KJ586295.1) co-localize in the same genome locus here named Hd31-34. Each proviral locus was characterized by the presence of two different direct repeated sequences (DRJ1 and DRJ2) at the extremities of each of the overlapping segments. Scale bar: 1000 nt.

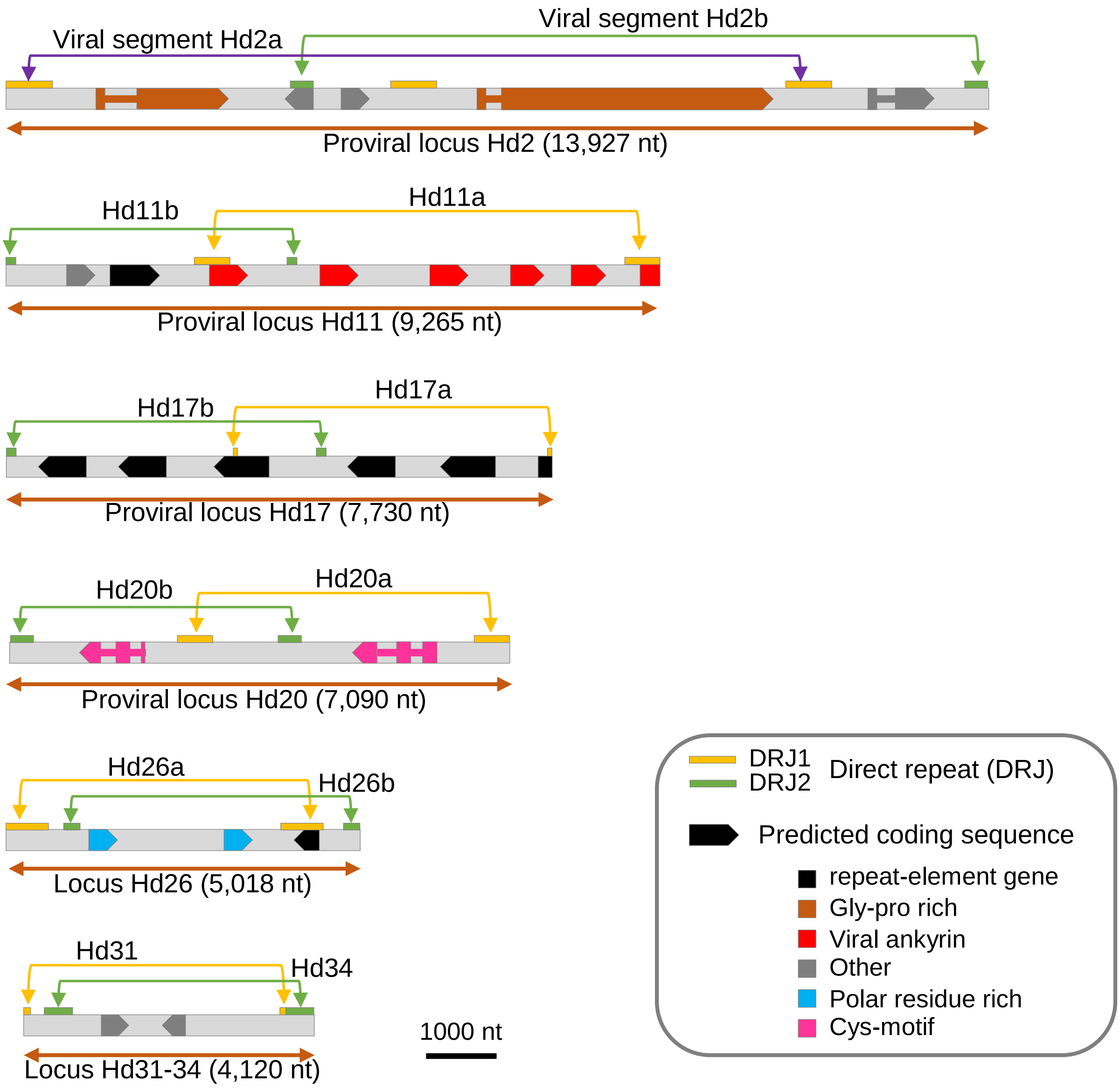

Supplement: Supplementary file 6 — Additional file 6: Figure S1. H. didymator proviral loci corresponding to two segments previously described as “distinct” but sharing part of their sequence [36]. Segments Hd2a (GenBank: KJ586332.1) and Hd2b (GenBank: KJ586327.1) co-localize in the same genome locus here named Hd2; segments Hd11a (KJ586322.1) and Hd11b (KJ586302.1) co-localize in the same genome locus here named Hd11; Hd17a (KJ586314.1) and Hd17b (KJ586316.1) co-localize in the same genome locus here named Hd17; Hd20a (KJ586312.1) and Hd20b (KJ586297.1) co-localize in the same genome locus here named Hd20; Hd26a (KJ586301.1) and Hd26b (KJ586306.1) co-localize in the same genome locus here named Hd26; and finally, Hd31 (KJ586299.1) and Hd34 (KJ586295.1) co-localize in the same genome locus here named Hd31-34. Each proviral locus was characterized by the presence of two different direct repeated sequences (DRJ1 and DRJ2) at the extremities of each of the overlapping segments. Scale bar: 1000 nt. [file 12915_2020_822_MOESM6_ESM.pdf]
