# Supplementary material for: Genomic architecture of endogenous ichnoviruses reveals distinct evolutionary pathways leading to virus domestication in parasitic wasps
Source: BMC Biol. 2020 Jul 24;18:89. doi: 10.1186/s12915-020-00822-3 (PMC7379367; doi:10.1186/s12915-020-00822-3)

Additional file 7. Dispersion of the viral loci within ichneumonid genomes.

Table S8. Distance (in bp) between two segments, a segment and an IVSPER or between two IVSPERs localized in the same scaffold.

Scaffold ID	Segment/IVSPER ID	Position in scaffold	Distance (bp) between segments	Distance (bp) between segment/IVSPER	Distance (bp) between IVSPERs
Hyposoter didymator					
scaffold29771	IVSP_U37	16848-18686			
scaffold29771	Hd46	19344-23452		659	
scaffold128213	Hd23.1	208205-212661			
scaffold128213	Hd23.2	248624-251985	35,964		
scaffold91	Hd_IVSPER-1	453857-467876			
scaffold91	Hd15	469105-474091		1,230	
scaffold91	Hd33	485063-488897	10,972		
scaffold91	Hd24	535698-540394	46,802		
scaffold91	Hd_IVSPER-2	541304-567914		911	
scaffold91	Hd29	572514-576869		4,601	
scaffold65	Hd37	342293-346000			
scaffold65	Hd3	437082-447095	91,083		
scaffold64	Hd14	36336-41531			
scaffold64	Hd32	88702-96617	47,172		
scaffold64	Hd42	2320523-2323679	2,223,907		
scaffold64	Hd21	2353107-2357474	29,429		
scaffold377	Hd8	2186417-2193772			
scaffold377	Hd4	2459681-2470006	265,91		
scaffold59	Hd12	674917-680818			
scaffold59	Hd16	690205-697908	9,388		
scaffold59	Hd11	2183512-2192701	1,485,605		
scaffold59	Hd10	2500377-2506883	307,677		
scaffold128243	Hd44.2	4197203-4202033			
scaffold128243	Hd44.1	4203985-4206993	1,953		
scaffold351	Hd17	2329273-2337002			
scaffold351	Hd18	2681961-2686656	344,96		
scaffold184	Hd45.2	3564791-3566841			
scaffold184	Hd41	3768924-3776876	202,084		
scaffold175	Hd36	3796140-3799877			
scaffold175	Hd38	3800393-3804056	517		
scaffold175	Hd26	10942034-10947051	7,137,979		
scaffold127548	Hd6	5808388-5818848			
scaffold127548	Hd2	5940296-5954232	121,449		
scaffold127548	Hd7	6062921-6070986	108,690		
scaffold127548	Hd_IVSPER-4	6832835-6848646		761,850	
scaffold127548	Hd_IVSPER-3	10761570-10787001			3,912,925
scaffold127548	Hd_IVSPER-5	10860001-10861630			73,001
scaffold127548	Hd47	12134587-12139089		1,272,958	
scaffold127548	Hd5	12941247-12954959	802,159		
scaffold127548	Hd27	13338255-13342256	383,297		
Campoletis sonorensis					
scaffold_50	Cs_IVSPER-3	218627-227236			
scaffold_50	CsQ	290527-303069		63,292	
scaffold_6122	Cs_IVSPER-1	122689-154282			
scaffold_6122	IVSP_U36L	234803-235273		80,522	
scaffold_14	CsG	76017-84672			

scaffold_14	CsG2	192247-200584	107,576	
scaffold_28	Cs_IVSPER-2	7310-40578		
scaffold_28	CsC	25280-32629		65,859
scaffold_28	CsW	614005-629811	581,377	
scaffold_16	CsX6	504600-513812		
scaffold_16	Cs_IVSPER-5	2424942-2428691		1,911,131

Figure S2. Graphical representation of the mean distance (in Kbp) between viral loci in *H. didymator* and *C. sonorensis* genomes. Data are given between 2 segments, between a segment and an IVSPER, and/or between 2 IVSPERs.

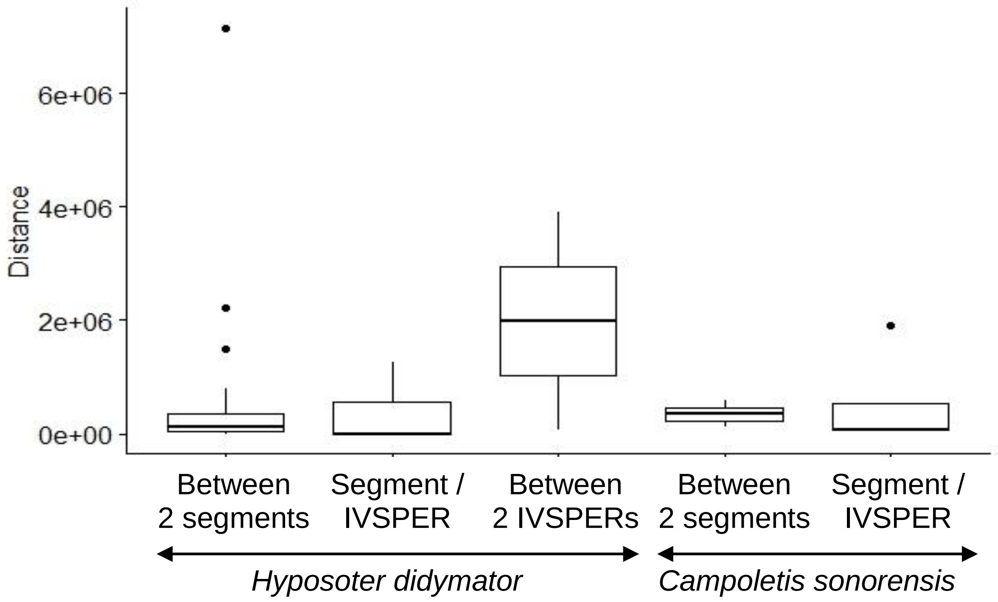

Supplement: Supplementary file 7 — Additional file 7: Dispersion of the viral loci within ichneumonid genomes. Table S8. Distance (in bp) between two segments, a segment and an IVSPER or between two IVSPERs localized in the same scaffold. Figure S2. Graphical representation of the mean distance (in Kbp) between viral loci in H. didymator and C. sonorensis genomes. Data are given between 2 segments, between a segment and an IVSPER, and/or between 2 IVSPERs. [file 12915_2020_822_MOESM7_ESM.pdf]
