# Supplementary material for: Genomic architecture of endogenous ichnoviruses reveals distinct evolutionary pathways leading to virus domestication in parasitic wasps
Source: BMC Biol. 2020 Jul 24;18:89. doi: 10.1186/s12915-020-00822-3 (PMC7379367; doi:10.1186/s12915-020-00822-3)
Supplement: Supplementary file 8 — Additional file 8: Table S9. Transposable elements (TE) found in Hyposter didymator segments, IVSPERs and neighboring regions. The LOLA package [86] was used to assess if some particular TE were enriched close to viral circles or IVSPER. Genomics positions were enlarged to 10 kbp at each segments ends and sampled against 1000 other similar regions from the genome, then used it a random reference. LOLA identifies overlaps and calculates enrichment for each TE. For each pairwise comparison, a series of columns describe the results of the statistical test (pvalueLog: -log10(pvalue) from the fisher’s exact result; oddsRatio: result from the fisher’s exact test; q-value transformation to provide false discovery rate (FDR) scores automatically). Some TE are enriched around viral locations, but after FDR correction, nothing was significant. [file 12915_2020_822_MOESM8_ESM.pdf]

TE family	pValueLog	oddsRatio	qValue
RSX-incomp_MCL345_Hd-B-P240.102-Map4	3.0918611809	17.7821472342	0.3349924897
noCat_MCL24_Hd-B-R551-Map20	2.8996118151	4.7330642255	0.3349924897
RIX-comp_MCL591_Hd-B-R5563-Map3_reversed	2.8512836938	39.1567664587	0.3349924897
noCat_MCL6_Hd-B-R206-Map20	2.7428462035	6.23463961	0.3349924897
RXX-TRIM_MCL42_Hd-B-G1267-Map5	2.7210184405	33.4299844597	0.3349924897
RLX-incomp_MCL488_Hd-B-R4280-Map3_reversed	2.6946367599	32.3691115766	0.3349924897
DTX-incomp_MCL29_Hd-B-R2332-Map20	2.4301167503	23.5485060582	0.5279688384
noCat_MCL674_Hd-B-R817-Map20	2.3493809132	21.361553524	0.5563552372
noCat_MCL32_Hd-B-P0.268-Map20	2.2436635223	3.5674867377	0.630836842
DMX-incomp_MCL506_Hd-B-R4459-Map4	2.1531036649	16.833546992	0.699389996
noCat_MCL1_Hd-B-R3553-Map20	2.1112339162	5.4812696426	0.7001586835
RXX-TRIM_MCL286_Hd-B-G414-Map4	2.0474614404	126.9993162993	0.7433278323
DTX-incomp_MCL26_Hd-B-P123.194-Map8	1.9685388055	13.4457664706	0.8117307217
RXX-TRIM_MCL276_Hd-B-G345-Map4	1.942283062	3.9690366006	0.8117307217
noCat_MCL370_Hd-B-R1303-Map20	1.9072778818	12.4661076982	0.821209453
RIX-incomp_MCL588_Hd-B-R5512-Map3	1.8271666032	2.724421063	0.9258411828
RLX-incomp_MCL663_Hd-B-R7854-Map3_reversed	1.6685508923	9.2725494301	1
RXX-TRIM_MCL72_Hd-B-G1239-Map12	1.6543524573	5.270349224	1
noCat_MCL62_Hd-B-G863-Map20	1.5630543946	4.8496542888	1
RIX-incomp_MCL101_Hd-B-G2185-Map3_reversed	1.5150174868	33.879581236	1
RXX-TRIM_MCL117_Hd-B-G570-Map4	1.5150174868	33.879581236	1
RXX-TRIM_MCL24_Hd-B-G1465-Map5	1.3754542738	24.213201794	1
RXX-TRIM_MCL42_Hd-B-G1488-Map4	1.3656818056	23.649915417	1
RIX-incomp_MCL374_Hd-B-R1487-Map3	1.2967139065	3.7766989824	1
RIX-incomp_MCL402_Hd-B-R2054-Map3	1.2764334714	2.2091797975	1
DXX-MITE_MCL158_Hd-B-P88.216-Map6	1.2753500619	5.6025026049	1
RXX-TRIM_MCL252_Hd-B-G2042-Map5	1.2609500026	5.4979815512	1
noCat_MCL51_Hd-B-G1882-Map20	1.2205488211	16.6762866175	1
noCat_MCL214_Hd-B-G1561-Map20	1.2006604748	15.8928215465	1
RIX-comp_MCL538_Hd-B-R4997-Map9	1.1879210812	15.4042477348	1
RXX_MCL495_Hd-B-R4392-Map3	1.1879210812	15.4042477348	1
noCat_MCL280_Hd-B-G363-Map11	1.1816979489	15.1742302343	1
noCat_MCL39_Hd-B-G539-Map17	1.1462403296	13.9282132115	1
DTX-incomp_MCL577_Hd-B-R5370-Map3	1.141547324	3.2421357768	1
RIX-comp_MCL383_Hd-B-R1664-Map4	1.1350799278	13.5578466416	1
DTX-incomp_MCL230_Hd-B-G1755-Map4	1.103343304	12.5477356701	1
DXX-MITE_MCL108_Hd-B-G829-Map6	1.0279382182	2.4282562686	1
noCat_MCL24_Hd-B-R2328-Map14	1.0277971244	2.8868187124	1
DHX-comp_MCL1_Hd-B-R539-Map20_reversed	1.0259762345	2.881362401	1
DXX-MITE_MCL122_Hd-B-R2760-Map15	1.0250959422	10.3704731009	1
DXX-MITE_MCL74_Hd-B-R215-Map14	1.0087334898	9.9587101772	1
DTX-incomp_MCL361_Hd-B-P76.225-Map8	0.9705889493	9.0725947733	1
noCat_MCL16_Hd-B-R1527-Map20	0.9475342594	2.6527528238	1
RXX-TRIM_MCL188_Hd-B-G1290-Map3	0.9455850846	1.7435664586	1
noCat_MCL13_Hd-B-G1765-Map20	0.9358557215	8.3256437109	1
noCat_MCL6_Hd-B-R237-Map20	0.929967673	2.2284565677	1
DXX-MITE_MCL25_Hd-B-G1524-Map9	0.9216897078	3.4561597075	1
RYX-comp_MCL688_Hd-B-R9118-Map3	0.9164256318	7.9345346081	1
RXX-TRIM_MCL73_Hd-B-G2038-Map8	0.8937733659	3.3204097	1
RLX-incomp_MCL31_Hd-B-R547-Map6_reversed	0.8868566945	1.6341481554	1
noCat_MCL7_Hd-B-R2178-Map20	0.8511231227	3.1209573276	1
DXX-MITE_MCL352_Hd-B-P313.38-Map3	0.8473302785	6.6785777778	1
DXX-MITE_MCL32_Hd-B-R139-Map20	0.846925586	3.101831095	1
RXX-TRIM_MCL234_Hd-B-G181-Map3	0.8344052643	6.4654600637	1
DTX-incomp_MCL236_Hd-B-G1825-Map6	0.8219145777	6.2652977175	1

noCat_MCL41_Hd-B-R236-Map20	0.8208340445	1.7446647698	1
DTX-incomp_MCL25_Hd-B-G2324-Map6	0.8194662029	6.2267382269	1
DTX-incomp_MCL153_Hd-B-G2301-Map3	0.8170339431	6.1886488699	1
RXX-TRIM_MCL603_Hd-B-R5997-Map7	0.8170339431	6.1886488699	1
DTX-incomp_MCL584_Hd-B-R548-Map13	0.8130360085	2.0013230859	1
RXX-TRIM_MCL7_Hd-B-G1097-Map8	0.7922308672	2.8605725867	1
DXX-MITE_MCL70_Hd-B-P337.14-Map3	0.7663758131	2.7513763818	1
RLX-incomp_MCL54_Hd-B-R5062-Map20	0.7663758131	2.7513763818	1
RSX-incomp_MCL319_Hd-B-G733-Map3	0.763034416	5.3971063058	1
RXX-TRIM_MCL28_Hd-B-R7888-Map3	0.7588768696	5.3401248076	1
RXX-TRIM_MCL655_Hd-B-R7621-Map3	0.7568159678	5.3120798255	1
DTX-incomp_MCL160_Hd-B-R228-Map20	0.7535795485	1.8901977721	1
DXX-MITE_MCL155_Hd-B-P127.190-Map3	0.7387823134	5.0720909652	1
DYX-comp_MCL658_Hd-B-R7678-Map3_reversed	0.7253607958	4.8999404933	1
RXX-TRIM_MCL249_Hd-B-G2026-Map3	0.7234842518	4.8749826179	1
noCat_MCL8_Hd-B-G2371-Map13	0.7070302453	4.6725752544	1
RXX-TRIM_MCL452_Hd-B-R3514-Map4	0.7052487061	4.6510751114	1
noCat_MCL6_Hd-B-R194-Map20	0.6964747246	4.5464170259	1
RXX-TRIM_MCL481_Hd-B-R395-Map3	0.6896112481	4.466550903	1
RLX-incomp_MCL23_Hd-B-R962-Map20_reversed	0.6719703402	2.3778316076	1
RIX-incomp_MCL622_Hd-B-R636-Map6	0.6714097285	4.2587377697	1
RLX-incomp_MCL68_Hd-B-R4393-Map20	0.6682013519	4.223076421	1
DXX-MITE_MCL357_Hd-B-P48.242-Map20	0.6650227558	4.1880116123	1
DTX-incomp_MCL478_Hd-B-R3763-Map7	0.6525965454	4.0533816299	1
DXX-MITE_MCL58_Hd-B-R511-Map20	0.6510747343	4.0370651819	1
noCat_MCL131_Hd-B-R1669-Map20	0.6478961799	2.2884714862	1
RXX-TRIM_MCL180_Hd-B-G1165-Map3	0.6429418281	2.2703511169	1
RXX-TRIM_MCL238_Hd-B-G1844-Map7	0.6391408207	3.9118814922	1
DTX-incomp_MCL253_Hd-B-G2043-Map6	0.6318915448	3.8374522737	1
RXX-TRIM_MCL195_Hd-B-G1379-Map3	0.6262031527	3.7799252801	1
RXX-TRIM_MCL411_Hd-B-R235-Map16	0.6247960905	3.7658138208	1
DXX-MITE_MCL693_Hd-B-R9264-Map5	0.6234890207	1.6555564319	1
DTX-incomp_MCL684_Hd-B-R902-Map10	0.6233949533	3.7518082086	1
RXX-TRIM_MCL548_Hd-B-R507-Map4	0.6233949533	3.7518082086	1
noCat_MCL62_Hd-B-R2380-Map20	0.5904100882	2.0838032092	1
RXX-TRIM_MCL73_Hd-B-G113-Map3	0.5864027323	3.3974193202	1
RXX-TRIM_MCL113_Hd-B-G390-Map6	0.5753530305	3.2972497971	1
RIX-incomp_MCL676_Hd-B-R825-Map3	0.5646716699	3.2027818585	1
DMX-incomp_MCL578_Hd-B-R5378-Map4_reversed	0.5520867271	3.094379232	1
noCat_MCL32_Hd-B-G30-Map20	0.5263988761	1.2730907769	1
noCat_MCL5_Hd-B-P15.85-Map20	0.5174311102	1.471408109	1
RXX-TRIM_MCL95_Hd-B-G194-Map3	0.5131384268	2.7779119951	1
RXX-TRIM-chim_MCL120_Hd-B-G775-Map3	0.5009853554	1.3784244978	1
DMX-incomp_MCL512_Hd-B-R4711-Map5	0.4877238088	1.4206701288	1
DTX-incomp_MCL7_Hd-B-R1930-Map20	0.4830592436	1.5149809545	1
noCat_MCL22_Hd-B-G1253-Map20	0.4725339059	2.4763142152	1
noCat_MCL34_Hd-B-R3658-Map20	0.4674077527	2.4401506036	1
noCat_MCL123_Hd-B-P10.263-Map20	0.4657190782	2.4283406034	1
RXX-TRIM_MCL339_Hd-B-G968-Map4	0.4648784443	2.4225032309	1
RXX-TRIM_MCL665_Hd-B-R789-Map3	0.4644963818	1.475399453	1
RXX-TRIM_MCL224_Hd-B-G1719-Map3	0.4640402633	2.4166909532	1
noCat_MCL5_Hd-B-P66.85-Map19	0.4615026965	1.6654762654	1
RXX-TRIM_MCL184_Hd-B-G1227-Map4	0.45363783	1.1805146205	1
RXX-TRIM_MCL28_Hd-B-R502-Map3	0.4454128834	2.289563753	1
RXX-TRIM_MCL451_Hd-B-R341-Map5	0.4377664947	1.4189352099	1
noCat_MCL10_Hd-B-G74-Map20	0.4331461681	2.2086567971	1

RIX-incomp_MCL140_Hd-B-R6366-Map4	0.4203029107	1.5421197468	1
DTX-incomp_MCL32_Hd-B-R4819-Map4	0.406251768	1.5010596817	1
DTX-incomp_MCL256_Hd-B-G2078-Map3	0.4042905298	1.4953679096	1
RXX-TRIM-chim_MCL348_Hd-B-P274.73-Map5	0.4019518834	1.4885931129	1
RXX-TRIM_MCL637_Hd-B-R7246-Map4	0.4013326849	2.0089937354	1
noCat_MCL50_Hd-B-R1740-Map15	0.3986796423	1.9930080883	1
noCat_MCL364_Hd-B-R1253-Map14	0.3912479847	1.4577529779	1
DMX-incomp_MCL405_Hd-B-R222-Map20	0.3822734279	1.3034327869	1
RIX-comp_MCL544_Hd-B-R5026-Map5	0.3783754578	1.8733576764	1
RXX-TRIM_MCL46_Hd-B-G2097-Map8	0.3621193776	1.3751644089	1
RXX-TRIM_MCL318_Hd-B-G73-Map3	0.3436850473	1.6805912316	1
DTX-incomp_MCL225_Hd-B-G1737-Map3	0.3410486502	1.6665185354	1
noCat_MCL161_Hd-B-R815-Map11	0.3391169761	1.2148008391	1
DTX-incomp_MCL127_Hd-B-P302.46-Map3	0.3368902142	1.6444801303	1
noCat_MCL97_Hd-B-R6288-Map15	0.3312893466	1.615100795	1
RXX-TRIM_MCL28_Hd-B-G2282-Map3	0.3219210779	1.5667245447	1
RXX-TRIM_MCL114_Hd-B-G483-Map6	0.3130805068	1.2401068364	1
noCat_MCL1_Hd-B-R963-Map20	0.299843028	1.4563906564	1
noCat_MCL306_Hd-B-G583-Map20	0.2980761169	1.1996520693	1
RSX-incomp_MCL5_Hd-B-G228-Map20	0.2959376017	1.4373933238	1
RIX-comp_MCL653_Hd-B-R7590-Map3	0.2921013288	1.4188792931	1
RSX-incomp_MCL82_Hd-B-G1278-Map10	0.2891642091	1.4048018915	1
DTX-incomp_MCL259_Hd-B-G2134-Map5	0.2694245101	1.1233524744	1
noCat_MCL1_Hd-B-R4412-Map11	0.2640654804	1.2878349252	1
DXX-MITE_MCL14_Hd-B-P47.118-Map20	0.2536426545	1.2409239579	1
DTX-incomp_MCL394_Hd-B-R188-Map20	0.2450851275	1.203105246	1
RXX-TRIM-chim_MCL192_Hd-B-G1334-Map10	0.2364315959	0.9922069894	1
RIX-incomp_MCL598_Hd-B-R5917-Map3	0.2355418741	1.0031928146	1
DTX-incomp_MCL461_Hd-B-R3637-Map20	0.2353767113	0.9903427048	1
noCat_MCL412_Hd-B-R2362-Map11	0.2346684256	1.1578873661	1
RXX-TRIM_MCL71_Hd-B-G2274-Map5	0.2260147519	1.1209801583	1
noCat_MCL466_Hd-B-R3699-Map14	0.2206649633	1.0984520824	1
RXX-TRIM_MCL10_Hd-B-R1696-Map20	0.2109875594	1.0582066404	1
RLX-incomp-chim_MCL11_Hd-B-R22-Map20_reversed	0.2104350094	1.0559972371	1
RXX-LARD_MCL384_Hd-B-R1675-Map3	0.2090615066	1.0503449303	1
RXX-TRIM_MCL464_Hd-B-R3667-Map3	0.2065598622	0.959031564	1
RXX-TRIM_MCL110_Hd-B-R5307-Map5	0.1987212447	1.0082286258	1
RXX-TRIM_MCL377_Hd-B-R1609-Map4	0.1979522999	1.005127167	1
RLX-incomp_MCL54_Hd-B-P318.33-Map3_reversed	0.1919304422	0.9809773784	1
RXX-TRIM_MCL79_Hd-B-G2126-Map3	0.1723189789	0.903900026	1
noCat_MCL93_Hd-B-R2089-Map20	0.1674326606	0.8850441704	1
RXX-TRIM_MCL564_Hd-B-R5172-Map15	0.1657437361	0.8745686984	1
RIX-incomp_MCL678_Hd-B-R8483-Map3	0.1635201187	0.8700389473	1
DXX-MITE_MCL117_Hd-B-R1594-Map4	0.1462109344	0.8045676088	1
noCat_MCL5_Hd-B-G78-Map20	0.1453236875	0.8012485537	1
DXX-MITE_MCL297_Hd-B-G48-Map3	0.1359950272	0.7747208501	1
noCat_MCL315_Hd-B-G669-Map20	0.1356370048	0.7652186037	1
RXX-LARD-chim_MCL594_Hd-B-R5678-Map3	0.1345078989	0.7878228382	1
RIX-incomp_MCL15_Hd-B-G1297-Map3	0.1305252237	0.7463447871	1
DXX-MITE_MCL96_Hd-B-G196-Map3	0.1263827996	0.7311123679	1
DMX-incomp_MCL66_Hd-B-R3066-Map17	0.1244417631	0.7239923695	1
DTX-incomp_MCL113_Hd-B-R5878-Map4	0.1220663735	0.759390942	1
noCat_MCL19_Hd-B-R2562-Map17	0.1192660006	0.7528902472	1
RXX-TRIM_MCL95_Hd-B-R6534-Map5	0.1147221127	0.7174703903	1
RXX-TRIM_MCL28_Hd-B-G178-Map4	0.1112394297	0.7079398792	1
noCat_MCL9_Hd-B-R3260-Map16	0.1085343855	0.6659751842	1

RIX-incomp_MCL491_Hd-B-R4342-Map4	0.1044343647	0.6510892298	1
noCat_MCL128_Hd-B-R1465-Map20	0.1000402666	0.6351497453	1
RXX-TRIM_MCL667_Hd-B-R795-Map5	0.0975245311	0.6260265601	1
DTX-incomp-chim_MCL612_Hd-B-R6025-Map5_reversed	0.0931414881	0.6899358077	1
RSX-incomp-chim_MCL208_Hd-B-G1541-Map3	0.0891490312	0.5956339155	1
RXX-TRIM_MCL388_Hd-B-R1756-Map9	0.0863366673	0.5854104599	1
noCat_MCL15_Hd-B-R3885-Map13	0.0848698051	0.580072218	1
RSX-incomp_MCL217_Hd-B-G1583-Map3	0.0791764514	0.5593007656	1
DXX-MITE_MCL358_Hd-B-P5.265-Map20	0.0773128808	0.5524791622	1
RXX-TRIM_MCL385_Hd-B-R1706-Map13	0.0746684208	0.6892940088	1
RXX-TRIM_MCL90_Hd-B-G1523-Map3	0.068366169	0.5195071398	1
DTX-comp_MCL265_Hd-B-G2378-Map3_reversed	0.0619437942	0.5640107097	1
DTX-incomp-chim_MCL537_Hd-B-R4985-Map3_reversed	0.0615340652	0.493981838	1
noCat_MCL18_Hd-B-R4871-Map16	0.061070211	0.5612192237	1
RXX-TRIM_MCL390_Hd-B-R1837-Map3	0.0568658509	0.5914017165	1
DXX-MITE_MCL355_Hd-B-P340.11-Map3	0.055172682	0.4698285649	1
RXX-TRIM_MCL340_Hd-B-P119.195-Map3	0.0545491057	0.5399454565	1
RXX-TRIM_MCL21_Hd-B-R3610-Map20	0.0540213362	0.465405888	1
RXX-TRIM_MCL333_Hd-B-G880-Map4	0.0523701959	0.4590321415	1
RXX-TRIM_MCL313_Hd-B-G651-Map4	0.0513366438	0.4550225203	1
RXX-TRIM_MCL389_Hd-B-R1786-Map3	0.047883421	0.4415031979	1
noCat_MCL5_Hd-B-G50-Map20	0.0438426823	0.4254078205	1
RXX-TRIM_MCL335_Hd-B-G889-Map4	0.0414978164	0.4159070724	1
noCat_MCL48_Hd-B-R3271-Map12	0.0408626668	0.4920686584	1
noCat_MCL85_Hd-B-R4856-Map16	0.0313926664	0.3731481726	1
RXX-TRIM_MCL310_Hd-B-G615-Map4	0.0311198844	0.371942253	1
DTX-incomp_MCL77_Hd-B-R904-Map5	0.0299219006	0.3666060922	1
noCat_MCL509_Hd-B-R4481-Map11	0.0286037895	0.493747686	1
RXX-TRIM_MCL626_Hd-B-R6590-Map4	0.0255797328	0.346641977	1
RXX-TRIM_MCL128_Hd-B-P38.249-Map3	0.0243581546	0.3408201367	1
DTX-incomp_MCL7_Hd-B-G850-Map20	0.0222815205	0.3306744379	1
noCat_MCL140_Hd-B-R390-Map12	0.0186776674	0.5517706473	1
RIX-incomp_MCL489_Hd-B-R4307-Map5	0.014071373	0.3702452214	1
RXX-TRIM_MCL386_Hd-B-R1716-Map20	0.0113445143	0.352553533	1
DMX-incomp_MCL545_Hd-B-R5047-Map8_reversed	0.0083618304	0.419379345	1
RIX-incomp_MCL483_Hd-B-R4195-Map4	0.0067427716	0.367743497	1
DXX-MITE_MCL70_Hd-B-G1085-Map6	0.0061251295	0.2292382524	1
RIX-incomp_MCL593_Hd-B-R5611-Map4	0.0024254658	0.1868463071	1
DXX-MITE_MCL112_Hd-B-G39-Map20	0.0023776273	0.1860982987	1
DXX-MITE_MCL144_Hd-B-R9074-Map7	0.0019474929	0.178906413	1
RXX-TRIM_MCL18_Hd-B-R1776-Map20	0.001677916	0.1738712735	1
RXX-TRIM_MCL444_Hd-B-R3261-Map12	0.0008579917	0.1541730841	1
noCat_MCL524_Hd-B-R4846-Map15	0.0001496817	0.3543836046	1
noCat_MCL459_Hd-B-R3584-Map11	2.6742164455795[illegible]	0.2187300696	1
