# Supplementary material for: Genomic architecture of endogenous ichnoviruses reveals distinct evolutionary pathways leading to virus domestication in parasitic wasps
Source: BMC Biol. 2020 Jul 24;18:89. doi: 10.1186/s12915-020-00822-3 (PMC7379367; doi:10.1186/s12915-020-00822-3)
Supplement: Supplementary file 9 — Additional file 9: Table S10. List of direct repeat junctions (DRJ) found at the ends or within proviral segments genes identified in Hyposoter didymator and Campoletis sonorensis genome scaffolds. Are indicated the scaffold name, the name of the proviral segment, its length and position in the scaffold, the name of the DRJ, its size and position in the scaffold and the DRJ sequence. Nucleotide identities are indicated for each pair of DRJ. [file 12915_2020_822_MOESM9_ESM.pdf]

Scaffold	Proviral segment	Proviral segment size (nt)	Segment position in scaffold	DRJ name	DRJ size (nt)	DRJ position in scaffold	sequence	Identity DRJs (%)
**Hyposoter didymator**								
scaffold128243	**Hd44.1**	3009	4203985-4206993	Hd44.1_DRJ1R	192	4203985-4204176	ATGGTTGAGATTTGTAGTTCACGTTTCATCCGTTGATTGCAGCCGTGCTTCCGATGCATCAGATTGACTCAAACGTACCATCTTTCAAACCACGCATGGCTTCTAATGGTGAAGGCAGTGACCAGAACGCTGATTGTTTTGTTCCCTTTCGTAATTCCTTTGCCGTGAACTCTGCACTAGACATACAAAGTC	73%
scaffold128243	**Hd44.1**	3009	4203985-4206993	Hd44.1_DRJ1L	192	4206802-4206993	ATGGTTGAGCTCTGCACGTCAAGTTCCACGCGATGATTGCAGCCGAGCTTCCGATATATCAGATTGACTCAAACGTGTGGGTTTTCGAACCACGTAAGGCGTTCAATGGTGGAGATAGTCTCCATAACGTTGGTCCTATTGTTACTTTCTGTATTTTCCTTGCTGTGACAACTTCACGAGACTCACAGAGTC	
scaffold64	**Hd42**	3157	2320523-2323679	Hd42_DRJ1R	214	2323466-2323679	GCTGCACGTCGTACGGCAGGCTTTCATGGGTCTACCGTGCCTGCTGCCAGGATCGTTGTCCAACTGTTATCTGATTCGCCTGCTGACAGAAACTCGTAGCGGCGCAGTCTTACTCCTCTTGGTAATGGCTCAAGAAAATATAAAAATAATAGCTTAGGTTGCATGCCCGCGAACGTACTGACGTGCATTAGGGTTCGCTGAACGC	87%
scaffold64	**Hd42**	3157	2320523-2323679	Hd42_DRJ1L	223	2320523-2320745	GCCGCACCTCGAACGGGGCATGCTCTCACGGGTCTACCGTGCCTGCTCTGCCAGGATCGTTGTCCAACTGTTGTCTGATTCACAGCTGCTGACAGAAACTCGTAGCAGCGCGGTCTTACTCCTCTTGGTAATGGCATGAGAAAAGATAAGAAAATAATAGCTTAGGTTCCATGCCCGCGAACGTGCCGACGTGCATCAGGGTTCGCTGTAAAGCGTTATCAAT	
scaffold128213	**Hd23.2**	3362	248624-251985	Hd23.2_DRJ1R	208	248624-248831	GTGCTCCGCCGTTGATGCTGCTCCACGGTCATGTGCGTCGATGTTCGAAAGCCAGGTCAAGAAGTTTGTGAGAAATATGTGATTTTTTCTCCAACGATCACACTCTCTCCTCCGCTTGTTCCTTTGAGCGATTGCGAGCTTTATGCGGCGTCGAATAGTCACGTAGACAATGACATGCTCCGTGATGGATCCAAGACGGAACGATG	74%
scaffold128213	**Hd23.2**	3362	248624-251985	Hd23.2_DRJ1L	204	251782-251985	GTGCGCGCCCGTTGATGCTGCAGCGTCGTGTACGTCGCGATGCGACGGTCTGGTCAACAGGTTCGCGAGAAAGAAGTGAATTTCTCTTCAACGGGGCATCCTCATAGCTTCTCGTTCCGTTGGCCGATGGCAAGCTTTATGCAGCATCGAATAGTCACGTAGACAATGACATGCTCGATGGTTGATCCACGACGGGAAGCTGCG	
scaffold127348	**Hd40**	3494	926528-930021	Hd40_DRJ1R	175	926528-926702	TCCTCGCCATTCTTCGTTCACGTTTCTGTGAACAAACAATAACAAAACGTTGAATACAGTGGTGGACGAGCTCACCTGTGTTGCGGAGATCAAGAAGGAATCTTTTTTTTTCACTTTACCTCAAGAAGGACTGATGCTTCCGCCACGGTTTCTAAAAAGCTGCCGCAGGTGGTGC	89%
scaffold127348	**Hd40**	3494	926528-930021	Hd40_DRJ1L	176	929846-930021	TCCTCGCCATTCTTCGTTCACGTTTCTGTGAACAACGAGAAGAAAACGTTGAGTACAGTGGTAAACGAGCTCACCTGTGTTGCGGAGATCAAGGGGAATCTTTTTTTTTCTCTTTACCTCAAGAAGGACTGATGCTTCTTCCGCTACGGTTTCGGAAAAGCTTCTGCAGGCGGTGC	
scaffold175	**Hd38**	3664	3800393-3804056	Hd38_DRJ1R	188	3803869-3804056	AGCATGCCTCGCTGTACTGGACGTAGATATTCTAATAGATTACCGCAGCAATGGCTACGTGACAGACTCGTAAGACCAGTAGCACGGCTATAAGAGTGCAAACGATACCAAGCTCTCGGGAACCAGTCGTCGGCGAGTCATTGCACTTGCGACTTACACTGCAGAGCAGGCCAGACGCCGATGTCACT	77%
scaffold175	**Hd38**	3664	3800393-3804056	Hd38_DRJ1L	188	3800393-3800580	AGCAGGTCCCGTTGTACTGGACAACGACATTCAACAGCCTACGGCAACAGAGACTGCATGACAGACCCGTAAAGCCAGTAGCACGGCTATTGAAGTGCAAACGCCACCAAGCTCTCAATATTTATTTGCCAGCGAGTCATTGCACCAGCGACTTACCGCTGCAGAGCAGACCAGACGTCGATATCACT	
scaffold65	**Hd37**	3708	342293-346000	Hd37_DRJ1R	211	345790-346000	TTGATAACGAATTACGGTAAATGCTGATGCACGTCGATGCGTTCGCGAGCATGCAACCCAAGCTATTCTTTTGTTCTTTTATCATGCCGGCACCAAGAGATAAGACCGCGCCGCTACGAGTTTCTGTCAGCAGACGAATCGGAGAATGGTGTGACAACGCTCTTGCAGCAGCAGGCACGGTAGACCCGTGAAAGCATGCCGTACA	82%
scaffold65	**Hd37**	3708	342293-346000	Hd37_DRJ1L	202	342293-342494	TTGATAACGCATTACGTCAAACGCTAATGCACGTCGACGTGTTCACGGGCATGCAGCCTGAGCTATTCATCTTTTCTCATGCCATTACCATGAGGTGAGACCGCCGCTACGAGTTTCTGTCAGCTGAGGAACCGGAGAACGGTTGGACAACGCTCTTGACAGCAGGCACGGTAGACCCGTGAGAGCATGCCGTACAGCGTGC	
scaffold116	**Hd35**	3710	473341-477050	Hd35_DRJ1R	132	473341-473472	ATCACCGACACGCTGACGTGCCCCGCAGCAGGATGGAGCGCAGCTTCACGTCGTGGATCAACCATCGAGCACGTCATTGTTTACGTGACTATTCGATGCTGCATAAATCTTGCCAACGGCCAACGGAACGAG	91%
scaffold116	**Hd35**	3710	473341-477050	Hd35_DRJ1L	132	476919-477050	ATCACCGAAACGCTGACGTGCCCCGCAGCAGGATGGAGCGCAGCTTCACGTCGTGGATCAACCATCGAGCACGTCATTGCTTACGTGACTATTCGACGTCGCATAAAGCTCGCCATCGCTCAAAGGAATGAG	
scaffold175	**Hd36**	3738	3796140-3799877	Hd36_DRJ1R	208	3799670-3799877	ATTGTAAAGTTTTTCTGGTTGCCTATTGACTGTGGAGTGACATTAGGGTGTGGTCTGCTCTGCCGCCGTAAGTCGCAGGTGCAATGACTCGCTGGTGAATGAATGTTGAGAGCTTGGTGGCGTATGCACTTCAATAGTCGTGCCACTGGCCTCACGGGTCTGTCATACAGTCACCGTTGCCGTAGGCTGTTCAGTGTCTTCGTCCA	75%
scaffold175	**Hd36**	3738	3796140-3799877	Hd36_DRJ1L	209	3796140-3796348	ATTGTAAAATTCTCTTGTTTGCCCGATGGCTTTGGAGTGACAGCGGCGTCTGGTCTGCTCTGCAGCGGTAAGTCGCAAGTGCAATGACTCGCCGACGAATGGTTACCGAGAGCTTGGTATCGTATGAACTTTCACAGCCGTGCTGCTGGTCTTCCGAGTCTGTCACGTAGCCACTGTTGCGGTAATCTATTAAAATATCTACGTCC	
scaffold91	**Hd33**	3835	485063-488897	Hd33_DRJ1R	233	488665-488897	AATCAACATGTCCGACGTTCTAGCTTCCTCAAGCAGTTGCATGAAGCTTCAGTCGGTTTGCAACAACGATCGTTTGTTTCGTTTGCGCTGAAAGCACGTTCCAACCAATAATGGATGCGAGACTCGTCCACGGTGGCTCGTGGGTTTAAATTCCGCTTTGGACATGCATTCATCGTCCTGATTGACGTTCCAACTGGACAGATACGATTCCTTCAAGCTCGAAGAGTCTACTA	81%
scaffold91	**Hd33**	3835	485063-488897	Hd33_DRJ1L	234	485063-485296	AATCAACATATCCAACGTGTTCGCTTTCTCAGGCAGTTGAATGAAGCTTCAGTAGGTTTGCAACACCGATTATTTATTTCGTTTGCGATGAAAGCACGTTCAACCGAATAACGTATGCGAGACTCGTCCGCGTTAGCTTCGCGGTTCTGAATTCCTTTTAAAACCTGCATCCAACGTCGTGCTTGTCGTTGCAACTGTCCAGATACGATCCCTCCATGCTCGAAGAGTCTGCTA	
scaffold127548	**Hd27**	4002	13338255-13342256	Hd27_DRJ1R	140	13342117-13342256	CCATCGCGAGCTACCGATAGCACCGCTCTGCACGTGACTATCCGAGGCTGCATCGAGATCTTGGTAACACAATAGAACGAGGTGCGCCGAGTGCGCTATCTTTGAAGAAAAAACCATGTATTCCGCTCGAGCGTATCAAC	81%
scaffold127548	**Hd27**	4002	13338255-13342256	Hd27_DRJ1L	138	13338255-13338392	CCATCGAGCTACTGATAAGACCGCGACGCACGTGACTCTTCACCGCCGCATGGAGTTCGTGGTCACACAATAGAATGAGGTGCAGCGACTGTGCTATCTTTGAAGAAAAAACCATGTATTCTACTCAGGCGTATCACC	
scaffold29771	**Hd46**	4109	19344-23452	Hd46_DRJ1R	555	22508-23062	ACTTTGGCATGATGCCCGAGTTCAGCCGATAAAATCCTAACATACCATTTCTCGAAGCCAACCGCTCCCACGTATACACTACCCGGCCACAGCACGAAGCGACGATTTAGGAGGACTCGTCAACGCAACATACCGGCATTTCCCTAACTTCCGATCGATCATCTATGTACTGCATCGAAAGCAAGTTTTTTCTCGGGAACCGATGTGCTCGGTCCGAGCTTTGAAGAATTGTTTGCAGATAGCAGGGTAGTTAAGTACAGGTATGTGAAGTCTACAATCGCGGTTTTGAAGTCTACAATAGGGATTCTTTACAGTACTCGAGAAGGTCCCTTTGAGTGTGAAATCGTCAAGAGAATGCACAGAGTTCAATGATAAACGAGAGTCGTTCGGAGTCTGCATCATAACTAATGATTTTACGGCAACCTGATGACTTGTGCAATCTTGCCGGAGATGGAGTGAACTGCATATCACGTATGTACTAAACCGAATAGCAAGTGGTGATAAACAGACTATGTTCTGGGGGTTCCGAAAGACTTTGCGACTTAGCAAGGCCCG	82%
scaffold29771	**Hd46**	4109	19344-23452	Hd46_DRJ1L	558	19344-19901	ACTTTGGCACGATGCCCGTGTTGACTGGATAAATGCCTAAAATAACTTTTTTTTCAAGCCACCAGCTTCCAATGGGACACTGCTCGGTCACAGCACGAAGCTACGATGCGGGAGGACTCGTAATCGCAACGTACCGGCTTTTCCCCAACTGCCGATCGATCATCTATGTAATGCGTCGAACTCAAATTTTTCTTCTCGGGAACCAACGTGCTCGGTCCAAGCTTCGAAAAATTGTTTGCAGATATCAGGGTAGTTAAGTAAAGGTATGTAAAGTCCACAACCGCGGTTTTGAAGCCCGTCACTACGGATTCTTTTCAGTGCTTGAGAAGGTCTCTTTGAATGTGGAATCGTTAAGAGAATGCACACAGTTCAATGATGAACGAGAATGGTTCGGATTCTGCATCGTGACTAATGATTTGTCGGCAACCCCATGGCTGATTTAATCTTGCGGGAAATGGAGTGAACTGCATATCCCGTATATACTAAACCGAGTAGTGAGAGTGGTAATAAACAGACCATGTTCTGGGACTTAGTAAGACTGCGACATAGCAAGGCCCG	
scaffold29771	**Hd46**	4109	19344-23452	Hd46_DRJ2R	372	23081-23452	ATACGCAATAGAACAACGTGGTGGTCTGGATCTGTTCAAGCTTGAAAACTGATGTTCTTTTCACGGCAACAAGCCTTCGTTAATTCAGATGAGCAATGTAAACGTGATCAAGCACAGGCACTGGACCACTTGACGTACTTCCAATGCGCTTCTTTAGTTGCGATAAATAAGCCAGTCACTGTGAGGTCAGGGTCATACATCCTCACACTGAAAACCATGGATGAAATGCAAAGACAATAATCACCGAACCACCTAATGTCACTGTAAAATAGTACTATATGAATGAAATAGTCGAATGCATAATTCATCACTAGTCGGCCGTCTATAATGACGATGGCCGTAACCTCAATTGACCTGTTTCACAATTCGGTA	88%
scaffold29771	**Hd46**	4109	19344-23452	Hd46_DRJ2L	371	21450-21820	ATATGCAATAGAACAACGTGGTGGCGTGGAACTGTTGAAGCTTGAAAACTGACGTTCTTTTCACGGCAACAAGCCTTTGCTAATTCAGATAAGCAAAGTGAACGTGTTCAAGCACGGGCATTGGACCACTCAACGTACTTCCAATGCGCTTCCTCAGCTGCGATAAATAGGCCAGTCACTGTGAGGTTAGGGGTATACATCCTCACACTGAAAACCATGACGAAATGCAAAGACAATAATCACCGAACCACCTAGTGTCACTGCGAAACAGTACTATATGAATCAAATAGTGAAATGTATATTTCATCATCTATCAGCCGTCTATAATGACAATGGCCGAGACCTCAATTGAACTGCTTAACAATTCAGTA	
scaffold127549	**Hd31-34**	4119	144445-148563	Hd31-34_DRJ1R	129	148037-148165	GGTATCAATAAGTTGCAAGTCAGCAGGGCATCTACAAAATGCAGTTGGAGCACAGCCAACAAATACCATATTAAGTAATCACCTACTTAAGCGATAAGCACTCCGGCACGCGTGATCAGAGCGCATACT	86%
scaffold127549	**Hd31-34**	4119	144445-148564	Hd31-34_DRJ1L	129	144445-144573	GGTATCAATAAGTTACAAGTTAGGAGGGCATCTATAAAATGCAAGTCGGGGACAGCCAACAAACATCATATAGAGTAATCACCCACTTAAGCGATAAGCACTCCGGCACACGTGATCGAGGCGCATACT	
scaffold127549	**Hd31-34**	4119	144445-148564	Hd31-34_DRJ2R	396	148166-148563	CGTGCTGAGCAATGAGCTCAAAATGCACGTATGGTCATGTGCATTGATAACGTACGTCGTAGTGGCCGGTCTGGAAGGCCGATTTTAGCACAATCATCACGAACTTGGAGAATAGGTCGATCGAACCGTGTTACATCCGTCAGTGCATGCGGATGCCATCAACTGCTTCAGAACCCGATGGTACACTCCATGATCTCTAACTCTCGCGGAATAATCAGTAAATCTTTGAAGTGACCAATCAACACTCTCCCGACACAATGATGCGGAGCATGCGGAGGTTTGCTTTATGACAACTTTGAGACCCCGTCGATGCCATAGAGGTATGGCTTGTCGAACGGAGACTACGAGCGCAGTGCATCGTCACAAGTTCCTGTCCTGTTTCATCAACTAGAAATG	89%

scaffold127549	**Hd31-34**	4119	144445-148564	Hd31-34_DRJ2L	394	144723-145116	CGTGCCGAGCAATGAGCTTAAAATGCACGTATGGTCACGTGCATTGATAACGTACGTCGTAGTGGCCGGTCTGGAAAACCGATTTTAGCACAATTATCACGAACTCAAAGAATAGGTCGATCGAACCGCGTTACATCCGTCAGTGCATGCGGATGCCATCAACTACCCCAGAACCCGTTGGTACACTCCACGATCTCTAACTCTCGCGGAATAATCAGTAAGTCTTTAAATTGATTAATCAATACTCTTTCGACATAATGATGCGGAGCATGCGGAGGTTTCCTTTAAAACAACTTCGAGGCCTCGTCGATGCGATAGAGGTACAGCGCGTCGAACGAGGGTTACGAGCGAAGTGCACCGCCAAGTTCCTGTCCTGTTTCATCAACTGGAAATG	[illegible]
scaffold22	**Hd39**	4122	1002620-1006741	Hd39_DRJ1R	423	1002686-1003108	AGCTGAATACGACAGAGTGAGGTGCTATCAAGAATGAACTAGAAAACATTTCCAAAGTTGCCGTGAGTTTCCGAAACCACTACGATCATGGGAAGCCTCTTTTGGCAAGCACGCTCTACAGGAGCGAGACAGAGACTTTGAGCTGCTCAGAACGTTCAGTGGCGTCCGATGATCTACGGCACTAGTCGAATCGCTTTAGTAGTTGTGCTGGCTGACAGCGTTCTCAATCAAATGGTCTTTCTGCACGTAGAACAACAAGGTGTACTGTTGAATGTTTGTAGGAAAATCTAGTTTCCTAACGCAGATAGATGCTGGGATGTCGTGCAGCGACATAGATTTGAAGTAGTTTGAGCGACACTCCGCTACACGTTATTCGTCACAATGCTACACAGTGCTGCAAGATTTTCAAGCG[illegible]	94%
scaffold22	**Hd39**	4122	1002620-1006741	Hd39_DRJ1L	423	1006319-1006741	AGCTGAATACCACACACAGAGTGAGGTGCTATCAAGAATGAACTAGAGAACATTTCCAAGTCGGCCGTGAGTTTGCGAAACCACTACGATCATGGGAAGCCTCTTTTGGAAAGCACGCTCTACAGGAGCGAGACAGAGACTTTGAGCTGCTCAGAACGTTCAGTGGCGTCCGATGATCTACGGCACTAGTCGAATCGCTTTAGTAGTTGTGCTGACAGCGTTCTCAATCAAATGGTCTTTCTGCACGTAGAACAACAAGGTGTACTGTTGAAAGTTTGTAGGAAAATCTAGTTTCCCAACGCAGATAGATGCTGGGATGCCGTGCAGCGACATAGATTTGAAGTAGTTTGAGCGACGCTCCGCCACACGTTCTTCGTCACGATGCTACACAATGCTGAAAGATTTCCAAGCG[illegible]	
scaffold22	**Hd39**	4122	1002620-1006741	Hd39_DRJ2R	307	1002620-1002926	GGCATATGTGCAAGTCAAAGGCGAATGAAGGGAATCATACAATTTGTCATTGCGGGCTGTTAACGTAGCTGAATACGACAGAGTGAGGTGCTATCAAGAATGAACTAGAAAACATTTCCAAAGTTGCCGTGAGTTTCCGAAACCACTACGATCATGGGAAGCCTCTTTTGGCAAGCACGCTCTACAGGAGCGAGACAGAGACTTTGAGCTGCTCAGAACGTTCAGTGGCGTCCGATGATCTACGGCACTAGTCGAATCGCTTTAGTAGTTGTGCTGGCTGACAGCGTTCTCAATCAAATGGTCTTTC	84%
scaffold22	**Hd39**	4122	1002620-1006741	Hd39_DRJ2L	332	1005941-1006272	GGCTTATGAGCAAGTTGAAGGCGAATAAAGAGAATCATACTGTTTGTTATCTCTGGCTGTTAACGTGGGGCCATGTCTCGTTGAGTCACTGAGCTGAATACCACGTACGGAGTGAGGTGCTATCAAGAATGAACTAGAAAACATTTCCAAGTCGGCCGTGAGTTTGCGAAACCACTACGATCATGGGAAGCCTCTTTTGGCAAGCACGCTCTACAGGAGCGAGACAGAGACTTTGAGCTGCTCGGAACGTTCAGTGGCGTCCGATGATCTACGGCACTAGTCGAATCGCTTTAGTAGTTGTGCTGACAGCGTTCTCAATCAAATGGTCTTTC	
scaffold1868	**Hd43**	4159	193639-197797	Hd43_DRJ1R	69	195404-195472	GCGGAGATAAGAAAGTTAGTTTCGAAGCTCAAACAGATTCAGACCACGACGTTGTTCTATGGCATACGG	83%
scaffold1868	**Hd43**	4159	193639-197797	Hd43_DRJ1L	69	197729-197797	GCGGAGAAAAGAATGTTAGTTTCGAAGCGTAACGGTTCCAGACCACCGCGTTGTCCTTTGGCATACGGA	
scaffold1868	**Hd43**	4159	193639-197797	Hd43_DRJ2R	949	193639-194587	ATGGAACAGATGTTTCAATGCACAGTCCTGCATTAGTCGGAAGTATTTGTTACGGTCGTCCATCTCGAAAGAAGTTCGTGAACGTAATGAGTCATGTTCTCCGATTCGCATCTATTTATAGATAACACACTGTATACGAGTCGCGAACGGTTCGCTATGGTTGGGATTCCCCGCGTTTTATTTCGTCGCGATTTTATTGACCCGGCTATTTTAAATATTCAGACGGTTGGTTATATATCTATCTGCGGTTTCTCACCGGGTTGAACCCGGAAGCCAATCGGGCCTTGCTAATTTCTTCAGGAATATGTTGATCATAGGTTGTTCGAAAGATATCCGAAAGGTATGAATAACATTGAAGTCTTCCCGAAGTATCAGAACATCGTTTGTTCATTACGACATAGTACTCAGTTTGTTTCAGTATATACGTGATATGCAGTTCGCCCAATCTCCGGTAAGGTTGGAGGAGCCATGAGCCAACAAATCATTAGTCATAAGCCAGACTCCGAACAACTCTCGCTCATTACTGAACTGTCTATATTCTCCTGACAATTGCACACTCGAACGAATCTCCTTACGCATTGAAATGTATCCGTAGGGTGAAGAGTAACTCATGTTGTAGCTTTGTACGTTCATATACTTATATACCCTGTTATCTGCAGACAATCCTTCGAAGCTCGGACCGAGCAGGTTTCGGAGGAGGAACACTTGTGTTCGACGCACCACGTAGTTGATCGATCAGTGGTCGGAAAGGAACTGGTACGGTGTGATTACCACCCATGTCTCGAGTCGTTGCTTTGTGCTGTGACCGAACAGCGTGTAGTAAGTTGGAGCGGTGTCTCGACAAATGGTATTTCGGGATTTTATTAACCGAGCTTGGGCATCGTCCCGCAGTTGACAGAAGCTCATGTTCGTTCTCAAATTTACGGAGTGATGTTCCTTCTTCCACCAT	92%
scaffold1868	**Hd43**	4159	193639-197797	Hd43_DRJ2L	945	196556-197500	ATGGAACAGATGTTTCAATGCACACTTCTGCATTAGTTGGAAGTATTTGTTACGGTCTGCCATCTCGAAAGAAGTTCGTGAACGTAATGAATCATGTTCTCCGATTCGCATCTGTTTATAGATAAGACACTGTATACGTGTCGCGAACGGTTCGCTATGGTTGGGATTCCCCGCGTTTTGTTTCGTCGCGATTTTATTAGCCCGGCTATTTTAAATATTCAGACGGTTGGTTATATATCTATCTGCGGTTTCTCACCGGATTGAACGCGGAAGCCAATCGGTCCATGCTAATTTGTTCAGGAATATAATGATCATAGGTTGTTCGAAAGATATCCGAAAGGTATGAATAACATTGAAGTCTTCCCGAAGTATCAGAACATCGTTGGTCCATTACGACATAGTACTCAGTTTGTTTCAGTATATAGGTGATATGCAGTTCACCCAATCTCCGGTAAAGTTGGACGAGCCATGAGCCAACAAATCATTAGTCATAAGCCAGCCTCCAAACAACTCTCGCTCATTACTGAACTGTCTATATTTTTCTGACAATTGCACACTCGAACGAATCTCCTTACGCATTGAAATGTATCCGTAGGGCGAAGAGTAACTCATGTTGTAGCTTTGTACGTTCATATACTTATCGACCTTGTTATCTGCAGACAAACCTTCGAAGCTCGGACCGAGCGGGTCGGTTTCAGAGGAGGAACACTTGTGTTCGACGCACCACGTAGTTGATCGATCAGTGTTCGGGAAGAAACCAGTACGTTGCGATTACCACACACGTCCCAAATCGTTGCTTCGTGCTGTGACCGAGCAGCGTGTAAGTTGGAGCGGTGCCTGGAAAAATGGTATTTCGGGATTCAATCAGGTGAGCTTGCGCATCGTCCCGAAGTTAGAAGCTCATGTTCGCTCTCAAATTTCCGGAGTGATGTTCCTTCTTACCAT	
scaffold67	**Hd30**	4164	5867182-5871345	Hd30_DRJ1R	176	5870745-5870920	ACATGGTGTATGTTCAGACCAAGTCTCCCTCGCTACGAGGCTAGTTGGTTGGTCCATCCGTGCATTGCACGCTCGCTGCGGGTTGGCACCCAGCATCGTGCCACTACTGCGACTCCGGCACCTTACGAGCTGCAGCGATAAGACACTTCGTGCTGTGGAGCACGGTGTCAACGGTG	89%
scaffold67	**Hd30**	4164	5867182-5871345	Hd30_DRJ1L	179	5867182-5867360	ACGTGATATATGCTGAAAACAGGTCTGACTCGCTACGAGGCTAGTTGGTTGGTCCATCAGTGCATTGCACGCTCGCTACGGGTTGGCACGCAGCATCGTGCCACTGCTGCAACTCCAGCACCTTACAAACTGCAGCGATAATACACTTCGTGCTGTGGAGCACGGTGTCAACGGTGT	
scaffold67	**Hd30**	4164	5867182-5871345	Hd30_DRJ2R	98	5871248-5871345	TCACAGTCGCCACTAGTGCTGAATTAAAAGATATTTTTCCTACGCGAGGCATCATTATCGCTGTGACCTTATCCGACAAGCTGTTGCACATAATAATG	77%
scaffold67	**Hd30**	4164	5867182-5871345	Hd30_DRJ2L	107	5867359-5867465	TCCCAGCTGCCGCTGGTGCTGCATATATTAAAAGATATTTTTCTACAAGAGGCATCCTTATCGCTGTAACCTTTTTCGAAACGCTGTAAATATGCACATAGTAA	
scaffold161	**Hd25**	4174	2449400-2453573	Hd25_DRJ1R	193	2453381-2453573	ATTCACGACTCGTTGAGAACGGATCGCAGTTGTCGTAATAGGCATATGCAAGCCCAGCTATCATTGTGTCATTTGGAGGCAGACAGAATTATGTCGGAGTCATAATCCGTCTTCAGCTCTCTCGAGCTCTGTGTACCATTTTAGTGAACCGATAGGAATCGTGGAAAAGCGATGAGAATGAACAACCAGTCCA	85%
scaffold161	**Hd25**	4174	2449400-2453573	Hd25_DRJ1L	191	2449400-2449590	ATTGACAACTCGTAGAGAACGGATCTCAGTTGTCAAATAAGCATATGCAAGGAAAGCCATCATTGCGTCATTTGGGGGTAGACAGGCTTACGTCGGAGTCATAATCCGTCTTCAGCTCTCTCGAGCTGTGTAACACCATAGAGAACTGATAGGAAACGTGGAAATGCGATGGGAATGAACAGCCGAGTCCA	
scaffold119	**Hd22**	4178	698427-702604	Hd22_DRJ1R	105	698427-698531	CAATGGCAGGAGCTTGACAAGGCAGATGATATCGAGTGAGTGCGTAGGTCTCTGGAACCGCGGGTCGTGAACAATATCGTGACCATTGATCGCCACAGCGAC	90%
scaffold119	**Hd22**	4178	698427-702604	Hd22_DRJ1L	105	702500-702604	CAATGGCAGGAGCTTGGCAGGACAGATGACATCGAGTGAATGCGTAGGAGTCTGAAACAGCGCGTCGTGAACAATATCGTGACCATTGATCGCCATAGCGAC	
scaffold91	**Hd29**	4356	572514-576869	Hd29_DRJ1R	458	576412-576869	ATTTCGTGGCAAGCGACTTTTCATCGTAGAACGGATGGCCACCGCTGTGCGTTGTTGAACCAATGATCCAACTTTGTGTTCTTCAGGAACGGTGAACATTCGTGCAATCAACTTGACACGTCGTCATGCGGCAAGAAACTCGATAGACCATGAGGAATATCGTAACGTCATTCATCGTGCACGCATTCCTAGGACAAGTCTCTGCGCTCGAATCGTTGCGGATGCATTTGGAAGCAATGTGTCTCCAATTATGTTGGGAAAAAGTGTCCAAAAATTTATTAGATTGACCGAATGTTAATTTGAAGCTCTTGAGAAATTCACAGCTCGAGGTTAACGAGAGTGTGAAGGGATAAGCGTCACGTTCTTCATGGACGGTGCGGCTCGCACACGTCGCATGGAGCTTGCGAGTTCCAAATAAAACAACTGCGTGGGGTATTGTTTATTTCTTGAGAGCACCG	82%
scaffold91	**Hd29**	4356	572514-576869	Hd29_DRJ1L	450	572514-572963	ATTTCGTGGCAGGCTTAGTTTTGATGGAGGGACGTAGCGATCGCAGTGCGTCGCTGCATCAATGATGCAGCTTTGTGTCCTTCTCGAACGGTGAACATTCGTGCAACCAACCTGACACGTCGCCATTCGGAGAGAGGTTCGATAGACCATGAGGAATATCCAAACGTCATTCATTGTGCGCACTTCTAGGCTATTGCAATCGAATCATTGCAGATACGCATTTTGAAGCAACGCGGTTCAAATGATGTTGGGGAAAAGTGTGCAAACATTTATTAGATTGACCGAATGTTCATTTGAACCTCTTGAAAAACTCACAGCTCGAGGTTGAGAGTTTGACGGGATAGACGTGACGTTCTTCATGGAAGGTGTGGCTCGCAAACGTCGCATGCAGCTTGCGAGTTCCAAATGAAAACAACTGCGCGGGGTATTGTTTATTTCTGGAGAACACCG	
scaffold64	**Hd21**	4368	2353107-2357474	Hd21_DRJ1R	116	2357359-2357474	TCCATTTTCGATCCTCGTGAGCTGCGGGGGCTGTGAGTGACGTTCCTGGAAACGTTGAGCAGCTTCTTCATCCGTGACAAAGATGGTCTCGAATTGAGGAGCTTATCATACGGCAG	81%
scaffold64	**Hd21**	4368	2353107-2357474	Hd21_DRJ1L	114	2353107-2353220	TCAATTTTTGATGCTCGTGAGCTGCGGGAGTCGTGAGCGTCGTTCCTGGTCACGTTGAGCAGCTTATTCACCCCTGACAAGGATTGCCTCGAATTGGAGGCTTTGCAACGGCAG	
scaffold198	**Hd19**	4440	1265454-1269893	Hd19_DRJ1R	105	1269789-1269893	TTTTTGCTGCTTCATGTCTCAGAGCAGCTTGTCTACATGATACACGCTGATTTGTCTGTGACGGCACGATAATTCGCCTCATGCTGCACAAGCTTTGTGAGCTT	86%
scaffold198	**Hd19**	4440	1265454-1269893	Hd19_DRJ1L	106	1265454-1265559	TTTTTGCTGGTTCATGTTTCAGAGCAGCCGGTCTATGTGATACACGCTGCTTAGCCTGGTGACGGCACGATGGTTCCCCTCATGCTGCACGAGCTCTGTGAGC	
scaffold128213	**Hd23.1**	4457	208205-212661	Hd23.1_DRJ1R	137	208205-208341	TCCTTTGAGCGATGGCGAGCTTTATGCGGCGTCGAATAGTCACGTAGACAATGACATGCTCCGTGGTTAATCCAAGACGGAACGATGCGTATCCGCCATATTGTGGGGCATGTCAGCGTTTCGGTGATAAGGTTGCA	81%
scaffold128213	**Hd23.1**	4457	208205-212661	Hd23.1_DRJ1L	136	212526-212661	TCCGTTGGCCGATGGCAAGCTTTATGCAGCATCGAATAGTCACGTAGACAATGACATGCTCGATGGTTGATCCACGACGGGCAGCTGCGCTCCGTCTTGCTACGGGGCACGTCAGCGTTTCGGTGATTGCGTTGCA	
scaffold127548	**Hd47**	4503	12134587-12139089	Hd47_DRJ1R	85	12139005-12139089	CTGCTGGCCAGCACGTTCCACGAACAGTCTGAGATCTTGAACTGCGGAGAACGTTCAGTGGTCTCACACGATCTACGGCAGCACT	87%
scaffold127548	**Hd47**	4503	12134587-12139089	Hd47_DRJ1L	85	12134587-12134671	CTGCTGGCAAGCACGTTCTACGAACAGTCTGAAACCTTGAATAGCGTAGACCGTACAGTGTTCTCGCACGATCTACGGCAGCAGT	

scaffold264	**Hd28**	4614	135485-140098	Hd28_DRJ1R	639	139460-140098	GCCTTTGGAGCTAAACTTGCACGAGATTTCGGTGTCCGTGATAACTGAATGGCATTGTCAGGCGTAATATTTATGACGGCGGTGATGTTAACGTAGGTTCAGGCGTTAGACTGAGAGCCGGAAGGTATGGTAGTCGTTCGACCGTCTCCGAATCACGCTGACGAGAACTCTTCGTTCATCGCTTAAGCTCTGAGGACTTGACGCAATCAAGTTCTGGATGATAAACGCCAGACGCAAAGTTTTGTTTCCATAGATCACAAGTAAGAACAGTTCTTACGGACCTGACAGTCCAACATTACTCTCACGACACTGTAAAACCTATGGACGTACTTCGCACGTACCCGAGTCCACGAAACACTCGTGCTACATCCATTTGACGTTGGCCTTCTGACAAGGCCCACTATAGAGTCCCAGCGTGTGCCCGTGTCCCGGAAGGAGCTTTTACTCCCAACCGACTTTTTAGTGTGACCTTTGTCGTTACAGTTGCGTTACGTAACTACTACTGTGGAACTTTTCACTACCACAGTTACAACAGCTTATCGGCAACAGTTCCTTCAGTATCAAGAAGTAATTATTCAAGTGGAGAATGGCCGGGGTCGGAGTTGCATGAAAAGCACGGAAGTGTGTACAATACAGACG	78%
scaffold264	**Hd28**	4614	135485-140098	Hd28_DRJ1L	664	135485-136148	GCCTTTGCAGCTGAGCTGCATGAGGTTTTCATGTCCTCGATAACTGGATTCCATTGTCAGGCGTAAGCATTATGACGGCGGTGGTGTTAGGCGTTGCTTGGGAAGGTTGGTGAGCCACGTCGACGTAGATTCAGGCGTGAAACGGAGAGCTGAAAGGTATAGTAGTCGTTCGACCAACTCGGAATCACACCGACGAAAACTATTTGTCCATTGCTCAGGCTATGAGGACTTGAGGCAATCAAGTTCCGGACGCAAAGTTTTGTTTCCGGCCATAGATCACAAGTAAGAACAGTTCTGGCAGACTGGACAGTCCACCGATACCCTCACAAGACTGTAAAACCTCTGGACGTACTGCGCACGTACCCAAGTCCACGAAACACTCGTGCCACATCCATTTGATGTTGGCTTCTGCCAGGGCATGTCGCTATAGATTCCCAGCGTGTGCCCGTGTCCCAGAAAAAGCTTTGTTCACAACCGCGACTTTTGACTGTCACCTTTGTTGTTACAGTTGCGTTACGTAACTACTACTGTGGAACTTCTCACTGCCACAGTCACAACAGCCTATCGACAACTGTTCTTTCAGTATCAAGAAGTCATTATGCAAGTAGAGAATGGCCGGGGTTTTAAGTTGCATGAAAGCACTCAATTGTCTACAATGCAGACG	
scaffold82201	**Hd51**	4632	1077-5708	Hd51_DRJ1R	221	5368-5588	GCACGTTGTACGGCATGCACTCACAGGTCTACCGTGCCTGCTGTCAAGAGCGTTGTCCAACTGTTCTCCGATTCATGTGCTGACAGAAACTCGTAGCGGCGGTCTTATCTCTTGGTGCTGGCATGATAATAGAACAAAAGAATATAGCTTAGGTTGCATGCCCGCGAGCGCATCGACGCGCATCAGCGTTTGCCGTAATGCGTTTATCAATGACGAGCCAT	89%
scaffold82201	**Hd51**	4632	1077-5708	Hd51_DRJ1L	222	1077-1298	GCACGTTATACCGCATGCTTTCACGGGTCTACCGTGCCTGCTGCTGCAAGAGCCTTGTCCAACCATTCTCCGATTCGTCTGCTGACAGAAACTCGTAGCGGCGGTCTTATCTCTTGGTGCTGGCATGATAAAAGAACAAAAGAATAGCTTAGGTTGCATATGCCCGTGAACGCAGCGACGTGCATTAGCGTTTGCCGTAATGCGTTATCAATGACGAGCCAT	
scaffold82201	**Hd51**	4632	1077-5708	Hd51_DRJ2R	122	5587-5708	ATTTGTTTCTGTTTGCTTCGGTTTATAGCAACGCGAAGAACGGCCACCGTTGTTATCACTCTCGAGCGACATACACACGTGAGAGTGATGATGATTGTTGAGGCGGCCGATACAAAAGGCAG	95%
scaffold82201	**Hd51**	4632	1077-5708	Hd51_DRJ2L	120	1473-1592	ATTTGTTTCTGTTTGCTTCGGGTTAGCAACGTGAAGAACGGCCACCATTGTTATCACTCTCGAGCGACATACACACGTGAGACTGATGATGATTGTTGAGGCGGCCGATACAAAAGGCAG	
scaffold351	**Hd18**	4696	2681961-2686656	Hd18_DRJ1R	118	2686539-2686656	AGCGACGAATGCTCGTCATACAGAAACGATGTCAGCAGGTCGTCCGACTGAAACGCTTGCAGAGAAGACTCAAACTCAACAGCTAATGGTTCGAGAAAAACTGATTGAGCTGGTAGAA	81%
scaffold351	**Hd18**	4696	2681961-2686656	Hd18_DRJ1L	118	2681961-2682078	AGCGACGGCGGATCGTCATTCAGAAACAATGTGAGCAGGTCGTTCGCCTGAAACACTTGCAGACAATGTTTAAACCCAACGGCTCATGTTTCACGAAAAACCAATTGAGCTGGTAGAA	
scaffold91	**Hd24**	4697	535698-540394	Hd24_DRJ1R	662	539733-540394	CGTCTGCATTGTAGACAATTGAGTGCTTTCATGCAAATTAAGCCCCGGTCATTCTCTACTTGTATAATGACTTCTTGATACTGAAAGAACAGTTGTCGATAGGCTGTTGTGACTGTGGCAGTGAGAAGTTCCACAGTAGTAGTTACGTAACGCAACTGTAACAACAAAGGTGACTGTCAAAAGTCGGTTGGGAATAAAGCTTTTTCTGGGACACGGACACACGCTGGGAATCCATAGTGACATGCCCTGGCAGAAGCCAACGTCAAATGGATGTGGCACGAGTGTTTCGTGGACTCGGGTACGTGCGCAGTACGTCCATAGGGTTTCACAGTCTTGTGAGGGTAATGGTGGACTGTCAAGTCTGTCAGAACTGTTCTTACTTGTGATCTATGGCCGGAAACAAAACTTTGCGTCCGGAACTTGATTGCCTCAAGTCCTCGGAGCCTGAGCAATGGACAAATAGTTCTCGTCGGCGTGATTCCGAGGCGGTCGAACGACTACTATACCTTTCAGCTCTCCGTTTCGCGCCTGAATCTACGTCGACGTAGCTCACCAACCTTCCCAACGAACGCCTAACACCACCGCCGTCATGATGCTTACGCCTGACAATGGAATCCAGTTATCGCAGATATGAAAGCCTCGTGCAGCTCAGCTGCAAAGGC	77%
scaffold91	**Hd24**	4697	535698-540394	Hd24_DRJ1L	628	535698-536325	CGTCTGTATTGTATACACTTCCGTGCTTTTCATGCAACTGAGACCCCGGCCATTCTCTACTTGAATAATTACTTCTTAATACTGAAGAAACTTGTTGGGTCTGTGGCAGTTAAAAGTTGCGCAGCAGTAGTTACGTAACGCAACTGTAACGACAAAGGTGACAGTCAAAAGTCGGTTGGGAATAAAAGCTCTATCCGGGGCACGGGCACACGCTGGGACTCTATAGTGCGACATGCCCTGTCAGAAGCCAACGTCAAATGGATGTGGCACGAGTGTTTCGTGGACTCGGGTACGTGCGCAGTACGTCCAGAGGTTTTACAGTAACGTGAGGGTAATGTTGGACTGTCAGGTCCGTCAGAACTGTTCTTACTTGTGATCTATGGAAGCAAGACTTTGCGTCTGGCGTTTGTCGTCCAGAATTTGATTTCGTCAAGTCCTCAGAGCTTAAGCGATGGACGAAGAGTTTTCGTCAGCGTGGTTCGGAGACGGTCGAACGACTACCGTACCTTCCAGCCTCAGTCTAACGCCTGAACCTACGTTGATATCACCGCCGTCATAAATATTACGCCTGACAATGCCATTCAGTTATCACGGACACCGAAATCTCGTGCAGTTTAGCTCCAAAGGC	
scaffold128243	**Hd44.2**	4831	4197203-4202033	Hd44.2_DRJ1R	287	4197203-4197489	CAGTTCGTCAGTGGGATGAACAGTGCTACCGAGCCCTGCTTTCAACTTTGCAACAGATTTGGAACCTGCTACTCACACTTCGTCTATCTTATGTATTAGCTACTTGGACAATGACGTCTATCCATCAGGACCAGTTTGTATTGGTATATTATATCTTACCAGAGGTATGCAAGTTTGTGGCAACGTCTTAAGCACCAAATGAGGATTTCTAATGTTTCGGACACTGTACGTCCCGTGCAGTTGTCACGGCTAGAAAATCATGAAAAGCAACCAGAGGACAAGCGTTC	80%
scaffold128243	**Hd44.2**	4831	4197203-4202033	Hd44.2_DRJ1L	288	4201746-4202033	CAGTTCGTCAGTGGGCTAAACAGTGCTACCGAGCCCTGCTTTCAACTTTGCAATATATTTGATACCTGCTAACTACCACTCTTCTATCACATTTATTGGGTACTTGGACGATGCCATTTATCCATCACGAACGATTTGCCATTCGCATCTTATACCTCATCGGATGTATGCGAGTTTGTGGCTACGTCTTTTGAACCGAATGTACATTTCTAATGTTTCGGACTCTGTACGTCCTGTGCAGTCGTCACGGCAAGAGAATTATGGAAAGGAAGGAGACGATCAACGTTC	
scaffold91	**Hd15**	4987	469105-474091	Hd15_DRJ1R	210	473882-474091	GCTCTGTAATCTCGAATAAAACCAAAACATTCAGTTCATGCCTTGATATGAGCACCGTGAACGTTCATCAAAGACGTTCTGCCGGGTCGTGCCGATTACACTCAGTCTTTAAGATCTGGAAGACTCCCTGAATAGTAATGGGTGATAAGATGGCTGGAGTACGAATAGCAGTTGATCGAACAGCTTGTGGGAGATTACCAAATAGTC	78%
scaffold91	**Hd15**	4987	469105-474091	Hd15_DRJ1L	210	469105-469314	GCTCTGTAGTTTCGAATGAAAGGAGAACGAGTTGTTCATTTTTTGTGATATCCACTGTGAACATTATCCGACAACGTTCCACCGGGTCGTGCCGATTACACTCGGTCCTTAAGCTCTGGAAGACTACCTGAATAGCAACGTATGATAAGATTGCTGGAGGACGGATACCAGTTGACCGAACAGCCCGTGGGTGATTACTAAATGGTC	
scaffold175	**Hd26**	5018	10942034-10947051	Hd26_DRJ1R	592	10945930-10946521	ATCATGCTTGGTCATGTCGATCATTGCTGTGATGCGGTTATTGTGGTTGATGAATCAACGATCAATGTCGAAACATGTGTGACGAAACTCTTGACAAGGCAAAGTGCTGACTGTTGCGGTATCAATAAGTTACAAGTTGGCAGGGCATCTACAAAATTTTGGTTTCTGTAGTTGATGTATGCAAGTCAACCTCAGCTAACAAACACAAGATAGGGTAATCGGCCACCAAGTGCTAAACGCCCCGACAGCCGTGATTGGAGCGCATACTGGCCGTTGATGCTATCATTTGTGTAATTCTCACACTGCCGACTTATGTTACCAGCAGATTTATCAGTTACTGCAGAAACAAAATAGAGTTTGTACCTTCCAGCTGACGCTTGTGTGCCCCGACATGCATGCAAATGAACGTACCTAGCAATGAGCATAAAATGCACGTATGGTCGGGTGCAATGATAACGTGCGTCGTAGTGGCTAGTCTTGAAGACCGATAGTAGCACAATCATCACGAACTTGAAGAATGAGAGTATCGAACCCTGTTGCATCTCTCAGCTCATATGGATGCGATCAGCTACCCCAGAACCCGATGGTTCAC	91%
scaffold175	**Hd26**	5018	10942034-10947051	Hd26_DRJ1L	592	10942034-10942625	ATCACGGTTAGGCATGTCGATTATTGCTGTATTGCGGTTATTGTGGTTGATGAATCAACGATCAATGTCGAAACATGTGTGACGAAACTCCTGACAAGGTAAAGTGCTGACTGTTGCGGTATCAATAAGTTACAAGTTGGCAGGGCATCTACAAAATTTTGGTTTCTGTAGTTGATATATGCAAGTCAACCTCAGCTAACAAACACAAGATAGGGTAATCGGCCACCAAGTGCTAAACGCCCCGACAGCCGTGATCGGAGCGCATACTGGCCGTCGATGCTATCATTTGTGCAATTCTCATACTGCCGACTTGGGTTACCAGCAGATTTATCAGTTACTGCAGAAACAAGATTGAGTTTGTACCTTCCAGCTGACGTTCGTGTGTCCCAACATGCATGCAAATGAACGTGCCGAGCAATGAGCTTTAAATGCACGTTTGGTCGGTTGCATTAATAACGTACGTCGTCGTGGCCAGTCTTGAAGACCGATCGTGGCACAATCATCACGAACTTGTAGAATAGGTGGACCAAACCGTGCTGTATCCGTCAGCGCATGCGGATGCGATCAGCTACCCCAGAACCCGATGGTACAC	
scaffold175	**Hd26**	5018	10942034-10947051	Hd26_DRJ2R	208	10946844-10947051	AGCAAATCTGTAAACGCTGTCTTTGCTCAGTCGTGACGCTTGGCATTATTTATGACTCAAAAAGACCTGCAACAAGTAATGACGTCGCGATATGGAGAATGACAAAATGCCGATGAGACAAGGATCGACCCAGCTAATGCATACTTCGTTTATGCGCTAACTATGAGTGTAGCAATTTTCCTTCTCGGGACAAGAGAAAGAGTAGTGT	80%
scaffold175	**Hd26**	5018	10942034-10947051	Hd26_DRJ2L	208	10942843-10943050	AGCAAATCTGTAGAAGCTATCATTGTTTAGTCGTGTCGCCTCACATAATTCATAACTCAAAAAGACTTGCAACAAGTAATGGCGTTGCGATATGGAGAATGACAAAACGCCGTTAACACGGGGACCGATCTGACTAATACGTACTTCGTTTATGTGCTCACTATGAGTGTAGCAATTATCCGAATAGGCAAGAGAGAGGCAGTAGAG	
scaffold64	**Hd14**	5196	36336-41531	Hd14_DRJ1R	78	41454-41531	CGTGCTGCGTATCCACTGTTCCACGTCAAACGACCTTCGCAGCAAGCCTGACTAGTGCCAAGTATCTACCGCGTCAGC	85%
scaffold64	**Hd14**	5196	36336-41531	Hd14_DRJ1L	78	36336-36413	CGTGCGTCGTATCAACTGTTCCACGTAAAACGGCCGTCGTAGCAAGCCAGACCAGTGCCAAGCATCTACCTTGTCAGC	
scaffold128246	**Hd49**	5265	677866-683130	Hd49_DRJ1R	107	683024-683130	CGCGCAGACAACTTAAAACCATGGTTCGTCGATAATGTCGTCGTACACGTAATGCAGGTTCAACTCGTGATACGCGAACGTCCCCAATACGAAGCCGTTCCCG	83%
scaffold128246	**Hd49**	5265	677866-683130	Hd49_DRJ1L	104	677866-677969	CGCGCAGAGACCTTAAAACCATCGTTCGCCGTTTATATCGTCGTACACGTAGTGCAGGTTGAGCCCGTGATAAGCGAACGTCAGCAATGCGAAGCCGCCGTT	

scaffold128241	**Hd13**	5757	402487-408243	Hd13_DRJ1R	673	407571-408243	TCGTAAACGGGCAGCCTGTTCAGATGTTATGGTCGACCATGGGTCCTGAGGCTTATGTACCACATCGCTTAGACACTTTGGGACTCTGGAAGTTTTTTCTCTACAAGAAGTTGACACCCGTGTGCAAAGTACCCAAGTGCATCCGCTGAAAAGCTAACGATTCATCGAACCGTCTTACGGGAACACACGTCACCACTGACGTGTACTAGAGGTATAGGATCGGACGGCCATCGAGAGTACATGGCAAAGCGTCGCTAACGTCTCTGCGAAAGTACGCTACCATGGTGTAGTAGTGTCTATCCGCGTCATGAAAAGTTTTCCCGACGAACCTATGAAAGTCGCCGACATACTCTCTCGTGAGCCAAAGCCACTGTCGACCCGCTCGTGTGAAAAATATGTTTTTTTCTTTAACAACAACACCCTCGTGACACATCGTTTCATTGTACGACGATGAGCACGATGGTCATGTTGAATGGTCACGTACGCAGCGATGGTGTGACGACAACACTGAATCACGCTTACTGTACATCGATTACGTTTCAGAAAAAATGCATCCACGCGAACTTGAAATGTAGATGTATGACAACGTCAGTTGAAGTGACCGATATGCATTTAGCTTCTTTTACCATCCCACACGGTCAGCTCAAAATATACACGTGTATCCCCTATGATA	92%		
scaffold128241	**Hd13**	5757	402487-408243	Hd13_DRJ1L	675	402487-403161	TCGTAAGCGGACAGCCTGTTCAGATGTTATGGTCGACCATGGGTCCTGAGGCTTATGTACCACATCGCTTAGACACTTTGGGACTCTGGAAGTTTTTTCTCTACAAGAAGTTGACACCCGTGTGCAAAGTACCCAAGTGCATCCGCTGAAAAGCTAACGATTCATCGAACCGTCTTACGGGAACACACGTCACCACTGACGTATACTAGAGGTTAAAATCGGACGGCAAGCGAAAGTGCATGGCAAAGCGTCACTAACGTCTCTGCGACAGTGCGCTACCATGGTGTAGTAGTGTCGATCCACGTCAAGCAAAGTTTTCCCGACGAACCTATGAAAATCGCCGACATACTCTCTCGTGAACCAAAGCCACTGTCGACCTGCTCGCGTGAAAAATATGTTTTTCTTGAACAACACCACTCTCCTCACACGTCGTTTCATTGTACGGCGATAAGCACGATGGTCAACGTCGAATGGTCACGTACGTCGCAATGGTGTGACGACAACACTGAATCACGCTTACTGTACATCGATTGCGTATCAGAAAAAATGCCTGCACCCGAGATTGAAGTGCAGATATATTACAACGTCTGCAGTTGTAGTGACCGACATGCATTTAGCTTCTTTTACAATCCCACACGGTCAGCTTAAAATATTCTCGTGTATCCCCTATGATA			
scaffold357	**Hd50**	5787	2218373-2224159	Hd50_DRJ1R	902	2223258-2224159	CTTGCTGTGTTGATGAGGTACCATACCCGTGCCGGGGGCAAACACGTAAGCATCCAGCGTGGCGTTGAACCAGCTCTTGCAATAAGTATTCAACGGTTACGCAGGCTACTGTAAGAACTTTCAGATTTTAACATGTACGATTAGCGCATGCAATGAAGGATGACTCGACATCAGTTCTTTCAACGATTCTTTCATGAGATTGCATGGTGCAGTACTCCTATCGATCCGTTGAAAGAGCTAGTTATTAGTGTTATCTATATGATATGCCGAGTGTACTACGGCCCCCTGAACATGAAAACTCTAGTACATTCTTCCAATGGTAGAGGTACGAAGCGTTTTTCACATGAAGCATTAATACGTTCCAAACGTCGATGCAGTCTGGAGCTGAACGGTATTCGTCAGACGAGCACCAACAATCCTCGCCATAGCATGGCATCAATAATGCATGAGCGTAAGCAGAGAGGTTGCGACTGTGCTACAACAATGTAAGTATAAGTTCTATCTTACGGAACTTGTCAACGAGTAACAAGTCCTAATAACTCACACATCCAGAGCCAACTATGATCATTGACTCTGAGGATACTGTAAGGCTTGTCGAACTGTGCGAGAAGAATCTTGAAACCAAATGCAGGGCGGGTCCTTAGGAAATCATGAAAGCCCGCTTGTAGACTCGTAAATGGTTTTCAGAAAAAGTCTTTCATGATGAATCTGCACAATTTGAATGATGTCATTGCGGTTAAACGTAAATATCATTACGGCTATTTTTGCAATGTTTTTAACCGTAATATAATCCCATACCTACATCCATGACAAGCGGTATTGTTATAGAACTCCGCTTTTTCCTTGTTGCTTATCATGATTCAAATCAGTACTAACTCTATGATAGTGCACACATAGCGTTC	95%		
scaffold357	**Hd50**	5787	2218373-2224159	Hd50_DRJ1L	890	2218373-2219262	CTTGCTGTGTTGATGAGGTACCATACCTGTGCCGGGGACAAACGCGTAAGCATCCAGCGTGGCGTTAAACCAGCTCTTGCAATAAGTATTCAACGGTTACGCAGGCTACTGTAAGAACTTTCAGTTTTTAACATGTACGACTAGCGCATGCAATGAAGGATGACTCGACATCCGTTCTTTCATGAGATTGCATGGTGCAGTACTCCCATCGATCCGTTGAAAGAGCTAGTTATTAGTGTTATCTATATGATATGCAGAGTGTACTACGGCCCCCTGAACATGAAAACTCTAGTACATTCTTCCAATGGTAGAGGTACGAAGCGTTTTTCACATGAAGCAGTAATATGTTCCAAACGTCGATGCAGTCTGGAGCTGAACGGTATTCGTCAGACGAGCACCAACAATCCTTGCCATAGCATGGCATCAATAATGCATGAGCGTAAGCAGAGAGATTGCGACTGTGCTACAACAATGTGAGTATAAGTTCTATCTTATGGAACTTGTTAACGAGTAACAAGTCCTAATAACACACATATAGAGAGCCAACTATGATCATTGACTCTGAGGATACTGTAAGGCTTGTCGAACTGTGCAAGAAGAATCTTGAAACCAAATGCAGGGGGGGTCCTCAGAAAATCATGAAAGCCCGCTTGTAGACTCGTAAATGGTTTTCAGAAAAAGTCTTTCATGATGAATCTGCACAATTTGAATGATGTCATTGCGGTTAAACGTAAATACCATTACGGCTATTTTTGCAATGTTTTTAACCACAATATAATCTGATACCTACATCCATGACAAGCGGTATTGTTATAGAACTCCGCTTTTTCCTTGTTGCTTATCATGATCTAAATCAGGACTAACTCTATGATGGTACACCCATAGCGTTC			
scaffold59	**Hd12**	5902	674917-680818	Hd12_DRJ1R/L	411	680408-680818	ATAGAACGCTTATCACCACCAATCTTTGGCGTCATACTGTTTGCGGCTTATCTGCAGTTGGTACTTATGGATCAAGTTGAGGAAAACCTTATCCAGGCGTCTCATACCCTTGTATGGTCTGCTCCGTAACTGAACATCGCAGGTACACATACTCAGAGAGTAATCCACACTCAGACCTTACTCAAGACATATGATGGGCATATTCATAAGCATAGTTTGTGATATATAACGGAGCTTTGCATCCCATGTGCACATAACTCTAACAGAGATAAGTAGGGAGATCTCCAATGCTATAGGGCTCGAGTGGTTCCTACAGAGCGGCGCTTTTTCTTATCGGTCAACTAATAAAGCTGTCTTAGATGTTTTTTTTGCTCGGTCGTGCTCGCACAAATCCATCCTGCTCGGCAGAGA	84%		
scaffold59	**Hd12**	5902	674917-680818	Hd12_DRJ1R/L	412	674917-675328	ATAGTACGATAATTGCCGCCAGTCTTTGGCGTACTACTGTTTACGGCTTATCTGGAGTTGTCACTCATGAGTCAAGTTAAGAAAAACCTCATTTAGATGTCTCATATTCCTGTATGGTCTGCTCCGTACCTAAACGTCGCGGGTACACGTACTCAGAAAGGAATGCACACCCAGGCCTTGCTCAAGACATATGATGAGCATATTCGTAAGCATAGTTTGCGATATACAACGGAGCTTTGCATCCCATGTGCACATAACTCCATCAGAGATGAACGGGGAGATCTCCAATGCTATAAGGCCCTAGTGGTTCCTACAAAGCGACGGTTTTACGTATCGGTCAACTGCTCCAGCTGTCATAGGTGTTTGTTGTTGCTCGGTCGTGCTTGCAGTAATCCATCCTGCTCGGCAGAGA			
scaffold59	**Hd10**	6507	2500377-2506883	Hd10_DRJ1R	251	2500584-2500834	AAAATTACGACCAAGGTGGATGCCGTGATGATGTAGTGGCATAGTGAAGGTGTAGCGCCTGCTAAGCCCAACAACTGTATACCCTGGACTACAATCCGTCACGGCGCTTGGAGCATACCCCAGTAACAACGTGACTTATGTGGTAAAAAGCAGATTTCAGTACCAGAGCAAATACCTGTGCTATCTGTATGCACCAGATTTACAATCTGTTAGCGTCAGTGTCTACACAATGATGTGGCATACGATAGATG	78%	84%	70%
scaffold59	**Hd10**	6507	2500377-2506883	Hd10_DRJ1int	234	2503546-2503779	AAAGTTACGACGAAGGTGGATGCCGTGATGATGTAGTGGCATAGCAAAGGTGTAGCGGCTGCTGGGACCAACTACTGTGTACCCTGTGATACAATCCGTCACGGTGCTTCGAGTATGGTCTCGAGCACAGTCGTTCCTACTTCCATGTCAGAACAAATACCTGTGCTACCTGTGCACCAGATTTTCAATCTGGTAGCGTCAGTGTCTACACAATGGTGTGGCATACGACAGGTG			
scaffold59	**Hd10**	6507	2500377-2506883	Hd10_DRJ1L	288	2506596-2506883	AAAATTACGACCAAGGTGGATGCCGTGATGATGTAGCGGCATAGTGAAGGTGTAGCACCTGCTAAGCCCAACAACTGTATACCCTGGACTACAATCCGTCACGGCGCTTGGAGCATACCCCAGTAGCAACGTGACCTATGTGGTAAAAAACAGATTTCAGTTACCACTGGACTTGAGCGGAGTTGTTCTTACTTTCATGCCAGAGCAAATACCTGTGCTATCTGTATGCACCAGATTTACAATCTGTTAGCGTCAGAGTCGACACAATGATGTGGCATACGATAGGTG			
scaffold59	**Hd10**	6507	2500377-2506883	Hd10_DRJ2R	206	2500377-2500582	CGTTGCTGTGCCGTAGTGGACATAGCTTTGCAGCAGCCTCGATGAGGTCATGATCATCTGCATGCGGCTCTCCAAAACTGCAGTGCTAAAAGTGGTTCGCAAAAGAATGTGACAACATACCGTTCCCTCTAGATTATATTCTGTCATTGCGAACAATAAGTGTGAACAAGAACTTAGTTAGATGTCTCAGGTTCTTTTGCGGCAAC	87%		
scaffold59	**Hd10**	6507	2500377-2506883	Hd10_DRJ2L	205	2506390-2506594	CGCTGCGATGCTGTAGTAGCCGTAGCGTTGCAGTTGCATTGACGAGGTTCATATCGTCTGCATGCGGCTCTCCAAAACTGCAGTGCTAAAGGTGGTTCGCAAAAAAATGTGACAACATTCCGTTCCCTCTAGATTATCTTCTGTCATTGCGAACAGTAAGTGTGAACTAGAACTTATTAGATGTCTCAGGTTATTTCACGGCGAC			
scaffold144	**Hd20**	6864	1449468-1456331	Hd20_DRJ1R	215	1451905-1452119	GAACGTGTTCCCGCATTAAAGGAGTTTCAGGGAAAAATAAATTTACTCTGAGTCGGTAATTCAACGTTTCACAATGTTGGACCGGGCGAGATGCATTCAGCTGCTTCTGCCTTGGATTCGATGCTGGAATCCTGTGACCACGTCGTCGTCAATCGGAGAACTGCATTGCTCACAGTGCTATCGACCTATTCGCTGTGGCTACCGTA	84%		
scaffold144	**Hd20**	6864	1449468-1456331	Hd20_DRJ1L	214	1456118-1456331	GAACAGTGTTCCCCCATTGATGGGGTTTCAGGGTAATGAATGTACTCTGAGTCGGTATGTCAATGTTTCACAATGTTGGACCGGGCGTGATGCATTCAGCTACTTCTGCATCGGATTTGATGCTGCGGCATCATGTGAACACGTCGTTAATTGTAGAACTGCATTTGCTCACAGTCCCATTGACCTATTCGCTATGGCTGCCATAAA			
scaffold144	**Hd20**	6864	1449468-1456331	Hd20_DRJ2R	308	1449468-1449775	GAATAGACCGTTAAGAATGACCGAAGCCGCAATGAATGTGCGCTTCTTGATGGTGCCCTTTCAGGGAGATTTAGAGCAATTTCTATAGTAGCTTTCCACAACTTTGACATAACATAAAATGTTTACATCGATACTTAACACGTGCCCATGCGCTCGCCAGAAAGAGTCGTATCTCCGCTGCAAGCGACAGATATGCAGGTTCGCATTGCAGTCGCAGCCGCGGGCATGACCCTAGCGAGATCCTGAACAGAAATCATACTTGGTCTGACATTCTTATACCAGCACTCCACCGAGATTGCAGCTTCCCA	86%		
scaffold144	**Hd20**	6864	1449468-1456331	Hd20_DRJ2L	300	1453262-1453561	GAATAGGACCGTTAGGTATGACCGAAGCCGCAATGAACGTGCGCTCCTTGATGGTGCCCCTTCAGGAAGGTTTGGAGTAATTTCTATAGTAGATTTTCACAACTTTGACATAACATAAAATGTTTACATCGATACTTAACACGTGTCCATGCGCTCGCCAGAAAGAGTCGTATCTCTGACAGATCTGCAGGTTCGCGTTGCAGTCTCAACCGTGGGCATGACCCTAGCGAGATCCTAGGTATGAAACATACTTGACCTAACATTCTTATACTAGAACGCCACCAATATTGCGGCTTCCCA			
scaffold377	**Hd8**	7356	2186417-2193772	Hd8_DRJ1R	719	2189916-2190634	AAAGCAACAGCGATTGCATTTCAAATGAAGATAAGTACATGCAATGATAAATATCACAAGGTCACGTAACTCATAGGGTATTGGGTAGTTTTTCGAGATACGCAGCGCTTCATAAAGCACGTTCAACTTGGACGTCCAAAGCGTTGATAGCGTAACTTAGCGTTCGACTTGGAGCTGTAAACGCTGACGAGCGTTCAGACGGATGCGTCCACGGCACGTGCAGTCCAAGTCGAATGCGTTCGACAAACGCTTCCAACGCTTTCAACGTTGTGAGCGTCCAAGTTGAACGTGCCGATAAGCTGATGGATATAACTACGAGGATGCTGGTGATGATATCAGGAGAAGCAGAGGCAGCTCTCACGAATGACTACTTGCTGTGTTCGAGCAGCTTTTTCTATTCTATATGCAATTTCGTTGGCTCCGGTCAACACTCTAAAGTGATGTGGCCATACGGCTATGAGATGGATGCGCTTGACGACAGTGTCAAAGGTCAACGCCCTCGAGTAGCACATGGTCCAAAAATTCGTCTCATTCATTCGGACGCTAATTAAAGTGAGACAATGAATCCTGTTAATATATTCATGCACATGATCCAACGTACACTGAAAAACCTGGTGTGTTTGAACGATAGCGTAGACTTGTCCATCATGCTGTTGTTTTTCCAATCATTGCATCCGGCACTGCAGCAGGAATGCGTGCACCCTGTTGCCCCTTTTTTC	95%		

scaffold377	**Hd8**	7356	2186417-2193772	Hd8_DRJ1L	720	2186417-2187136	AAAGCAACAGCGATTGCATTCGGAATGAAAACAAGTACATGCAATGATAAATATCACAAGGTCACGTAACTCATAGGGTATTGGGTAGTTCTTCGAATTACGCATCGCTTCATAAGGCACGTCCAACTTGGACGTCCAAAGCGTTGAAAAGCATTACTTAGCGTTCGACTTGGAGCTGTAAACGCTGACGAGCGTTCAGACGGATGCGTCTACGGCACGTGTAGTCCAAGTCGAATGCGTTCGACAAACGCTTCCAACGCTTTCAACGTTGTGAGCGTCCAAGTTGAACGTGCCGATAAGCTGATGGATATAACTATGAGGATGCTGGTGATGATATCAGGAGAAGCAGAGGCAGCTCTCACGAATGACCGCTTGCTGTGTTCGAGTAGCTTTTTCTATTCTATATGCAATTTCGATGGCTCCGGTCAACACTCTGAAGTGATGTGGCCATACGGCTATGAGATGGATGCGCTTGACGACAGCGTCAGAGGTCAACGCCCTCGAGTAGCACATGGTCCAAACATTCGTCTCATTCATTCGAACGCTAATTAATGTCAGTCAATGAATCCTGTTAATATATTCATGCACGTGATCCAACGTACACTGAAAAACCTGGTGTGTTTGAACGATAGGGTAGACTTGTCCATCGTGCTGTTGCTTTACCAATCATTGCATCCGGCACTGCAGCAGGAATGCGTGCACCCTGCTGCGCTTTTTTC	[illegible]
scaffold377	**Hd8**	7356	2186417-2193772	Hd8_DRJ2R	292	2193481-2193772	ATAACGTGTTATGGCGACGTTTTACCAAATCCAAGAAACATTGCTTAACTATCAGTAATTACCAGACACCAGTAACTACTCTAAAAGCAACAGCGATTGTATTCGGAATGAAAATAAGTACATGCAATGATAAATATCACAAGGTCACGTAACTCATAGGGTATTGGGTAGTTTTTCGAAATACGCACCGCTTCATAAAGCACGTTCAACTTGGACGTCCAAAGCGTTGAAAAGCGTTACTTAGCGTTCGACTTGGAGCTGTAAACGCTGACGAGCATTCAGATGGATGCGT	91%
scaffold377	**Hd8**	7356	2186417-2193772	Hd8_DRJ2L	291	2189833-2190123	ATGACATGTTATGGCGGTGTTTTATCAAATCCAAGACACATTGCTCAGTATCAGTAATTATCAGACTACCAGGAGCTACACTAAAAGCAACAGCGATTGCATTTCAAATGAAGATAAGTACATGCAATGATAAATATCACAAGGTCACGTAACTCATAGGGTATTGGGTAGTTTTTCGAGATACGCAGCGCTTCATAAAGCACGTTCAACTTGGACGTCCAAAGCGTTGATAGCGTAACTTAGCGTTCGACTTGGAGCTGTAAACGCTGACGAGCGTTCAGACGGATGCGT	
scaffold59	**Hd16**	7704	690205-697908	Hd16_DRJ1R	65	694965-695029	TTGTCATGAGCGTAACTACATTTAAACTTGAAGCACTGTAAGAAGGAACGGTCTCACAGTACCTT	85%
scaffold59	**Hd16**	7704	690205-697908	Hd16_DRJ1L	65	690205-690269	TTGTCATGAGAGGAACTAAATTTAAACTTGTACCAACGTAAGAGGAAACGCTCTCACAGTACCTT	
scaffold59	**Hd16**	7704	690205-697908	Hd16_DRJ2R	172	697723-697908	TAGATTATGCAAACGTGGCAAAGCGGTGTATGGCTTACGAGATGCGGCTGACCGCTGCATGTCAACGATAAGCAGATCATTCTACAAGAAGTATGTTTGTTCTAGGCGTTCTAAGTGTCGTTCTATGATAACGTGAGCTGTAGGTTTCTGATCATTCGGCCCCAGCTTGGAT	94%
scaffold59	**Hd16**	7704	690205-697908	Hd16_DRJ2L	172	691583-691754	TACATTATGCAAAAGTGGCGAAACGGTGTATGGCCTACGAGATGCGGGTGACCGCTGCATGTCAACGATAAGCAGATAGTTCTACAAGAAGTATGTTTGTTTTAGACGTTCTAAGCGTCGTTCTATGATAACGTGAGCTGTAGGTTTCTGATCATTCGGCCCCAGCTTGGAT	
scaffold351	**Hd17**	7730	2329273-2337002	Hd17_DRJ1R	70	2332468-2332537	GTGTTGCGAGCAATAATGGTGGAAACACCTGTCATGGCACGCGTCCACTGCAGGCTGTACAAACGCTCTA	77%
scaffold351	**Hd17**	7730	2329273-2337002	Hd17_DRJ1L	73	2336930-2337002	GTGTTGCTTAGGAATAATGGTGGAAGCATCCGCGTACTTACACGTGTTCACTGGAGGCTGTACGAGCGCTCTA	
scaffold351	**Hd17**	7730	2329273-2337002	Hd17_DRJ2R	161	2329273-2329433	CGTGGAGCGTGGAATGTTGACGAGTCATAGAATTTCGGCTGGCACGAGCACGTGCAAGTTGAAGTTGTACGCCTGCGATGACGAGAAGTTATCGACAGGAGTTGACAACTTTGGCTGCGACTTCCGAACGCCAGATGGCCTGATGGTGGCTGTCCCGTAAG	79%
scaffold351	**Hd17**	7730	2329273-2337002	Hd17_DRJ2L	161	2333675-2333835	CGTGCATCATGGCATCTTCACGAGTCATAGACATTCGGCAGGCACGAGCGAGAACAAGTTGAAGTTGTAGGCGTGCACAGACAAGGAGTTATAGACGGGCGTCGACAACTTCGGCTGTGACTTTTGAATGCCAGATTGCCTGATGGTAGCTGACCAATAAG	
scaffold64	**Hd32**	7916	88702-96617	Hd32_DRJ1R	613	92195-92807	AGGCCTGACCTCAAGCCCGATAACCTACCGCTTATTTACCGAGTACCTACGAGAGTCGCTCAAAAACTGTTTATACATAAAGTTTCTACTGCATGCTTGAGGCCTATCTACAAATACTGCAATGTAGTTGAAAGTGCAATCCATCGCAATGCTTACTTTAGCACCTTATTATGTAAGAATTCTGCATTTGCGATACTGCTTGCACTTTACAGTGAGTGCATGAACGATTCTCCATGGTTAACCACCCACGGTACTTCATTTCATGTTTTCGCCATGACGTTGGAGATGCTCTGCTCGAACGCTAATCATGGTGACAATGCAATAAGCTGGGTGGAATGCTGACGTTAAGAATGAAATCATTACCGTTGATAGGAATACCTAAATATGCACATTTTCCTGTTCATTCAATTAGTCCCACCGCAAATAATGTTCTTTACCTACATACATTAGGTATTGTAATAATACATTGTCGCGATCTTATGCCGATGCATACTACCTGACAAGACGCTTCGCTCTGTACGTAAGGCGTCAGCTACATCTACAACGAGCGCTCTGTGGGTTGACTATCTTAAATCACTCGTCCTCGCCGCAAATATCAAGCCGTAACAAGTCT	91%
scaffold64	**Hd32**	7916	88702-96617	Hd32_DRJ1L	626	95992-96617	AGGGCTGACTTCATGCCGAATAACCTACTGCTCATTCACTGAGTACCTACGGGAGTCGCTCAAAAACTGCAATACATATGGCTTCTACTGCATGTGCATGCTTGACGCCGATCTACAGCCCAAAATACTGCAATGTAGTTGAAAGTGCAATCCATCGCATAATGCTTACTTTAGCACCTCGTTATGTAAGAACTCTGCATTCACGATACTACTTGCACGTTTCAGTAAGTGCATGAAAGATTCTCCGTGGTTAACCACCCACGGTACGTCATTTCATGTTTACGCCATGACGTTGGAGATGCTCTGCTCGAACGCTAATCATGGTGACAATGCAATAAGCTGGGTGGAATGCTGACGTTAAGAATGAAATCATTACCGTTGACAGGAATACCTAAATATGCACATTTTCCTGTTTATTCAATTAGTCCCAGCGCAAATAATGTTGTTTACCTACATACATTAGGTATTGTAATAATACATTGTCGCGATCTTATGCCGATGCATACTACTTGACAAGACGCTTCGCTCTGTACGTAAGGCGTCAGCTATATCTACAACGAGCGCTCTGTGGGTTGACTATCTTAAATCACTCGCCCTCGCGGCAAATCTCAAGCCGTAACAAATCT	
scaffold64	**Hd32**	7916	88702-96617	Hd32_DRJ2R	757	88702-89458	TGAAGGAGATGATGAAGGAGCTGTGGGGCTGCAAACAAAGCGAGCGTCAGGGAGGAACCTGGAAGTAATGGAAGTGAAGTGTTGAACGAAAACCGCCGCACCAGTTATGTACGCAGCTGTCCCAACTAGCATGTAAGGTTTCAACTCTGGCTGTGTTCAGCGGTTGTGTGTATTAAGTGGTGATAGTGTTGTAAGCTTCTAAAATAAATAATTCGACTCCATTCGTATTTCTTTCCTTTAGAGACCGTGTGAGCTCGGCAAAATCTGCTCAGCTCACATGCATCTTTTCCCAGCAGTAGGACAAACATTTTCTTCAACTTGATCCTCTTGAGGCAGGCACTCATACCGAGGACAGACATACACACTTCTATTACTTCCAAGAAGCTCCTCCAATAGTTGGCATAGTCCGAGCTTGTACGGTGTCCACCCAAACAGTATAGCTCAAGAGGTATATGCATTCAACACTTAACACTACTTATTTCTACACCTATGCTAGGAATAATCATTTGTTTTATCTTACTGCTATCTTCAGGGCTACAGGTGCTAACGACAGTTCGTGCCAGCATCAGCTCACCTGAGTTTATGCTTATAACAGCTGCAGCACCCAAGGCTTTGCATTTCGTGTAGATAACACTTATCATTATTTACAGCTAAGTCTAGCTTCGCATATCTTGCCGTTGGGGACTTGGTGCAAGAGCTGGTCAAATGCAGGATACATGTTCACATGTTCATTGGTTAAGGGTACAGTTGACCATCT	90%
scaffold64	**Hd32**	7916	88702-96617	Hd32_DRJ2L	750	93300-94049	TGAAGGAGATGGTGAAGGAGCTGTGGGGCCGCAAACAAAGCGAGCGTCAGGGAGGAACCTGGAAGTAATGGAAGTGAAGTGTTGAAAGAAAACCGCTGCACCAGTTATGTACGCAGCTATCCCAACTAGCATGTAAGGTTTCAACTCTGGCTGTGTTCAGCGGTTGTGTGTATTAAGTGGTGATATTGTTGTAAGCTTCTAAAATAAATAATTTGACTCCATTCATGTTTCTTTCCTTTAGAGACCGTGTGAGCTCGGCAAAATCTGCTCAGCTCACATGCATCTTTTTCCAGCAGTAGGAAAAACATTTTCTTCAACTTGATCCTCTTTAGGCAGGCACTTATACCGAGGACAGACATACACACTTCTATTACTTCCAAGAAGCTTCTCCAATAGTTGGCATGGTCCGCGCTTGTACGGTGTCCATCCAAACAGTATAGCTCAAGAGGTATATGCATTCAACACTTAACACTACTTATTTCTACACCTATGCTAGGAATAATCATTTGTTTTATCTTACTGCTATCTTCAAGGCTACTGACGCTAACGACGGTTCGTGCCAACATCAGCTTACCTAAGTACACTTATGACACTGCAACACAATCATTTGCAATGCGTGTAGATAGCACTTACTACTACAAATAAGTCTAGCTTGGTAAATGTTGCTATTGCAGACTTGGTGCAAGAGCTGGTTATGCAGCACACGACGGGACGCATGCTCGCATTCGTTAAGGGTACGATTGAGCCTCT	
scaffold184	**Hd41**	7953	3768924-3776876	Hd41_DRJ1R	551	3768924-3769474	AAGTATAACTCATAGGATGTTTGGCAATTCTCTGCATAAGCGCGGCTTTTTATTTCAAACAAGACGATGACTCTCGCATTTACAGTGACGTACTAAGGGGCGTGGGAAACTTGTGTCGGCTGGGTATGCCGGGTCGTATACTAAGTTTCCCACAACGCACGTAAACCAAAAAACTTCACTTAGACACAAACTATAGGAACGGACCGAACGTTTTCTCACTAACTGCACGTAACGTGAATGCTGCCATGTAATGCATTGCCGATCGCTGTCAAGCACCATTACTCACGTCAGCTTCGTCGCGATGATATCGTTATCACCGTGAGAACTGGTACTAAAGACTATTCAAAAGCAACGTCTGAGAGAGCACGCAGGATTTTCGAAGACCAACAAAGGTAAAACCGTCGCGTCAGAGTTGCATGGAGACTCGCAAACTGATCACAAACGACGTCTACCTTTCACACTCGTGAAAATGTTACATTGAGCTGTCAGTGGTACTTCATTCCACGCCGGCGAGAGTCCCTTTTGGTAGAAAAATATGCACGCATTTCTGT	85%
scaffold184	**Hd41**	7953	3768924-3776876	Hd41_DRJ1L	540	3771924-3772463	AAGTGTAACTTATAGGATGTTAGGCAATTCTCTGAATGAGCGCGGCTTTTCATATCAAACAATATGCTACCGTATTTATAGCGACACTAAGAGGCGTGGGAAACTTGTGTCGGCTGGGTATGCCGGGTCGTATCCAAGTTTCCCCCGACGCACGTAAACCAAAAAACTTCACTTGGACACAAACTATAGGAACACACCTAACGTTTTCTCACTAACTGCACGTAACGTGAATGCTGCCAGGTAATGCATTGCCGATCGCTGTCAAGCACCATTACTCACGTCAGCTTCGTCCCGATGATATCGTTATCACCGTGAGAACTGGTACTGAAGACTATTCAGAAGCGGTGTGTGAGAGCAGGCCAGATTTTCAAAGATGAACAAGGGTAGATCCGTCGTGTCATAGTTACATAGAGCAACTTTGCAACTGATCACAAGCGACGTCCACGTGTTAAAGTCGTGAAAATATTAAATTGAGTTGTCTGTACTATTTAATTCCATATCGGCGAGTGTCTAGGTAGGAAAATATGCGAGGATTTCTGT	
scaffold184	**Hd41**	7953	3768924-3776876	Hd41_DRJ2R	305	3771631-3771935	AACTTGTAGCATCTAGTAGACTCAAAATACCAATCATGTCTGAGAGATCTATGTATCCGGTGGTGGCGTAGTTATCTGAGAAGCTAACAAAGCTCGTGCAGTATGAGGGGAACAATCATGCCGTCACCAGGCGAACCATGTGTATCACGTAGATAAGCTAGTCAGAGTAATGAACCAGCTATACATGTTACTTGCTCCCAACGATTACCAGAACGCCGGTAGTTCTACAGCAACCAAAAGAATTTGCTTTGCGATCCAAAGTAGGTCTATCCACCTATAGCTTTTACAAGCTGAAGTGTAACTTA	81%
scaffold184	**Hd41**	7953	3768924-3776876	Hd41_DRJ2L	306	3776571-3776876	AACTTGTAGCATCTGGTAGACTCGAAATAGCAATCATGTCTAAAGGATATATGTACCCGGTGGTGGCGTAGTTATCTGGGAAACTCACAAAGCTCGTGCAGCATGAGGAGAACCATCGTGCCGTCACCAGGCAAAGCAGCACGTATCACGTAAACAAGCTGCTCAGAGACATGAACGAGCTATGCATGCTATTGGCTACCAACGGTTACCAGACCGCCAGCAGTTCCGCAGCAAACAAAGACATTTGCTTTCCGACTTCAAGTGGGCTTATCCACCGATAGGTATCACAAGGTAAAGTATAAGTTA	
scaffold127548	**Hd7**	8066	6062921-6070986	Hd7_DRJ1R	362	6062921-6063280	GATTTCGATTCCCTTCCGATCTCGTTTCACCTATCCTCGTCACTCTTACTCGACACCGACCAGAAACAACCCATTGGGTATATTGGTGGAACGAAGCGTGATTTACATCGAAATCTCCTGCGCAGCATTACGACCGGTTTATTGGTAAAACGATACGAAGTGAGAACGGAAGTGATCAAAATAAAAAGATGAAGGATTTATCGATTTGTTGCCTGACCATTTTCCTCAACGTTCAAATCCCTCCTTCTAGTCAGTTTCTTTTTATCGTGTAACCGAAACATCGCTTGGACAGCTGAATTTTGTTTCGTTGCATCTTTGGTGATATCTTTTTTTTGACGTCCTCGCGTGTATACTCGAGCC	79%
scaffold127548	**Hd7**	8066	6062921-6070986	Hd7_DRJ1L	366	6070623-6070986	GATTTCGATATCCTTCCCATTTTGTTTTACCTGTCGCGTCGTAACTCTTATTCGACACTCACCAGAAACACCCATTGGCAGGGTATATTGTTGGAACGAGGCGTCACTTACACCGAAATCTTCTACGCAGCGCCACGACCGATTTACTGGTAAAACGATGTGAAGATAGAACGAGAGTGATGAAAGGGAAAAGATGAAGGATTTTATCGATTTGTCACTTCACAAATTTCCTTACCGTTCGAGCCGCTCTTTCTAATCAGTTTCTTTATTGTATAACCGAAACATCACTTAGGCAGCTGAATTTTGTTTCGTTTAATCTTTTATCACATGTCTTTTCAGACGTCCACGCGTGTATGCTCCAGCC	

scaffold59	**Hd11**	9190	2183512-2192701	Hd11_DRJ1R	495	2186118-2186612	ACCTACATCTGGGTTTTCAAAACATTCGTCGGACCGACCGAGAGTGTGAGATAATACGTCACTTGAGGACGATATTCTGATGACCTGTCACCTATCACTCATTG<br>TTTTGAGATAATTTGATAAAATGTTGTATAAATACTCATTCCAACAACAAAAAGTCTCTTCTGTCAACATCCTCTGCTGAGATTGTTTCAATTATTTGGCAACAAT<br>GGCGACTTCTCAAATCGACAAATTGTTCGACAAGGATCCTTTCACCGGGAATACAATTTTTCACGAGCTCGCCTACATTGGATCACTGACGCTGCTCAAGCGAT<br>TCCGGAACAACCTCGACGAGCCATGTACCTTCATCCTGCAACAGTTTAACTCCGATGGTGAGTACAGTATCCACGTTGCAGCAAACGTCCACAGAGATCTACA<br>TGCAATTCAAGTGATCGAGCTACTGCGGGATTTGGGAGCAGATCTGGATGCAAGGGACGATCAATTAGGCATTACCGT	97%			
scaffold59	**Hd11**	9190	2183512-2192701	Hd11_DRJ1L	493	2192209-2192701	ACCTCCATGAGTTTCCAAAACATTCGTCGGACCGACCGAGAGTGAGAGATAATACGTCACTTGAGGACGATATTCTGATGACCTGTCACCTATCACTCATTGTT<br>TTGAGATAATTTGATAAAATGTTGTATAAATACTCATTCCAACAACAAAAAGTCTCTTTTGTCAGCATCCTATGCTGAGATTTTTTCAATTATTTGTCAACAATGGC<br>GACTTCTCAAATCGACAAATTGTTCGACAAGGATCCTATCACCGGGAATACAATTTTTCACGAGCTCGCCTACATTGGATCACTGACGCTGCTCAAGCGATTCC<br>GGAACAACCTCGACGAGCCATGTACCTTCATCCTGCAACAGTTTAACTCCGATGGTGAGTTCAGTATCCACGTTGCAGCAAACGTCCACAGAGATCTACATGC<br>AATTCAAGTGATCAAGGTATTGCGGGATTTGGCAGTAGATCCGGATGCAAGAGACGATCAATTAGGCATTACCGT				
scaffold59	**Hd11**	9190	2183512-2192701	Hd11_DRJ2R	95	2183512-2183606	CATCATAAGTCGAAGTAACTGACGGGGTGTTGGACGAATCGCCGGAATACCCACGGCTCAAAATATCGACAAACGTAATTGTGATGGTCGTGATG	80%			
scaffold59	**Hd11**	9190	2183512-2192701	Hd11_DRJ2L	95	2187479-2187575	CATCATAAGTTAAAGTAACTGCTGGGGTGTAGGACGACTTGCCGGAATAAACACGGCAGTGAATGTCGACAAACATAACTTTGATGGTGGTGCTG				
scaffold429	**Hd48**	9673	531833-541505	Hd48_DRJ1R	616	536626-537241	ATTTCTTTATGTTTCAGATTGCACTTGAAACGTGCGGGATACATGCGACCACTTTTTATCCATTCGCGATTACCTACATTTGAATCGTCAACCAATAAGAAGGCT<br>TGATGCAGCCGTATCTCAATATCAAGGTGCCAGAGGTTCGTGACGTTCGTCGCTGTGTGATCATCGAGGGGCTACCAACCGTTGAAGTCGCGACTGCGTACG<br>TGAATGCACGGACGACTCAGAGCAGACCATCAACTCGATCAAGCACAGATCCAAGTGAAAGATTTTACAACTGCTAGAGGGAACCACAAGGAGTCAGGATCG<br>CGAACGAGAAGGATGCTGGATGTTCACAAGTATCAAGTAGAACGAGAAGCCTGATAGCTTAGAGATATGACTTCAAGCACCAGTTGCAACAGCAGGCACGCG<br>CCACGTGCCGTAAAGTCTTTATGTTCTGATGATAACCCCAAGTACGTGATGCAAACCGATTTTATATGCAATCGCTACTTGGATACGGCGGACAACACGGTGTC<br>GTGACGGAGTCTTGGAATCTGTAATTGCAACGCTGAGATTGCATGCTGGTTGCAATGCCAAGAAGCACGAAGTAGCTGCAGCACATGCTTGGACTCGGACA	84%			
scaffold429	**Hd48**	9673	531833-541505	Hd48_DRJ1L	610	531833-532442	ATTTCTTCATGTTTCTGATTGCACACAAAACGTGTGAGACACATGTGCGACAACTTTTTATCCAATCACGATTCCCTACACTTAGTTCGGCAACCAAGAGTGCTT<br>GGGTAGCCGTTTTTCAATATCAAGATGCCAGAGGCTCGTGACGTTCGTCACTGTGTGATCATCGAGGTGCTATCAACCTTTGAAGTCCGTCGCGACTGCGTAC<br>GTGAGTGCACGGACGACTCAGAGCAGACCATCAACTCGATCGAGCACAGATCCAAGTGAAAGATTTTACAATCGCTCGAGGGAACCAGAAGGAGTTGCGATC<br>ATCAACGAGAAGGATGCTGGATTATTACAAGTCTCAAGCAGAACGAGAAGCCGTGATAACTTGGCGAGATAACTTCAGGCGCCAGTTGCAACAGCAGGCACG<br>CGCCATGCGCTGTAAAGTCTGTATGTTCTGCTGGTAACCCCATACGTGGTGCGGACCGATTTTATATGCAATCTCTACTTGGATACTGCCGACAACACGGTTT<br>GGCGCTGGAATCTTGGGCTGTGTGATTGCAACACTTGGATTGGATACTGGTTGTAATGCCAAGAAGCACAGAGTAGATACACCGCATGCTTGGAC				
scaffold429	**Hd48**	9673	531833-541505	Hd48_DRJ2R	205	541301-541505	GTCTTTGATTCTCCAGACCGACCACCAAGACCTACCTACCACTTAAATCGGACCGTCAACTCGATCAAGCACGAGATCGAAGAGAAAGATGACAGTCGCTAAA<br>AAGGAAGTAATAGTAGTGACAATCGTGGAAGAGGAGGATGCTGGGATCTTACGAGTCCCCAAGCAGAGCGAGGGGAAAGTGACAGCGCAGAGATCGTAGCT	91%			
scaffold429	**Hd48**	9673	531833-541505	Hd48_DRJ2L	205	536223-536427	GTCTTTGATTCTCCAGACTGACCTCTGAGACCCACCGACGACTCAAATCGGACCGTCAACTCGATCAAGCACGAGATCGAAGAGAAAGATGACAGTCGCTGA<br>AAAGGAAGTAATAGTAGCTACAATCGTGAAAGAGTAGGATGCTGTAATCTTACGAGCCCCCAAGCAGAGGGAGGGGAAAGTGACAGCGCAGAGAGTGTAGCT				
scaffold65	**Hd3**	10014	437082-447095	Hd3_DRJ1R	156	441738-441893	ACGTCCGCGAGAATGCGGACGATAACGTTCTCGAAAAGTGCATAGTGCAGATACTTTTCAGAGGCGAGCTCAGAGGTAAGAGAACGGTAGTTTAGACAGCGT<br>AAGTGTCATACATGCAAGAAGTCGTTTGGATGCAGGTTTTATGCTATCGCTTCG	76%		93%	
scaffold65	**Hd3**	10014	437082-447095	Hd3_DRJ1int	149	437664-437812	ACGTCTGCGAGAATGCGGATGATAACGTTCTCGCAAAGTGCATAGTCCGGATACTTGGCAGAAGCAAGCCGCGAGGTTCCGTTTGGTCCGCGTAAGTGTCAT<br>ACACGCGACAAGTCGTATCGATGCTAATTTTGTTCAATCGCTTCGTA		80%		
scaffold65	**Hd3**	10014	437082-447095	Hd3_DRJ1L	159	437082-437240	ACGTCTGCAAGAATGCGGACGATAACGTTCTTGAAAAGTGCATAGTGCAGATACTTGGCAGAGGCGAGCTCCGAGGTAAGAGTTACGGTAGTTTGGACAGCG<br>TAAGTGTCATACATGCAAGAAGTCGTATCGATGCAGGTTTTATGCTATCGCTTCGTA				
scaffold65	**Hd3**	10014	437082-447095	Hd3_DRJ2R	75	447021-447095	GAGGGTCGGCGGCGTTATCCGAGAGGACGCTTGCAAACTTGACGAGCAGACGCATCATCGAAGAGCTCAGCCACG	80%		85%	
scaffold65	**Hd3**	10014	437082-447095	Hd3_DRJ2int	77	441661-441737	CTCGCAGGTCGGCGGCGTTAGCCGGAGGGCTCTTGCAGTCATGACGAGTGGACTTACAATCGAAGAACTCAGCCATG		89%		
scaffold65	**Hd3**	10014	437082-447095	Hd3_DRJ2L	77	437587-437663	CTCGAGGGTCGGCGGCGTTAGCCGGAGGACTCTTGCAGAAATGACGAGTCGACGTACAATCGAAGAGCTCAGCCACG				
scaffold377	**Hd4**	10326	2459681-2470006	Hd4_DRJ1R	345	2469662-2470006	TACGGACGGGCCATGATCCAACGGCATCGGCGCGGCCACGATATTGCAGTGCAGACTCTCTTATCAGCAGCAGGTTCTGCATCATGGTGTCGGCGGCCGTG<br>ACAGCGTCAATAGGTTTGAAAATTAGTTACTGATTACATGGGTCTCGAAGATTATACGGGTTTACACTTTGAAGACCCGTGCATTGTAGATCTTTAGAGCCTAA<br>CGTGGAGTTTCAGAGGGTTTGGTTCACAACCGTTTTTAATTCCACAATGCTTCGAGACGTTCGACACTACGTGACGAGGTGCTCAGCCGTTGATGCTGCGCTA<br>CATCGTGTCCACGAATGGATCACGCGATGCGATAACA	on 289nt<br>84%		(3 fractions) on 160nt 88%	
scaffold377	**Hd4**	10326	2459681-2470006	Hd4_DRJ1int	433	2466097-2466529	TACGGACGGGCCATGTTCCAACGGCGTCGGCGCGGCCACGATATTGCAGTGCAGACTCCCTTATCAGCAGCAGCTTCTGCATCATGATGTCGGCGACCGTG<br>ACAGTGTCAATAGGTTTGAAAATGAGTTACTGATTCCATGGGTCTCGAAGATCGTACAGGTTAACACTTCCAAGACCCGTGCATCGTAGATCGTTAGAGCCTAA<br>CGTAGTGGCTCAGAGCTAGGGTTTGGGGTGACCGTTTTGAATAGTACAGTGCTTGTTGCAATGTTCGTTACTACGTGATGAGTGCCAGTACGTGATGAACGTT<br>GGAACCTTATACTTACGATCTCCGCAACGCGTTGTTATATGTACACCATAACGCCTTTAGCGAAGCTACTCTGCCGTTGAGGCTGCATTGTATCGCGTTTGCAC<br>[illegible]		72%		
scaffold377	**Hd4**	10326	2459681-2470006	Hd4_DRJ1L	385	2459681-2460065	TACGGACGAGCCATGTTCCTACGGCGTTGGCGCGGCCTCGATATTGCAGTGCAGCACTCCCTTATGATCAGCAGCAGGTTCTGCATCATGGATGTCGGCGAC<br>CGTGACAGTGTCAATAGGTTTGAAAATTAGTTACTTATTTATCATGGGTCTCGGAGATCTGCATGGTGTAAATCTTTAGAGAGAGTGCAGCAACCGTTTTTAATT<br>GCACGACGCTTCGAGACGTTTGTCACTACGTGATGGATGTCAGTACGTGACGAACGTGGCGAGCTAATGACGATCTCTGCAACAGGTTGCGATATGTAGACC<br>AACGCGTTTAGCGGACGTGCTCAGCCGTGGATGCTGCGCTACATCGTGTTCGCACGAATGTCACGATGCGATAACA				
scaffold127548	**Hd6**	10461	5808388-5818848	Hd6_DRJ1R	311	5808388-5808698	ACCGGCTAATGCTAGGATGGTCGTTGATTACGGGCAACCACGTTGACACATGTTGGCACCCAACGAGGGATCTTCACCCTGGGCCCTGGGCCTGAGATCTTT<br>TGCCCGAGACTGCACCCGGCATCGGCAAACTATTCTGCCTCGGCAATTTTTCCTCGGTCGATCACTCTACTGTATCGTGATCCACCCCTTTGGAAACAACATG<br>TTCGACACATTATCTAAATGAGTAGAACGTTTCTGTTTACAGCGTCATAGAGATGCGTTGCGCTTGTAAAAAATGGTCTGCATGGCAATTGCAGTCTCGCTCCG<br>[illegible]	89%	on 270nt: 74%		
scaffold127548	**Hd6**	10461	5808388-5818848	Hd6_DRJ1int	323	5813253-5813575	ACCGGCTAATGCTAGGATGGTCGTTGATCACGGGCAACCACGTTGACACATGTTGGCACCCGACGACGGATCTGCACCCTGGGCCCTGGGCCTGAGATCAT<br>TCTCCCAAGACTGCACCCGGCATGGCTAACTATTCTGCCTCGGCAATTTTTCCCCAGTCGATCCACTCTACTGTATCGTGATCCACCCCCTTGGAAACAATAC<br>GTTCGACACATTATGTAAATGAGTAGAACGCGAGGTAGAACGTTTCTATTGACAGCATCATAGAGATGCGTTGTGCTTGTAAAAAATGGTCAACATGGCAATTG<br>[illegible]			on 282nt: 71%	
scaffold127548	**Hd6**	10461	5808388-5818848	Hd6_DRJ1L	269	5817294-5817562	GCAACTATGTTGACACGTGTTGGCGTCCAACGACGAATCTGCATCCTGAGTTTCATATCATTTGCTCAAGACTGCACCAAGCATGGCCAGCTATCCTGTCTCG<br>GCAATTTTTTCACCGTCCGTCGTTCTACTGTGTCGTGATCTGCCCTCTTGAGAGTAATTAATTTAACACATCATCTGAATAAATAGAACCTCTTGGTTCAGAGCG<br>TCATGGAGAGGCGTTGTGCTTATAAAAAATGGTTTCTACGGCAATTGCAGTCTCCGTTGTG				
scaffold127548	**Hd6**	10461	5808388-5818848	Hd6_DRJ2R	101	5808626-5808726	TGTTTACAGCGTCATAGAGATGCGTTGCGCTTGTAAAAAATGGTCTGCATGGCAATTGCAGTCTCGCTCCGTGGTGCAGCATGATACAGCAGAGCAAACCT	75%			
scaffold127548	**Hd6**	10461	5808388-5818848	Hd6_DRJ2L	97	5818752-5818848	TGTTCGCAGCGACATCGAGATGCGTTCGGCTTGAATAAAATGGTCTGCACAGCAATTGTACTCTCCATGGCGATGCATGGTACAGCGAAGTAAAACT				
scaffold127548	**Hd5**	10510	12941247-12954959	Hd5_DRJ1L	209	12941247-12941455	CACGTTCTAAACTCCACTCGTCCTTCGTTCACGGCATCGCCGTGCAGCACCCATTCCTCAATTCTTGCGCTGCACGAATTATAGTATAGCCTCGTTGTGAATAC<br>CTGAAGTAACGCATAACCCACTTCGGGAATGACGACCAAATGGTTGCCGATGGCTCGCACTTCCATCCCCGTTCGGTTATGCATTCACTGACGATAATCAGAG	on 209nt: 86%		on 209nt: 70%	
scaffold127548	**Hd5**	10510	12941247-12954959	Hd5_DRJ1int1	491	12946722-12947212	CACGTTCTAAACTTCACTCGTCCTTCGTTCGCGGCATCGCCATGCAGCACCCGTTCCTCAATTCTTGCGCTGGGCAAGTTATAGTATAACCTTGTTGCGAATGC<br>CTGAAGGAACGCAGAATTCACTTTGGGAATGACGGCAAATTGCTTGCCGACGGCTCACACTTTCATCCCCGTTCGGTTATGCATTCACTGCCAGTAAGCAAGC<br>TCATCATAGACAACCCAATCTCATAAGGCCGAGAATCGTCCTCGATAACACGGGCGTTATCAAGAGCTGAGTCCCAGAACGATCTGCCATTTTTGTTATTGCGA<br>GATGATAAAACAGCACATGTTCTTAACGCTTGTTTCTTATTACACACTTGTAAATTGCAAATGCGGGAATGAGAACTTAGCGTAGAGAATTGCTGCAACAATGCA<br>GGCATCAGAAAATGAAGTTGGACGCCAGCAGAAATTCAGCGGTAGAATCTACAGATCTGTGTAAACACCCTCACA		on 369nt: 84 %		on 383nt: 86%
scaffold127548	**Hd5**	10510	12941247-12954959	Hd5_DRJ1int2	369	12949974-12950342	ACTCGTCCTTCATTCGCGGCATCGCCGTGCAGCGCCCGTTCCTCAATTCTCGCGCTGGGCACGCTATACATAGTGTTGTCTCGTTGTGAAACCTGAAGTAATG<br>CATAACCCACTCGGGATTGACGGTACATTGCTTCACGACGACTCGCACTTTCATTCCCGTTCGGTTATGCATTCGCTGACGATGAGCGAGCTCATCTTAGACA<br>ACTTAATCTCGTAAGGCCGAGAATCGTCCTCTATAACATGGGCGTTATCAAGGGCTTGAGTCCAAGAACGATCTGCCATTTTTGCTATTGCGACATGATCGAAC<br>AGCTCATGTTCTTAACGCTTGTTGCTTATTGCATCCTTGTAAATTCCAAGTTCGGGAAT	on 254nt: 88%			
scaffold127548	**Hd5**	10510	12941247-12954959	Hd5_DRJ1R	464	12954496-12954959	GTACTGAACTCCATTCGTCCTCCGTTCACGGCATCGCCGTGCAGCACCCACTCTGTTCCTCAATTCTTCTCCACTATAGCTCTTCGGGAATGACGGCGAATTG<br>CTTGCCGACGGCTCGCACTTCCATCCCCGTTCGGTTATGCATTCACTGCCGATAAGCGAGCTCATCCTAGACAACCCAATCTCATAAGGCCGAGAATCGTCCT<br>CGATAACATGAGCGTTATCAAGAGCTGAGTCCAAGAACGATCTGCCATTTGTGCTATTGCGACATGATGGAACAGCTCATGTTCTTAACACTTGTTCCTTATTC<br>CACACTTGTCGATTTCAAGTGCGGGATTGAGAGCTTGCAGTATACAAAAGCGTAAAGAATGTTGCTGCAGCAATGTATGCATGAGAAGGTGAAGTTGGACGCC<br>GGCAGAAATTGAGCGGAAGGATCTACGGATCTGTGTAAACAATTCCTCACA				

scaffold127548	**Hd2**	13937	5940296-5954232	Hd2_DRJ1R	637	5951349-5951985	CATTACTGTCGGCCTCGAACATAGCAGCTGGATCGCGAGGAGACGTGTTCGTGAGAAGGTCTAGCGATCGGCTGTTATCACAGAGCCACCGTCCACGTTTACTGCACATGTTCTGCCCACACTTTGGAAATTCCGCATTTGCGGGATATGATAAACAAAAGTCGCGTACGCATACGCTGAAATACAGCATGCGTCACACGACATGTATAGGTATGGGGAATTACAAAATATCAAAACAAGAATAGAAACAGCGGAATGTACTCGAGCGCCATGATGTCATCCAGATTACACGGTTTCAGTTTGCGTTCTACATATAGTTTATGAACAAAGAAATCGACTGTGGCAGGTACACATGCAAAAAGGTTCTAGCAGATCTTTCCAGTTGCATTCTGGAATGCCAAAAAATCTGTCGCCAGCGTGCTTAGTTGACGCCGTGACTGCCGCTCCATGCAGTGCTAACCCAACTGATTGTTTGTCACGTACGTGACGGTCGCAGTGATGCTACTATGGAGATAACAAGTTGTGCACTGATTAACGTCCGGCAGGGCTTCACCTGTCGTACACCGACAACTTATTTCTTTATCGTATCTGGACTATACTGTTTACTGGAGCGGTCCCCTTTCCTGTAAAATAGTTT	96%	86%	
scaffold127548	**Hd2**	13937	5940296-5954232	Hd2_DRJ1int	640	5945753-5946392	CATTACTGTCGGCCTGAAACATAGTAGCTGGATCGCGAGCAGACATGTTCGTGAGAAGGTCTAGTGATCGGCTGTTATCACAGAGCCACCGTCCACGTTTACTGCACATGTTCTGCCCACACGTTGGAAATTCCGCATTTGCGGGATATGATAAACAAAAGTCGCCTACGCATACGCTGAAATACAGCATGCGTCACACGACATGTATAGGTATGGGGAATTACAAAATATCAAAACAAGAATAGAAATAGCGGAATGTACTCGAGCGCCATGATGTCATCCAGATTACACGGTTTCAGTTTGCGTTCTACATATAGTTTATGAACAAAGAAATCGACTGTGGCAGGTACACATGCAAAAAGGTTCTACCAGATCTTTCCAGTTGCATTCTGGAATGCCAAAAAATCTGTCGCCAGCGTGCTTAGTTGACGCCGTGACTGCCGCTCCATGCAGTGCTAACCCAACTGATTGTTTGTCACGTACGTGACGGTCGCAGTGATGCTACTATGGAGATAACAAGTTGTGCACTGATTAACGTCCAGCAGGGCTTCACCTGTCGTACACCGACATTTCATGTCTTTATTATCGCATCTGCACTACACCGTTTGCTGGAGCGGTCTCCTGTTCTGTAAAATAGTAT			86%
scaffold127548	**Hd2**	13937	5940296-5954232	Hd2_DRJ1L	651	5940296-5940946	CATTATTGTCGGCCGCAAACATAGGAACTGGATCGCGAACAGACATGTTCGTGAGAAGGTCTAACGATCGGCTGTTATCACAGGGTCACCGTCCACGTTTACTGCCCACGTTTACTGCACATGTTCTGCCCACATGTTGGAAATTCCGCATTTGCGGGATATGATAAACAAAAGTCGCCTTGGCATACGCTGAAGTTCAGCATGCGTCACACAACATGTGTAGGTATGGGGAATTACAAAATATCAAAACAAGGAAAGAAACAGCGGAATGTACTGGAGTGCCGTGATGACATGCAGACTACACGGTTCCAGTTTGCGTTCTACATATAGTTCATGAACAAAGAAATCGAATACGGTGAGTACACATGCAAAATGTTCCAGCAGATCTTTCCGGTTGCATTCTGGAGCACCAAAAAATCTGTTGCCAATGGGCTGAGGTGACGCCGTGACTGCAGCTCCATGCAACGCTAACCTAAGTAATTGTTTGTCACGTACGTGACGGTTACCGTGATGTTGCTATGGAGATAAGAAGTTGTGCACTGATTATAGTCGAGCATGGTTCACCTGTCGTACGGCGACAACTTATGTTCTTATTATCGCATCTGCACTACACCGTTTGCTGGAGCGGTCTCCTGTTCTGGAAAATAGTAT			
scaffold127548	**Hd2**	13937	5940296-5954232	Hd2_DRJ2R	294	5944329-5944622	TTCTGTATCATGCTGCAGCACGCAGCTAGACTGCAATTGCCATGCAGACCGTTTTTTACAAGCGCAATGCATCTTCTTAACGCTCTAAACAGAAACGTTCTACTTATGTAGAAAATGTGTCCAACAATGAGTTCCCGAGAGAGCGGATCGCGATACAAGAGAACGACGGTCCGTAAAGAAATTGCCGAGGCGCAATAGTTGACCAATGCACGGTGCAGTCTTGAGCAAATGATCTCAAGCCCGGGATGCAGATCTCTCATTGAGCGCCGACGTGTCAACATGGTTGCCTACCAT	84%		
scaffold127548	**Hd2**	13937	5940296-5954232	Hd2_DRJ2L	296	5953937-5954232	TTCTGTATCATGCTGCACCACGGAGCGTGACTGCCATTGCCATGCAGACCATTTTATACAAGCGTAACGTATTTCCATGACGCTCTGAACATGAACGTTCTACGTATTTAGAAAACGTGAGCAACTGATTGTTCCCGAGAGAGCGGATCGCGATACACGAGAACGACGGTCCGTGAAAAAATTGCCGAGTCACAATAATTCACCAATGCACGGTGCAGTCTTGAGCGGAAAATCTCAAGCCCGATATGCAGATCCGTCATTGGGCGCCGACATGTGTCAACATGGTTCCTGACCAT			
scaffold90	**Hd1**	NA	1-14771 (partial)	Hd1_DRJ1R	203	14569-14771	GCGCCTCTCACGTACGCTCCTAAACGGTGATGAAGCTCGGTAATCATCTTCACTTCGACATGATCGACGACTCAATTCAGTCAATTCAATCGCATGTTTTCATTCTCGAATGTCAAAGGCCGATAAAGCCGGCAGTGCGCAGGAAGAGTGAACGGAAATGAAAACTGAGATTTCTCTATATTATTAAAAATTCAAAAAATTTA	96%		
scaffold90	**Hd1**	NA	1-14771 (partial)	Hd1_DRJ1int	203	1-203	GCGCCTCTCACGTACGCTCCTAAACGGTGATGAAGCTCAGAAATCATCTTTACTTCGACATGATCGATGAATCAATTTAGTCAATTCAATGGCATGTTTTCATTTTCGAATGTCAAAGGCCGATAAAGCCGGCAGTGCGCAGGAAGAGTGAATGGAAATGAAAACTGAGATTTCTCTATATTATTAAAAATTCAAAAAATTTA			
scaffold90	**Hd1**	NA	1-14771 (partial)	Hd1_DRJ2R	255	12006-12260	CAGCTAAGACACTATATGTAGTTTTAGAAGTACGCCATATCTACAGTTGCGCTATGAAGCACAATATTCTCCAGAACCTGAGTCTCTAATGTTGTATTGCATTTGTTGACGGTGGCCACAGCCCGCGCAGGAGCATGTCGTTCCATTCATATGACATTAGCCGAACCGCAAGCAAGCGGTTACACCCGATACGGTCATAGCAGATAGTGAGCTTTATAGTTTTTGTTACGACACATGTGTGTTTATATATATGTA	89%		76%
scaffold90	**Hd1**	NA	1-14771 (partial)	Hd1_DRJ2int-1	252	7539-7790	CAGCTAATACACTGTGTAGTTTTAGAGGTACGTCGTATTTACAGGTGCGCTATGAAGCACATCATTCTCCCGGACCTGAGTCTCTAATGTTGTATTGCATTAGTTGACGGTGGCCTCAGCCCGCGCAGGAGCAGGTCGTTCCATTCATATGACATTAGCCAAACCGCAAGCAAGCGGTCACACCCGATACAGACATAGCAGATAGTGAGCTTTATAGGTTTTGTTACTACATATGTGTCTTATATGTGCGTA		74%	
scaffold90	**Hd1**	NA	1-14771 (partial)	Hd1_DRJ2int-2	249	1114-1362	CATCTAATACTCTATATGTAGTTTTAGCAGGACGTCGTGTGCTTAGTTTTGCACTTGCAAGTAGTATATTCTTCCGAACCTGACTCTCTGACGTCGCATTAGTTGACGGTGGCCACAGCCCGCGTAGGAGCATGTCATTCGATCCATACGTTACCCGAACCGAAAGCAAGCGTTTCCACCTGATACGGACTTACAGATAGTGAGCTTTATAGTCTATGAATTCGTGCAATATGTGTGTATATGTGTGTA			
scaffold128215	**Hd9**	17892	526917-544808	Hd9_DRJ1R	157	526917-527073	TCCCAAAATCGTCAACTTGTGATAAAACATCCAACGAAGGCATCTCTCAGAGAATCCGGTGCAGCTCCAGGATGGTCCGCCGTATCGGATTCCACCACAACCCGATACAGCTGAACATCTGGGACATCTGACGGGAACTCTCTGCTCAGTTTCAATT	90%		
scaffold128215	**Hd9**	17892	526917-544808	Hd9_DRJ1L	156	542329-542484	TCCCTAAATCGTCAACTTTTGATAAAACATCCAACGGAAGCATCTTTCAGAGAATCCACGGAGCTGCAGGATGGTCCACCGGCTCGGATTCCACCACAACCCGATACAGCTGAATATCTGGGACATCTGACGGGAACTCTCTGTTGAGTTTCAATT			
scaffold128215	**Hd9**	17892	526917-544808	Hd9_DRJ2R	305	530838-531142	ATGCTAGGTTTCACATGCATTTGTGGGTTACGGCTGTACCTGTATGTACATGTAAGTGTCGTTTTGCACGTGCTATCAATCAGACAAAAAAGAAGGCGATAGTATAGACTCTCGTCCGTTCGGCTGCAGCTGCACTAGCGAGATCCACTCTGTGTCTCCTGTCTATAGCTTACTTATTACTTGCAATTGTAAAAGTATGGGAAAACTGAGGGCTAGGAACACAGTAGTTTTTATGTCGTCTTTTAATAGATCCCGTCAGCAAGAGATGAGATGGTTAATAAGTTAGCGAACTAGAGAGAGGCGGA	85%		
scaffold128215	**Hd9**	17892	526917-544808	Hd9_DRJ2L	308	544501-544808	ATGCTAGGTTTCATATGCATTTGTGGATCACGGCTGTACCTGTATGTACTTGTAAGTGTCGTTTTGCACGTGCTATCAATCAGACAAAAAAAGAGGGCGATAAAGGAGAGACTACTCTCGTCCGTTCGGCTGTAGCTGCACCAGCGAAATACCCTCTCTGTCTCCTGTCTATAACTTACTCATTGCCGACAATTGTAAACGTATGGGAAAACTGAGGGCTAGGAAGACATTGGTGGTTGTGCCGTCTTTCAATCGATCTCTTCGGCACGAGATGAGATAGTTAAGTTAGCGAACTAGGAAAAGGCGGA			
**Campoletis sonorensis**										
scaffold_11	CsA	6368	861628-867995	CsA_DRJ1L	244	861628-861871	AATTGACGGCAAGCAGATCATGTGCCGTGCATTCCCCAGAGGTAATCGCAATTTGTGTTATCAAAACTGAGTCATGACTTGGCGACTCACTAGGCTGGATGCCAAGGATTCTGATCGCTGTCCACCGGCGGTAATGCACCGTCGTACCCGACCCGGAGATCATTTTCAACTTGACCGTTTCTTTTCAAAATTACGGAAAATATGGAACCTGTCTGCTTCCAAATGCAGTTGACGTCGGTTGTGA	91%		
scaffold_11	CsA	6368	861628-867995	CsA_DRJ1R	249	867747-867995	AATTGACGGCAAGCACGGCATGTGCCGGCATGTGCAGTCCCCAGAGGTAATCGCAACTCGTGTTGTCAACGCTGAGTCGTGACTTGGCGACTCACTAGGCTGGATGCCAAGGATTCTGATAGCTGTCCAGCGGCGTTAATGCACCGTCGTACCCGACCCGGAGATCATTTTAAACTTGACCGTTTCTTTTCAAAATTACGGAAAATATGGAACCTGTCTGCTTCCAAATGCAGTTGACATCAGATGTGA			
scaffold_49	CsB	6626	22030-28655	CsB_DRJ1L	110	22030-22139	AGACTTGCTACGGTTTAAGAGCTGCTGTCAGAGCTGATTCGTGATAGACCAACACGATGTACGCTTCCTGCGGGAACAGATAAGCACCCGGGCCACCCAGTTGATGCAGT	76%		
scaffold_49	CsB	6626	22030-28655	CsB_DRJ1R	112	28544-28655	AGACTCGCTACCGTTCGGCAGCTGCTATACCAGAGCTGGTACGTGAGTAGTTGACACGATATACGCTTGCTTCGGGAACAGATGATCTCCCGGGACAGCCTGCCGATGCAGT			
scaffold_17	CsE	7990	1330025-1338014	CsE_DRJ1L	233	1330025-1330257	GAGCTGTCTGGAGCCAACCAAGCAACGGAACGAAAAGAAACGAATGTCTATCATCGTTGAATTACAACCAGGGAACTTTTAAAAAAATCTTTCCTAGGATTGAACCACGGATTAGAACTATACATGCAGATCAAGCTCATGCGGATCGGTTCGTGTAAGGCACTCATGTGCATAGCTATTAGACCTCGCATGATCGAACAATCACGTTTTGAAGCGTGGGCACGATGCGGACC	93%		
scaffold_17	CsE	7990	1330025-1338014	CsE_DRJ1R	230	1337785-1338014	GAGCTGTCTGGAGCCAACCAAACAACGAAACGAGAAGAAACCAATGTCTATCATCGTTTAATTACAACCAGGGAACTTTTTAAAAACTCGTTCCTAGGATTGAAGCACGGATTGGAGCTATTGCAGATCAAGCTCATGCGGATCGGTTCGTGTAAGGCACTCATGTGCATAGCTATTAGACCTCGCATGATCAAACAATCACGTTTTGAAGCGTGGGCACAACGCGGACC			
scaffold_131	CsF	8155	808380-816534	CsF_DRJ1L	259	808380-808638	ACTGCCTGGTTCGTGTTTGTTCGCTAGCTTCTGCTCGCTTTGTTTCTAACCTAACCCAAGAATGTTCGCACGGACCCGTTATTGGTGCCCGATAACAGCTTTCTTTATACTCTTATGGTCCTGTTCTACAACCAGGTTCCAGCTTATTTCTATTTTTCAAATCCAGTATCGGCCCAGTTCACTGCTTCAAAAACAGTGAGCTTGTCAGACACGTAAACCATCAATTTTTTGTACAATCCACAATTTAAAAATAAAAAAG	78%		
scaffold_131	CsF	8155	808380-816534	CsF_DRJ1R	256	816279-816534	ACTGCTGCGTTCGTGCTCATTTGCTAGCTTTCGCTCGCTTTGTCTGTACTTAGCCTGACAATGTTATCATGGACCTGTTATTGGTACTCGATGACAGCTTTCTTTATACTCTTATGATCCTGTTCGTCAACGAGGTTCAAAGTTGCTTCCATCTTTCAAATCTAGTATCAGCCCAGTTCGCTGTTCAAGAAATAGTGAGGTTGCCGGACTTGTAGACCATCAATCATTGGTATGCTACACAACTTAGAGATAAAAG			

scaffold_10	CsD	8168	961052-969219	CsD_DRJ1L	711	961052-961762	GAAAAGTTTTGGGAGAGTTGGCTGTTGAAGTTGCGAGTTGAACTTCAGTTTCTCCCATGTCAAAACTACGTTGAATGAAATTGAGTTGAGCTTAGACGACGTTCGACGAAACGGCCAGAAGCTCAGAGAAGATTGTCAACGAGCATCCAGCACAGAGTCGAGTGAGCCTAGCCACGAAGTTCGTGCCGTACGAGATGAGTCGTCGAAAAGCATAGGACGGCGATAGTACGTTATACCCCGTTGACGGATGGCAATGTCTCGTAAAGAAGTGAAAAGCTTCATGTTGAGCAAGTGGTGATCCGTCGATTTGAACCGAGCATCACTCGCATGATAGCCTCTCGTAGTGTCACTTCTAGAAGAACCGCACGTCGCTTCTCAGCAACAATTTACAGGTTTGGCAGTTGTTACACCGCAACTAAGCGACAATAACTGCATTTTGACATACGACAGGTTTGTATCGTTATCATTGCACGTGAAGTTTCATCCACCAATGGTGCTCGACCTGTGAATACATTGGATAGCGTGACTTAGCGAGTACCTTCTTTTATCACGACACCAAACTGCGTCAACAGCGTCTACCGTCAGCTCCAATATGTGGATAGGAGCATGAGAGTTATCTGCTGCTCGCATATACCGTCTTTTCACTGTCCCCGCCCCAGGTACACGCCAGATAAACTAACAATGTGCTCACGTACAGATTTCAAAAGTAGG	98%		
scaffold_10	CsD	8168	961052-969219	CsD_DRJ1R	711	968509-969219	GAAAAGTTTTGGGAGAGTTGGCTGTTGAAGTTGCGAGTTGAACTTCAGTTTATCCCATGTCAAAACTACGTTGAATGAAATTGAGTTGAGCTTAGACGACGTTCGACGAAACGGCCAGAAGCTCAGAGAAGATTGTCAACGAGCATCCAGCACAGAGTCGAGTGAACCTAGCCACAAAGTTCGTGCCGTACGAGATGAGTCGTCGAAAAGCATAGGACGGCGATAGTACGTTATACCCCGTTGACGGATAGCAATGTCTCGTAAAGAAGTGAAAAGCTTCATGTTGAGCAAGTGGTGATCCGTCGATTTGAACCGAGCATCACTCGCATGATAGCCTCTCGTAGTGTCACCTCTAGAAGAACCGCACGTCGCTTCTTAGCAACAATTTACAGGTTTGGCAGTTGTTACACCGCAACTAAGCGACAATAACTGCATTTTGACTTACGACAGGTTTGTATCGTTATCATTGCACGTGAAGTTTCATCCACCAATGGTGCTCGACCTGTGAATACATTGGATAGCGTGACTTAGCGAGTACCTTCTTTTATCACGACACCAAACTGCGTCAACAGCGTCTACCGTCAGCTCCAATATGTGGATAGGAGCATGAGAGTTATCTGCTGCTCGCATATACGGTCTTTTCACTGTCCCTGCCTCAGGTACACACCAGATAGACCAACAATGTGCTCACGCAAAGATTTCAAAAGTAGG			
scaffold_14	CsG2	8338	192247-200584	CsG2_DRJ1L	173	192247-192419	GATCGACGGCAGCATCTTTTTCATCAACGAGTGACAATCGTCACCAGCCCCCGGCATTGTAGAATTGAAGAACGGATTCATAACCTTCTTGGTTGACAAACAGAGCGTGTAGACGCAATAATCTATCGAGGGTCCCAAAGGCCACATACCGTACGATCTTTGAGACCGACGGA	74%		
scaffold_14	CsG2	8338	192247-200584	CsG2_DRJ1R	173	200412-200584	GATCGATGGCAGCATCTCTTCCATCAGCCAGCGACCAACATCACGAGCCTCCGGCATCCAAGAATTGAAGAACATTTGTATAACCTTCTTGATTGACCAGCGGAGCGTGTGGATGCGATGATCGTTTGAGGGGTTCAAAGGTCGTATACTTAATAATTATTCAGACCCACGGA			
scaffold_14	CsG	8656	76017-84672	CsG_DRJ1L	372	76017-76388	ACAGCATTTGACATTGAAAATGTTTCTCCGCGAGTGTGAGTAAGAGACGGTAAGATAATTTCTGTTATCTTCAGCTCTCACTTTTCACAATTGATCCCATGATACTGCAGAGTGCGGCCAGAATATGCTGCGCACTGTAAGCGTGGTGACAACTGTGTCCAGAGCAAGCTCCCACCATGTCCACTGTTTCGCGTAAACTTTTCTCGTAGCAAAGACTTATTGCTATCAGTTACAGTGTCACACGGTGGACGCTTGGCACAGGTTGACAGGTTGGCCTGCTGAGGTTGGAGATATGCAACGGGAGCTGGTAACGCAACACACTCACTTCGATATACGCATGCTAGGGGAAAACAGTTAATATTCTGCTAATGC	97%		
scaffold_14	CsG	8656	76017-84672	CsG_DRJ1R	372	84301-84672	ACAACATCTGACACTGAAAATGTTTCTCCGCGAGTGTGAGTAAGAGACGGTAAGATAACTTCTGTTATCTTCAGCTCTCAGTTTTCACAATTGATCCCATCATACTGCAGAGTGCGGCCAGAATATGCTGCGCACTGTAAGCGTGGTGACAACTGGGTCCAGAGCAAGCTCCCACCATGTACACTGTTTCGCGTAAACTTTTCTCGTAGCAAAGACTCATTGCTATCAGTTACCGTGTCACACGGTGGACGCTTGGCACAGGTTGACAGGTTGGCCTGCTGAGGTTGGAGAGATGCTACGGGAGCTGGTAATGCAACACACTCACTTCGATATACGCATGCTAGGGGAAAACAGTTAATATTCTGCTAATGC			
scaffold_22	CsI	8779	695663-704441	CsI_DRJ1L	204	695663-695866	GACATCTCTATCAGAGGACATCTCTACCAATCCAGTCAAGCATTCCCAGCTCGAGGATTCCGATCTTCTTGAGTTGCGGTTGATGGATTAGATTAGTAGAGATGTCCTCTGTTTAGAGATGTCCTCTCTACAGCCACCAGTTTTTTTTCTGTATTCAGTTTGCAAAAGTGAAATGTTGCAGACTAAGAGCGTAGACGAATCATG	84%		
scaffold_22	CsI	8779	695663-704441	CsI_DRJ1R	202	704240-704441	GAAATCTCTACCACCGGACATCTCTACCAGTCCAGTCAAGCAATCCCAACCCAAGGATTCCAATCTTCTTGAGTTTCAATTGGTGGATTGGATGGTAGAGAGGGCCTCTGGGTAAAGATGTCGACTCTACAGCCACCAGCTTTTTTCTGTATTCAGATTGCAAAAGTGAAATGGTAAAAACTAAGAGCGTAGGCGATTCATG			
scaffold_128	CsI2	9042	110016-119057	CsI2_DRJ1L	174	110016-110189	CGCCTGAAAACGGCCTTCATTTAATCTGGAAACGAACCTAAGACAGTTGGACTAACAAGAATCGTGGCTCAGCGACTACCTAATCCTTCAATGTAATCTAACCATGATTGGCAGTTTGTGATACAGCTTCCGCTGTTGTCAAAGTAAGCTAAACAGAAGATCGAGGGAAACAAT	75%		
scaffold_128	CsI2	9042	110016-119057	CsI2_DRJ1R	177	118881-119057	CGCCTGATAATGACATTCAGTTATTCCGGACACCAACCTCCTGCAGGTGGCCTCACAGGGCACGTGGCTCAGCGATTTCCTAACCCTTAAATGCAATCTCACTATGATTGGTACTTTGTAATACACCCCCCGCTGCTCTCAAGCCAAGTCGTTAAGCAGAAGAACGAGGGAAACAAT			
scaffold_38	CsH	9050	1398066-1407115	CsH_DRJ1L	731	1398066-1398796	GCAAATGTTCAGTGCATAGCCTACCTGTCGTTGGACGGTGACGAAGTTGTGAACACCGTGCTATCATTTGAGCGAAGCGCTTTCGCATTTTTAATTTATTTTCCAAAGTTGAATGCGTTAAGCCCCTTCAAATAGCCTTCAGATAGTTTTTAGCGGAGTTTTTCACAAGCTCAAGAATTGACGTTGGTTGCAAATGCTGCAACGGCACGTAAACTCGCCAGCTTACAATGACCTGATATCTCGCATTGCACAGCGCTTTGACTATAGTTTTTTGGTCGCTTATAGTTATTTTGGATGTCGTACGTATGCGTATAGTATGCATGGTATACCTCCCACATGTCTACTAATGTGCTTGTCCTCTTGAACGTATATGTTTTTTTTCTATGGATGTATGCAGGAGTGCGTTTTCGTTGTTTTCAGTAATTTCGTTATCGTTATTCGTGTGGAGGATGCATTAAATACGTTCATGATATCTCAATGTGTGTGTGATAAGTTGCTTATTTTCATATGTGTGATCACTTTTATCAAGCAGTTCTTAATCGTAGAATAGTTAGACTAGATGCGGAAACTGGTTAGGCGTTACGATCGACACGATGTGCACATTGCCCTGAACACTGCTGATATTTTCTTGTAAACGCGATTTTCATTATATTCGCGGCATACTATAGTATGCTAGATCAACTGTATCGACACGGTATCAAGTGTTCCCGGAGCAAGCGTGTAATCATGGT	98%		
scaffold_38	CsH	9050	1398066-1407115	CsH_DRJ1R	731	1406385-1407115	GCAAATGTTCAGTGCATAGCCTACCTGTCGTTGGACGGTGACGAAGTTGTGAACACCGTGCTATCATTTGAGCGAAGCGCTTTCGCATTTTGAATTTATTTTCCAAAGTTGAATGCGTTAAGCCCCTTCAAATAGCCTTCAGATAGTTTTTAGCGGAGTTTTTCACAAGCTCAAGAATTGACGTTGGTTGCAAATGCTGCAACGGCACGTAAACTCGCCAGCTTACAATGACCTGATATCTCGCATTGCACAGCGCTTTGACTATAGTTTTTTGGTCGCTTATAGTTATTTTGGATGTCGTATGTATGAGTATAGTATGCATGGTATACCTCCCACATGTCTACTAATGTGCTTGTCCTCTTGAACGTATATGTTTTTTTTCTATGGATGTATGCAGGAGTGCGTTTTCGTTGTTCTCAGTAATTTCGTTATCGTTATTCGTGTGGAGGATGCATTAAATACGTTCATGATATCTCAATGTGTGTGTGATAAGTTGCTTATTTTCATATGTGTGATCACTTTTATCCAGCAGTTCTTACTCGTAGAATAGTTAGACTAGATGCGGAAACTGGTTAGGCGGTACGGTCGACACGATGTGCACATTGCTCTGAACACTGCTGATATTTTCTTGTAAACGCGATTTTTATTATATTCGCGGCATATTATAGTATGCTAGATCAACTGTATCGACATGGTATCAAGTGTTCCCGGAGCAAGCGTGTAATCGTGGT			
scaffold_16	CsX6	9213	504600-513812	CsX6_DRJ1L	234	504600-504833	TAACTAGATTCTTACTTTGGTTCTGTGAGAACGTGACCCGGTCACGTTTGTCATTATTCGATCAAAACTTATTAGTCTTGCTTATTTTAATTCTTCACGGTTGAAAGATTGTCCTCGAGATTCTGATTCCGAAGCATTAGAACGACTATGAATCGTCTACACTCTTAGTCTTCACCATTTCACTTTTGCGAACTGAATACAGAAAAAAAGCTGGTTACTGTGGAGTGGACATCT	89%		
scaffold_16	CsX6	9213	504600-513812	CsX6_DRJ1R	231	513582-513812	TAATACATGCTTACTTTGTTTCTGTGAAAACATTACGTGGAGTTTGGTCATCATTCGACCAAAACTTAATAGTCTTGCTTATTTTAATTCTTCACCGTTGAAAGATTGTCCTCGAGATTCTGATTCCGAAGCATTAGAACGACTATGAATCGTCTACGCTCTTAGTCTTCACTATTTCACTTTTGCAAACTGAATACAGGAAGAAAGCTGGTGGCTGTAGAGTGGACATCT			
scaffold_15	CsJ	9484	2621922-2631405	CsJ_DRJ1L	342	2621922-2622263	TCCCTGCGAATCCACAGCAAGACTCCGTCACCGCTCCATCGTCGGCCACGCTGCTTGACATTGCTGAGCAGTTGAAAAGTTCTGCAGAAATGACGGCGTTACACGGCATCATGCATGAGGGTACAAGGCTGCGACAAGCGACGACTGAGCTCAACCACGGTTTATTTTGTTCTAGCTTCAAGCGTCGCTGATGTACCTCTGAGCGAAAAAAGATGCAGCAATGTTTTTACATTTGTACATTTTTTTTTAGTACCAGTAACCATTGATGTGTTGCTCGTTGTATACTGATGGATGGGAATCTGCTTTTGAGTCGTCCCCCGAACATTTACGCATAACGCAAGC	75%		91%
scaffold_15	CsJ	9484	2621922-2631405	CsJ_DRJ1int	246	2628392-2628637	TTGACATTACTCAACATTTGAAACGTCCTATGGACCTAACGGTGTTGCTGGATCATGTATGCGGGTGCAAAGCTTTGACGAGCATTGACTGGGCTCACTTATGGTTTTTTGTTGTAGCTTCAAGCGTCGCAGATGCATCATTAATTGCACAAAGGTGCATCAATATTTTCACATCTGTACATTTTTTTGATTACCGGTAACCATTGTTGTGTTACTCGTTTTATTTGGACGGATGGGAATCCGCTT		73%	
scaffold_15	CsJ	9484	2621922-2631405	CsJ_DRJ1R	341	2631065-2631405	TCTCTGCGAATCCACATCAAGACTACCTTCGCTACATCGTCGACCACACTGCTTGAAATCACTAAGTAGTTGAAACGTTCTGCAGAAGTGACGGCGTTACACGGCATCATGCATGAGGGTACAGGGCTGCGACGAGCGACGACTGAGCTCAACCATGGTTTATTTTTGTTCTAGCTTCAAGCGTCGCAGATGTATCTCTGAGCGAAAAAAGATCCAGCAATGTTTTTACATTTGTACATTTTTTCTTAGTACCGGTAACCATTAATGTGTTGCTCGTTCTGTACTGATGAATGAGAACCTGCTTTTGAGTCGTCCCCCGAACATTTACGCATAACGCAAGC			
scaffold_35	CsX8	9999	164467-174465	CsX8_DRJ1L	249	164467-164715	TTGGTAGCTTGCTTAACGTCAGCATCCTTCTTGTTCGTGATCCGTACTGTTTTTGCCTTCATTTTCACATGAACATCATCTTTCTCTCCGTTCTTGCGATAGCGGCAAACGAGGGATCACGTTTGAGTGGTTTGTCTGCTCGCGCATGTCATAGTTACAGCTATTCATGATCTCCAGCAGGACCTCTCCCATTGCACACTAACTAACATCGTGTGCTCGTGACATCGTGTAATAAAGAACAGTTGCTTC	80%		
scaffold_35	CsX8	9999	164467-174465	CsX8_DRJ1R	252	174214-174465	TTGTTGGCTTGTTGAATGTCAGCATCCTTCTTGTTCGTGATCAGAACTGTTTTCATCTTCACTTCAACTTTAACATCATATTCCTCTCTGTCCTTGCGATTGAAGCAAACGATGGAGCTTGTTTGAGTGGTCCATCTGCTCGCGCGTATGTCATAGTGACTGATCTTCATGATCTCCGGTAGCACCTCTTCCACTGCTCACTGACGAACATCGTGAGCTCGTGGCATCGTGGAATTGAAGAACAATTGCATC			

scaffold_4391	CsL	10024	8079-18102	CsL_DRJ1L	660	8079-8738	TTTAGAACCTTACGGACACAGTTGATCTGCTCCGTCTTGCCAAGTGCCTGCCATGGTAGCTTATGCTTGTCATCTGTCTGAGTTCGTTAACGCTTACCTGTTCTGCTTGGCGGCTTGTTCAATCTCAGCATCCCTCTTGTTCGTGATCAGAACGGTTTTTACCTTCATTTTAACATAAACATCATCTTCCTCTCCGTTCTTACGATTGAGGCAAACGATGGTTCGCGTTTGAATGGTTTGTCTGCTCGCGTATGTGATAGTTACTGATTTTCGTGATCACCGGCAGCACGTTTTTCATTGCTCACTGACAAACATCGTGAGCTCGTGGCATCGTGGAATTGAAGAACAATTGCATCACATGGTTTTGGTTGACAAACAACTCTGTCATAATAATATTATTTGTCGTGCATGAGTCCGACAGGTCTTGCACCGCACAATCTTCGACAGTCGTGCAATTTTTTGGCAACCTTCACATCCATTTCAATGTCGACATTACCATGTAAAGCTTGCTGAAGATCGCTCTTCTGCGGATCCAAGTCAGCGTCAACGCTATAGGATGAAGATTCGTCAGTAAACTCGTTTTAGAAGTACTGTAGGTCAAATCCGTGGTATATCAGAACTTTTCGAATACGAAGTTGTCCTCGTCACAAAAACATACGC	93%				
scaffold_4391	CsL	10024	8079-18102	CsL_DRJ1R	634	17469-18102	TTTAGAAACTTACGGACACAGTTGATGTGCTCCGTCTTGCCAAGTGCCTGCCATGGTAGCTTATGCTTCTCATCTGTCTGAGCTCGTTGACGCTTCCTTGTCCTGCTTTACGGTTTGTTCAATGTCAGCATCCCTCTTGTTCGTGATCAGAACGGTTTTTACCTTCATTTTAACATAAACATCATCTTCCTCTCCGTTCTTACGATTGAGGCAAACGATGGTTCGCGTTTGAATGGTTTGTCTGCTCGCGTATGTGATAGTTACTGATTTTCGTGATCACCGGCAGCACGTTTTTCATTGCTCACTGACAAACATCGTGAGCTCGTGGCATCGTGGAATTGAAGAACAATTGCATCACATGGTTTTGGTTGACAAACAACTCTGTCATAATAATATTATTTGTCGTGCATGAGTCCGACAGTCGTGCAATTTTTTGGCAACCTTCACATCCATTTCAATGTCGACATTACCATGTAAAGCTTGCTGAAGATCGCTCTACCGCGGATCCAAGTCAGCGCCAACGCTATAGGATGAAGACTCGTCAGTAAACTCGTTTTAGAAGTACTGTAGGTCAAATCCGTGGTATATCAGAACTTTTCGAATACGAAGTTGTCCTCGTCACAGAAACATACGC					
scaffold_5	CsN	10943	175169-186111	CsN_DRJ1L	254	175169-175422	CTCTCTATTTGCTAGATTCATTGGAAAGTTACGTGTCCCCATATATATTAGCCAAAGTCATACACATGGGAGGAGCGAACGTGAATTCGAGGCTCTTAAGAGATCCCCAGGTAAAAGTTCCAAAACAAGTTGACGTTGCACAATTTGGGTACGTGTAGTTTGCACACGGGATATGGAGCTTTCAAGTTGAGAATGAAACCAACGGGTCCGGATATTGTTTAGTTTTCAAGAGCACCGCGTAACGTAATCAAAGA	78%				
scaffold_5	CsN	10943	175169-186111	CsN_DRJ1R	258	185854-186111	CTCTCAATTTGCCAGATTCATTGAGAAGTAATGTGTCTCCGGATTAACTAGCTGGAATATTACTAAGATGGATGGAGTGAACGTGAATTTGAGGCATTTTAGAGATCTCCATGTAAAAGTTAATAAGATAAGTTGACGTTGCACGGTTTGGGAACTGGTAGTTATGTACACGGTACATGAATCTTTCAAGTCAAGGATGAAACCAACGGGTCGGGATATTGTTTACTTCTGAAGAGCAGTGCGTAACGTACTCAAAGA					
scaffold_110	CsX2	10806	248068-258873	CsX2_DRJ1L	123	248068-248190	GAAATACTATTTCACGACCTTGTGATTCATAGATCGTTGACGTTGACTATACTGAATTGGAGATAGAATCGACAACCCATGCAATTGTCTCCTGCAAACTTCGAAATATGCAGGTTGAAGAGC	74%				
scaffold_110	CsX2	10806	248068-258873	CsX2_DRJ1R	125	253752-253876	GAAATACCATTTTCCACGGCCTAGCCATGGATAGATTGTTGAAGTTATCTATACTGAATTTGGAATAGAATCGACAGACCATGCAATTCTATTCTCCGAACGACGAACTGTAAAAGTTAAATAGC					
scaffold_110	CsX2	10806	248068-258873	CsX2_DRJ2L	127	249564-249690	TGTGCAGAACCGAAAGTGATTGAAAGTGAATCGAGTAATTAGAACGATTAAACATAAGCTAGTATGTGGGCAGGTTGTTTGTGTATTATTTTTTGATTTTCGAAATGATGAAATTATGCCATAAAAT	72%				
scaffold_110	CsX2	10806	248068-258873	CsX2_DRJ2R	128	258746-258873	TGAGCAGAGCCGAAAGCGAATGAAAGTATATCGAGTGACAATCGGCATTGGACGTGAGATAGTAGGTAGACAGATAGTGTATTTTTTTTTGTTTTGATTTTCGAATTGTTAAAATTGTGCAATGAAAT					
scaffold_5218	CsP	12113	15720-27832	CsP_DRJ1L	277	15720-15996	TGCTTAGCTCTGAGTCAGATTGTAGTTCAATTACTGAGTGAAAGGTGGTTGTCTTGAATTTCCAATGGTGTTAGCTATTTGGAACCCACTGAAAAAAATAGCCACCTGTTGCGGTTGGTGTTTACATTGTTTCCCTCGTTCTTCTGCTTAGCTTGACTTGAAAGCAGCGGGGGGTGTATCACAAACTCCCAATCATAGTGAGATTACATTCAAGGGTTAGGAAATCACTGAGCCACGTGCCCTGTTAGGCCATCTGCACGCGGTTGGTGTCCGCGAT	72%				
scaffold_5218	CsP	12113	15720-27832	CsP_DRJ1R	274	27559-27832	TGCTTAGCCAGACGTCGGCCTGTAGTACTATCACTGAACGAGACGGCGCTGTCTTGTGTTTCTCATGGTGCTACTTAACTGGAACTCGAGGAAAAGACAGCCACTTATATCGGTCGAAGTCAAACTGTTACCCTGGATCTCCTGTTTAGCTTACTCTGACAGCAGCGGGAGCTGTATCACAAACTGCTGATCATGGTTAGATCACACTGAAGGGTCAGGTATTCGCTGAGCCACCATTCTTGTTAGTCCAACTGCCCTAAGTTCGTGTCCGGAT					
scaffold_23	CsM	12197	1422227-1434423	CsM_DRJ1L	307	1422227-1422533	AACATTTTCTAAAGTTCCCACGAATTTTTCGGGCAACTGGTACGTTCTCCAGCCATCGTACAGAACTGTTTAGGGATATTTTCACAGCGAGACGACGGAGTGAAATTCGATCGACAAGAAAGTATGTCTTTGAGTACGTTACGCGGTGCTCTCAAGAAGTAAACAATATCCCGACCCGTCGGTTTCATCTTTGACTTGAAAGCTGCATGTACCATGTGCAAACTACCCGTTCCCCAAATTGTGCAACGTCAACTTGTCTTGGTAACTTTTACCTGGGGATCTCTTAAGAGCCTCAAATTCACGTTCA	87%				
scaffold_23	CsM	12197	1422227-1434423	CsM_DRJ1R	307	1434117-1434423	AACATTCTGTAAAGCTCCCGCGAGCTGTTCGGATGACTTCAACGTTGTCCAGCCATTGTACAGAACTGTTTAGGGATATTTTCATAGCAAGACGAACTAGTGGAATCCGCGCGACGAGAATGTACGTCTTTGATTACGTTACCCAGTGCTCTTGAGAACTAAACAATATCCCGACCCGTTGGTTTCATCCTTAACTTGAAAGCTCCATGTACCGTGTGCAAACTACCCGTTCCTCAAAATGTGCAACGTCAACTTGTCTTGTTAACTTTTACCTGGGGATCTCTTAAGAGCCTCAAATTCACGTTCA					
scaffold_50	CsQ	12543	290527-303069	CsQ_DRJ1L	437	290527-290963	CAATAAGGTTGATTGAACAGACCAATGTTGTTGCAGGCAGCTTAAGGACTGCTTGTAAAATGATGATCCATGGGATCGTCGGTCTCAGTTTGACCAAGTTTTGATTTTAATAGCAATTGAAATAACGACGCATTGTAATCCGTCTTCGGACCATATAGTGCTGGCTCCATGTCCATGTCGGTAAAGAGGCTCACGTGCTAAGGAAAACATGCGTTTTACGAGATAAACCATTGTATTCATGTCTGGATTCTACGAAACCATTGAATTAATCGATCTGATTGTAATTCATACTTGGACCATAATACATGCAGAATAATCAAGTAGTAAAGGGGCTTGCGAGTCAAAAGACACGTGGCCTCTACCGTCGTGCAATTGAACGAACAAAACAGATTCTTACGTCTGTTCTGTGGCAACATG[illegible]	88%		87%		
scaffold_50	CsQ	12543	290527-303069	CsQ_DRJ1int	383	293120-293502	CAGTAAGGTTGATTGAACAGACCAATGTTGCTGTCCAGGCAGCTTGAGGACTGCTTGTAAAATGATACTCCATGGGATCGTCGCCATCACATCGACCAAGTTTGGATTTCAATAGCAATTGACATAACGATACATTGTAATCCGTTTTTGGAACATATAGTGCTGGCTCCATGTCCTTGTTGCAAAAGAGGCTCACATGCTAAGGAAAACATGCGTTTTACGAGATAAACAATTGTATTCAAGTCGGGATACTACGAAACCATTGATATAATCTGATTGTAATTCATACTCGGACCATAATAGATGCAGAATAGTCAAGTAGTAAAGGGGCTTAGGTGTCAAAAAACACGTGTGCTCTACCGTGGTGGTATTGAACGAACAAA		79%			
scaffold_50	CsQ	12543	290527-303069	CsQ_DRJ1R	408	302662-303069	CAGTAACGTTGATTGAACAGATCAGGGGTGCTGTTGCAGGCAGCTTAAGCAACTTCTTGTAAAATGACAATCCATTTTTAATAGCAATTGAAATAACGACGCATTGTAATCCGTTTTCGGACCATATAGTGCTGGCTCCATGTCCATATCGGAAAAGAGGCTCACGTGCTAAGGAAAACATGCGTTTTACGAGATAAACAATTGTATTCATGCCTGGATTCTACGAAACCATTGAATCAATCGATCTGATTGTAATTCATACTTGGACCATAATACGTGCAGAATAATCAAGTAGTAAAGAGGCTTGCGAGTCAAAAGACACGTAGGCTCTACCGTCGTGCAATTGAACGAACAAAACAGATTCTTACGTCTGCTCTGTGGCAACATGACCTGCAGTTAGGTTATCAT					
scaffold_149	CsU	15338	374074-389411	CsU_DRJ1L	511	374074-374584	AACCGTGGTCATCTTTGTCGCCACCAAGATCTGTCTGTCTCAAAGTGACGCTGACCTCTCAATTAGCTGTTCGAGTTGTTGATGGAAGGATGTGCATTCGAAGTTCCACGCTCTGCAATGTATTATAAGCTCGACTGCGCCATTGGTGGAGGATGATATGGCAGGTCGAAAAGATGGTACCAAGTGACGTGTACTTTTATTTTAAACGACACCACAAGAGGTTATCGTGCGGGTGCTGGTCGGGTCGAACAATGTCCACAAACCACTACCTGTACGATGTGAAGCTTCCACTAGTTTATCACGCAGAGCTGCCGTTCCACGGGATTGCACGGCGCCATCGCTTGCGTTTACTCTATACGTGTACTCGTGCAGTACAAGCTTGATAGCTATGTTCATATGACCACATGCTTGGTGGGCAGTGAAAATATTGCAACAACGACCGCTCCATCTAACAATGTACAAACATGATTCATGTGCTATTGCGATAGTTTTGATGCAGATTGTTCGACTA	96%				
scaffold_149	CsU	15338	374074-389411	CsU_DRJ1R	511	388901-389411	AACCGTGGTCATCTTTGTCGCCACCAAGCTCTGTCTGTCTCAAAGTGACGCTGACCTCTCAATTAGCTGTTCGAGTTGTTGATGGAAGGATGTGCATTCGAAGTTCCACGCTCTGCAATGTATTATAAGCTCGACTGCGTCATTGATGGAGGATGATATGGCAGGTCGAAAAGATGGTACCAAGTGACGTGTACTTTTATTTTAAACGACACCACGAGAGGTTATCGTGCGGGTGCTGGTCGGGTCGAACAATGTCCACAAACCACTACCTGTACGATGTGAAGCTTCCACTAGTTTATCACGCAGAGCTGCCGTTCCACGGGATTGCACGGCGCCATCGCTTGCGCTTACTCTATACGTGTACTCGTGCGGTACAAGCTTGATAGCTATGTTCATATGACCCCATGCTTGGTGGGCAGTGGAAATATTGCTATTACGACCGGTTCATCTAACACTGTACAAACATGATATATATGCTATTGATATAGTTTTGATACAGATTGTTCAACTA					
scaffold_28	CsW	15807	614005-629811	CsW_DRJ1L	223	614005-614227	GATTCTGCTGAATGATTCTACAGGCCATCATCCGTTTAGGTTTGACACTGCGCTCGATGTCCCGTGGTTACCCCGGCTTACAGTACTTAGTTACTAACTACTCAACAGAATGCAAACTTATTTCGATGTTTAGAGTTCTAGCACGAGGAACAGCAGCAGGACGCGGGTAGGTGCTGTAACGGTGTAACGAATGCCTGCATTGCATTTTATGCCATAATCCATC	86%	90% (on 184nt)	77%	77%	
scaffold_28	CsW	15807	614005-629811	CsW_DRJ1int-1	223	616606-616828	GATTCTGCTGGATGAATCTACAGGTCACCATCCGTTTAGGTTCGACACTGTGCTTGATATCCTGTGGTTACCCTGGCTCACAGTAATTAGTTACTAACTAATCGACAGATTGAAAAAGTTTTTCGATGTTCAAAGTTCTAGCATGAGGAACAGCAGCAGAAAGCGGGTAGGTGCTGTAGCCGTGTAATGAATGCCTGCTATGCATTCTGTGCTATAATCCATC					75%
scaffold_28	CsW	15807	614005-629811	CsW_DRJ1int-2	206	619316-619521	GATTCTGCTGGATGAATCTACAGTATGACACTGTGCTTGATGTCCTGTGGTTACCCCGGCTTACATTTCTTAGTTACTAAGTACTCAACAGAAAGAAAACCTATTTCAATGTTTAAAGTTCTAGCACGAAGAACAGCAGCAGGACGCGGGTAGGTGCTGTAACGGTGTAACGAATGGCTGCTATGCATTCTATGCTACAATCCATC	76%				
scaffold_28	CsW	15807	614005-629811	CsW_DRJ1R	223	629589-629811	GATTCTGCTTAATGATTGTACATGTTATCATTCAATTGGGTTTGACCCCGTGTTCGATAACCTGTGGTTCTCCCGACTTACAGTCATTCGTTACAAACCACTCAATCAAATAAAAACTTATTTCGACGTTTAAAATTCTAGCACATGGAACAGCAGCAAGACGCGGGTAGACGCTGTAACCCTTTAGTGACTACCTGCCATGCATTCTCCGCCACGATCCATC					
scaffold_5890	CsZ	15871	134147-150017	CsZ_DRJ1L	99	134147-134245	AAACAATCGCATCAAACGCTCTTGGTTGACGTACAATGTGTGTGTGGATGCAATGGTTCACCGTGGGTCCCAAAGATGGCATTTCGTACGATCTTCAAG	77%				
scaffold_5890	CsZ	15871	134147-150017	CsZ_DRJ1R	97	149921-150017	AAACAGTTGCGTCAAAGACTTTTGGTTGACACTCAATGTGTGTGGATGCAATGGTCTACCGCAGGTCCCATAAGTCGCATACCGTACGTTCTTCGAG					
scaffold_5934	CsX1	17335	19391-36725	CsX1_DRJ1L	214	19391-19604	GTATACACCACCGTACCACTTGGAACGCAAGAACTCTGATGGATTGTGGCAATCTTCCAATAAAGTACCTCGAACTTGTGTTATGGATCTGTATGGACGTGGAACCGTTGCCGTGCAAGATCCTTATAGAACGTTTTATCCATCGACACGTGTGCGTGAGGACGTTCCTCTCAAAGTCGGTGCAATATCATCATGCGATAACGGTTG	76%				

scaffold_5934	CsX1	17335	19391-36725	CsX1_DRJ1int1	216	21903-22118	GTATGCACGTCACGGTACCACTTGGTCCGCAAGCATTATGATGGGTTGTGACAAAGTTCCAGTATTCTACCTCGAACATTTGTTGTCAATCTTTATGGACGTGG<br>GCACGTGGTCGTGCAAAAGCCTTATGAAACGTTTTACTTATCGTAGAACGTGCGCGACGACGTTCGCCACGAAATCAGTGCAATATCATTGTGCGATAACAGT	[illegible]	72%	88%	78%
scaffold_5934	CsX1	17335	19391-36725	CsX1_DRJint2	216	27254-27469	GTATACACGCCACGGTACCACTTGGGCCGTAAGCAGTATGATGGGTTGTGGCAAAGTTCCAGTCTCCTACCTTAAGTATTTGTTGTCAATATTTATGGACGTAG<br>GCACGTGGCCGTGCAAAAGCCTTATAAAACGTTTTAGTCAATGGAGAGGTTTTCTAACGTCGTTTGTCTTGAAGCTGGTGCATTTATCAATGCGAGATGACAGT	85%			
scaffold_5934	CsX1	17335	19391-36725	CsX1_DRJ1R	216	36510-36725	GTATACACGCCACGGTACCACTTGGCCCGTAAGCAGTATGATGGGTTGTGGCAAAGTTCCAGTCTCCTACCTTCAACATTTGTTGTCAATCTTTATGGACGTAG<br>GCACGTAGCCATGCAAAAGCCTTATGAAACGTTTTATTCATCGGAGAGCGTGCGCCACGACGATCCTCTCAAAGTCGGTGCAATATCATCGTGCGATAACAGT				
scaffold_116	CsT	23217	7789-31005	CsT_DRJ1L	638	7789-8426	GTACGCCGCGACGTCACAGTGTTCGACAGTCTAGCCTCCACACCAATTAGCTATTACGAAAGCACCGAAGTAGTTTTTAAGCACTGACTATGAATATTAGCCAT<br>GACTTCACAGCACCGAACATCCAAAGGACGCTGCATATTAGTTTGTTCTTCGATGGGCGGTTGTTTGTAAATGCACTGCTTTCGAGACAGGAAAAGGAAGAGG<br>ATTTCATTAGAAAAGGGTCATACAGATGCTAACTTCCAACGTCAGAATCATTAGTCATATTCTGAAGAGTTCTACAACTTTTGTAATTAATCAATTGAAAAATCGT<br>ACAATATATTGATTTAATAATAAGAAATAACTTGGTTGATATCTTGGTTATTTATAGGTCATTCCAAAAATGCGAATAAAATAAAAATAAATTAGCAAGAAATTTTA<br>ACTCGTAACACACTTTTTACGATCGGATCACTAATTAGTATTAACTCAAGCCTGTTAATTATTTATTTTTTTCCTCAAGATAAAGATTGACATTATAATTTCTGCGA<br>TGGTCGTAAAAGCGCGCATATTTGCTGGTTTCAATTTACGACCCATGTTAACATTTAACACAACGCGACGTATTCGCAGCCTGGTACACTGAACTCTGAAAAGC<br>ATTGTGA	96%			
scaffold_116	CsT	23217	7789-31005	CsT_DRJ1R	640	16673-17312	GTAAGCCGTGACGTCATAGTGTTCCACAGTCTAGCCTCCACACCACTTAGCCATTACGAAAGCACCCAAGTAGTTTTTAAGCACCGACTATGAATATTAGCCAT<br>GACTTCACAGCACCGAATATCCAAAGGACGCTGCATATTAGTTTGTTCTTCGATGGGCGGTTGTTTGTAAATGCACTGCTTTCGAGACAGGAAAAGGAAGAGG<br>ATTTCATTAGAAAAGGGTCATGCAGATGCTAACGTCCAACGTCAGAATCATTAGTCATATTCTGAAGAGTTCTACAACTTTTGTAATTAATCAGTTGAAAAATCG<br>TACAATATATTGATTTAATAATAAGAAATAACTTGGTTGATATCTTGGTTATTTATAGGTCATTCCAAAAATGCGAATAAAATAAAAATAAATTAGCAAGAAATTTT<br>AACTCGTAACACACTTTTTACGATCGTATCACTAATTAGTATTAACTCATGCCTGTTAATTATTTATGGTTTTTTCCTCAAGATAAAGATTGACATTATAATTTCTG<br>CAATGTCCGTAAAAGCGCGCATATTTGCTGGTTTTAATTTACGACCCATGTTTACATTTAACACAACGCGACGTATTTGTATCCTGGTACACTGAACTCTGAAAA<br>GCATTGTGA				
scaffold_116	CsT	23217	7789-31005	CsT_DRJ2L	1093	15405-16497	TACATACATGTAGCGCACAATCGCCTAATTAGATCATCGACATAGAAGCTGACATGGAATAAACGAACTCTATCATCTTGAAGGTTTGGGTTTTTCGAATACATC<br>TAATTCGGTATCGAAGCAGCCAGAACGTCGAACATGTCCATACATAAACCGTGCATTAAACTTGGATGCTGAAAAAGTTGAGAGTGCTTCACTGCCGTACTGC<br>GTATTGTTATATCGTCACGTATATTGTAATACCGGAGTACGACAGTAGCGTTTGCAGTGTCCAAGTCGAATGCACGGGAACCTAACATCATCATCGTTATAATT<br>GAAAAACTCATATATATGCCAGACCATAGATGGCAGAAGCCGTACAGAATTTGAACTTAAAGTACACAAATTACAAGGAGACTTGATGTAATGTCATCACTACC<br>TGAAATGAAAAAAATGGACTGTCCATTCCTATTTTTATCATTTCATCTGTGACATAGCCGTTGGCCGTATCTTGCTGGGTGTACACCTGAACGTGGCGGGCATG<br>TATAAATCATTATTGTTGAAGCGCAGTTCGCTCAACTCAATAGTTGTTGGTTTCAGAGAACTACTTATTAGTGTAAATAAGTGGTAGAATTTTTTTACCGGTTAAA<br>TTTCTTTGCCGTTATGAATTTTATTTCTCAGCGGGCAAGAAGTGAGGCCCGTGAACATTGATACCTATCTTATGCCAGAAGACAACTATGCCACTCTCCAGTAA<br>TGATCACATATCCTACCTAGCTTTTTCGAAAACTGCTGCATCGTTTCTTGTCGGGTGATACAGTCGAGTGATCGTCACTATATTCTGTCATCTTTCAAGCCACAA<br>CGTTGTCATTCTCTGCAAGACCTTTCTAACAATGAATAATGTCGAATGTACACTATGGATGAGGTGAATCCAAGGCTTTTGTGGTTTTGACCGAAGTGTTCCAC<br>GAATGACGGTGACTCTAGGTCAGTACACTTGAATTATTTTGCAGAAATTTCACCGTATACAAAGCGCCTTAAAGAATTTAAAAAAGATTGCAGTGATGATATGTA<br>TCATAGAAGTTTATTTGGTAAGCAATTGCGACAGCAATCTCCAGTGGAA	89%			
scaffold_116	CsT	23217	7789-31005	CsT_DRJ2R	1132	29874-31005	TACATACATGCAGTACACAATCGCCCAATTAGATCATCGACATAGAAGCTGACAAGGAATAAACCAACTCTATCAGCTTGAAGGTTTGGGTTCTTCGAATACAT<br>GTGATTCGGTATCAAAGCAGTCAGAACGTCGAACATGTCCATGCATGAACCGTGCATTTAACTTGGATGCTGAAAAAGTTGAGAGTGCTTCACTGCCGTACTG<br>CGTATTGTTATATCGTCACGTATATTGTGATACCGAAGTACGGCAGTAGCGTTTGCAGTGTCCACGTCGAATGCACGGGAACCTAACATCATCATCGTCATCAT<br>TGAAAAACTCATATATATGCCAGACCATAGATGGCAGAAGCCTGTACAGAATTTGAAGTTAAAGTCCGCAAATTACAAGAAACAGACTTGACGTAATGTCATCA<br>CTATCTGAAATGAATAAATGGACTGTCCATTGCTATTTTTATCATTTAATCTGTGACATAGCCGTTGGCCGTATCTTGCTGGGTACACCTGAACGTGGCGAGCA<br>TGTATAAATCATTATTATTGAAACGCAGTTCGCGCAACTCAATAGTTGTTTGTTTCAGAGAACTACTTATTTATAGAGTATGATTGTGGCATTAAAAAATGCTGGT<br>AGTGTAAATAAGTGGTAGAATTTTTTTACCGGTTAAATTTCTTTGCCGTTATGAATTTTATTTCTCAGCGGGCAAAAAGTGAGGCCCGTGAACATTGATACCTAT<br>CTTATGCCAGAAGACAACTATGCCACTCTCCAGTAATGATCACATATCCTACCTAGCTTTTTCGAAAACTGCTGCATCGTTTTTTGTCGGGTGATACAGTCGAG<br>TGATCGTCACTATATTCTGTCATCTTTCAAGCCACAACGTTGTCATTCTCTGCAAGACCTTTCTAACAATGAATAATGTCGAATGTACACTATGGATGAGGTGAA<br>CCCAAGGCTTTTGTGGTTTTGACCGAAGTGTTCCACCAACGACGGCGACTCTTGGTCGTCGGTACACTTGGATTATTTTGCAGATTTTCTACCGCATACAAGGA<br>ACCTCTAAGCATATTTGGAAAAAAGTGCAGTGACGATGGTACCGTGGAAGCTCATTCGGAAACAATTGCAACAGAAATCTACAGTGGAA				
