# Supplementary material for: Genomic architecture of endogenous ichnoviruses reveals distinct evolutionary pathways leading to virus domestication in parasitic wasps
Source: BMC Biol. 2020 Jul 24;18:89. doi: 10.1186/s12915-020-00822-3 (PMC7379367; doi:10.1186/s12915-020-00822-3)
Supplement: Supplementary file 10 — Additional file 10:. DRJs analysis. Figure S3. Examples of the different types of DRJ position. a. Proviral segment with two copies of a single direct repeat (DRJ1L and DRJ1R), one at each end of the segment. b. Proviral segment with two distinct repeated sequences (DRJ1, in yellow and DRJ2, in green), each present in two copies (DRJ1L and DRJ1R, DRJ2L and DRJ2R). c. Proviral segment with two repeated sequences, each present in two or more copies. DRJ1s in yellow, DRJ2s in green, HdIV genes represented by arrows. Table S11. DNA motifs found in the direct repeated sequences flanking the IV segments inserted in wasp genomes. Analysis was performed using the DNAMINDA2 webserver (http://bmbl.sdstate.edu/DMINDA2/annotate.php); the input dataset was composed of 99 DRJ sequences (right junctions of HdIV and CsIV segments). A total of 89 motifs were obtained; only those whose occurrence exceed 70% of the DRJs are reported. Table S12. Result of genome search using motifs predicted with DMINDA 2.0 webserver. Occurrence rate of motifs predicted with DMINDA 2.0 webserver in DRJs and whole genome sequences. Each of the two motifs was search among the 6 bp kmers present in the whole genome (201,969,604) and in the DRJs (33,930). The significance was evaluated using a Chi2 (taking into account the ratio of these motifs / all the other motifs in the DRJS and in the genome). [file 12915_2020_822_MOESM10_ESM.pdf]

Additional file 10. DRJs analysis.

Figure S3. Examples of the different types of DRJ position. **a.** Proviral segment with two copies of a single direct repeat (DRJ1L and DRJ1R), one at each end of the segment. **b.** Proviral segment with two distinct repeated sequences (DRJ1, in yellow and DRJ2, in green), each present in two copies (DRJ1L and DRJ1R, DRJ2L and DRJ2R). **c.** Proviral segment with two repeated sequences, each present in two or more copies. DRJ1s in yellow, DRJ2s in green, HdIV genes represented by arrows.

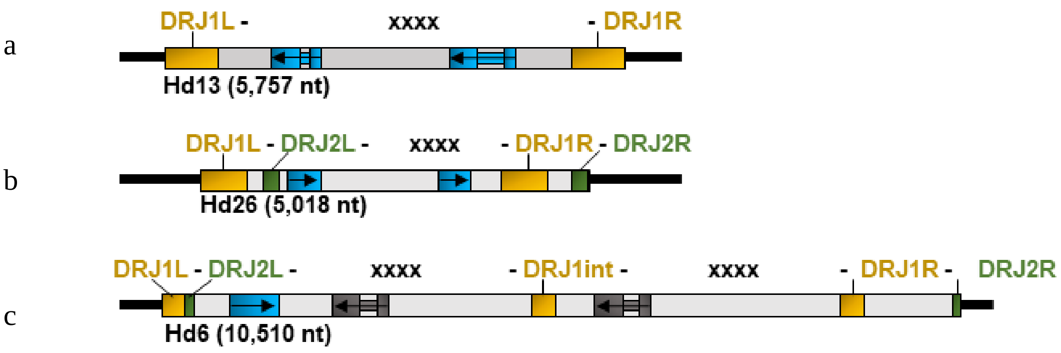

Table S11. DNA motifs found in the direct repeated sequences flanking the IV segments inserted in wasp genomes. Analysis was performed using the DNAMINDA2 webserver (http://bmbl.sdstate.edu/DMINDA2/annotate.php); the input dataset was composed of 99 DRJ sequences (right junctions of HdIV and CsIV segments). A total of 89 motifs were obtained; only those whose occurrence exceed 70% of the DRJs are reported.

Motif	Length (nt)	Consensus logo	Consensus sequence	Nb of occurrence in DRJs (n=99 DRJ sequences)	Nb of DRJs containing at least one motif (/99)
Motif-62	6	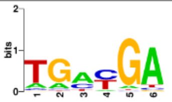	TGAYGA	1231	97
Motif-56	6	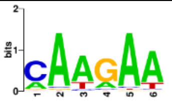	CAAGAA	638	97
Motif-58	6	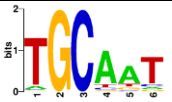	TGCAAT	380	90
Motif-68	7	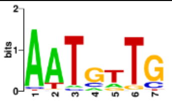	AATGTTG	281	82
Motif-88	9	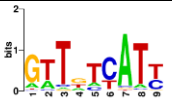	GTTGTCATY	205	76
Motif-10	8	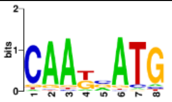	CAATMATG	197	75

Motif-59	6	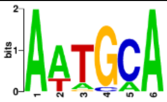	AATGCA	195	73

Table S12. Result of genome search using motifs predicted with DMINDA 2.0 webserver
Occurrence rate of the motif in DRJ and whole genome sequences. Each motif was search among the 6 bp kmers present in the whole genome (201,969,604) and in the DRJs (33,930). The significance was evaluated using a Chi2 (taking into account the ratio of these motifs / all the other motifs in the DRJS and in the genome).

Motif	DRJ	Whole genome	P-value
TC[G,A]TCA	61	291,908	0.1015
CAAGAA	31	169,153	0.6959
