# Supplementary material for: Genomic architecture of endogenous ichnoviruses reveals distinct evolutionary pathways leading to virus domestication in parasitic wasps
Source: BMC Biol. 2020 Jul 24;18:89. doi: 10.1186/s12915-020-00822-3 (PMC7379367; doi:10.1186/s12915-020-00822-3)
Supplement: Supplementary file 11 — Additional file 11 Manual analysis of the DRJ regions containing an excision site. Alignments of Sanger-sequenced DRJ regions from integrated and circular forms of seven H. didymator IV segments containing a putative excision site. [file 12915_2020_822_MOESM11_ESM.pdf]

Additional file 11. Manual analysis of the DRJ regions containing an excision site

A CLUSTAL O(1.2.4) multiple sequence alignment was performed on the DRJs. The sequences used for alignment have 3 different origins: (i) DRJs from *H. didymator* genome; (ii) DRJs from *H. didymator* BAC clones; (iii) HdIV segments, e.g. PCR products (cloned in pGEM and Sanger sequenced) from encapsidated HdIV DNA template.

Color code:
DRJ1R (or right junction) underlined in dark grey; DRJ1L (or left junction) underlined in light grey
Nucleotides that differ between DRJ1R and DRJ1L are indicated in red (in blue, differences between genome and BAC sequences)
Regions in the segment sequence where potentially occurred a switch between right/left junctions are underlined.

Segment Hd30
Sequences used for alignment:
(i) DRJs from genome: Hd30_DRJ1R, Hd30_DRJ1L
(ii) DRJs from BAC clone # AB-06P08: right_AB, left_AB
(iii) HdIV segments: Contig_AB-20, Contig_AB-17, Contig_AB-16; plus sequence SH_AB previously sequenced

Alignment

```
left_AB           AGCCGACGTGATATATGCTGAAAACAGGTCTGACTCGCTACGAGGCTAGTTGGTTGGTCC
Hd30_DRJ1L        AGCCGACGTGATATATGCTGAAAACAGGTCTGACTCGCTACGAGGCTAGTTGGTTGGTCC
Contig_AB-17      AGTCAACATGGTGTATGTTCAGACCAAGTCTCCCTCGCTACGAGGCTAGTTGGTTGGTCC
Contig_AB-20      AGTCAACATGGTGTATGTTCAGACCAAGTCTCCCTCGCTACGAGGCTAGTTGGTTGGTCC
SH_AB             AGTCAACATGGTGTATGTTCAGACCAAGTCTCCCTCGCTACGAGGCTAGTTGGTTGGTCC
Contig_AB-16      AGTCAACATGGTGTATGTTCAGACCAAGTCTCCCTCGCTACGAGGCTAGTTGGTTGGTCC
right_AB          AGTCAACATGGTGTATGTTCAGACCAAGTCTCCCTCGCTACGAGGCTAGTTGGTTGGTCC
Hd30_DRJ1R        AGTCAACATGGTGTATGTTCAGACCAAGTCTCCCTCGCTACGAGGCTAGTTGGTTGGTCC
                  ** * ** ** * **** * * * ** ****  ***************************

left_AB           ATCAGTGCATTGCACGCTCGCTACGGGTTGGCACGCAGCATCGTGCCACAGCTGCAACTC
Hd30_DRJ1L        ATCAGTGCATTGCACGCTCGCTACGGGTTGGCACGCAGCATCGTGCCACTGCTGCAACTC
Contig_AB-17      ATCAGTGCATTGCACGCTCGCTACGGGTTGGCACGCAGCATCGTGCCACTGCTGCAACTC
Contig_AB-20      ATCCGTGCATTGCACGCTCGCTGCGGGTTGGCACCCAGCATCGTGCCACTACTGCGACTC
SH_AB             ATCCGTGCATTGCACGCTCGCTGCGGGTTGGCACCCAGCATCGTGCCACTACTGCGACTC
Contig_AB-16      ATCCGTGCATTGCACGCTCGCTGCGGGTTGGCACCCAACATCGTGCCACTACTGCGACTC
right_AB          ATCCGTGCATTGCACGCTCGCTGCGGGTTGGCACCCAGCATCGTGCCACTACTGCGACTC
Hd30_DRJ1R        ATCCGTGCATTGCACGCTCGCTGCGGGTTGGCACCCAGCATCGTGCCACTACTGCGACTC
                  *** ****************** *********** ** ***********  **** ****

left_AB           CAGCACCTTACAAACTGCAGCGATAATACACTTCGTGCTGTGGAGCACGGTGTCAACGGTGTTC
Hd30_DRJ1L        CAGCACCTTACAAACTGCAGCGATAATACACTTCGTGCTGTGGAGCACGGTGTCAACGGTGTTC
Contig_AB-17      CAACACCTTACAAACTGCAGCGATAATACACTTCGTGCTGTGGAGCACGGTGTCAACGGTGTTC
Contig_AB-20      CGGCACCTTACGAGCTGCAGCGATAAGACACTTCGTGCTGTGGAGCACGGTGTCAACGGTGTTC
SH_AB             CGGCACCTTACGAGCTGCAGCGATAAGACACTTCGTGCTGTGGAGCACGGTGTCAACGGTGTTC
Contig_AB-16      CGGCACCTTACAAACTGCAGCGATAATACACTTCGTGCTGTGGAGCACGGTGTCAACGGTGTTC
right_AB          CGGCACCTTACGAGCTGCAGCGATAAGACACTTCGTGCTGTGGAGCACGGTGTCAACGGTGTTC
Hd30_DRJ1R        CGGCACCTTACGAGCTGCAGCGATAAGACACTTCGTGCTGTGGAGCACGGTGTCAACGGTGTTC
                  *  ******** * ************ *************************************
```

Segment Hd12

Sequences used for alignment:

(i) DRJs from genome: Hd12_DRJ1R, Hd12_DRJ1L

(ii) DRJs from BAC clone # BG-42L09+BL-56G14): left_jct_BGBL1, right_jct_BGBL1

(iii) HdIV segments: Contig_BGBL1-11, Contig_BGBL1-16, Contig_BGBL1-19

Alignment

```
left_jct_BGBL1      --AGTACGGTAATTGCCGCCAGTCTTTGGCGTTATACTGTTTGCGGCTTATCTGGAGTTG
Hd12_DRJ1L          ATAGTACGATAATTGCCGCCAGTCTTTGGCGTACTACTGTTTACGGCTTATCTGGAGTTG
Contig_BGBL1-19     --AGAACGCTTATCACCACCAATCTTTGGCGTCATACTGTTTGCGGCTTATCTGCAGTTG
Contig_BGBL1-11     --AGAACGCTTATCACCACCAATCTTTGGCGTCATACTGTTTGCGGCTTATCTGCAGTTG
Contig_BGBL1-16     --AGAACGCTTATCACCACCAATCTTTGGCGTCATACTGTTTGCGGCTTATCTGCAGTTG
right_jct_BGBL1     --AGAACGCTTATCACCACCAATCTTTGGCGTCATACTGTTTGCGGCTTATCTGCAGTTG
Hd12_DRJ1R          ATAGAACGCTTATCACCACCAATCTTTGGCGTCATACTGTTTGCGGCTTATCTGCAGTTG
                      ** *** * **  ** *** **********  ******** *********** *****

left_jct_BGBL1      TCACTCATGGGTCAAGTTAAGAAAAACCTCATTTAGATGTCTCATATTCCTGTATGGTCT
Hd12_DRJ1L          TCACTCATGAGTCAAGTTAAGAAAAACCTCATTTAGATGTCTCATATTCCTGTATGGTCT
Contig_BGBL1-19     GTACTTATGGATCAAGTTGAGGAAAACCTTATCCAGGCGTCTCATACCCTTGTATGGTCT
Contig_BGBL1-11     GTACTTATGGATCAAGTTGAGGAAAACCTTATCCAGGCGTCTCATACCCTTGTATGGTCT
Contig_BGBL1-16     GTACTTATGGATCAAGTTGAGGAAAACCTTATCCAGGCGTCTCATACCCTTGTATGGTCT
right_jct_BGBL1     GTACTTATGGATCAAGTTGAGGAAAACCTTATCCAGGCGTCTCATACCCTTGTATGGTCT
Hd12_DRJ1R          GTACTTATGGATCAAGTTGAGGAAAACCTTATCCAGGCGTCTCATACCCTTGTATGGTCT
                      *** ***  ******* ** ******* **  **  ********  * **********

left_jct_BGBL1      GCTCCGTACCTAAACGTCGCGGGTACACGTACTCAGAAAGGAATGCACACCCAGGCCTTG
Hd12_DRJ1L          GCTCCGTACCTAAACGTCGCGGGTACACGTACTCAGAAAGGAATGCACACCCAGGCCTTG
Contig_BGBL1-19     GCTCCGTAACTAAACGTCGCGGGTACACGTACTCAGAAAGGAATGCACACCCAGGCCTTG
Contig_BGBL1-11     GCTCCGTACCTAAACGTCGCGGGTACACGTACTCAGAAAGGAATGCACACCCAGGCCTTG
Contig_BGBL1-16     GCTCCGTACCTAAACGTCGCGGGTACACGTACTCAGAAAGGAATGCACACCCAGGCCTTG
right_jct_BGBL1     GCTCCGTAACTAAACATCGCAGGTACACATACTCAGAGAGTAATCCACACTCAGACCTTA
Hd12_DRJ1R          GCTCCGTAACTGAACATCGCAGGTACACATACTCAGAGAGTAATCCACACTCAGACCTTA
                    ******** ** *** **** ******* ******** ** *** ***** *** ****

left_jct_BGBL1      CTCAAGACATATGATGAGCATATTCGTAAGCATAGTTTGCGATATACAACGGAGCTTTGC
Hd12_DRJ1L          CTCAAGACATATGATGAGCATATTCGTAAGCATAGTTTGCGATATACAACGGAGCTTTGC
Contig_BGBL1-19     CTCAGGACATATGATGAGCATATTCGTAAGCACAGTTTGCGATATACAACGGAGCTTTGC
Contig_BGBL1-11     CTCAAGACATATGATGAGCATATTCGTAAACATAGTTTGCGATATACAACGGAGCTTTGC
Contig_BGBL1-16     CTCAAGACATATGATGAGCATATTCGTAAGCATAGTTTGCGATATACAACGGAGCTTTGC
right_jct_BGBL1     CTCAAGACATATGATGGGCATATTCATAAGCATAGTTTGTGATATATAACGGAGCTTTGC
Hd12_DRJ1R          CTCAAGACATATGATGGGCATATTCATAAGCATAGTTTGTGATATATAACGGAGCTTTGC
                    **** *********** ******** *** ** ****** ****** *************

left_jct_BGBL1      ATCCCATGTGCACATAACACCATCAGAGATGAACGGGGAGATCTCCAATGCTATAAGGCC
Hd12_DRJ1L          ATCCCATGTGCACATAACTCCATCAGAGATGAACGGGGAGATCTCCAATGCTATAAGGCC
Contig_BGBL1-19     ATCCCATGTGCACATAACACCATCAGAGATGAACGGGGAGATCTCCAATGCTATAAGGCC
Contig_BGBL1-11     ATCCCATGTGCACATAACACCATCAGAGATGAACGGGGAGATCTCCAATGCTATAAGGCC
Contig_BGBL1-16     ATTTCATGTGCACATAACACCATCAGAGATGAACGGGGAGATCTCCAATGCTATAAGGCC
right_jct_BGBL1     ATCCCATGTGCACATAACTCTAACAGAGATAAGTAGGGAGATCTCCAATGCTATAGGGCT
Hd12_DRJ1R          ATCCCATGTGCACATAACTCTAACAGAGATAAGTAGGGAGATCTCCAATGCTATAGGGCT
                    **  ************** * * ******* *   ******************** ***

left_jct_BGBL1      CTAGTGGTTCCTACAAAGCGACGGTTTTACGTATCGGTCAACTGCTCCAGCTGTCATAGG
Hd12_DRJ1L          CTAGTGGTTCCTACAAAGCGACGGTTTTACGTATCGGTCAACTGCTCCAGCTGTCATAGG
Contig_BGBL1-19     CTAGTGGTTCCTACAAAGCGACGGTTTTACGTATCGGTCAACTGCTCCAGCTGTCATAGG
Contig_BGBL1-11     CTAGTGGTTCCTACAAAGCGACGGTTTTACGTATCGGTCAACTGCTCCAGCTGTCATAGG
Contig_BGBL1-16     CTAGTGGTTCCTACAAAGCGACGGTTTTACGTATCGGTCAACTGCTCCAGCTGTCATAGG
right_jct_BGBL1     CGAGTGGTTCCTACAGAGCGGCGCTTTTTCTTATCGGTCAACTAATAAAGCTGTCTTAGA
Hd12_DRJ1R          CGAGTGGTTCCTACAGAGCGGCGCTTTTTCTTATCGGTCAACTAATAAAGCTGTCTTAGA
                    * ************* **** ** **** * ************  *  ******* ***

left_jct_BGBL1      TGTTTGTTGTTGCTCGGTCGTGCTTGCAGTAATCCATCCTGCTCGGCAGAGA
Hd12_DRJ1L          TGTTTGTTGTTGCTCGGTCGTGCTTGCAGTAATCCATCCTGCTCGGCAGAGA
Contig_BGBL1-19     TGTTTGTTGTTGCTCGGTCGTGCTTGCAGTAATCCATCCTGCTCGGCAGAGA
Contig_BGBL1-11     TGTTTGTTGTTGCTCGGTCGTGCTTGCAGTAATCCATCCTGCTCGGCAGAGA
Contig_BGBL1-16     TGTTTGTTGTTGCTCGGTCGTGCTTGCAGTAATCCATCCTGCTCGGCAGAGA
right_jct_BGBL1     TGTTTTT--TTGCTCGGTCGTGCTCGCACAAATCCATCCTGCTCGGCAAAGA
Hd12_DRJ1R          TGTTTTTT-TTGCTCGGTCGTGCTCGCACAAATCCATCCTGCTCGGCAGAGA
                    ***** *  *************** ***  ****************** ***
```

Segment Hd16

Sequences used for alignment:

(i) DRJs from genome: Hd16_DRJ1R, Hd16_DRJ1L

(ii) DRJs from BAC clone # BG-42L09+BL-56G14: left_jct_BGBL2, right_jct_BGBL2

(iii) HdIV segments: Contig_BGBL2-12, Contig_BGBL2-16, Contig_BGBL2-19, SH_BGBL2

Alignment

```
left_jct_BGBL2       ------------------------------------------------------------
Hd16_DRJ1L           ------------------------------------------------------------
Contig_BGBL2-19      ATAGAGTTGTCATGAGCGTAACTACATTTAAACTTGAAGCACTGTAAGAAGGAACGGTCT
Contig_BGBL2-12      ATAGAGTTGTCATGAGCGTAACTACATTTAAACTTGAAGCACTGTAAGAAGGAACGGTCT
Contig_BGBL2-16      ATAGAGTTGTCATGAGCGTAACTACATTTAAACTTGAAGCACTGTAAGAAGGAACGGTCT
SH_BGBL2             ATAGAGTTGTCATGAGCGTAACTACATTTAAACTTGAAGCACTGTAAGAAGGAACGGTCT
right_jct_BGBL2      ATAGAGTTGTCATGAGCGTAACTACATTTAAACTTGAAGCACTGTAAGAAGGAACGGTCT
Hd16_DRJ1R           ------TTGTCATGAGCGTAACTACATTTAAACTTGAAGCACTGTAAGAAGGAACGGTCT


left_jct_BGBL2       ------------------------------ATAAAATTGTCATGAGAG-----GAACTAA
Hd16_DRJ1L           ------------------------------------TTGTCATGAGAG-----GAACTAA
Contig_BGBL2-19      CACAGTACCTTACCTACACAAGTCTCCATCATCCTACACTAATAAGGATAAGAAAAATAA
Contig_BGBL2-12      CACAGTACCTTACCTACACAAGTCTCCATCATCCTACACTAATAAGGATAAGAAAAATAA
Contig_BGBL2-16      CACAGTACCTTACCTACACAAGTCTCCATCATCCTACACTAATAAGGATAAGAAAAATAA
SH_BGBL2             CACAGTACCTTACCTACACAAGTCTCCATCATCCTACACTAATAAGGATAAGAAAAATAA
right_jct_BGBL2      CACAGTACCTTACCTACACAAGTCTCCATCATCCTACACTAATAAGGATAAGAAAAATAA
Hd16_DRJ1R           CACAGTACCTTACCTACACAAGTCTCCATCATCCTACACTAATAAGGATAAGAAAAATAA
                                                            * ** **        ** ***

left_jct_BGBL2       ATTTAAACTTGTACCAACGTAAGAGG------AAACGCTCTCACAGTACC-TTTAGCTGC
Hd16_DRJ1L           ATTTAAACTTGTACCAACGTAAGAGG------AAACGCTCTCACAGTACC-TTTAGCTGC
Contig_BGBL2-19      AAGTAAACACATTCAACACAAACTTTTATTTCAATCTAAGGAACAGTATATGCTGCACTA
Contig_BGBL2-12      AAGTAAACACATTCAACACAAACTTTTATTTCAATCTAAGGAACAGTATATGCTGCACTA
Contig_BGBL2-16      AAGTAAACACATTCAACACAAACTTTTATTTCAATCTAAGGAACAGTATATGCTGCACTA
SH_BGBL2             AAGTAAACACATTCAACACAAACTTTTATTTCAATCTAAGGAACAGTATATGCTGCACTA
right_jct_BGBL2      AAGTAAACACATTCAACACAAACTTTTATTTCAATCTAAGGAACAGTATATGCTGCACTA
Hd16_DRJ1R           AAGTAAACACATTCAACACAAACTTTTATTTCAATCTAAGGAACAGTATATGCTGCACTA
                     *  *****   * * *    **          ** *      ******     *

left_jct_BGBL2       AAGCTTTAGATTAGATTCAACTGCACCATGGCCGGAACTGTTGCGAGCCGAGTCAAGCAA
Hd16_DRJ1L           AAGCTTTAGATTAGATTCAACTGCACCATGGCCGGAACTGTTGCGAGCCGAGTCAAGCAA
Contig_BGBL2-19      TTAACACTACTCAGATTAAGCTGCACCATGGCCGGAACTGTTGCGAGCCGAGTCAAGCAA
Contig_BGBL2-12      TTAACACTACTCAGATTAAGCTGCACCATGGCCGGAACTGTTGCGAGCCGAGTCAAGCAA
Contig_BGBL2-16      TTAACACTACTCAGATTAAGCTGCACCATGGCCGGAACTGTTGCGAGCCGAGTCAAGCAA
SH_BGBL2             TTAACACTACTCAGATTAAGCTGCACCATGGCCGGAACTGTTGCGAGCCGAGTCAAGCAA
right_jct_BGBL2      TTAACACTACTCAGATTAAGCTGCACCATGGCCGGAACTGTTGCGAGCCGAGTCAAGCAA
Hd16_DRJ1R           TTAACACTACTCAGATTAAGCTGCACCATGGCCGGAACTGTTGCGAGCCGAGTCAAGCAA
                               * ***** * ****************************************

left_jct_BGBL2       CACTTCTGTGTCCGTACGTTCAGCCATAGAATAATATTCCAAGACGTCTGGATTGTTTTG
Hd16_DRJ1L           CACTTCTGTGTCCGTACGTTCAGCCATAGAATAATATTCCAAGACGTCTGGATTGTTTTG
Contig_BGBL2-19      CACTTCTGTGTCCGTACGTTCAGCCATAGAATAATATTCCAAGACGTCTGGATTGTTTTG
Contig_BGBL2-12      CACTTGTGTGTCCGTACGTTCAGTCATGGAATAATATTCCAAGACGTCTGGATTGTTTTG
Contig_BGBL2-16      CACTTGTGTGTCCGTACGTTCAGTCATGGAATAATATTCCAAGACGTCTGGATTGTTTTG
SH_BGBL2             CACTTGTGTGTCCGTACGTTCAGCCATGGAATAATATTCCAAGACGTCTGGATTGTTTTG
right_jct_BGBL2      CACTTGTGTGTCCGTACGTTCAGCCATGGAATAATATTCCAAGACGTCTGGATTGTTTTG
Hd16_DRJ1R           CACTTGTGTGTCCGTACGTTCAGCCATGGAATAATATTCCAAGACGTCTGGATTGTTTTG
                     ***** ***************** *** ********************************

left_jct_BGBL2       ATGCACATTTGCAATGATCTCAGTGAACATCTCTTTAGATTCTTTAAGAAGAATCGCGGG
Hd16_DRJ1L           ATGCACATTTGCAATGATCTCAGTGAACATCTCTTTAGATTCTTTAAGAAGAATCGCGGG
Contig_BGBL2-19      ATGCACATTTGCAATGATCTCAGTGAACATCTCTTTAGATTCTTTAAGAAGAATCGCGGG
Contig_BGBL2-12      ATGCACATTTGCAATGATCTCAGTGAACATCTCTTTAGATTCTTTAAGAAGAATCGCGGG
Contig_BGBL2-16      ATGCACATTTGCAATGATCTCAGTGAACATCTCTTTAGATTCTTTAAGAAGAATCGCGGG
SH_BGBL2             ATGCACATTTGCAATGATCTCAGTGAACATCTCTTTAGATTCTTTAAGAAGAATCGCGGG
right_jct_BGBL2      ATGCACATTTGCAATGATCTCAGTGAACATCTCTTTAGATTCTTTAAGAAGAATCGCGGG
Hd16_DRJ1R           ATGCACATTTGCAATGATCTCAGTGAACATCTCTTTAGATTCTTTAAGAAGAATCGCGGG
                     ************************************************************
```

```
left_jct_BGBL2       GATCATATATTTAGTCAACCATGCGGAGACGTGCGCCGTACAGAAATGATGCCAGTGGAG
Hd16_DRJ1L           GATCATATATTTAGTCAACCATGCGGAGACGTGCGCCGTACAGAAATGATGCCAGTGGAG
Contig_BGBL2-19      GATCATATATTTAGTCAACCATGCGGAGACGTGCGCCGTACAGAAATGATGCCAGTGGAG
Contig_BGBL2-12      GATCATATATTTAGTCAACCATGCGGAGACGTGCGCCGTACAGAAATGATGCCAGTGGAG
Contig_BGBL2-16      GATCATATATTTAGTCAACCATGCGGAGACGTGCGCCGTACAGAAATGATGCCAGTGGAG
SH_BGBL2             GATCATATATTTAGTCAACCATGCGGAGACGTGCGCCGTACAGAAATGATGCCAGTGGAG
right_jct_BGBL2      GATCATATATTTAGTCAACCATGCGGAGACGTGCGCCGTACAGAAATGATGCCAGTGGAG
Hd16_DRJ1R           GATCATATATTTAGTCAACCATGCGGAGACGTGCGCCGTACAGAAATGATGCCAGTGGAG
                     ************************************************************

left_jct_BGBL2       TTCGTTCTTACACTCACGTCCAGGTGAAACCACTTTATTGGGTTTTTTCTCGCAGGAGCA
Hd16_DRJ1L           TTCGTTCTTACACTCACGTCCAGGTGAAACCACTTTATTGGGTTTTTTCTCGCAGGAGCA
Contig_BGBL2-19      TTCGTTCTTACACTCACGTCCAGGTGAAACCACTTTATTGGGTTTTTTCTCGCAGGAGCA
Contig_BGBL2-12      TTCGTTCTTGCACTCACGTCCAGGTGAAACCACTTTATTGGGTTTTTTCTCGCAGGAGCA
Contig_BGBL2-16      TTCGTTCTTGCACTCACGTCCAGGTGAAACCACTTTATTGGGTTTTTTCTCGCAGGAGCA
SH_BGBL2             TTCGTTCTTGCACTCACGTCCAGGTGAAACCACTTTATTGGGTTTTTTCTCGCAGGAGCA
right_jct_BGBL2      TTCGTTCTTGCACTCACGTCCAGGTGAAACCACTTTATTGGGTTTTTTCTCGCAGGAGCA
Hd16_DRJ1R           TTCGTTCTTGCACTCACGTCCAGGTGAAACCACTTTATTGGGTTTTTTCTCGCAGGAGCA
                     ********* **************************************************

left_jct_BGBL2       GGTGTCGCTGGCGACCGCCTTGCACTTCTGGAAGCGAATCTTCTTCACGAACTGTAGTAT
Hd16_DRJ1L           GGTGTCGCTGGCGACCGCCTTGCACTTCTGGAAGCGAATCTTCTTCACGAACTGTAGTAT
Contig_BGBL2-19      GGTGTCGCTGGCGACCGCCTTGCACTTCTGGAAGCGAATCTTCTTCACGAACTGTAGTAT
Contig_BGBL2-12      GGTGTCGCTGGCGACCGCCTTGCACTTCTGGAAGCGAATGTTCTTCACGAACTGTAGTAT
Contig_BGBL2-16      GGTGTCGCTGGCGACCGCCTTGCACTTCTGGAAGCGAATGTTCTTCACGAACTGTAGTAT
SH_BGBL2             GGTGTCGCTGGCGACCGCCTTGCACTTCTGGAAGCGAATGTTCTTCACGAACTGTAGTAT
right_jct_BGBL2      GGTGTCGCTGGCGACCGCCTTGCACTTCTGGAAGCGAATGTTCTTCACGAACTGTAGTAT
Hd16_DRJ1R           GGTGTCGCTGGCGACCGCCTTGCACTTCTGGAAGCGAATGTTCTTCACGAACTGTAGTAT
                     *************************************** ********************

left_jct_BGBL2       TACGGGCAGCCTCACAAAATCTCTCTCACCCGGCGGCCGCTCCCAAAATAGAGGCATGAG
Hd16_DRJ1L           TACGGGCAGCCTCACAAAATCTCTCTCACCCGGCGGCCGCTCCCAAAATAGAGGCATGAG
Contig_BGBL2-19      TACGGGCAGCCTCACAAAATCTCTCTCACCCGGCGGCCGCTCCCAAAATAGAGGCATGAG
Contig_BGBL2-12      TACGGGCAGCCTCACAAAATCTCTCTCACCCGGCGGCCGCTCCCAAAATAGAGGCATGAG
Contig_BGBL2-16      TATGGGCAGCCTCACAAAATCTCTCTCACCCGGCGGCCGCTCCCAAAATAGAGGCGTGAG
SH_BGBL2             TACGGGCAGCCTCACAAAATCTCTCTCACCCGGCGGCCGCTCCCAAAATAGAGGCGTGAG
right_jct_BGBL2      TACGGGCAGCCTCACAAAATCTCTCTCACCCGGCGGCCGCTCCCAAAATAGAGGCGTGAG
Hd16_DRJ1R           TACGGGCAGCCTCACAAAATCTCTCTCACCCGGCGGCCGCTCCCAAAATAGAGGCGTGAG
                     ** **************************************************** ****

left_jct_BGBL2       CCAGTCCCAATTTATCAGCAGGCGATCTTCTTCTGGCCTTGTTGCATCGAAGCTGTAGCG
Hd16_DRJ1L           CCAGTCCCAATTTATCAGCAGGCGATCTTCTTCTGGCCTTGTTGCATCGAAGCTGTAGCG
Contig_BGBL2-19      CCAGTCCCAATTTATCAGCAGGCGATCTTCTCCTGGCCTTGTTGCATCGAAGCTGTAGCG
Contig_BGBL2-12      CCAGTCCCAATTTATCAGCAGGCGATCTTCTTCTGGCCTTGTTGCATCGAAGCTGTAGCG
Contig_BGBL2-16      CCAGTCCCAATTTATCAGCAGGCGATCTTCTTCTGGCCTTGTTGCATCGAAGCTGTAGCG
SH_BGBL2             CCAGTCCCAATTTATCAGCAGGCGATCTTCTTCTGGCCTTGTTGCATCGAAGCTGTAGCG
right_jct_BGBL2      CCAGTCCCAATTTATCAGCAGGCGATCTTCTTCTGGCCTTGTTGCATCGAAGCTGTAGCG
Hd16_DRJ1R           CCAGTCCCAATTTATCAGCAGGCGATCTTCTTCTGGCCTTGTTGCATCGAAGCTGTAGCG
                     ******************************* ****************************

left_jct_BGBL2       GACGGGGCATGCCTTCCCGTTCAGGAAAGTCAAGTCCAGGTTTCGGATCGATGTAAGAAA
Hd16_DRJ1L           GACGGGGCATGCCTTCCCGTTCAGGAAAGTCAAGTCCAGGTTTCGGATCGATGTAAGAAA
Contig_BGBL2-19      GACGGGGCATGCCTTCCCGTTCAGGAAAGTCAAGTCCAGGTTTCGGATCGATGTAAGAAA
Contig_BGBL2-12      GACGGGGCATGCCTTCCCGTTCAGGAAAGTCAAGTCCAGGTTTCGGATCGATGTAAGAAA
Contig_BGBL2-16      GACGGGGCATGCCTTCCCGTTCAGGAAAGTCAAGTCCAGGTTTCGGATCGATGTAAGAAA
SH_BGBL2             GACGGGGCATGCCTTCCCGTTCAGGAAAGTCAAGTCCAGGTTTCGGATCGATGTAAGAAA
right_jct_BGBL2      GACGGGGCATGCCTTCCCGTTCAGGAAAGTCAAGTCCAGGTTTCGGATCGATGTAAGAAA
Hd16_DRJ1R           GACGGGGCATGCCTTCCCGTTCAGGAAAGTCAAGTCCAGGTTTCGGATCGATGTAAGAAA
                     ************************************************************

left_jct_BGBL2       CTGTTGATATGCTACGTTGAAACGTATAAATTCGATGAAGCTGAAAGGGGCGCCATTCGG
Hd16_DRJ1L           CTGTTGATATGCTACGTTGAAACGTATAAATTCGATGAAGCTGAAAGGGGCGCCATTCGG
Contig_BGBL2-19      CTGTTGATATGCTACGTTGAAACGTATAAATTCGATGAAGCTGAAAGGGGCGCCATTCGG
Contig_BGBL2-12      CTGTTGATATGCTACGTTGAAACGTATAAATTCGATGAAGCTGAAAGGGGCGCCATTCGG
Contig_BGBL2-16      CTGTTGATATGCTACGTTGAAACGTATAAATTCGATGAAGCTGAAAGGGGCGCCATTCGG
SH_BGBL2             CTGTTGATATGCTACGTTGAAACGTATAAATTCGATGAAGCTGAAAGGGGCGCCATTCGG
right_jct_BGBL2      CTGTTGATATGCTACGTTGAAACGTATAAATTCGGCGAAGTTGAAAGGGGCGCCATTCGG
Hd16_DRJ1R           CTGTTGATATGCTACGTTGAAACGTATAAATTCGGCGAAGTTGAAAGGGGCGCCATTCGG
                     **********************************  **** *******************
```

```
left_jct_BGBL2      CATGGCAGGCCCGCGGAACGTGAAGGTGTCCACCGGGTAAGTATGTGTTCTGTCGTGATC
Hd16_DRJ1L          CATGGCAGGCCCGCGGAACGTGAAGGTGTCCACCGGGTAAGTATGTGTTCTGTCGTGATC
Contig_BGBL2-19     CATGGCAGGCCCGCGGAACGTGAAGGTGTCCACCGGGTAAGTATGTGTTCTGTCGTGATC
Contig_BGBL2-12     CATGGCAGGCCCGCGGAACGTGAAGGTGTCCACCGGGTAAGTATGTGTTCTGTCGTGATC
Contig_BGBL2-16     CATGGCAGGCCCGCGGAACGTGAAGGTGTCCACCGGGTAAGTATGTGTTCTGTCGTGATC
SH_BGBL2            CATGGCAGGCCCGCGGAACGTGAAGGTGTCCACCGGGTAAGTATGTGTTCTGTCGTGATC
right_jct_BGBL2     CATGGCAGGCCCGCGGGACGTGAAGGTGTCCACCGGGTAAGTAAGTGTTCTGTCGTGATC
Hd16_DRJ1R          CATGGCAGGCCCGCGGGACGTGAAGGTGTCCACCGGGTAAGTAAGTGTTCTGTCGTGATC
                    **************** ************************** ****************

left_jct_BGBL2      TTCGTTGAACGGCATGTTGAGTTCTGGATATCACCTGCGTCTGGACGATGTTCCTTTGTG
Hd16_DRJ1L          TTCGTTGAACGGCATGTTGAGTTCTGGATATCACCTGCGTCTGGACGATGTTCCTTTGTG
Contig_BGBL2-19     TTCGTTGAACGGCATGTTGAGTTCTGGATATCACCTGCGTCTGGACGATGTTCCTTTGTG
Contig_BGBL2-12     TTCGTTGAACGGCATGTTGAGTTCTGGATATCACCTGCGTCTGGACGATGTTCCTTTGTG
Contig_BGBL2-16     TTCGTTGAACGGCATGTTGAGTTCTGGATATCACCTGCGTCTGGACGATGTTCCTTTGTG
SH_BGBL2            TTCGTTGAACGGCATGTTGAGTTCTGGATATCACCTGCGTCTGGACGATGTTCCTTTGTG
right_jct_BGBL2     TTCGTGGAACGGCATGTTGAGTTCTGGATATCACCTGCGTCTGGATGATGTTCCTTCGTG
Hd16_DRJ1R          TTCGTGGAACGGCATGTTGAGTTCTGGATATCACCTGCGTCTGGATGATGTTCCTTCGTG
                    ***** *************************************** ********** ***

left_jct_BGBL2      AATGTTCACGGTTGAGTGTCTAGCCGTCGACTGCGCGCAAATCCATTTATACCACTCCAA
Hd16_DRJ1L          AATGTTCACGGTTGAGTGTCTAGCCGTCGACTGCGCGCAAATCCATTTATACCACTCCAA
Contig_BGBL2-19     AATGTTCACGGTTGAGTGTCTAGCCGTCGACTGCGCGCAAATCCATTTATACCACTCCAA
Contig_BGBL2-12     AATGTTCACGGTTGAGTGTCTAGCCGTCGACTGCGCGCAAATCCATTTATACCACTCCAA
Contig_BGBL2-16     AATGTTCACGGTTGAGTGTCTAGCCGTCGACTGCGCGCAAATCCATTTATACCACTCCAA
SH_BGBL2            AATGTTCACGGTTGAGTGTCTAGCCGTCGACTGCGCGCAAATCCATTTATACCACTCCAA
right_jct_BGBL2     AATGTTCACGGTTGAGTGTCTAGCCGTCGACTGCGCGCAAATCCATTTATACCACTCCAG
Hd16_DRJ1R          AATGTTCACGGTTGAGTGTCTAGCCGTCGACTGCGCGCAAATCCATTTATACCACTCCAG
                    ***********************************************************

left_jct_BGBL2      TAGCTTATCAT
Hd16_DRJ1L          TAGCTTATCAT
Contig_BGBL2-19     TAGCTTATCAT
Contig_BGBL2-12     TAGCTTATCAT
Contig_BGBL2-16     TAGCTTATCAT
SH_BGBL2            TAGCTTATCAT
right_jct_BGBL2     TAATTTATCAT
Hd16_DRJ1R          TAATTTATCAT
                    **  *******
```

<u>Segment Hd29 (present in BAC clone # BR-08O01)</u>

Sequences used for alignment:

(i) DRJs from genome: Hd29_DRJ1R, Hd29_DRJ1L

(ii) DRJs from BAC clone # BR-08O01: left_jct_BR, right_jct_BR

(iii) HdIV segments: Contig_BR-1, Contig_BR-4, Contig_BR-7, Contig_BR-10, SH_BR

Alignment

```
left_jct_BR      -----------------------------------TAGCGATCGCAGTGCGTCGCTGCAT
Hd29_DRJ1L       ATTTCGTGGCAGGCTTAGTT-TTGATGGAGGGACGTAGCGATCGCAGTGCGTCGCTGCAT
Contig_BR-10     -----------------------------------TGGCCACCGCTGTGCGTTGTTGAAC
Contig_BR-1      -----------------------------------TGGCCACCGCTGTGCGTTGTTGAAC
Contig_BR-7      -----------------------------------TGGCCACCGCTGTGCGTTGTTGAAC
SH_BR            -----------------------------------TGGCCACCGCTGTGCGTTGTTGAAC
Contig_BR-4      -----------------------------------TGGCCACCGCTGTGCGTTGTTGAAC
right_jct_BR     -----------------------------------TGGCCACCGCTGTGCGTTGTTGAAC
Hd29_DRJ1R       ATTTCGTGGCAAGCGACTTTTCATCGTAGAACGGATGGCCACCGCTGTGCGTTGTTGAAC
                                                    * ** * *** ****** * ** * 

left_jct_BR      CAATGATGCAGCTTTGTGTTCTTCTCGAACGGTGAACATTCGTGCAACCAACCTGACACG
Hd29_DRJ1L       CAATGATGCAGCTTTGTGTCCTTCTCGAACGGTGAACATTCGTGCAACCAACCTGACACG
Contig_BR-10     CAATGATCCAACTTTGTGTTCTTCAGGAACGGTGAACATTCGTGCAATCAACTTGACACG
Contig_BR-1      CAATGATCCAACTTTGTGTTCTTCAGGAACGGTGAACATTCGTGCAATCAACTTGACACG
Contig_BR-7      CAATGATCCAACTTTGTGTTCTTCAGGAACGGTGAACATTCGTGCAATCAACTTGACACG
SH_BR            CAATGATCCAACTTTGTGTTCTTCAGGAACGGTGAACATTCGTGCAATCAACTTGACACG
Contig_BR-4      CAATGATCCAACTTTGTGTTCTTCAGGAACGGTGAACATTCGTGCAATCAACTTGACACG
right_jct_BR     CAATGATCCAACTTTGTGTTCTTCAGGAACGGTGAACATTCGTGCAATCAACTTGACACG
Hd29_DRJ1R       CAATGATCCAACTTTGTGTTCTTCAGGAACGGTGAACATTCGTGCAATCAACTTGACACG
                 ******* ** ******** ****  ********************* **** *******
```

```
left_jct_BR     TCGCCATTCGGAGAGAGGTTCGATAGACCATGAGGAATATCCAAACGTCATTCATTGTGC
Hd29_DRJ1L      TCGCCATTCGGAGAGAGGTTCGATAGACCATGAGGAATATCCAAACGTCATTCATTGTGC
Contig_BR-10    TCGTCATGCGGCAAGAAATTCGATAGACCATGAGGAATATCGTAACGTCATTCATCGTGC
Contig_BR-1     TCGTCATGCGGCAAGAAATTCGATAGACCATGAGGAATATCGTAACGTCATTCATCGTGC
Contig_BR-7     TCGTCATGCGGCAAGAAATTCGATAGACCATGAGGAATATCGTAACGTCATTCATCGTGC
SH_BR           TCGTCATGCGGCAAGAAATTCGATAGACCATGAGGAATATCGTAACGTCATTCATCGTGC
Contig_BR-4     TCGTCATGCGGCAAGAAATTCGATAGACCATGAGGAATATCGTAACGTCATTCATCGTGC
right_jct_BR    TCGTCATGCGGCAAGAAATTCGATAGACCATGAGGAATATCGTAACGTCATTCATCGTGC
Hd29_DRJ1R      TCGTCATGCGGCAAGAAACTCGATAGACCATGAGGAATATCGTAACGTCATTCATCGTGC
                *** *** ***  ***   *********************  ************ ****

left_jct_BR     GCACT--TCTAGGCTTTTGCAAT---CGAATCATTGCAGATACGCATTTTGAAGCAACGT
Hd29_DRJ1L      GCACT--TCTAGGCTATTGCAAT---CGAATCATTGCAGATACGCATTTTGAAGCAACGC
Contig_BR-10    ACGCATTCCTAGGACAAGTCTCTGCGCTCGAATCGTTGCGGATGCATTTGGAAGCAACGT
Contig_BR-1     ACGCATTCCTAGGACAAGTCTCTGCGCTCGAATCGTTGCGGATGCATTTGGAAGCAATGT
Contig_BR-7     ACGCATTCCTAGGACAAGTCTCTGCGCTCGAATCGTTGCGGATGCATTTGGAAGCAATGT
SH_BR           ACGCATTCCTAGGACAAGTCTCTGCGCTCGAATCGTTGCGGATGCATTTGGAAGCAATGT
Contig_BR-4     ACGCATTCCTAGGACAAGTCTCTGCGCTCGAATCGTTGCGGATGCATTTGGAAGCAATGT
right_jct_BR    ACGCATTCCTAGGACAAGTCTCTGCGCTCGAATCGTTGCGGATGCATTTGGAAGCAATGT
Hd29_DRJ1R      ACGCATTCCTAGGACAAGTCTCTGCGCTCGAATCGTTGCGGATGCATTTGGAAGCAATGT
                 * * *              *     *              * ****** ******* *

left_jct_BR     GGTTCAAATGATGTTGGGGAAAAGTGTGCAAACATTTATTAGATTGACCGAATGTTCATT
Hd29_DRJ1L      GGTTCAAATGATGTTGGGGAAAAGTGTGCAAACATTTATTAGATTGACCGAATGTTCATT
Contig_BR-10    GGTTCAAATGATGTTGGGGAAAAGTGTGCAAACATTTATTAGATTGACCGAATGTTCATT
Contig_BR-1     GTCTCCAATTATGTTGGGAAAAAGTGTCCAAAAATTTATTAGATTGACCGAATGTTAATT
Contig_BR-7     GTCTCCAATTATGTTGGGAAAAAGTGTCCAAAAATTTATTAGATTGACCGAATGTTAATT
SH_BR           GTCTCCAATTATGTTGGGAAAAAGTGTCCAAAAATTTATTAGATTGACCGAATGTTAATT
Contig_BR-4     GTCTCCAATTATGTTGGGAAAAAGTGTCCAAAAATTTATTAGATTGACCGAATGTTAATT
right_jct_BR    GTCTCCAATTATGTTGGGAAAAAGTGTCCAAAAATTTATTAGATTGACCGAATGTTAATT
Hd29_DRJ1R      GTCTCCAATTATGTTGGGAAAAAGTGTCCAAAAATTTATTAGATTGACCGAATGTTAATT
                *  ** *** ******** ******** **** *********************** ***

left_jct_BR     TGAACCTCTTGAAAAACTCACAGCTCGAGTTTAAGGAGAGTTTGACGGGATAGACGTGAC
Hd29_DRJ1L      TGAACCTCTTGAAAAACTCACAGCTCGAGGTTG---AGAGTTTGACGGGATAGACGTGAC
Contig_BR-10    TGAACCTCTTGAAAAACTCACAGCTCGAGTTTAAGGAGAGTTTGACGGGATAGACGTGAC
Contig_BR-1     TGGAGCTCTTGAGAAATTCACAGCTCGAGGTTAACGAGAGTTTGAAGGGATAAGCGTCAC
Contig_BR-7     TGGAGCTCTTGAGAAATTCACAGCTCGAGGTTAACGAGAGTTTGAAGGGATAAGCGTCAC
SH_BR           TGGAGCTCTTGAGAAATTCACAGCTCGAGGTTAACGAGAGTTTGAAGGGATAAGCGTCAC
Contig_BR-4     TGGAGCTCTTGAGAAATTCACAGCTCGAGGTTAACGAGAGTTTGAAGGGATAAGCGTCAC
right_jct_BR    TGGAGCTCTTGAGAAATTCACAGCTCGAGGTTAACGAGAGTTTGAAGGGATAAGCGTCAC
Hd29_DRJ1R      TGAAGCTCTTGAGAAATTCACAGCTCGAGGTTAACGAGAGTGTGAAGGGATAAGCGTCAC
                ** * ******* *** ************ **     ***** *** ******   *** **

left_jct_BR     GTTCTTCATGGAAGGTGTGGCTCGCAAACGTCGCATGCAGCTTGCGAGTTCCAAATGAAA
Hd29_DRJ1L      GTTCTTCATGGAAGGTGTGGCTCGCAAACGTCGCATGCAGCTTGCGAGTTCCAAATGAAA
Contig_BR-10    GTTCTTCATGGAAGGTGTGGCTCGCAAACGTCGCATGCAGCTTGCGAGTTCCAAATGAAA
Contig_BR-1     GTTCTTCATGGGCGGTGCGGCTCGCACACGTCGCATGGAGCTTGCGAGTTCCAAATGAAA
Contig_BR-7     GTTCTTCATGGACGGTGCGGCTCGCACACGTCGCATGCAGCTTGCGAGTTCCAAATGAAA
SH_BR           GTTCTTCATGGACGGTGCGGCTCGCAAACGTCGCATGCAGCTTGCGAGTTCCAAATGAAA
Contig_BR-4     GTTCTTCATGGACGGTGCGGCTCGCACACGTCGCATGGAGCTTGCGAGTTCCAAATAAAA
right_jct_BR    GTTCTTCATGGACGGTGCGGCTCGCACACGTCGCATGGAGCTTGCGAGTTCCAAATAAAA
Hd29_DRJ1R      GTTCTTCATGGACGGTGCGGCTCGCACACGTCGCATGGAGCTTGCGAGTTCCAAATAAAA
                ***********  **** ******** ********* ****************** ***

left_jct_BR     ACAACTGCGCGGGGTATTGTTTATTTCTGGAGAACACCG
Hd29_DRJ1L      ACAACTGCGCGGGGTATTGTTTATTTCTGGAGAACACCG
Contig_BR-10    ACAACTGCGCGGGGTATTGTTTATTTCTGGAGAACACCG
Contig_BR-1     ACAACTGCGCGGGGTATTGTTTATTTCTGGAGAACACCG
Contig_BR-7     ACAACTGCGCGGGGTATTGTTTATTTCTGGAGAACACCG
SH_BR           ACAACTGCGCGGGGTATTGTTTATTTCTGGAGAACACCG
Contig_BR-4     C-AACTGCGTGGGGTATTGTTTATCTCTTGAGAGCACCG no switch ????
right_jct_BR    C-AACTGCGTGGGGTATTGTTTATTTCTTGAGAGCACCG
Hd29_DRJ1R      C-AACTGCGTGGGGTATTGTTTATTTCTTGAGAGCACCG
                  ******* ************** *** **** *****
```

Segment Hd22

Sequences used for alignment:
(i) DRJs from genome: Hd22_DRJ1R, Hd22_DRJ1L
(ii) DRJs from BAC clone # CN-29M18: left_junction, right_junction
(iii) HdIV segments: Contig_CN-31, Contig_CN-33, Contig_CN-36, Contig_CN-39

Alignment

```
left_junction     CAATAGGGTGAATTCAAACGCTGGTCCAAATGGCAGGAGCTTGGCAGGACAGATGACATC
Hd22_DRJ1L        ----------AATTCAAACGCTGGTCCCAATGGCAGGAGCTTGGCAGGACAGATGACATC
Contig_CN-31      CAATAGGGTGAATTCAAACGCTGGTCCAAATGGCAGGAGCTTGGCAGGACAGATGACATC
Contig_CN-33      CAATAGGGTGAATTCAAACGCTGGTCCAAATGGCAGGAGCTTGGCAGGACAGATGACATC
Contig_CN-39      CAATAGGGTGAATTCAAACGCTGGTCCAAATGGCAGGAGCTTGGCAGGACAGATGACATC
Contig_CN-36      CAATAGGGTGAATTCAAACGCTGGTCCAAATGGCAGGAGCTTGACAAGGCAGATGATATC
right_junction    CATTGAGAAAAACTCAAGCGCCGATAGCAATGGCAGGAGCTTGACAAGGCAGATGATATC
Hd22_DRJ1R        ----------AACTCAAGCGCCGATAGCAATGGCAGGAGCTTGACAAGGCAGATGATATC
                            ** **** *** * *   *************** ** * ******* ***

left_junction     GAGTGAATGCGTAGGAGTCTGAAACAGCGCGTCGTGAACAATATCGTGACCATTGATCGC
Hd22_DRJ1L        GAGTGAATGCGTAGGAGTCTGAAACAGCGCGTCGTGAACAATATCGTGACCATTGATCGC
Contig_CN-31      GAGTGAATGCGTAGGAGTCTGAAACAGCGCGTCGTGAACAATATCGTGACCATTGATCGC
Contig_CN-33      GAGTGAATGCGTAGGAGTCTGAAACAGCGCGTCGTGAACAATATCGTGACCATTGATCGC
Contig_CN-39      GAGTGAATGCGTAGGAGTCTGAAACAGCGCGTCGTGAACAATATCGTGACCATTGATCGC
Contig_CN-36      GAGTGAGTGCGTAGGTCTCTGGAACCGCGGGTCGTGAACAATATCGTGACCATTGATCGC
right_junction    GAGTGAGTGCGTAGGTCTCTGGAACCGCGGGTCGTGAACAATATCGTGACCATTGATCGC
Hd22_DRJ1R        GAGTGAGTGCGTAGGTCTCTGGAACCGCGGGTCGTGAACAATATCGTGACCATTGATCGC
                  ****** ********  **** *** *** ******************************

left_junction     CATAGCGACTTGCCGCATCTCAGGATCGTGATCTAAAA--------CGTTTCACGTGA
Hd22_DRJ1L        CATAGCGACTTGCCGCATCTCAGGATCGTGATCTAAAA--------CGTTTCACGTGA
Contig_CN-31      CACAGCGACTTGGCGACTTGGCACACGTCAGCGTCAAGTTCCACAAAATCTGACGCGA
Contig_CN-33      CACAGCGACTTGGCGACTTGGCACACGTCAGCGTCAAGTTCCACAAAATCTGACGCGA
Contig_CN-39      CATAGCGACTTGGCGACTTGGCACACGTCAGCGTCAAGTTCCACAAAATCTGACGCGA
Contig_CN-36      CACAGCGACTTGGCGACTTGGCACACGTCAGCGTCAAGTTCCACAAAATCTGACGCGA
right_junction    CACAGCGACTTGGCGACTTGGCACACGTCAGCGTCAAGTTCCACAAAATCTGACGCGA
Hd22_DRJ1R        CACAGCGACTTGGCGACTTGGCACACGTCAGCGTCAAGTTCCACAAAATCTGACGCGA
                  ** ********* **  *      *        * **           * * *** **
```

Segment Hd19

Sequences used for alignment:
(i) DRJs from genome: Hd19_DRJ1R, Hd19_DRJ1L
(ii) DRJs from BAC clone # CP-38F08+CQ-40J21: CPCQ_right, CPCQ_left
(iii) HdIV segments: jct_CPCQ-28, jct_CPCQ-27, jct_CPCQ-23, jct_CPCQ-22

Alignment

```
CPCQ_left         TTTTTGCTGGTTCATGTTTCAGAGCAGCCGGTCTATGTGATACACGCTGCTTAGCCTGGT
Hd19_DRJ1L        TTTTTGCTGGTTCATGTTTCAGAGCAGCCGGTCTATGTGATACACGCTGCTTAGCCTGGT
jct_CPCQ-28       TTTTTGCTGCTTCATGTCTCAGAGCAGCTTGTCTACATGATACACGCTGATTTGTC-TGT
jct_CPCQ-27       TTTTTGCTGCTTCATGTCTCAGAGCAGCTTGTCTACATGATACACGCTGATTTGTC-TGT
jct_CPCQ-23       TTTTTGCTGCTTCATGTCTCAGAGCAGCTTGTCTACATGATACACGCTGATTTGTC-TGT
jct_CPCQ-22       TTTTTGCTGCTTCATGTCTCAGAGCAGCTTGTCTACATGATACACGCTGATTTGTC-TGT
CPCQ_right        TTTTTGCTGCTTCATGTCTCAGAGCAGCTTGTCTACATGATACACGCTGATTTGTC-TGT
Hd19_DRJ1R        TTTTTGCTGCTTCATGTCTCAGAGCAGCTTGTCTACATGATACACGCTGATTTGTC-TGT
                  ********* ******* **********  *****  ************ ** * *  **
```

```
CPCQ_left        GACGGCACGATGGTTCCCCTCATGCTGCACGAGCTCTGTGAGCTTT
Hd19_DRJ1L       GACGGCACGATGGTTCCCCTCATGCTGCACGAGCTCTGTGAGCTTT
jct_CPCQ-28      GACGGCACGATGGTTCCCCTCATGCTGCACGAGCTCTGTGAGCTTT
jct_CPCQ-27      GACGGCACGATGGTTCCCCTCATGCTGCACGAGCTCTGTGAGCTTT
jct_CPCQ-23      GACGGCACGATGGTTCCCCTCATGCTGCACGAGCTCTGTGAGCTTT
jct_CPCQ-22      GACGGCACGATAATTCGCCTCCTGCTGCACGAGCTCTGTGAGCTTT
CPCQ_right       GACGGCACGATAATTCGCCTCCTGCTGCACAAGCTTTGTGAGCTTT
Hd19_DRJ1R       GACGGCACGATAATTCGCCTCATGCTGCACAAGCTTTGTGAGCTTT
                 ***********  *** **** ******** **** **********
```

Segments Hd28

Sequences used for alignment:

(i) DRJs from genome: Hd28_DRJ1R, Hd28_DRJ1L, Hd24_DRJ1R (reverse), Hd24_DRJ1L (reverse)

(ii) DRJs from BAC clone # CR-47M17: CR_left, CR_right

(iii) HdIV segments: Contig_CR-05, Contig_CR-06, Contig_CR-09, Contig_CR-10

Alignment

```
CR_left          GCCTTTGCAGCTGAGCT-GCATGAGGTTTTCATGTCCTCGATAACTGGATTCCATTGTCA
Hd28_DRJ1L       GCCTTTGCAGCTGAGCT-GCATGAGGTTTTCATGTCCTCGATAACTGGATTCCATTGTCA
Contig_CR-05     GCCTTTGGAGCTAAACTTGCACGAGATTTCGGTGTCCGTGATAACTGAATGGCATTGTCA
Contig_CR-06     GCCTTTGGAGCTAAACTTGCACGAGATTTCGGTGTCCGTGATAACTGAATGGCATTGTCA
CR_right         GCCTTTGGAGCTAAACTTGCACGAGATTTCGGTGTCCGTGATAACTGAATGGCATTGTCA
Hd28_DRJ1R       GCCTTTGGAGCTAAACTTGCACGAGATTTCGGTGTCCGTGATAACTGAATGGCATTGTCA
                 ******* **** * ** *** *** ***   * ***  ******** **  ********

CR_left          GGCGTAAGCATTATGACGGCGGTGGTGTTAGGCGTTGCTTGGGAAGGTTGGTGAGCCACG
Hd28_DRJ1L       GGCGTAAGCATTATGACGGCGGTGGTGTTAGGCGTTGCTTGGGAAGGTTGGTGAGCCACG
Contig_CR-05     GGCGTAATATTTATGACGGC---------------------------------GGTGATG
Contig_CR-06     GGCGTAATATTTATGACGGC---------------------------------GGTGATG
CR_right         GGCGTAATATTTATGACGGC---------------------------------GGTGATG
Hd28_DRJ1R       GGCGTAATATTTATGACGGC---------------------------------GGTGATG
                 *******   **********                                  *  * *

CR_left          TCGACGTAGATTCAGGCGTGAAACGGAGAGCTGAAAGGTATAGTAGTCGTTCGACCAACT
Hd28_DRJ1L       TCGACGTAGATTCAGGCGTGAAACGGAGAGCTGAAAGGTATAGTAGTCGTTCGACCAACT
Contig_CR-05     TTAACGTAGGTTCAGGCGTTAGACTGAGAGCCGGAAGGTATGGTAGTCGTTCGACCGTCT
Contig_CR-06     TTAACGTAGGTTCAGGCGTTAGACTGAGAGCCGGAAGGTATGGTAGTCGTTCGACCGTCT
CR_right         TTAACGTAGGTTCAGGCGTTAGACTGAGAGCCGGAAGGTATGGTAGTCGTTCGACCGTCT
Hd28_DRJ1R       TTAACGTAGGTTCAGGCGTTAGACTGAGAGCCGGAAGGTATGGTAGTCGTTCGACCGTCT
                 *  ****** ********  * ** ****** * ******  **************  **

CR_left          CGGAATCACACCGACGAAAACTATTTGTCCATTGCTCAGGCTATGAGGACTTGAGGCAAT
Hd28_DRJ1L       CGGAATCACACCGACGAAAACTATTTGTCCATTGCTCAGGCTATGAGGACTTGAGGCAAT
Contig_CR-05     CCGAATCACGCTGACGAGAACTCTTCGTTCATCGCTTAAGCTCTGAGGACTTGACGCAAT
Contig_CR-06     CCGAATCACGCTGACGAGAACTCTTCGTTCATCGCTTAAGCTCTGAGGACTTGACGCAAT
CR_right         CCGAATCACGCTGACGAGAACTCTTCGTTCATCGCTTAAGCTCTGAGGACTTGACGCAAT
Hd28_DRJ1R       CCGAATCACGCTGACGAGAACTCTTCGTTCATCGCTTAAGCTCTGAGGACTTGACGCAAT
                 * ******* * ***** **** ** ** *** *** * ***  ********** *****

CR_left          CAAGTTCCGGACGCAAAGT----------TTTGTTTCCGGCCATAGATCACAAGTAAGAA
Hd28_DRJ1L       CAAGTTCCGGACGCAAAGT----------TTTGTTTCCGGCCATAGATCACAAGTAAGAA
Contig_CR-05     CAAGTTCTGGATGATAAACGCCAGACGCAAAGTTTTGTTTCCATAGATCACAAGTAAGAA
Contig_CR-06     CAAGTTCTGGATGATAAACGCCAGACGCAAAGTTTTGTTTCCATAGATCACAAGTAAGAA
CR_right         CAAGTTCTGGATGATAAACGCCAGACGCAAAGTTTTGTTTCCATAGATCACAAGTAAGAA
Hd28_DRJ1R       CAAGTTCTGGATGATAAACGCCAGACGCAAAGTTTTGTTTCCATAGATCACAAGTAAGAA
                 ******* *** *  **                 **    ********************

CR_left          CAGTTCTGGCAGACTGGACAGTCCACCGATACCCTCACAAGACTGTAAA-ACCTCTGGAC
Hd28_DRJ1L       CAGTTCTGGCAGACTGGACAGTCCACCGATACCCTCACAAGACTGTAAA-ACCTCTGGAC
Contig_CR-05     CAGTTCTTACGGACCTGACAGTCCAACATTACTCTCACGACACTGTAAA-ACCTATGGAC
Contig_CR-06     CAGTTCTTACGGACCTGACAGTTCAACATTACTCTCACGACACTGTAAA-ACCTATGGAC
CR_right         CAGTTCTTACGGACCTGACAGTCCAACATTACTCTCACGACACTGTAAA-ACCTATGGAC
Hd28_DRJ1R       CAGTTCTTACGGACCTGACAGTCCAACATTACTCTCACGACACTGTAAA-ACCTATGGAC
                 *******  * ***  ****** ** *  *** ***** ********** **** *****
```

```
CR_left         GTACTGCGCACGTACCCAAGTCCACGAAACACTCGTGCCACATCCATTTGATGTTGGCTT
Hd28_DRJ1L      GTACTGCGCACGTACCCAAGTCCACGAAACACTCGTGCCACATCCATTTGATGTTGGCTT
Contig_CR-05    GTACTTCGCACGTACCCGAGTCCACGAAACACTCGTGCTACATCCATTTGACGTTGGCCT
Contig_CR-06    GTACTTCGCACGTACCCGAGTCCACGAAACACTCGTGCTACATCCATTTGACGTTGGCCT
CR_right        GTACTTCGCACGTACCCGAGTCCACGAAACACTCGTGCTACATCCATTTGACGTTGGCCT
Hd28_DRJ1R      GTACTTCGCACGTACCCGAGTCCACGAAACACTCGTGCTACATCCATTTGACGTTGGCCT
                ***** *********** ******************** ************ ****** *

CR_left         CTGCCAGGGCATGTCGCTAT--AGATTCCCAGCGTGTGCCCGTGTCCCAGAAAAAGCTTT
Hd28_DRJ1L      CTGCCAGGGCATGTCGCTAT--AGATTCCCAGCGTGTGCCCGTGTCCCAGAAAAAGCTTT
Contig_CR-05    TCT----GACAAGGCCCACTATAGAGTCCCAGCGTGTGCCCGTGTCCCGGAAGGAGCTTT
Contig_CR-06    TCT----GACAAGGCCCACTATAGAGTCCCAGCGTGTGCCCGTGTCCCGGAAGGAGCTTT
CR_right        TCT----GACAAGGCCCACTATAGAGTCCCAGCGTGTGCCCGTGTCCCGGAAGGAGCTTT
Hd28_DRJ1R      TCT----GACAAGGCCCACTATAGAGTCCCAGCGTGTGCCCGTGTCCCGGAAGGAGCTTT
                       * ** *****  *  *** ********************** ***  ******

CR_left         GTTCACAACCGCGACTTTTGACTGTCACCTTTGTTGTTACAGTTGCGTTACGTAACTACT
Hd28_DRJ1L      GTTCACAACCGCGACTTTTGACTGTCACCTTTGTTGTTACAGTTGCGTTACGTAACTACT
Contig_CR-05    T-ACTCCCAACCGACTTTTTAGTGTGACCTTTGTCGTTACAGTTGCGTTACGTAACTACT
Contig_CR-06    T-ACTCCCAACCGACTTTTTAGTGTGACCTTTGTCGTTACAGTTGCGTTACGTAACTACT
CR_right        T-ACTCCCAACCGACTTTTTAGTGTGACCTTTGTCGTTACAGTTGCGTTACGTAACTACT
Hd28_DRJ1R      T-ACTCCCAACCGACTTTTTAGTGTGACCTTTGTCGTTACAGTTGCGTTACGTAACTACT
                   * *     ******** * *** ******** *************************

CR_left         ACTGTGGAACTTCTCACTGCCACAGTCACAACAGCCTATCGACAACTGTTCTTTCAGTAT
Hd28_DRJ1L      ACTGTGGAACTTCTCACTGCCACAGTCACAACAGCCTATCGACAACTGTTCTTTCAGTAT
Contig_CR-05    ACTGTGGAACTTTTCACTACCACAGTTACAACAGCTTATCGGCAACAGTCCCTTCAGTAT
Contig_CR-06    ACTGTGGAACTTTTCACTACCACAGTTACAACAGCTTATCGGCAACAGTTCCTTCAGTAT
CR_right        ACTGTGGAACTTTTCACTACCACAGTTACAACAGCTTATCGGCAACAGTTCCTTCAGTAT
Hd28_DRJ1R      ACTGTGGAACTTTTCACTACCACAGTTACAACAGCTTATCGGCAACAGTTCCTTCAGTAT
                ************ ***** ******* *******  ***** **** **** ********

CR_left         CAAGAAGTCATTATGCAAGTAGAGAATGGCCGGGGTTTTAAGTTGCATGAAA-GCACTCAA
Hd28_DRJ1L      CAAGAAGTCATTATGCAAGTAGAGAATGGCCGGGGTTTTAAGTTGCATGAAA-GCACTCAA
Contig_CR-05    CAAGAAGTAATTATTCAAGTGGAGAATGGCCGGGGTTTTAAGTTGCATGAAA-GCACTCAA
Contig_CR-06    CAAGAAGTAATTATTCAAGTGGAGAATGGCCGGGGTCGG-AGTTGCATGAAA-GCACTCAA
CR_right        CAAGAAGTAATTATTCAAGTGGAGAATGGCCGGGGTCGG-AGTTGCATGAAAAGCACGGAA
Hd28_DRJ1R      CAAGAAGTAATTATTCAAGTGGAGAATGGCCGGGGTCGG-AGTTGCATGAAAAGCACGGAA
                ******** ***** ***** ***************    ************ ****  **

CR_left         TTGTCTACAATGCAGACGAG
Hd28_DRJ1L      TTGTCTACAATGCAGACGAG
Contig_CR-05    TTGTCTACAATGCAGACGAG
Contig_CR-06    TTGTCTACAATGCAGACGAG
CR_right        GTGTGTACAATACAGACGGG
Hd28_DRJ1R      GTGTGTACAATACAGACGGG
                 *** ****** ****** *
```
