# Supplementary material for: Genomic architecture of endogenous ichnoviruses reveals distinct evolutionary pathways leading to virus domestication in parasitic wasps
Source: BMC Biol. 2020 Jul 24;18:89. doi: 10.1186/s12915-020-00822-3 (PMC7379367; doi:10.1186/s12915-020-00822-3)

Additional file 12. Comparative analysis of *Campoletis sonorensis* and *Hyposoter didymator* viral segments.

Figure S4. Proviral segment size and gene content, i.e. the number of genes of each multigenic family found per segment. **a**. *C. sonorensis* ichnovirus (CsIV). **b.** *H. didymator* ichnovirus (HdIV). Ichnovirus genes: rep, repeat element genes; vinx, viral innexin; vank, viral ankyrin; cys, cys-motif rich protein; PRRP, polar residue rich protein; N, N gene; others: hypothetical proteins.

a

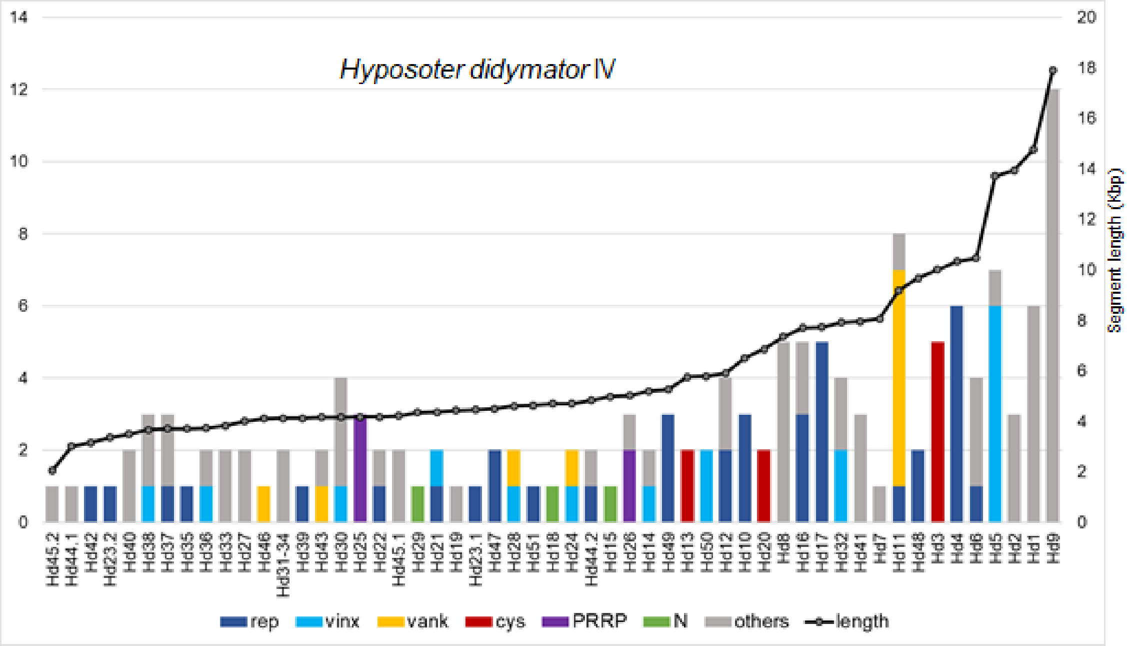

b

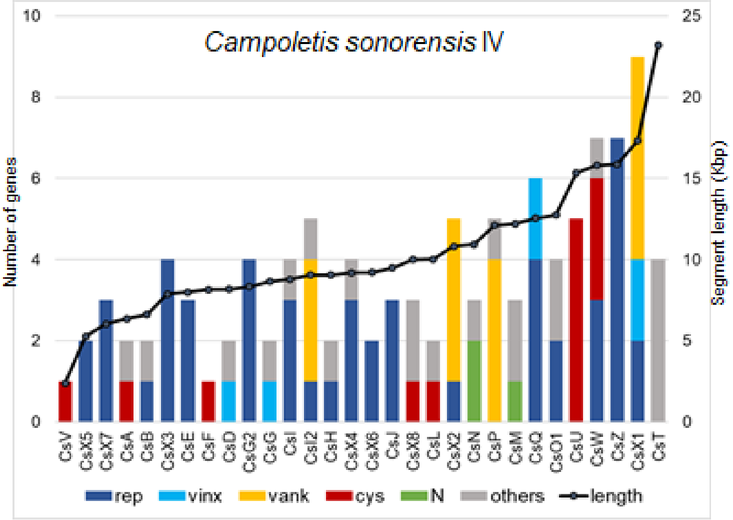

Figure S5. Synteny of *H. didymator* genomic regions where viral segments are inserted compared with *C. sonorensis* and other parasitoid genomes. **a.** Example of a syntenic region where only the *H. didymator* genome presents a viral segment insertion. *H. didymator* genes

from HD005010 to HD005030. **b.** The unique case found of a syntenic region where a viral segment in *H. didymator* and an IVSPER in *C. sonorensis* are inserted in the same position. *H. didymator* genes from HD010552 to HD010574. **c.** The unique case found of a syntenic region where a viral segment is inserted in both *H. didymator* and *C. sonorensis* genomes, but in two different positions. *H. didymator* genes from HD010503 to HD010526. Hd: *Hyposoter didymator*; Cs: *Campoletis sonorensis*; Vc: *Venturia canescens* (ichneumonid that has lost the ichnovirus ([69]); Md: *Microplitis demolitor* (braconid with a bracovirus); Fa: *Fopius arisanus* (braconid with virus-like particles). Numbers following the species name correspond to scaffold number for Hd, Cs and Vc, NCBI project codes for Md and Fa). Triangles within genomic regions correspond to predicted genes; triangles of the same color correspond to ortholog genes; white triangles are singletons or orphan genes. For better visualization, the name of the gene is indicated only for some viral (in red for segments, in blue for IVSPERs) genes. See Additional file 13, Table S13, for *H. didymator* genes list.

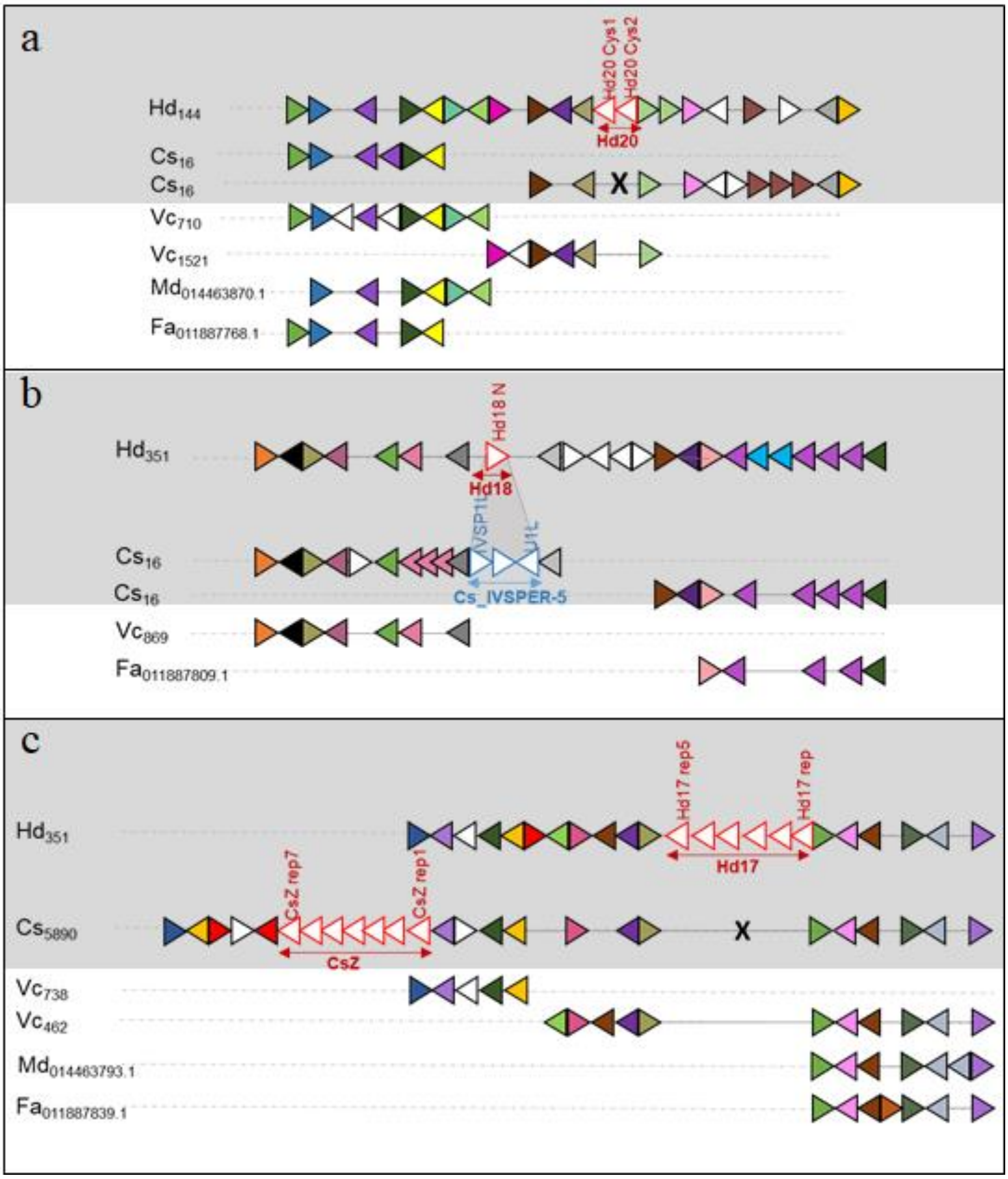

Supplement: Supplementary file 12 — Additional file 12: Comparative analysis of Campoletis sonorensis and Hyposoter didymator viral segments. Figure S4. Proviral segment size and gene content, i.e. the number of genes of each multigenic family found per segment. a. C. sonorensis ichnovirus (CsIV). b. H. didymator ichnovirus (HdIV). Ichnovirus genes: rep, repeat element genes; repM, repeat element genes with multiple repeated elements; vinx, viral innexin; vank, viral ankyrin; cys, cys-motif rich protein; PRRP, polar residue rich protein; N, N gene; Gly-Pro, glycine-proline rich protein. Figure S5. Synteny of H. didymator genomic regions where viral segments are inserted compared with C. sonorensis and other parasitoid genomes. a. Example of a syntenic region where only the H. didymator genome presents a viral segment insertion. H. didymator genes from HD005010 to HD005030. b. The unique case found of a syntenic region where a viral segment in H. didymator and an IVSPER in C. sonorensis are inserted in the same position. H. didymator genes from HD010552 to HD010574. c. The unique case found of a syntenic region where a viral segment is inserted in both H. didymator and C. sonorensis genomes, but in two different positions. H. didymator genes from HD010503 to HD010526. Hd: Hyposoter didymator; Cs: Campoletis sonorensis; Vc: Venturia canescens (ichneumonid that has lost the ichnovirus ([22]); Md: Microplitis demolitor (braconid with a bracovirus); Fa: Fopius arisanus (braconid with virus-like particles). Numbers following the species name correspond to scaffold number for Hd, Cs and Vc, NCBI project codes for Md and Fa). Triangles within genomic regions correspond to predicted genes; triangles of the same color correspond to orthologs; white triangles are singletons or orphan genes. For better visualization, the name of the gene is indicated only for some viral (in red for segments, in blue for IVSPERs) genes. See Additional file 13: Table S13, for H. didymator genes list. [file 12915_2020_822_MOESM12_ESM.pdf]
