# Supplementary material for: Genomic architecture of endogenous ichnoviruses reveals distinct evolutionary pathways leading to virus domestication in parasitic wasps
Source: BMC Biol. 2020 Jul 24;18:89. doi: 10.1186/s12915-020-00822-3 (PMC7379367; doi:10.1186/s12915-020-00822-3)
Supplement: Supplementary file 13 — Additional file 13: Table S13. List of the Hyposoter didymator genes present in the syntenic blocks represented in Additional file 12: Fig. S5 and in Fig. 4. Are indicated the H. didymator scaffold name, gene ID, its position in the scaffold, the number of the orthogroup to which it belongs and the result of the best match obtained following Blast similarity search. For each H. didymator gene, the corresponding Campoletis sonorensis gene ID, orthogroup number and position in C. sonorensis scaffold are indicated. [file 12915_2020_822_MOESM13_ESM.pdf]

Genes in synteny blocks Figure 5C, a

Hd scaffold	Position in scaffold	Hd gene ID	Orthogroup #	Best Blast hit			Cs gene	Orthogroup #	Position in scaffold
scaffold144	1380719..1397417	HD005010	OG0000722	gi|817190146|ref|XP_012270430.1|	uncharacterized protein LOC105694386 isoform X2 [Orussus abietir	Expect = 0.000e+0	gene.scaffold_16.g181.t1	OG0000722	2082674..2097848
scaffold144	1402033..1404758	HD005011	OG0008876	gi|1000730346|ref|XP_015587582.1	dynein assembly factor 4, axonemal-like [Cephus cinctus]	Expect = 8.300e-150	gene.scaffold_16.g180.t1	OG0008876	2075777..2079078
scaffold144	1405145..1406140	HD005012	OG0008079	gi|340719499|ref|XP_003398190.1|	vascular endothelial growth factor A-A [Bombus terrestris]	Expect = 7.800e-47	gene.scaffold_16.g179.t1	OG0008079	2074775..2075425
scaffold144	1410042..1410578	HD005013	OG0009360	gi|987906890|ref|XP_015428945.1|	PREDICTED: uncharacterized protein LOC107185710 [Dufourea nov	Expect = 2.700e-41	gene.scaffold_16.g178.t1	OG0009360	2064292..2070214
scaffold144	1412742..1423051	HD005014	OG0007034	gi|755949684|ref|XP_011301181.1|	PREDICTED: nucleoprotein TPR [Fopius arisanus]	Expect = 0.000e+0	gene.scaffold_16.g177.t1	OG0007034	2058216..2067490
scaffold144	1423611..1429572	HD005015	OG0004177	gi|1000729872|ref|XP_015587356.1	protein virilizer isoform X1 [Cephus cinctus]	Expect = 0.000e+0	NA		
scaffold144	1429964..1432653	HD005016	OG0007546	gi|1070214570|ref|XP_018376608.1	PREDICTED: pentatricopeptide repeat-containing protein 1, mitoch	Expect = 7.200e-127	NA		
scaffold144	1434554..1435954	HD005017	OG0009748	gi|1070154465|ref|XP_018340726.1	PREDICTED: RISC-loading complex subunit TARBP2-like [Trachymyr	Expect = 2.500e-30	NA		
scaffold144	1438643..1440697	HD005018	OG0003141	gi|645023101|ref|XP_008202437.1|	PREDICTED: neuralized-like protein 2 [Nasonia vitripennis]	Expect = 8.100e-114	gene.scaffold_16.g36.t1	OG0003141	254028..255633
scaffold144	1442194..1445201	HD005019	OG0003140	gi|1317987018|ref|XP_023287837.1	neuronal acetylcholine receptor subunit alpha-5 isoform X1 [Orussu	Expect = 2.500e-59			
scaffold144	1446370..1448826	HD005020	OG0009176	gi|939631532|ref|XP_008554100.2|	PREDICTED: neuronal acetylcholine receptor subunit alpha-5-like [N	Expect = 7.200e-80	gene.scaffold_16.g37.t1	OG0009176	257530..261137
scaffold144	1450485..1451362	HD005021	OG0007822	Cys2_Hd20					
scaffold144	1454299..1455511	HD005022	OG0007822	Cys1_Hd20					
scaffold144	1457007..1457513	HD005023	OG0001009	gi|805787897|ref|XP_012141035.1|	PREDICTED: uncharacterized protein LOC100876911 isoform X2 [M	Expect = 2.000e-20	gene.scaffold_16.g38.t1	OG0001009	270567..274688
scaffold144	1464373..1467934	HD005024	OG0001009	gi|795056915|ref|XP_011872016.1|	PREDICTED: uncharacterized protein LOC105564328 isoform X1 [Vc	Expect = 2.100e-124			
scaffold144	1472127..1477975	HD005025	OG0006050	gi|1000745701|ref|XP_015595595.1	cysteine and histidine-rich protein 1 homolog isoform X5 [Cephus c	Expect = 2.400e-190	gene.scaffold_16.g39.t1	OG0006050	278822..284602
scaffold144	1479354..1480103	HD005026	orphan	gi|759078894|ref|XP_011349245.1|	polyadenylate-binding protein 1 [Ooceraea biroi]	Expect = 2.100e-23	gene.scaffold_16.g40.t1	orphan	287471..288253
							gene.scaffold_16.g42.t1	orphan	331489..359465
scaffold144	1508038..1591917	HD005027	OG0001516	gi|755949182|ref|XP_011301029.1|	PREDICTED: regulating synaptic membrane exocytosis protein 1 iso	Expect = 0.000e+0	gene.scaffold_16.g43.t1	OG0001516	385633..394202
scaffold144	1601947..1606850	HD005028	orphan	gi|998501744|ref|XP_015510887.1|	PREDICTED: uncharacterized protein LOC107217756 [Neodiprion le	Expect = 2.200e-36	gene.scaffold_16.g44.t1	OG0001516	402325..405981
scaffold144							gene.scaffold_16.g45.t1	OG0001516	413940..415699
scaffold144	1607444..1607878	HD005029	OG0007431	gi|815909475|ref|XP_003489119.2|	chromosome transmission fidelity protein 8 homolog [Bombus imp	Expect = 9.300e-30	gene.scaffold_16.g46.t1	OG0007431	427568..428032
scaffold144	1609469..1618501	HD005030	OG0001137	gi|970888608|ref|XP_015109471.1|	PREDICTED: uncharacterized protein LOC107036203 [Diachasma all	Expect = 2.600e-122	gene.scaffold_16.g47.t1	OG0001137	431251..442478

Genes in synteny blocks Figure 5C, b

Hd scaffold	Position in scaffold	Hd gene ID	Orthogroup #	Best Blast hit			Cs gene	Orthogroup #	Position in scaffold
scaffold351	2570811..2574280	HD010552	OG0006536	gi|970890219|ref|XP_015110360.1|	PREDICTED: GPI mannosyltransferase 4 [Diachasma alloeum]	Expect = 6.400e-248	gene.scaffold_16.g210.t1	OG0006536	2319748..2322545
scaffold351	2575688..2584750	HD010553	OG0000820	gi|1000729971|ref|XP_015587409.1	histone deacetylase 6 isoform X3 [Cephus cinctus]	Expect = 0.000e+0	gene.scaffold_16.g211.t1	OG0000820	2323661..2331643
scaffold351	2585486..2590674	HD010554	OG0008529	gi|1000729989|ref|XP_015587415.1	mitochondrial intermediate peptidase isoform X1 [Cephus cinctus]	Expect = 1.300e-309	gene.scaffold_16.g212.t1	OG0008529	2333001..2336567
scaffold351	2592030..2601204	HD010555	OG0005613	gi|755986949|ref|XP_011311356.1|	PREDICTED: cullin-4A [Fopius arisanus]	Expect = 0.000e+0	gene.scaffold_16.g213.t1	OG0005613	2338026..2342787
scaffold351	2603266..2606649	HD010556	OG0007486	gi|1000718247|ref|XP_015598324.1	protein crooked neck [Cephus cinctus]	Expect = 1.700e-240	gene.scaffold_16.g214.t1	OG0007486	2344137..2346984
							gene.scaffold_16.g216.t1	orphan	2352827..2353396
scaffold351	2614497..2669283	HD010557	OG0000315	gi|1059231176|ref|XP_017760895.1	PREDICTED: nuclear factor 1 X-type-like isoform X7 [Eufriesea mexi	Expect = 0.000e+0	gene.scaffold_16.g218.t1	OG0000315	2360992..2364340
							gene.scaffold_16.g219.t1	OG0000315	2373432..2374064
							gene.scaffold_16.g220.t1	OG0000315	2387249..2388404
scaffold351	2678768..2680678	HD010558	OG0007969	gi|755943768|ref|XP_011299232.1|	PREDICTED: actin-related protein 2/3 complex subunit 4 [Fopius ari	Expect = 5.700e-81	gene.scaffold_16.g221.t1	OG0007969	2416191..2419469
scaffold351	2683737..2685050	HD010559	orphan	N1_Hd18			gene.scaffold_16.g223.t1	OG0010684	2424942..2425688
							gene.scaffold_16.g564.t1	orphan	2426081..2426764
							gene.scaffold_16.g224.t1	orphan	2427951..2428691
scaffold351	2688803..2689573	HD010560	OG0005107	gi|972178531|ref|XP_015191350.1|	PREDICTED: transmembrane protein 45B-like [Polistes dominula]	Expect = 3.600e-95	gene.scaffold_16.g225.t1	OG0005107	2430738..2431508
scaffold351	2690793..2699707	HD010561	OG0003436	gi|820860113|ref|XP_003697297.2|	PREDICTED: LOW QUALITY PROTEIN: enhancer of mRNA-decapping	Expect = 0.000e+0	NA		
scaffold351	2700580..2701786	HD010562	OG0007575	gi|1227092722|gb|OXU18804.1|	hypothetical protein TSAR_005200 [Trichomalopsis sarcophagae]	Expect = 3.400e-82	NA		
scaffold351	2706953..2708212	HD010563	OG0009633	gi|1580172942|ref|XP_028048451.	uncharacterized protein LOC114255006 [Monomorium pharaonis]	Expect = 1.100e-29	NA		
scaffold351	2712014..2713607	HD010564	OG0000118	gi|795032310|ref|XP_011863894.1|	PREDICTED: speckle-type POZ protein A-like [Vollenhovia emeryi]	Expect = 6.600e-20	NA		
scaffold351	2714791..2716848	HD010565	OG0005178	gi|1000730046|ref|XP_015587445.1	actin-related protein 10 [Cephus cinctus]	Expect = 2.800e-165	gene.scaffold_16.g124.t1	OG0005178	1439439..1441701
scaffold351	2717065..2719279	HD010566	OG0008078	gi|817195095|ref|XP_012273078.1|	eukaryotic translation initiation factor 3 subunit F [Orussus abietinu	Expect = 4.500e-139	gene.scaffold_16.g125.t1	OG0008078	1441946..1443651
scaffold351	2720235..2740116	HD010567	OG0000165	gi|915666462|gb|KOC68367.1|	Phosphatidylinositol 4-phosphate 5-kinase type-1 alpha [Habropod	Expect = 0.000e+0	gene.scaffold_16.g126.t1	OG0000165	1444610..1449264
scaffold351	2744556..2745842	HD010568	OG0000280	gi|998499361|ref|XP_015509568.1|	PREDICTED: venom peptide isomerase heavy chain-like [Neodiprior	Expect = 5.200e-119	gene.scaffold_16.g129.t1	OG0000280	1468965..1476057
scaffold351	2746325..2748699	HD010569	OG0010371	no hit					
scaffold351	2749640..2750960	HD010570	OG0010371	no hit					
scaffold351	2754546..2756977	HD010571	OG0000280	gi|755977086|ref|XP_011308165.1|	PREDICTED: trypsin-like [Fopius arisanus]	Expect = 1.600e-80	gene.scaffold_16.g130.t1	OG0000280	1479694..1481353

scaffold351	2757727..2759955	HD010572	OG0000280	gi|755977086|ref|XP_011308165.1|	PREDICTED: trypsin-like [Fopius arisanus]	Expect = 2.500e-81	gene.scaffold_16.g131.t:	OG0000280	1482054..1483447
scaffold351	2760364..2762511	HD010573	OG0000280	gi|755977083|ref|XP_011308164.1|	PREDICTED: trypsin II-P29-like [Fopius arisanus]	Expect = 4.300e-75	gene.scaffold_16.g132.t:	OG0000280	1484135..1488423
scaffold351	2765019..2770447	HD010574	OG0008378	gi|755977080|ref|XP_011308163.1|	PREDICTED: carboxypeptidase B-like [Fopius arisanus]	Expect = 8.000e-215	gene.scaffold_16.g133.t:	OG0008378	1489809..1493726

Genes in synteny blocks Figure 5C, c

Hd scaffold	Position in scaffold	Hd gene ID	Orthogroup #	Best Blast hit			Cs gene	Orthogroup	Position in scaffold
		NA					*gene.scaffold_5890.g33.*	*OG0000028*	*162272..162676*
		NA					*gene.scaffold_5890.g32.*	*OG0000021*	*161726..162136*
		NA					*gene.scaffold_5890.g31.*	*OG0000024*	*156325..156699*
		NA					*gene.scaffold_5890.g30.*	*orphan*	*151548..155209*
		NA					*gene.scaffold_5890.g29.*	*OG0000024*	*151509..151883*
		NA					*gene.scaffold_5890.g28.*	*OG0002056*	*146321..147118*
		NA					*/// *		
		NA					*gene.scaffold_5890.g59t*	*OG0002056*	*134843..135562*
scaffold351	2270991..2271302	HD010503	OG0000028	gi|170058994|ref|XP_001865168.1|	histone 1 [Culex quinquefasciatus]	Expect = 3.900e-48			
scaffold351	2273424..2278678	HD010504	OG0003636	gi|1000742598|ref|XP_015593950.1	equilibrative nucleoside transporter 4 [Cephus cinctus]	Expect = 1.500e-267	gene.scaffold_5890.g22.	OG0003636	128010..132737
scaffold351	2280628..2284708	HD010505	OG0001788	gi|954555894|ref|XP_014601533.1|	PREDICTED: serine/threonine-protein kinase ICK [Polistes canadens	Expect = 3.200e-256	gene.scaffold_5890.g20.	OG0001788	120284..125583
scaffold351	2288342..2289300	HD010506	orphan	no hit			gene.scaffold_5890.g18.	OG0000067	111355..111939
scaffold351	2292636..2299704	HD010507	OG0001161	gi|970889125|ref|XP_015109758.1|	PREDICTED: DENN domain-containing protein 1A-like isoform X1 [D	Expect = 0.000e+0	gene.scaffold_5890.g17.	OG0001161	101367..109432
scaffold351	2300822..2301232	HD010508	OG0000021	gi|170053440|ref|XP_001862674.1|	histone H3 type 2 [Culex quinquefasciatus]	Expect = 3.000e-64	gene.scaffold_5890.g16.	OG0000021	97954..100617
scaffold351	2302839..2303213	HD010509	OG0000024	gi|815814502|ref|XP_012228276.1|	PREDICTED: histone H2A-like [Linepithema humile]	Expect = 1.700e-58			
scaffold351	2304346..2305863	HD010510	OG0008873	gi|1000757495|ref|XP_015601701.1	activator of basal transcription 1 isoform X2 [Cephus cinctus]	Expect = 1.900e-83			
scaffold351	2306413..2308908	HD010511	OG0004415	gi|307203421|gb|EFN82496.1|	mRNA-decapping enzyme 2 [Harpegnathos saltator]	Expect = 1.000e-159	gene.scaffold_5890.g15.	OG0004415	95411..97479
scaffold351	2310086..2311637	HD010512	OG0007967	gi|954580551|ref|XP_014614868.1|	PREDICTED: heme oxygenase 1 [Polistes canadensis]	Expect = 3.300e-93			
scaffold351	2312957..2318568	HD010513	OG0006585	gi|1000715934|ref|XP_015586052.1	peroxisomal multifunctional enzyme type 2 isoform X1 [Cephus cin	Expect = 2.200e-259	gene.scaffold_5890.g14.	OG0006585	86991..95435
scaffold351	2320285..2328116	HD010514	OG0002518	gi|1000757483|ref|XP_015601693.1	hypoxia up-regulated protein 1 isoform X1 [Cephus cinctus]	Expect = 1.000e-304	gene.scaffold_5890.g13.	OG0002518	79568..85710
scaffold351	2329743..2330387	HD010515	orphan	Rep5_Hd17					
scaffold351	///								
scaffold351	2335459..2336166	HD010519	orphan	Rep1_Hd17					
scaffold351	2336786..2337193	HD010520	orphan	rep partial_Hd17					
scaffold351	2337418..2340007	HD010521	OG0002835	gi|808129402|ref|XP_012167532.1|	WW domain-binding protein 2 isoform X1 [Bombus terrestris]	Expect = 2.300e-101	gene.scaffold_5890.g12.	OG0002835	76069..78711
scaffold351	2345118..2346993	HD010522	OG0006513	gi|1227095170|gb|OXU20342.1|	hypothetical protein TSAR_005800 [Trichomalopsis sarcophagae]	Expect = 1.500e-104	gene.scaffold_5890.g11.	OG0006513	69897..70791
scaffold351	2349858..2352634	HD010523	OG0007554	gi|1059880711|ref|XP_017795737.1	PREDICTED: ubiquitin domain-containing protein UBFD1-like [Habrc	Expect = 2.500e-105	gene.scaffold_5890.g10.	OG0007554	63578..65852
scaffold351	2353611..2362101	HD010524	OG0003626	gi|970912543|ref|XP_015122543.1|	PREDICTED: polycomb protein suz12-B [Diachasma alloeum]	Expect = 0.000e+0	gene.scaffold_5890.g9.t:	OG0003626	55732..62546
scaffold351	2363619..2368282	HD010525	OG0004797	gi|1000730147|ref|XP_015587495.1	methyltransferase-like protein 22 [Cephus cinctus]	Expect = 5.700e-92	gene.scaffold_5890.g8.t:	OG0004797	51570..54218
scaffold351	2368680..2372470	HD010526	OG0006517	gi|1476364581|gb|AXY94695.1|	disulfide-isomerase [Habrobracon hebetor]	Expect = 1.100e-196	gene.scaffold_5890.g7.t:	OG0006517	44960..49478

Genes in synteny blocks Figure 6C, a

Hd scaffold	Position in scaffold	Hd gene ID	Orthogroup #	Best Blast hit			Cs gene	Orthogroup	Position in scaffold
Scaffold 91	339180-342661	HD016092	OG0004987	gi|780657091|ref|XP_011691874.1|	PREDICTED: solute carrier organic anion transporter family membei	Expect = 1.400e-275	gene.scaffold_14.g96.t1	OG0004987	672138..680445
Scaffold 91	370839-379409	HD016093	OG0004824	gi|769843355|ref|XP_011632893.1|	solute carrier organic anion transporter family member 5A1 isoforn	Expect = 0.000e+0	gene.scaffold_14.g97.t1	OG0004824	687109..693641
Scaffold 91	386420-391295	HD016094	OG0004411	gi|755943227|ref|XP_011299047.1|	PREDICTED: G-protein coupled receptor Mth isoform X2 [Fopius ari:	Expect = 9.200e-139	gene.scaffold_14.g98.t1	orphan	691335..692107
Scaffold 91	392450-394130	HD016095	OG0001577	gi|1492480063|ref|XP_026674315.1	probable RNA methyltransferase CG11342 isoform X1 [Ceratina cal	Expect = 5.800e-72	gene.scaffold_14.g99.t1	OG0001577	701142..708415
Scaffold 91	396109-397563	HD016096	OG0001965	gi|749787677|ref|XP_011147350.1|	myosin-2 essential light chain isoform X2 [Harpegnathos saltator]	Expect = 3.300e-77	gene.scaffold_14.g100.t:	OG0001965	709518..710732
Scaffold 91	398929-404614	HD016097	OG0004676	gi|972210109|ref|XP_015187432.1|	PREDICTED: WD repeat, SAM and U-box domain-containing protein	Expect = 2.500e-298	gene.scaffold_14.g101.t:	OG0004676	713039..718640
Scaffold 91	406627-407543	HD016098	OG0007680	gi|817183740|ref|XP_012278017.1|	GATA zinc finger domain-containing protein 1 isoform X2 [Orussus ɛ	Expect = 1.600e-105	gene.scaffold_14.g102.t:	OG0007680	720734..721681
Scaffold 91	408455-411290	HD016099	OG0005425	gi|48142301|ref|XP_397320.1|	AP-3 complex subunit sigma-2 isoform X1 [Apis mellifera]	Expect = 8.100e-92	gene.scaffold_14.g103.t:	OG0005425	722419..724763
Scaffold 91	412851-415250	HD016100	OG0001553	gi|383857701|ref|XP_003704342.1	PREDICTED: beta-1,3-galactosyltransferase 1-like isoform X2 [Mega	Expect = 3.200e-143	NA		
Scaffold 91	418638-419902	HD016101	OG0008604	gi|345486223|ref|XP_003425426.1	PREDICTED: G patch domain-containing protein 4-like [Nasonia vitri	Expect = 1.400e-47	NA		
Scaffold 91	420807-422918	HD016102	OG0004778	gi|970907664|ref|XP_015119881.1|	PREDICTED: type 2 phosphatidylinositol 4,5-bisphosphate 4-phosph	Expect = 7.800e-127	NA		
Scaffold 91	425105..426659	HD016103	orphan	no hit			NA		
Scaffold 91	427639..428218	HD016104	orphan	no hit			NA		
Scaffold 91	429397..431144	HD016105	orphan	no hit			NA		

Scaffold 91	433475..434375	HD016106	orphan	no hit			NA		
Scaffold 91	435700..436543	HD016107	orphan	no hit			NA		
Scaffold 91	437892..438750	HD016108	orphan	no hit			NA		
Scaffold 91	440012..441810	HD016109	orphan	no hit			NA		
Scaffold 91	443803..449675	HD016110	orphan	no hit			NA		
Scaffold 91	450623..452672	HD016111	OG0005467	gi|987911334|ref|XP_015431390.1|	PREDICTED: DNA repair protein XRCC1 [Dufourea novaeangliae]	Expect = 5.400e-86	NA		
Scaffold 91	453857..454591	HD016112	orphan	IVSP_U1			NA		
Scaffold 91	///						NA		
Scaffold 91	467172..467876	HD016611	orphan	IVSP_U25			NA		
Scaffold 91	467893..468866	HD016610	OG0008504	no hit			NA		
Scaffold 91	470135..471607	HD016121	orphan	N-gene1_Hd15			NA		
Scaffold 91	474019..474459	HD016122	orphan	no hit			NA		
Scaffold 91	475549..482783	HD016123	OG0008504	gi|1000724119|ref|XP_015610535.1	uncharacterized protein LOC107275188 isoform X2 [Cephus cinctus	Expect = 2.200e-140	gene.scaffold_50.g34.t1.	OG0008504	228808..236437
Scaffold 91	485760..486020	HD016616	orphan	U1_Hd33					
Scaffold 91	487963..488427	HD016613	orphan	U2_Hd33					
Scaffold 91	487593..496279	HD016124	OG0008877	gi|1573754945|ref|XP_028042060.1	inter-alpha-trypsin inhibitor heavy chain H4-like [Bombyx mandarin	Expect = 1.400e-43	gene.scaffold_50.g35.t1	OG0008877	239447..245088
Scaffold 91	496775..497693	HD016125	OG0008877	gi|1185578680|ref|XP_020720113.1	uncharacterized protein LOC100648520 [Bombus terrestris]	Expect = 2.200e-27			
Scaffold 91	498816..502616	HD016126	OG0007035	gi|817052951|ref|XP_012259318.1|	tetratricopeptide repeat protein 27 [Athalia rosae]	Expect = 4.100e-262	gene.scaffold_50.g36.t1	OG0007035	245526..250163
Scaffold 91	503255..504795	HD016127	orphan	no hit			gene.scaffold_50.g37.t1	orphan	250869..252609
Scaffold 91	506500..510737	HD016128	OG0008077	gi|970916129|ref|XP_015124492.1|	PREDICTED: uncharacterized protein LOC107046395 [Diachasma all	Expect = 3.700e-255	gene.scaffold_50.g38.t1	OG0008077	253612..257408
Scaffold 91	512122..516356	HD016129	OG0004493	gi|1061111877|ref|XP_017881969.1	uridine-cytidine kinase-like 1 [Ceratina calcarata]	Expect = 1.600e-268	gene.scaffold_50.g39.t1	OG0004493	258014..261162
Scaffold 91	517628..518212	HD016130	OG0002787	gi|665791319|ref|XP_008543211.1|	PREDICTED: inosine triphosphate pyrophosphatase [Microplitis den	Expect = 2.700e-82	gene.scaffold_50.g40.t1	OG0002787	262054..262638
Scaffold 91	521509..534951	HD016131	OG0003558	gi|665791317|ref|XP_008543209.1|	PREDICTED: uncharacterized protein LOC103568231 [Microplitis de	Expect = 0.000e+0	NA		
Scaffold 91	536918..537427	HD016132	OG0000328	Vank1_Hd24			NA		
Scaffold 91	538200..539270	HD016133	OG0010755	Vinx1_Hd24			NA		
Scaffold 91	541304..542734	HD016134	orphan	Ngene-4			NA		
Scaffold 91	///						NA		
Scaffold 91	567663..567944	HD016614	orphan	Ngene-3			NA		
Scaffold 91	573680..574969	HD016150	orphan	N1_Hd29			NA		
Scaffold 91	579785..581438	HD016151	OG0001925	gi|1000751647|ref|XP_015598681.1	protein l(2)37Cc [Cephus cinctus] >gi|1000751649|ref|XP_015598	Expect = 7.900e-133	NA		
Scaffold 91	583666..596697	HD016152	OG0000557	gi|970889107|ref|XP_015109747.1|	PREDICTED: myosin-VIIa-like [Diachasma alloeum]	Expect = 0.000e+0	NA		
Scaffold 91	733490..737430	HD016153	OG0002720	gi|1061140064|ref|XP_017893504.1	transcription factor Sox-2 isoform X1 [Ceratina calcarata]	Expect = 3.700e-147	NA		

Genes in synteny blocks Figure 6C, b

Hd scaffold	Position in scaffold	Hd gene ID	Orthogroup #	Best Blast hit			Cs gene	Orthogroup	Position in scaffold
Scaffold 127	5695132..5698339	HD001703	OG0002287	gi|1615708828|gb|TGZ38293.1|	Uncharacterized protein DBV15_00393, partial [Temnothorax longi	Expect = 1.200e-89	gene.scaffold_134.g10.t	OG0002287	124421..126230
Scaffold 127	5698820..5699218	HD001704	OG0007747	gi|817198610|ref|XP_012274957.1|	proteasome assembly chaperone 4 isoform X1 [Orussus abietinus]	Expect = 8.200e-35	gene.scaffold_134.g11.t	OG0007747	128625..129023
Scaffold 127	5700119..5709232	HD001705	OG0007746	gi|1000763707|ref|XP_015604930.1	midasin [Cephus cinctus]	Expect = 0.000e+0	gene.scaffold_134.g12.t	OG0007746	129973..143060
Scaffold 127	5715296..5722376	HD001706	OG0005758	gi|1000763721|ref|XP_015604938.1	uncharacterized protein LOC107272369 isoform X1 [Cephus cinctus	Expect = 6.200e-245	gene.scaffold_134.g13.t	OG0005758	142507..148607
Scaffold 127	5745780..5748027	HD001707	orphan	no hit			NA		
Scaffold 127	5789342..5804341	HD001708	OG0010542	gi|1000763707|ref|XP_015604930.1	midasin [Cephus cinctus]	Expect = 0.000e+0	gene.scaffold_6042.g1.t	OG0010542	7898..20048
Scaffold 127	5804928..5806155	HD001709	OG0008637	gi|817050671|ref|XP_012269284.1|	28S ribosomal protein S15, mitochondrial [Athalia rosae]	Expect = 3.900e-66	gene.scaffold_6042.g3.t	OG0008637	20431..21692
Scaffold 127	5806461..5808045	HD001710	OG0008638	gi|1000725354|ref|XP_015585242.1	protein AATF [Cephus cinctus]	Expect = 1.900e-132	gene.scaffold_6042.g4.t	OG0008638	22007..23884
Scaffold 127	5809242..5809907	HD016565	orphan	Rep1_Hd6					
Scaffold 127	///						gene.scaffold_6042.g5.t	OG0000017	38407..41846
Scaffold 127	5818232..5818829	HD016570	orphan	U1.2_Hd6					
Scaffold 127	5853342..5855008	HD001713	OG0000907	gi|954550203|ref|XP_014598460.1|	PREDICTED: fibrillin-1-like [Polistes canadensis]	Expect = 2.700e-10			
Scaffold 127	5859163..5862434	HD001714	OG0000759	gi|766921548|ref|XP_011505297.1|	PREDICTED: major royal jelly protein 1-like isoform X1 [Ceratosolen	Expect = 8.700e-75	gene.scaffold_6042.g6.t	OG0000759	56163..59912
Scaffold 127	5868688..5896004	HD001715	OG0000907	gi|1477752320|ref|XP_006561358.2	fibrillin-2 isoform X1 [Apis mellifera]	Expect = 1.000e-135	gene.scaffold_6042.g7.t	OG0000907	67573..82296
							gene.scaffold_6042.g8.t	OG0000907	84612..89146
Scaffold 127	5899594..5902437	HD001716	OG0008827	gi|925679321|gb|KOX76257.1|	hypothetical protein WN51_11586 [Melipona quadrifasciata]	Expect = 1.300e-41	gene.scaffold_6042.g9.t	OG0008827	95154..98396
Scaffold 127	5907509..5931751	HD001717	OG0000907	gi|951521230|ref|XP_014484382.1|	PREDICTED: uncharacterized protein LOC106749449 isoform X1 [Di	Expect = 1.400e-276	gene.scaffold_6042.g11.	OG0000907	102320..114213
Scaffold 127	5934850..5939667	HD001718	OG0003835	gi|936686298|ref|XP_014221120.1|	protein henna isoform X1 [Trichogramma pretiosum]	Expect = 8.800e-249	gene.scaffold_6042.g12.	OG0003835	131205..134233

Scaffold 127!	5941301..5941799	HD001719	orphan	no hit		NA		
Scaffold 127!	5941600..5943451	HD001720	orphan	GlyPro1_Hd2		NA		
Scaffold 127!	///					NA		
Scaffold 127!	5952553..5953461	HD016569	orphan	SerThr_Hd2		NA		
Scaffold 127!	5954908..5957857	HD001723	OG0008292	gi|970914653|ref|XP_015123688.1|PREDICTED: vacuolar fusion protein MON1 homolog A [Diachasma ;	Expect = 1.500e-249	NA		
Scaffold 127!	5958966..5960611	HD001724	OG0008989	gi|1000737514|ref|XP_015591270.1 pre-mRNA-splicing factor 38 isoform X1 [Cephus cinctus]	Expect = 7.300e-111	NA		
Scaffold 127!	5963322..5967084	HD001725	OG0006130	gi|665789861|ref|XP_008560590.1 PREDICTED: lipoma HMGIC fusion partner-like 3 protein [Microplitis	Expect = 1.500e-148	NA		
Scaffold 127!	5970045..5989460	HD001726	OG0003941	gi|1000737447|ref|XP_015591233.1 paired box pox-neuro protein isoform X2 [Cephus cinctus]	Expect = 3.600e-166	NA		
Scaffold 127!	6031309..6031662	HD001727	orphan	no hit		NA		
Scaffold 127!	6039464..6041203	HD001728	OG0008922	no hit		NA		
Scaffold 127!	6047894..6053795	HD001729	OG0004544	gi|970914023|ref|XP_015123344.1|PREDICTED: neither inactivation nor afterpotential protein G-like [D	Expect = 1.100e-201	NA		
Scaffold 127!	6055912..6056223	HD001730	orphan	no hit		gene.scaffold_12.g37.t1	orphan	313226..314796
Scaffold 127!	6065301..6068372	HD001731	orphan	U1_Hd7		gene.scaffold_12.g36.t1	orphan	269465..272474
Scaffold 127!	6163755..6209923	HD001732	OG0001182	gi|665784719|ref|XP_008543429.1|PREDICTED: zinc finger protein 608-like [Microplitis demolitor]	Expect = 8.000e-240	gene.scaffold_12.g35.t1	OG0001182	266509..281996
Scaffold 127!	6215883..6220739	HD001733	OG0009154	gi|665785226|ref|XP_008546042.1|PREDICTED: focadhesin [Microplitis demolitor]	Expect = 3.500e-300	gene.scaffold_12.g34.t1	OG0009154	254503..262293
Scaffold 127!	6223570..6225534	HD001734	OG0005749	gi|759044886|ref|XP_011331377.1|uncharacterized protein C1orf43 homolog [Ooceraea biroi]	Expect = 6.200e-121	gene.scaffold_12.g33.t1	OG0005749	246626..248241
Scaffold 127!	6233178..6236130	HD001735	orphan	gi|815899824|ref|XP_012236112.1|RNA polymerase II elongation factor Ell [Bombus impatiens]	Expect = 2.200e-20			
Scaffold 127!	6237301..6258275	HD001736	OG0001096	gi|1000725348|ref|XP_015585239.1 NGFI-A-binding protein homolog isoform X1 [Cephus cinctus]	Expect = 1.200e-181	gene.scaffold_12.g32.t1	OG0001096	233201..241710
Scaffold 127!	6262652..6270854	HD001737	OG0001213	gi|817221046|ref|XP_012286061.1|girdin isoform X1 [Orussus abietinus]	Expect = 4.100e-256	gene.scaffold_12.g30.t1	OG0001213	210669..216809
Scaffold 127!	6273273..6277538	HD001738	OG0000750	gi|970914033|ref|XP_015123349.1|PREDICTED: E3 ubiquitin-protein ligase RNF126 [Diachasma alloeum	Expect = 5.100e-91	NA		
Scaffold 127!	6295663..6305891	HD001739	OG0001026	gi|805820120|ref|XP_003707425.2|PREDICTED: monocarboxylate transporter 10-like isoform X2 [Mega	Expect = 4.400e-278	NA		
Scaffold 127!	6326868..6327170	HD001740	orphan	no hit		NA		
Scaffold 127!	6327762..6333610	HD001741	OG0001259	gi|755965602|ref|XP_011305227.1|PREDICTED: uncharacterized protein LOC105267814 [Fopius arisanu	Expect = 3.100e-204	NA		
Scaffold 127!	6336069..6336941	HD001742	OG0008975	gi|826447337|ref|XP_012531547.1|cytochrome c oxidase assembly factor 5 [Monomorium pharaonis]	Expect = 9.100e-32	NA		
Scaffold 127!	6337655..6338948	HD001743	OG0007279	gi|970893534|ref|XP_015112156.1|PREDICTED: lysM and putative peptidoglycan-binding domain-conta	Expect = 1.000e-66	NA		
Scaffold 127!	///							
Scaffold 127!	6498766..6500773	HD001752	OG0001027	gi|1059871709|ref|XP_017790766.1 PREDICTED: uncharacterized protein LOC108572935 [Habropoda la	Expect = 1.300e-295	gene.scaffold_57.g37.t1	OG0001027	701460..703459
Scaffold 127!	6525619..6535728	HD001753	OG0008714	gi|1000769997|ref|XP_015608184.1 zwei Ig domain protein zig-8 isoform X1 [Cephus cinctus]	Expect = 1.300e-115	gene.scaffold_57.g35.t1	OG0008714	673695..676530
Scaffold 127!	6576893..6577922	HD001754	orphan	no hit		gene.scaffold_57.g34.t1	OG0008714	667397..668577
Scaffold 127!	6589791..6590075	HD001755	orphan	no hit				
Scaffold 127!	6609826..6610452	HD001756	OG0010758	no hit				
Scaffold 127!	6654784..6656225	HD001757	OG0010662	no hit				
Scaffold 127!	6674792..6680997	HD001758	OG0010538	gi|817211154|ref|XP_012281702.1|uncharacterized protein LOC105700447 [Orussus abietinus]	Expect = 4.400e-177	gene.scaffold_57.g33.t1	OG0010538	549863..558077
Scaffold 127!	6687225..6691596	HD001759	OG0000577	gi|665815849|ref|XP_008556602.1|PREDICTED: acylcarnitine hydrolase-like [Microplitis demolitor]	Expect = 1.100e-123	gene.scaffold_57.g32.t1	OG0000577	527847..544092
Scaffold 127!	6694467..6698473	HD001760	OG0000577	gi|665815849|ref|XP_008556602.1|PREDICTED: acylcarnitine hydrolase-like [Microplitis demolitor]	Expect = 2.300e-118			
Scaffold 127!	6713912..6715341	HD001761	OG0010178	gi|954544039|ref|XP_014610010.1|PREDICTED: UPF0602 protein C4orf47 homolog [Polistes canadensis	Expect = 2.700e-76	gene.scaffold_57.g31.t1	OG0010178	512556..513765
Scaffold 127!	6755517..6755963	HD001762	orphan	no hit		gene.scaffold_57.g29.t1	orphan	480378..482423
Scaffold 127!	6788558..6789189	HD001763	OG0000376	gi|998503073|ref|XP_015511626.1|PREDICTED: uncharacterized protein LOC107218308 [Neodiprion le	Expect = 2.500e-25	gene.scaffold_57.g28.t1	OG0000376	445783..447418
Scaffold 127548						gene.scaffold_57.g27.t1	orphan	394294..400573
Scaffold 127!	6832835..6833713	HD001764	orphan	IVSP_U29		gene.scaffold_57.g26.t1.	orphan	391820..393241
Scaffold 127!	///					///		
Scaffold 127!	6845548..6848646	HD001769	orphan	IVSP_U34		gene.scaffold_57.g24.t1.	orphan	383305..386526
Scaffold 127!	6856068..6858621	HD001770	OG0000376	gi|755932577|ref|XP_011314368.1|PREDICTED: lachesin-like [Fopius arisanus]	Expect = 2.700e-25	gene.scaffold_57.g22.t1	OG0000376	370164..371046
Scaffold 127!	6861330..6863003	HD001771	OG0001625	gi|1070588568|ref|XP_018406776.1 PREDICTED: uncharacterized protein LOC108771307 [Cyphomyrme:	Expect = 1.500e-186			

Genes in synteny blocks Figure 6C, c

Hd scaffold	Position in scaffold	Hd gene ID	Orthogroup #	Best Blast hit		Cs gene	Orthogroup :	Position in scaffold
Scaffold 127!	10649612..1065171(	HD002066	OG0004297	gi|815768838|ref|XP_012233244.1|PREDICTED: caspase-3 isoform X1 [Linepithema humile]	Expect = 2.800e-70	gene.scaffold_12.g65.t1	OG0004297	668906..679965
Scaffold 127!	10653145..1065624(	HD002067	OG0009466	gi|972201540|ref|XP_015182858.1|PREDICTED: sodium channel protein Nach-like isoform X1 [Polistes (	Expect = 6.900e-140	gene.scaffold_12.g66.t1	OG0009466	681861..685155
Scaffold 127!	10696314..1070282	HD002068	OG0002519	gi|1389785382|ref|XP_024946756.1 uncharacterized protein LOC107273655 isoform X4 [Cephus cinctus	Expect = 1.000e-157	gene.scaffold_12.g67.t1	OG0002519	733521..738350
Scaffold 127!	10713573..1071834	HD002069	OG0002011	gi|817182955|ref|XP_012273795.1|coiled-coil and C2 domain-containing protein 1-like isoform X2 [Oru	Expect = 2.300e-247	gene.scaffold_12.g68.t1	OG0002011	752453..758163
Scaffold 127!	10718988..1072124	HD002070	OG0008538	gi|817182522|ref|XP_012271486.1|V-type proton ATPase subunit d [Orussus abietinus]	Expect = 3.600e-199	gene.scaffold_12.g70.t1	OG0008538	758815..760937
Scaffold 127!	10721836..1072315!	HD002071	OG0004841	gi|817182518|ref|XP_012271468.1|probable tRNA(His) guanylyltransferase isoform X1 [Orussus abietir	Expect = 2.500e-102	gene.scaffold_12.g71.t1	OG0004841	761672..762420

Scaffold 127	10723636..1072892	HD002072	OG0002227	gi|1000768710|ref|XP_015607512.1	probable methyltransferase TARBP1 isoform X1 [Cephus cinctus]	Expect = 8.500e-229	gene.scaffold_6122.g8.t	OG0002227	84619..90498
Scaffold 127	10729352..1073001	HD002073	OG0006556	gi|970884085|ref|XP_015126663.1|	PREDICTED: ATP synthase subunit g, mitochondrial [Diachasma allo	Expect = 9.800e-33	gene.scaffold_6122.g9.t	OG0006556	90282..90940
Scaffold 127	10730306..1073170	HD002074	OG0008346	gi|817057993|ref|XP_012269928.1|	zinc finger protein 330 homolog [Athalia rosae]	Expect = 2.700e-140	gene.scaffold_6122.g10.	OG0008346	91228..92932
Scaffold 127	10732736..1073820	HD002075	OG0006263	gi|665809833|ref|XP_008553302.1|	PREDICTED: arrestin domain-containing protein 2 [Microplitis demc	Expect = 3.600e-183	gene.scaffold_6122.g11.	OG0006263	93959..96475
Scaffold 127	10740693..1074533	HD002076	OG0007281	gi|1389758539|ref|XP_024943813.1	synaptotagmin-4 isoform X2 [Cephus cinctus]	Expect = 4.500e-179	gene.scaffold_6122.g12.	OG0007281	101239..105178
Scaffold 127	10749875..1075318	HD002077	OG0007282	gi|970904570|ref|XP_015118179.1|	PREDICTED: dolichol kinase isoform X1 [Diachasma alloeum]	Expect = 2.900e-175	gene.scaffold_6122.g14.	OG0007282	110423..112817
Scaffold 127	10755559..1075930	HD002078	OG0005355	gi|936697175|ref|XP_014226574.1|	glucose-6-phosphate isomerase [Trichogramma pretiosum]	Expect = 5.700e-287	gene.scaffold_6122.g15.	OG0005355	116428..120182
Scaffold 127	10761570..1076280	HD002079	orphan	IVSP_U15					
Scaffold 127	///								
Scaffold 127	10785523..1078700	HD002089	orphan	IVSP_N-2					
Scaffold 127	10788460..1079089	HD002090	OG0005583	gi|1000723328|ref|XP_015610175.1	probable cysteine--tRNA ligase, mitochondrial [Cephus cinctus]	Expect = 7.000e-170	gene.scaffold_6122.g28.	OG0005583	158304..160353
Scaffold 127	10790972..1079279	HD002091	OG0008727	gi|1000723356|ref|XP_015610186.1	ribosome production factor 2 homolog [Cephus cinctus]	Expect = 5.600e-130	gene.scaffold_6122.g29.	OG0008727	160438..162016
Scaffold 127	10794756..1080633	HD002092	OG0000882	gi|820843698|ref|XP_012340057.1|	PREDICTED: protein polybromo-1 isoform X4 [Apis florea]	Expect = 0.000e+0	gene.scaffold_6122.g30.	OG0000882	163578..176041
Scaffold 127	10808631..1080918	HD002093	OG0004828	gi|795054949|ref|XP_011871354.1|	PREDICTED: succinate dehydrogenase assembly factor 4, mitochon	Expect = 4.300e-21	gene.scaffold_6122.g31.	OG0004828	178896..179652
Scaffold 127	10809957..1081106	HD002094	OG0006539	gi|925679306|gb|KOX76242.1|	GPI mannosyltransferase 2 [Melipona quadrifasciata]	Expect = 2.200e-60			
Scaffold 127	10816751..1084960	HD002095	OG0003603	gi|1000760603|ref|XP_015603308.1	angiopoietin-2 [Cephus cinctus] >gi|1389766659|ref|XP_0249446	Expect = 4.400e-295	gene.scaffold_6122.g32.	OG0003603	208607..221477
Scaffold 127	10837195..1085690	HD002096	OG0009828	gi|1070171260|ref|XP_018346064.1	PREDICTED: angiopoietin-4 isoform X2 [Trachymyrmex septentrion	Expect = 3.600e-220	gene.scaffold_6122.g33.	OG0009828	227997..231684
Scaffold 127	10859683..1086000	HD002097	orphan	no hit					
Scaffold 127	10860001..1086053	HD002098	orphan	IVSP_U35			gene.scaffold_6122.g34.	orphan	234803..235273
Scaffold 127	10861166..1086163	HD002099	orphan	IVSP_U36					
Scaffold 127	10862390..1086479	HD002100	OG0006143	gi|107268279|ref|XP_015596389.1|	PREDICTED: serine/threonine-protein kinase rio2 [Cephus cinctus]		gene.scaffold_6122.g37.	OG0006143	241011..243008
Scaffold 127	10866093..1087121	HD002101	OG0007135	gi|749759480|ref|XP_011141208.1|	sorbitol dehydrogenase [Harpegnathos saltator]	Expect = 5.900e-157	gene.scaffold_6122.g38.	OG0007135	243912..249324
Scaffold 127	10872479..1087731	HD002102	OG0007814	gi|1569271860|gb|QBB02001.1|	putative UPF0528 protein CG10038 [Cotesia chilonis]	Expect = 1.200e-113	gene.scaffold_6122.g39.	OG0007814	250807..256468
Scaffold 127	10921663..1092207	HD002103	orphan	no hit					
Scaffold 127	10970846..1103466	HD002104	OG0002894	gi|1494653095|gb|RLU26944.1|	hypothetical protein DMN91_000743 [Ooceraea biroi]	Expect = 1.500e-225	gene.scaffold_6122.g40.	OG0002894	361174..391485
Scaffold 127	10983515..1098646	HD002105	orphan	no hit			NA		
Scaffold 127	11051421..1105729	HD002106	OG0000642	gi|972199364|ref|XP_015181683.1|	PREDICTED: serine/threonine-protein kinase tricorner isoform X1 [F	Expect = 4.700e-266	gene.scaffold_6095.g2.t	OG0000642	36848..43783
Scaffold 127	11089871..1109165	HD002107	orphan	no hit			gene.scaffold_6095.g3.t	orphan	79134..81658
Scaffold 127	11110231..1111272	HD002108	OG0000107	gi|970917263|ref|XP_015125099.1|	PREDICTED: putative RNA-binding protein EEED8.10 [Diachasma all	Expect = 8.200e-156	gene.scaffold_6095.g5.t	OG0000107	105829..109141
Scaffold 127	11118585..1111968	HD002109	OG0001394	gi|1000769696|ref|XP_015608031.1	dual specificity mitogen-activated protein kinase kinase dSOR1 isof	Expect = 1.300e-106	gene.scaffold_6095.g6.t	OG0001394	113898..116393
Scaffold 127	11120053..1112313	HD002110	OG0000139	gi|156547818|ref|XP_001606363.1|	PREDICTED: sodium/potassium-transporting ATPase subunit alpha-	Expect = 0.000e+0	gene.scaffold_6095.g7.t	OG0000139	115279..118353
Scaffold 127	11123403..1112437	HD002111	OG0001394	gi|571503163|ref|XP_393416.2|	dual specificity mitogen-activated protein kinase kinase dSOR1 [Api	Expect = 1.100e-91	gene.scaffold_6095.g8.t	OG0001394	117396..119865
