# Supplementary material for: Genomic architecture of endogenous ichnoviruses reveals distinct evolutionary pathways leading to virus domestication in parasitic wasps
Source: BMC Biol. 2020 Jul 24;18:89. doi: 10.1186/s12915-020-00822-3 (PMC7379367; doi:10.1186/s12915-020-00822-3)
Supplement: Supplementary file 14 — Additional file 14: Characteristics of the libraries used for genome assembly. Table S14. Campoletis sonorensis. Table S15. Hyposoter didymator. [file 12915_2020_822_MOESM14_ESM.pdf]

Additional file 14. Characteristics of the libraries used for genome assembly.

Table S14. *Campoletis sonorensis* genome

	**Library ID**	**Insert size (bp)**	**Read length (bp)**	**Data (Gb)**	**Sequence depth (X)**
Paired-ends	CS_WGS_001	250	150	19.18	74.05

Table S15. *Hyposoter didymator* genome

	**Insert size (bp)**	**Read length (bp)**	**Data (Gb)**	**Sequence depth (X)**	**Experiment ID (NCBI SRA)**
Paired-ends	250	135_145	6.96	19.89	SRX7136286
	500	100_110	1.52	4.33	SRX7136287
	800	100	3.01	8.60	SRX7136288
Mate-pairs	2,000	100	7.93	22.66	SRX7136289
	5,000	100	5.20	14.85	SRX7136290
